# Sixteen new lung function signals identified through 1000 Genomes Project reference panel imputation

**DOI:** 10.1038/ncomms9658

**Published:** 2015-12-04

**Authors:** María Soler Artigas, Louise V. Wain, Suzanne Miller, Abdul Kader Kheirallah, Jennifer E. Huffman, Ioanna Ntalla, Nick Shrine, Ma'en Obeidat, Holly Trochet, Wendy L. McArdle, Alexessander Couto Alves, Jennie Hui, Jing Hua Zhao, Peter K. Joshi, Alexander Teumer, Eva Albrecht, Medea Imboden, Rajesh Rawal, Lorna M. Lopez, Jonathan Marten, Stefan Enroth, Ida Surakka, Ozren Polasek, Leo-Pekka Lyytikäinen, Raquel Granell, Pirro G. Hysi, Claudia Flexeder, Anubha Mahajan, John Beilby, Yohan Bossé, Corry-Anke Brandsma, Harry Campbell, Christian Gieger, Sven Gläser, Juan R. González, Harald Grallert, Chris J. Hammond, Sarah E. Harris, Anna-Liisa Hartikainen, Markku Heliövaara, John Henderson, Lynne Hocking, Momoko Horikoshi, Nina Hutri-Kähönen, Erik Ingelsson, Åsa Johansson, John P. Kemp, Ivana Kolcic, Ashish Kumar, Lars Lind, Erik Melén, Arthur W. Musk, Pau Navarro, David C. Nickle, Sandosh Padmanabhan, Olli T. Raitakari, Janina S. Ried, Samuli Ripatti, Holger Schulz, Robert A. Scott, Don D. Sin, John M. Starr, UK BiLEVE, Panos Deloukas, Anna L. Hansell, Richard Hubbard, Victoria E. Jackson, Jonathan Marchini, Ian Pavord, Neil C. Thomson, Eleftheria Zeggini, Ana Viñuela, Henry Völzke, Sarah H. Wild, Alan F. Wright, Tatijana Zemunik, Deborah L. Jarvis, Tim D. Spector, David M. Evans, Terho Lehtimäki, Veronique Vitart, Mika Kähönen, Ulf Gyllensten, Igor Rudan, Ian J. Deary, Stefan Karrasch, Nicole M. Probst-Hensch, Joachim Heinrich, Beate Stubbe, James F. Wilson, Nicholas J. Wareham, Alan L. James, Andrew P. Morris, Marjo-Riitta Jarvelin, Caroline Hayward, Ian Sayers, David P. Strachan, Ian P. Hall, Martin D. Tobin

**Affiliations:** 1 Genetic Epidemiology Group, Department of Health Sciences, University of Leicester, Leicester LE1 7RH, UK; 2 Division of Respiratory Medicine, Queen's Medical Centre, University of Nottingham, Nottingham NG7 2RD, UK; 3 MRC Human Genetics Unit, MRC Institute of Genetics and Molecular Medicine, University of Edinburgh, Edinburgh, Scotland EH8 9AD, UK; 4 University of British Columbia Centre for Heart Lung Innovation, St Paul's Hospital, Vancouver, British Columbia, Canada V6Z 1Y6; 5 Generation Scotland, A Collaboration between the University Medical Schools and NHS, Aberdeen, Dundee, Edinburgh, Glasgow EH4 2XU, UK; 6 School of Social and Community Medicine, University of Bristol, Bristol BS8 1TH, UK; 7 Department of Epidemiology and Biostatistics, MRC -PHE Centre for Environment & Health, School of Public Health, Imperial College London, London SW7 2AZ, UK; 8 Busselton Population Medical Research Institute, Busselton, Western Australia 6280, Australia; 9 PathWest Laboratory Medicine WA, Sir Charles Gairdner Hospital, Western Australia 6009, Australia; 10 School of Population Health, The University of Western Australia, Western Australia 6009, Australia; 11 School of Pathology and Laboratory Medicine, The University of Western Australia, Western Australia 6009, Australia; 12 MRC Epidemiology Unit, University of Cambridge School of Clinical Medicine, Institute of Metabolic Science, Cambridge Biomedical Campus, Cambridge CB2 0SL, UK; 13 Usher Institute of Population Health Sciences and Informatics, University of Edinburgh, Teviot Place, Edinburgh EH8 9AD, Scotland, UK; 14 University Medicine Greifswald, Community Medicine, SHIP—Clinical Epidemiological Research, Greifswald 17489, Germany; 15 Department for Genetics and Functional Genomics, Interfaculty Institute for Genetics and Functional Genomics, University Medicine Greifswald, Greifswald 17489, Germany; 16 Institute of Genetic Epidemiology, Helmholtz Zentrum München German Research Center for Environmental Health, Neuherberg D-85764, Germany; 17 Swiss Tropical and Public Health Institute, Basel 4051, Switzerland; 18 University of Basel, Basel 4001, Switzerland; 19 Research Unit of Molecular Epidemiology, Helmholtz Zentrum München, German Research Center for Environmental Health, Neuherberg D-85764, Germany; 20 Institute of Epidemiology II, Helmholtz Zentrum München German Research Center for Environmental Health, Neuherberg D-85764, Germany; 21 Centre for Cognitive Ageing and Cognitive Epidemiology, University of Edinburgh, Edinburgh EH8 9AD, UK; 22 Department of Psychology, University of Edinburgh, Edinburgh EH8 9AD, UK; 23 Department of Immunology, Genetics, and Pathology, Biomedical Center, SciLifeLab Uppsala, Uppsala University, Uppsala 751 23, Sweden; 24 Institute for Molecular Medicine Finland (FIMM), University of Helsinki, Helsinki FI-00014, Finland; 25 The National Institute for Health and Welfare (THL), Helsinki FI-00271, Finland; 26 Department of Public Health, Faculty of Medicine, University of Split, Split 21000, Croatia; 27 Department of Clinical Chemistry, Fimlab Laboratories, Tampere FI-33101, Finland; 28 Department of Clinical Chemistry, University of Tampere School of Medicine, Tampere FI-33520, Finland; 29 KCL Department of Twins Research and Genetic Epidemiology, King's College London, London WC2R 2LS, UK; 30 Institute of Epidemiology I, Helmholtz Zentrum München, German Research Center for Environmental Health, Neuherberg D-85764, Germany; 31 Wellcome Trust Centre for Human Genetics, University of Oxford, Oxford OX3 7BN, UK; 32 School of Pathology and Laboratory Medicine, The University of Western Australia, Western Australia 6009, Australia; 33 Department of Molecular Medicine, Institut Universitaire de Cardiologie et de Pneumologie de Québec, Laval University, Québec, Canada G1V 0A6; 34 Department of Pathology and Medical Biology, University of Groningen, University Medical Center Groningen, Groningen 9700, The Netherlands; 35 Department of Internal Medicine B, Pneumology, Cardiology, Intensive Care, Weaning, Field of Research: Pneumological Epidemiology, University Medicine Greifswald, Greifswald 17489, Germany; 36 Centre for Research in Environmental Epidemiology (CREAL), Barcelona E-08003, Spain; 37 CIBER Epidemiología y Salud Pública (CIBERESP), Madrid 28029, Spain; 38 Pompeu Fabra University (UPF), Barcelona 08002, Catalonia, Spain; 39 Centre for Genomic and Experimental Medicine, University of Edinburgh, Edinburgh EH8 9AD, UK; 40 Department of Obstetrics and Gynecology of Oulu University Hospital ,MRC of Oulu University, Oulu 90220, Finland; 41 Division of Applied Health Sciences, University of Aberdeen, Aberdeen, Scotland AB24 3FX, UK; 42 Oxford Centre for Diabetes, Endocrinology and Metabolism, University of Oxford, Oxford OX1 2JD, UK; 43 Department of Pediatrics, Tampere University Hospital, Tampere 33521, Finland; 44 Department of Pediatrics, University of Tampere School of Medicine, Tampere FI-33520, Finland; 45 Department of Medical Sciences, Molecular Epidemiology and Science for Life Laboratory, Uppsala University, Uppsala 751 23, Sweden; 46 Department of Medicine, Division of Cardiovascular Medicine, Stanford University School of Medicine, Stanford, California 94305, USA; 47 Uppsala Clinical Research Centre, Uppsala University, Uppsala 751 23, Sweden; 48 Diamantina Institute, Translational Research Institute, University of Queensland, Brisbane, Queensland QLD 4072, Australia; 49 MRC Integrative Epidemiology Unit, Bristol BS8 1TH, UK; 50 Institute of Environmental Medicine, Karolinska Institutet, Stockholm SE-171 7, Sweden; 51 Department of Medical Sciences, Uppsala University, Uppsala 751 23, Sweden; 52 Institute of Environmental Medicine, Karolinska Institutet and Sachs' Children's Hospital, Stockholm SE-171 7, Sweden; 53 Department of Respiratory Medicine, Sir Charles Gairdner Hospital, Western Australia 6009, Australia; 54 School of Medicine and Pharmacology, The University of Western Australia, Western Australia 6009, Australia; 55 Genetics and Pharmacogenomics, Merck Research Labs, Boston, Massachusetts 02115, USA; 56 Division of Cardiovascular and Medical Sciences, University of Glasgow, Glasgow G12 8TA, Scotland, UK; 57 Department of Clinical Physiology and Nuclear Medicine, Turku University Hospital, Turku 20520, Finland; 58 Research Centre of Applied and Preventive Cardiovascular Medicine, University of Turku, Turku 20014, Finland; 59 Department of Public Health, University of Helsinki, Helsinki FI-00014, Finland; 60 Department of Human Genomics, Wellcome Trust Sanger Institute, Hinxton, Cambridge CB10 1SA, UK; 61 Comprehensive Pneumology Center Munich (CPC-M), Member of the German Center for Lung Research, Munich 85764, Germany; 62 Respiratory Division, University of British Columbia, Vancouver, British Columbia, Canada V6T 1Z4; 63 Alzheimer Scotland Research Centre, University of Edinburgh, Edinburgh EH8 9AD, UK; 64 Department of Medical Biology, Faculty of Medicine, University of Split, Split 21000, Croatia; 65 Respiratory Epidemiology and Public Health, Imperial College London, London SW7 2AZ, UK; 66 MRC Health Protection Agency (HPA) Centre for Environment and Health, Imperial College London, London SW7 2AZ, UK; 67 Department of Clinical Physiology, University of Tampere and Tampere University Hospital, Tampere 33521, Finland; 68 Centre for Population Health Sciences, Medical School, University of Edinburgh, Edinburgh EH8 9AD, Scotland, UK; 69 Institute of General Practice, University Hospital Klinikum rechts der Isar, Technische Universität München, Munich D - 81675, Germany; 70 Institute and Outpatient Clinic for Occupational, Social and Environmental Medicine, Ludwig-Maximilians-Universität, Munich 80539, Germany; 71 University Hospital Munich, Institute and Outpatient Clinic for Occupational, Social and Environmental Medicine, Ludwig-Maximilian University Munich, Munich 80539, Germany; 72 Department of Pulmonary Physiology and Sleep Medicine, Sir Charles Gairdner Hospital, Western Australia 6009, Australia; 73 Department of Biostatistics, University of Liverpool, Liverpool L69 7ZX, UK; 74 Estonian Genome Centre, University of Tartu, Tartu 50090, Estonia; 75 Center for Life Course Epidemiology, Faculty of Medicine, P.O.Box 5000, FI-90014 University of Oulu, Oulu FI-01051, Finland; 76 Biocenter Oulu, P.O.Box 5000, Aapistie 5A, FI-90014 University of Oulu, Oulu FI-01051, Finland; 77 Unit of Primary Care, Oulu University Hospital, Kajaanintie 50, P.O.Box 20, FI-90220 Oulu, 90029 OYS, Finland; 78 Population Health Research Institute, St George's, University of London, Cranmer Terrace, London WC1B 5DN, UK; 79 National Institute for Health Research (NIHR) Leicester Respiratory Biomedical Research Unit, Glenfield Hospital, Leicester LE3 9QP, UK; 80 William Harvey Research Institute, Barts and The London School of Medicine and Dentistry, Queen Mary University London, London E1 4NS, UK.; 81 UK Small Area Health Statistics Unit, MRC-PHE Centre for Environment and Health, School of Public Health, Imperial College London, London SW7 2AZ, UK.; 82 School of Medicine, University of Nottingham, Nottingham NG7 2RD, UK.; 83 Department of Health Sciences, University of Leicester, Leicester LE1 7RH, UK.; 84 Department of Statistics, University of Oxford, Oxford OX1 2JD, UK.; 85 Respiratory Medicine, University of Oxford, Oxford OX1 2JD, UK.; 86 Institute of Infection, Immunity & Inflammation, University of Glasgow, Glasgow G12 8TA, UK.; 87 Wellcome Trust Sanger Institute, Hinxton, Cambridgeshire CB10 1SA, UK.

## Abstract

Lung function measures are used in the diagnosis of chronic obstructive pulmonary disease. In 38,199 European ancestry individuals, we studied genome-wide association of forced expiratory volume in 1 s (FEV_1_), forced vital capacity (FVC) and FEV_1_/FVC with 1000 Genomes Project (phase 1)-imputed genotypes and followed up top associations in 54,550 Europeans. We identify 14 novel loci (*P*<5 × 10^−8^) in or near *ENSA*, *RNU5F-1*, *KCNS3*, *AK097794*, *ASTN2*, *LHX3*, *CCDC91*, *TBX3*, *TRIP11*, *RIN3*, *TEKT5*, *LTBP4*, *MN1* and *AP1S2*, and two novel signals at known loci *NPNT* and *GPR126*, providing a basis for new understanding of the genetic determinants of these traits and pulmonary diseases in which they are altered.

## Abstract (web summary)

Genetic and environmental factors impact on lung function, important in the diagnosis of pulmonary diseases. Here the authors use imputation of genotypes to the 1000 Genomes Project reference panel to identify novel, low frequency variants associated with lung function.

## Graphical abstract

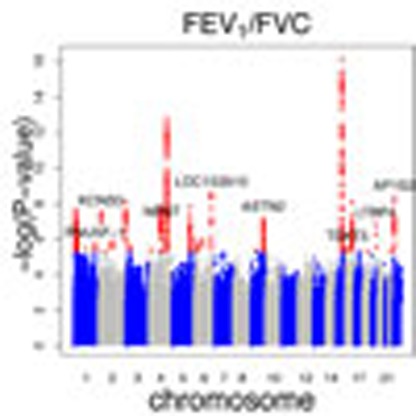

Lung function, as measured by spirometry, predicts morbidity and mortality^1^,^2^. Altered lung function is a key criterion for the diagnosis of chronic obstructive pulmonary disease (COPD), a leading cause of death worldwide^3^,^4^. The ratio of forced expiratory volume in 1 s (FEV_1_) over forced vital capacity (FVC) defines patients with airflow obstruction, while FEV_1_ is used to assess the severity of the obstruction. Reduced FVC values are seen in restrictive lung diseases such as pulmonary fibrosis^5^. While environmental risk factors, such as tobacco smoking or air pollution, play a significant role in determining lung function^6^,^7^, genetic factors are also important contributors, with estimates of heritability ranging between 39 and 54% (refs 8, 9).

Genome-wide association studies (GWAS) of around 2.5 million common (minor allele frequency (MAF)>5%) single-nucleotide polymorphisms (SNPs) in Europeans have identified 32 loci associated with lung function at genome-wide significance level (*P*<5 × 10^−8^)^10^,^11^,^12^,^13^,^14^. However, as for other complex traits^15^,^16^, these loci only explain a limited proportion of the heritability^11^,^13^. Among explanations for the ‘missing heritability' are a large number of, as yet, undetected common variants with modest effect sizes, in addition to low-frequency (1%<MAF≤5%) and rare (MAF≤1%) variants with larger effect sizes^16^,^17^. Of particular relevance to low-frequency variants, phase 1 of the 1000 Genomes Project^18^ sequenced 1,092 individuals from 14 populations, providing an imputation reference panel of ∼38 million SNPs and 1.4 million indels, including autosomal and X chromosome variants.

The aim of the current study, undertaken within the SpiroMeta consortium, was to improve coverage of low-frequency variants and detect novel loci associated with lung function by undertaking imputation of GWAS data to the 1000 Genomes Project^18^ Phase-1 reference panel in 38,199 individuals of European ancestry. We meta-analysed GWAS results across 17 studies and followed up the most significant associations with *in silico* data in up to 54,550 Europeans. We identify 14 new loci associated with lung function at genome-wide significance level, and novel distinct signals at two previously reported loci. These include two low-frequency variant association signals, which seem to be explained by non-synonymous SNPs. The results of these analyses implicate both previously considered and novel mechanisms influencing lung function.

## Results

**Figure 1:**
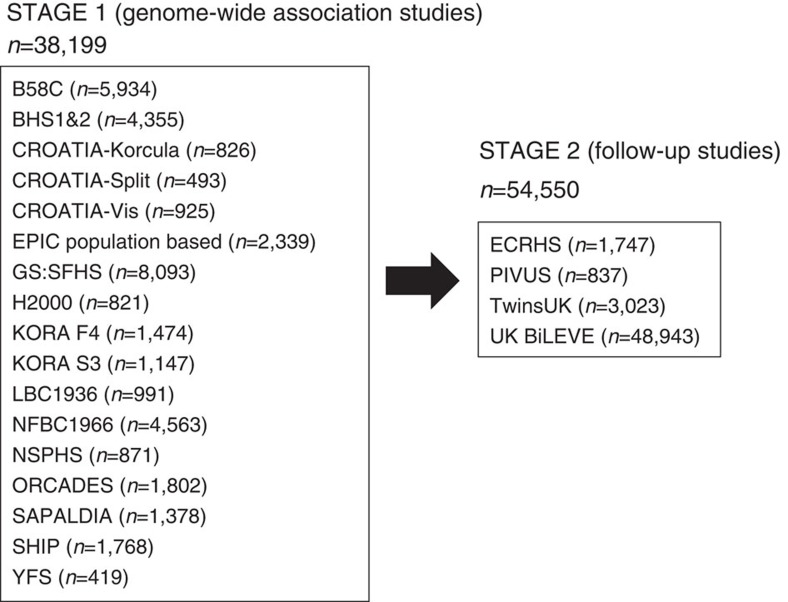
Study design for autosomal chromosome analyses. The discovery stage (stage 1) included 17 studies and 38,199 individuals. Fifty-five variants were followed up in stage 2, which comprised four studies and 54,550 individuals.

We undertook a meta-analysis of 17 GWAS imputed using the 1000 Genomes Project^18^ Phase-1 reference panel in a study of 38,199 individuals of European ancestry in stage 1 (Fig. 1), of which 19,532 were individuals not included in the discovery stage of previous meta-analyses of lung GWAS^10^,^11^,^12^,^13^. Characteristics of cohort participants, genotyping and imputation are shown in Supplementary Table 1. Each study adjusted FEV_1_, FEV_1_/FVC and FVC, for age, age^2^, sex, height and principal components for population structure, separately for never and ever smokers. Fourteen studies additionally undertook analyses for X chromosome variants (33,009 individuals, Supplementary Fig. 1 and Methods). Inverse normally transformed residuals were then used for association testing within each smoking stratum, assuming an additive genetic effect. Within each study, we combined smoking strata association summary statistics using inverse variance-weighted fixed-effects meta-analysis, and applied genomic control^19^ to account for residual population structure not accounted for by principal components. We subsequently combined study-specific estimates across studies using inverse variance weighing, and applied genomic control^19^ after fixed-effects meta-analysis. The genomic inflation factor across autosomal variants was 1.03 for each of the three traits, and across X chromosome variants was 1.04 for FEV_1_ and 1.00 for FEV_1_/FVC and FVC. Quantile–quantile plots are presented in Supplementary Fig. 2a. Variants with effective sample sizes (*N* effective, product of sample size and imputation quality summed across studies) <70% were filtered out, and a total of 8,694,268 variants were included in this genome-wide study.

**Figure 2:**
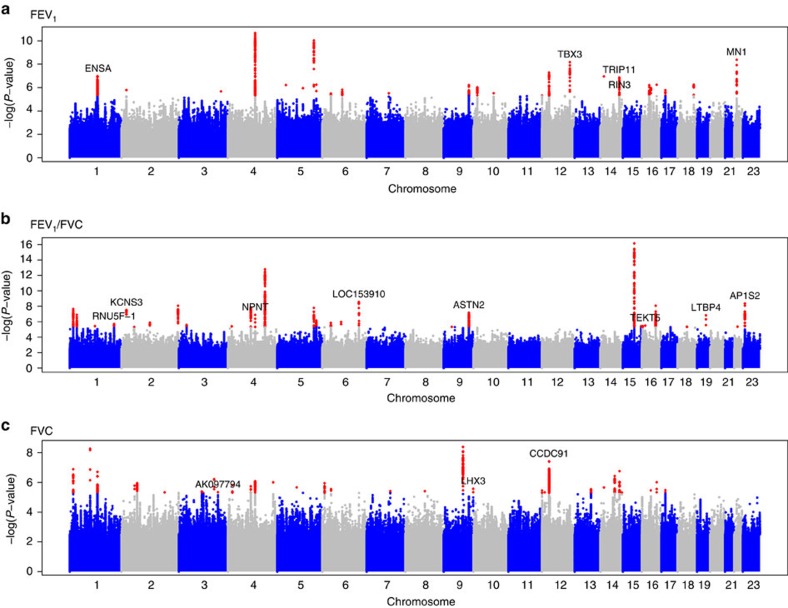
Manhattan plots for association results. (**a**) FEV_1_, (**b**) FEV_1_/FVC and (**c**) FVC. Manhattan plots ordered by chromosome and position for stage-1 results. Variants with *P*<5 × 10^−6^ are indicated in red. Novel signals that reached genome-wide significance after meta-analysing stage 1 and stage 2 are labelled with the nearest gene. Only variants with *N* effective ⩾70% are presented here.

Forty-eight SNPs and seven indels in independent autosomal chromosome regions (±500 kb either side of sentinel variant) with stage 1 *P*<5 × 10^−6^ were followed up in stage 2 using *in silico* data from four studies comprising 54,550 individuals (Fig. 1; Supplementary Table 2). One SNP on the X chromosome also met these criteria and was followed up in a subset of three studies comprising 52,359 individuals (Supplementary Fig. 1; Supplementary Table 2). Characteristics of follow-up (stage 2) cohort participants, genotyping and imputation are shown in Supplementary Table 1. Stage-2 studies adjusted the traits for age, age^2^, sex, height and principal components to account for population structure and ever-smoking status, and also undertook association testing on the inverse normally transformed residuals assuming additive genetic effects. Stage-2 estimates were combined across studies, and then with stage-1 estimates, using inverse variance-weighted fixed-effects meta-analysis. Thirteen SNPs and three indels, each representing new signals of association, met a genome-wide significance threshold corrected for multiple testing (*P*<5 × 10^−8^) after combining stage-1 and stage-2 results (Table 1; Fig. 2), of which 10 SNPs and three indels achieved independent replication meeting a Bonferroni-corrected threshold for 56 tests (*P*<8.93 × 10^−4^) in stage 2 alone.

### Sixteen novel association signals for FEV_1_, FEV_1_/FVC and FVC

Of the 16 novel signals reaching genome-wide significance, two represent distinct new signals for FEV_1_/FVC in previously reported loci^10^,^12^ (stage-1 *P* value conditioned on previously reported variant <5 × 10^−6^). Among the remaining 14, five new loci were identified for FEV_1_, six new loci for FEV_1_/FVC and three new loci for FVC (Table 1). The sentinel variants at the 16 loci were in or near the following genes: *MCL1-ENSA (*1q21.3), *LYPLAL1-RNU5F-1* (1q41), *KCNS3-NT5C1B* (2p24.2), *AK097794* (3q25.32), *NPNT* (4q24), *GPR126-LOC153910* (6q24.1), *ASTN2* (9q33.1), *LHX3* (9q33.1), *PTHLH-CCDC91* (12p11.22), *TBX3* (12q24.21), *TRIP11* (14q32.12), *RIN3* (14q32.12), *EMP2-TEKT5* (16p13.13), *LTBP4* (19q13.2), *MIAT-MN1* (22q12.1) and on chromosome X, *AP1S2-GRPR* (Xp22.2) (Supplementary Fig. 2b,c). To gain further insight into the associated variants, we assessed whether the novel sentinel variants, or their proxies, were associated with gene expression in lung tissues^20^ and blood^21^ (Methods, Supplementary Methods; Supplementary Table 3a) or were in DNase hypersensitivity sites^22^ in relevant cell types (Methods; Supplementary Table 3b). For relevant genes, we investigated RNA-seq splice isoforms in human bronchial epithelial cells (Supplementary Methods; Supplementary Fig. 3), searched for evidence of protein expression in the respiratory system^23^ (Supplementary Table 3c), assessed differential expression across the pseudoglandular and canalicular stages of fetal human lung development (Methods; Supplementary Table 3d) and assessed evidence for differences in gene expression in bronchial epithelial brush samples from COPD cases and smoking controls (Methods; Supplementary Table 3e).

The two novel signals in known loci were the strongest (*P*<5 × 10^−23^) association signals after meta-analysing stage 1 and 2. The strongest signal was for a low-frequency SNP near *GPR126* (rs148274477, MAF=2.4%, intergenic on chromosome 6) associated with FEV_1_/FVC (*P*=9.6 × 10^−26^, Table 1) and in high linkage disequilibrium (LD, *r*^2^=0.85) with a missense variant (rs17280293 (Ser123Gly), Supplementary Table 3f) in *GPR126*, but distinct from the previously reported signal for FEV_1_/FVC in this region^10^,^13^ (stage-1 *P* value for rs148274477 conditioning on rs3817928 (ref. 10) and rs262129 (ref. 13)=1.86 × 10^−7^, unconditional stage-1 *P*=2.68 × 10^−9^). *GPR126* encodes a G-protein-coupled receptor and is expressed in adult and fetal lung tissue^24^,^25^ (Supplementary Table 3d). Other studies have shown that *GPR126* is required for mice embryonic viability and cardiovascular development^26^, and that *GPR126* is expressed in adult mice lung^27^. More recently, GPR126 has been shown to bind type-IV collagen, a major collagen in the lung, leading to cAMP signalling^28^.

The second strongest signal (*P*=1.5 × 10^−23^, Table 1) was an intronic SNP (rs6856422) in *NPNT* on chromosome 4 associated with FEV_1_/FVC, distinct from the previously discovered signal for FEV_1_ in this region^10^,^12^,^13^. The stage-1 *P* value for this variant conditioned on the previously reported sentinel SNPs (rs17036341 (ref. 10) and rs10516526 (refs 12, 13)) and on the sentinel SNP for FEV_1_ in this analysis (rs12374256, *INTS12* intron) was 4.7 × 10^−6^ (unconditional stage-1 *P*=1.30 × 10^−7^). Proxies of the sentinel SNP were associated with expression of *INTS12* and *GSTCD* in blood (Supplementary Table 3a). *INTS12*, *GSTCD* and *NPNT* are contiguously positioned at 4q24, and are all expressed in adult and fetal lung tissues (Supplementary Table 3c,d). Our previous work characterizing *GSTCD* and *INTS12* showed that they are oppositely transcribed genes that are to some extent co-ordinately regulated, although while *GSTCD* expression in human lung tissue is ubiquitous, *INTS12* expression was predominantly in the nucleus of epithelial cells and pneumocytes^29^.

Among the 14 novel loci, six novel loci were associated with FEV_1_/FVC. One of them was a low-frequency variant (rs113473882, intronic in *LTBP4* on chromosome 19, MAF=1.5%, Table 1) in almost complete LD (*r*^2^=0.99) with a missense variant (rs34093919, Asp752Asn, Supplementary Table 3f) in *LTBP4*, which encodes a protein that binds transforming growth factor beta (TGFβ) as it is secreted and targeted to the extracellular matrix. Mice deficient in ltbp4 displayed defects in lung septation and elastogenesis, which may be TGFβ2 and fibulin-5 dependent^30^, and disruption of this gene in mice led to abnormal lung development, cardiomyopathy and colorectal cancer^31^. Variants near *LTBP4*, uncorrelated (*r*^2^<0.05) with the sentinel SNP we report here, have been associated with COPD^32^ and smoking behaviour^33^. A further novel FEV_1_/FVC locus mapping near *AP1S2* is the first to be reported for lung function on the X chromosome; sentinel SNP (rs7050036, intergenic) proxies were associated with the expression of *AP1S2* and *ZRSR2* in lung tissue (Supplementary Table 3a). Other new loci for FEV_1_/FVC were in or near *KCNS3* (2p24.2), *ASTN2* (9q33.1), *RNU5F-1* (1q41) and *TEKT5* (16p13.13).

The strongest signal for FEV_1_ in a novel locus was upstream of *TBX3* on chromosome 12 (Table 1); *TBX3* is involved in the TGFβ1 signalling pathway^34^. At a second novel locus for FEV_1_ (rs7155279, *TRIP11* intron on chromosome 14, Table 1), proxies of the sentinel variant were associated with lung and blood expression of *TRIP11*. *TRIP11* encodes a protein associated with the Golgi apparatus^35^. In the lung, rs7155279 showed strongest association with expression of *ATXN3* (Supplementary Table 3a), which encodes ataxin 3, a deubiquitinating enzyme. Expanded trinucleotide repeats in *ATXN3* cause spinocerebellar ataxia-3 (ref. 36). In blood, a proxy (*r*^2^=0.94) for rs7155279 showed strong association (*P*=3 × 10^−34^, Supplementary Table 3a) with the expression of *FBLN5.* Fibulin-5 was shown to be implicated in tissue repair in COPD^37^ and elastogenesis and lung development^30^. A third signal for FEV_1_ was a missense variant (rs117068593, Arg279Cys, Supplementary Table 3f) in *RIN3* on chromosome 14 (Table 1), which was ∼632 kb from the *TRIP11* sentinel SNP (rs7155279) and independent from it (*r*^2^=8.84 × 10^−5^). Although this is the first report of association of a *RIN3* variant with lung function, a correlated variant (rs754388, *r*^2^=0.99) was recently associated with moderate to severe COPD, although the association did not replicate in an independent study^38^. In a fourth novel region for FEV_1_, on chromosome 1, a sentinel SNP, rs6681426, ∼8 kb downstream of *ENSA* (Table 1) and a second signal ∼700 kb apart (rs4926386, Supplementary Table 2a) were both associated with *ARNT* expression in lung (Supplementary Table 3a). *ARNT* is differentially expressed during fetal lung development (Supplementary Table 3d) and acts as a co-factor for transcriptional regulation by hypoxia-inducible factor 1 during lung development^39^ and may regulate cytokine responses^40^. SNP rs6681426 was also associated with the expression of *LASS2* (also known as *CERS2*) in lung tissue (Supplementary Table 3a); lass2 knock-out mice develop lung inflammation and airway obstruction^41^. The other new locus for FEV_1_ was near *MN1* (22q12.1).

All three novel loci for FVC had sentinel variants or close proxies associated with expression of a nearby gene in lung, implicating *CCDC91*, *MLF1* and *QSOX2*, located on chromosomes 12, 3 and 9, respectively. The putative function of the key genes in each of the two known and 14 novel loci for FEV_1_, FEV_1_/FVC and FVC are summarized in Supplementary Table 4.

### Functional characterization of novel signals

The protein products of genes nearest to the sentinel variant of novel signals for lung function were expressed in bronchial epithelial cells, pneumocytes or lung macrophages (Supplementary Table 3c). Among the 16 novel signals of association with lung function, sentinel variants or close proxies were *cis* expression quantitative trait loci (eQTLs) in lung for *ARNT*, *MLF1*, *QSOX2*, *CCDC91* and *ATXN3* (Table 1; Supplementary Table 3a), and in eight loci the sentinel variant or at least one strong proxy (*r*^2^>0.8) was in a DNase hypersensitivity site in a cell type potentially relevant to lung function (in or near *ENSA*, *RNU5F-1*, *ASTN2*, *CCDC91*, *TBX3*, *RIN3*, *TEKT5* and *MN1*, Supplementary Table 3b). The sentinel variant association was explained (conditional *P*>0.01) by a missense variant in each of the two novel signals in which we detected a low-frequency sentinel variant (near *GPR126* and in *LTBP4*), and was explained in four of the remaining novel signals by a putatively functional variant (in or near *ENSA*, *AK097794*, *TEKT5* and *MN1*, Supplementary Table 3f and Methods). Genes in four of the novel loci showed differential expression across the pseudoglandular and canalicular stages of fetal lung development, particularly *EMP2* (Supplementary Table 3d). *MLF1* and *ATXN3* showed differences in expression levels in bronchial brushings between COPD cases and controls (Supplementary Table 3e). We detected novel splice isoforms of >20% abundance for *GFM1*, *TRIM32*, *LTBP4* and *MN1* in human bronchial epithelial cells (Supplementary Fig. 3; Supplementary Methods).

### Association in children

To assess whether the 16 new sentinel variants associated with lung, function in adults may act through an effect on lung development, we assessed their association in the ALSPAC study^42^ that includes 5,062 children (Supplementary Table 5a). Eleven of the 16 sentinel variants showed consistent directions of effect in adults and children. The association with FVC of variant rs6441207 on chromosome 3 in the noncoding RNA *AK097794* exceeded a Bonferroni-corrected threshold for 16 tests (Supplementary Table 5a).

### Association with smoking and gene by smoking interaction

The 16 new variants had consistent effect sizes in never smokers and ever smokers, and no gene–smoking interaction (*P*>0.05) in stage 1 (Supplementary Table 5b). We found no evidence that any of these signals were driven by smoking behaviour. Only the two-base-pair insertion on chromosome 1 (rs201204531) revealed an association (*P*=1.5 × 10^−3^) with smoking behaviour (heavy- versus never-smoking status) that met a Bonferroni-corrected threshold for 16 tests (Supplementary Table 5c). However, this variant also showed an association with FEV_1_/FVC in never smokers, and the allele associated with higher likelihood of being a smoker was associated with increased FEV_1_/FVC (Supplementary Table 5b,c).

### Associations with other traits

We queried the GWAS catalog^43^ for variants in 2-Mb regions centred on the sentinel variant for the 16 loci (Supplementary Table 5d). Five loci contained variants associated with height^44^,^45^,^46^ (Supplementary Table 5d). In the *GPR126* and *LHX3* loci, the previously reported height variants were not correlated (*r*^2^<0.2) with the lung function variants reported here. In the *AK097794*, *CCDC91* and *TRIP11* loci, the variants associated with height were correlated (*r*^2^>0.3) with the lung function sentinel variants, but the alleles associated with reduced height were associated with increased FEV_1_ or FVC. Associations with other traits have been reported for variants in LD (*r*^2^>0.3) with sentinel variants in regions of *RIN3* (Paget's disease^47^ and bone mineral density^48^), *ENSA* (body fat mass^49^ and melanoma^50^) and *LHX3* (thyroid hormone levels^51^). None of the novel signals relate to known asthma loci, and the association findings were consistent after removing individuals with asthma (Supplementary Fig. 4).

### Genetic architecture of lung function traits

**Figure 3:**
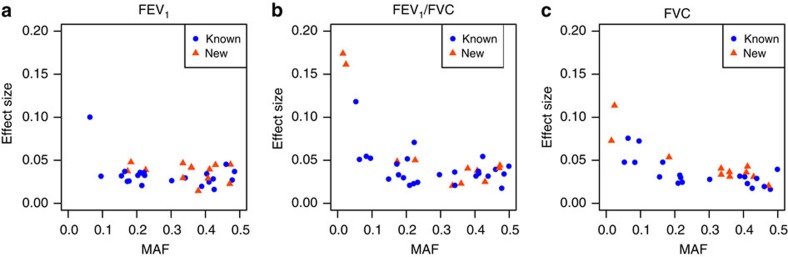
Minor allele frequency against effect-size plots (**a**) FEV_1_, (**b**) FEV_1_/FVC and (**c**) FVC. MAF is plotted against stage-1 effect sizes for variants within the 33 known^10^,^11^,^12^,^13^,^14^ and the 16 new signals, which had stage-1 *P*<0.05 for association with FEV_1_, FEV_1_/FVC and FVC separately. Known signals are represented with blue circles and new signals are represented with orange triangles.

The proportion of the additive polygenic variance explained by the 49 signals discovered to date (Supplementary Table 6), including new and previously reported signals^10^,^11^,^12^,^13^,^14^ is 4.0% for FEV_1_, 5.4% for FEV_1_/FVC and 3.20% for FVC (Supplementary Table 7). These estimates are likely upper bounds on the proportion of the variance explained due to the winner's curse bias. Across the 49 signals, we observed larger effect sizes for associations with lower-frequency variants (Fig. 3), supporting the hypothesis that lower-frequency variants will contribute to explaining the missing heritability^16^.

We examined the increase in coverage of low-frequency and common variants by the 1000 Genomes Project reference panel, compared with the HapMap imputation reference panel, at both the novel and previously reported loci (Supplementary Fig. 5a). The two association signals where the 1000 Genomes sentinel variants had low MAF (<5%), were not present when restricting the results only to variants that could be imputed using the HapMap imputation panel (rs113473882 and rs148274477 in Supplementary Fig. 5a).

For each of the 32 previously discovered regions^10^,^11^,^12^,^13^,^14^, we identified the most strongly associated variant present on the 1000 Genomes Project^18^ reference panel and the most strongly associated variant present on the HapMap reference panel using stage-1 results, and compared the stage-1 MAFs between these two groups of variants. The 1000 Genomes sentinel variants in or near *GPR126* (rs148274477), *TGFB2* (rs147187942) and *MMP15* (rs150232756) had MAFs that were more than twofold lower than the HapMap sentinel variant MAFs (Supplementary Fig. 5b) and were statistically independent (*r*^2^≤0.06) from the previously discovered HapMap-imputed sentinel variants^13^. The *GPR126* 1000 Genomes-imputed sentinel was described above as one of the 16 new signals. We tested the association of the 1000 Genomes-imputed sentinel variants near *TGFB2* and *MMP15* in UK BiLEVE (Supplementary Table 8), and found supportive evidence of association for the signal near *TGFB2* (rs147187942, MAF=9%, *P*=5.7 × 10^−3^).

### Pathway analyses

We undertook a pathway analysis using MAGENTA v2 (ref. 52) and stage-1 genome-wide results for FEV_1_, FEV_1_/FVC and FVC (Supplementary Methods). For FVC, the platelet-derived growth factor signalling, and the chromatin-packaging and -remodelling pathways were significant (*P*=2 × 10^−4^, false discovery rate (FDR)<0.3% and *P*=1.82 × 10^−4^, FDR<4%, respectively) (Supplementary Table 9).

## Discussion

In this study, we aimed to improve coverage of low-frequency variants and detect novel loci associated with lung function, by undertaking imputation of GWAS data in 17 studies and 38,199 individuals to the 1000 Genomes Project^18^ reference panel, and by following up the most significant signals in an additional 54,550 individuals. Overall, 16 new association signals attained a genome-wide significance threshold corrected for multiple testing (*P*<5 × 10^−8^) after meta-analysing stage 1 and stage 2, including 15 autosomal and one X chromosome signal. While two of the new findings relate to novel signals for FEV_1_/FVC in previously reported regions^10^,^12^, five new loci were identified for FEV_1_, six new loci for FEV_1_/FVC and three new loci for FVC. Including the 16 signals discovered in these analyses, the number of lung function signals discovered to date is 49 (refs 10, 11, 12, 13, 14), and they jointly explain a modest proportion of the additive polygenic variance (4.0% for FEV_1_, 5.4% for FEV_1_/FVC and 3.2% for FVC).

Some of the 49 distinct lung function signals^10^,^11^,^12^,^13^,^14^ seem to cluster close to each other. If we define regions as 500 kb either side of the sentinel variants, there are three regions that each include two distinct signals (in or near *INTS12-GSTCD*-*NPNT*, *GPR126* and *PTCH1* (refs 10, 12)), so that the 49 signals would map to 46 loci. If we use a wider definition of region (1,000 kb either side of the sentinel), there are four regions that each include two distinct signals (in or near *INTS12-GSTCD*-*NPNT*, *GPR126*, *PTCH1* (refs 10, 12) and *TRIP11-RIN3*). In addition, the human leukocyte antigen region on chromosome 6 includes three distinct signals (in or near ZKSCAN3-*NCR3*-*AGER*^10^,^12^,^13^) within 3.8 Mb. Furthermore, we have shown evidence of an additional signal in the *TGFB2* region, and the new lung function signal in *LTBP4* lies 179 kb away from a known COPD signal^32^. These findings are consistent with reports from very large studies of height and lipids^53^,^54^, which report multiple signals in associated regions, and highlight the importance of taking into account LD between variants to improve our understanding of known regions. Multiple signals within known regions are likely to explain some of the hidden heritability of these traits.

To identify pathways relevant to lung function, we undertook additional analyses using MAGENTA, which have implicated pathways for platelet-derived growth factor signalling and chromatin-packaging and -remodelling. Independent analyses undertaken in a concurrent study by the UK BiLEVE consortium, which focused on the extremes of the lung function distribution^55^, highlight the histone subset of the chromatin-packaging and -remodelling pathway. The TGFβ signalling pathway has now been implicated by three independent loci: an FEV_1_/FVC signal explained by a missense variant in *LTBP4*, which encodes a protein that binds TGFβ; an FEV_1_ signal upstream of *TBX3*, which is involved in the TGFβ1 signalling pathway^34^; and a previously reported signal downstream of *TGFB2* (ref. 17). In addition, a pathway involving fibulin-5 has been implicated by two of the novel loci (*LTBP4* and *TRIP11*). The identification, through different approaches, of pathways which appear to be involved in determining lung function should help focus future functional studies.

Pathways affecting lung function also have the potential to affect COPD risk, since lung function measures are used to diagnose the disease. Currently, 13 signals (in or near *TGFB2*, *TNS1*, *RARB*, *FAM13A*, *GSTCD*, *HHIP*, *HTR4*, *ADAM19*, *AGER*, *LOC153910*, *C10orf11*, *RIN3* and *THSD4*) out of the 49 lung function signals discovered to date^10^,^11^,^12^,^13^,^14^ have also shown association with some definition of COPD^38^,^56^,^57^,^58^,^59^,^60^. This illustrates that the study of lung function measures is a powerful approach to bring insights into the genetics of COPD.

In agreement with previous findings for other lung function loci^12^,^13^, none of the 16 new associations seem to be driven by either smoking behaviour or by a gene–smoking interaction. One variant showed association with smoking behaviour that met a Bonferroni correction for 16 tests in UK BiLEVE. This variant also had an effect in never smokers in stage 1, and the allele associated with increased lung function was also associated with increased risk of smoking, which does not suggest an association with lung function mediated by smoking behaviour. Variants in five out of the 16 loci associated with lung function in this study have also shown associations with height^44^,^45^,^46^. However, the variants associated with height were either independent of those associated with lung function, or if they were correlated, the alleles associated with increased height, were associated with decreased FEV_1_ or FVC. If the association with lung function was driven by an effect on height, we would expect consistent direction of effect between these two traits. Therefore, the associations identified for lung function in these regions are not likely to be driven by associations with height.

This study had a large follow-up stage, which included 54,550 individuals, of which 48,943 were contributed by the UK BiLEVE study. UK BiLEVE is a particularly powerful study since it has sampled UK Biobank individuals from the extremes of the lung function distribution, and it has spirometry performed in a uniform way across individuals. Had these data been available when we undertook the discovery stage of this study, their addition would have greatly improved the discovery power. Nevertheless, incorporating these data into the follow-up stage improved power to provide replication and deal with potential winners' curse bias. Another strength of the current study design was the increased coverage of common and low-frequency variants obtained through the imputation to 1000 Genomes Project^18^ reference panel. This enabled us to detect two low-allele-frequency variants (with MAF of 1.5 and 2.4% and stage-1 effect sizes of 0.17 and 0.16 s.d. units, respectively) that have an effect on lung function. No associations with lower allele frequency variants have been detected in this study, despite having power >80% in discovery to detect associations (*P*<5 × 10^−6^) for variants with MAF of 0.5 and 1%, and effect sizes above 0.3 and 0.2 s.d. units, respectively. The poorer imputation quality for low-allele-frequency variants coupled with the strict criteria we used to select variants for follow-up (*N* effective ⩾70%) have probably affected our ability to detect rare variants. For instance, a variant representing an additional signal for FEV_1_/FVC in the *GSTCD*-*INTS12*-*NPNT* region, reported by the UK BiLEVE study, where it was directly genotyped^55^, would have been detected in this analysis had we used a more lenient threshold (*N* effective >60%). Imputation quality for rare variants will improve as larger imputation reference panels become available.

In summary, 16 new association signals for lung function have been identified in this study, including two signals explained by non-synonymous low-frequency variants. These findings highlight new loci not previously connected with lung function or COPD, and bring new insights into previously detected loci. This study also highlights the added value of imputing to new reference panels as they become available. Understanding the molecular pathways that connect the newly identified loci with lung function and COPD risk has the potential to point to new targets for therapeutic intervention.

## Methods

### Study design

The study consisted of two stages. Stage 1 was a meta-analysis of 17 GWAS in a total of 38,199 individuals of European ancestry. Supplementary Table 1 gives the details of these studies. Fifty-six variants selected according to the results in stage 1 were followed up in stage 2 in 54,550 European individuals.

### Stage-1 samples

Stage 1 comprised 17 studies: B58C (T1DGC and WTCCC), BHS1 and -2, EPIC (obese cases and population-based studies), the EUROSPAN studies (CROATIA-Korcula, ORCADES, CROATIA-Split and CROATIA-Vis), GS:SFHS, Health 2000, KORA F4, KORA S3, LBC1936, NFBC1966, NSPHS, SAPALDIA, SHIP and YFS (see Supplementary Table 1a for the definitions of all abbreviations). All participants provided written informed consent and studies were approved by local Research Ethics Committees and/or Institutional Review Boards. Measurements of spirometry for each study are described in the Supplementary Note. The genotyping platforms and quality-control criteria implemented by each study are described in Supplementary Table 1b.

### Imputation

Imputation to the all ancestries 1000 Genomes Project^18^ Phase-1 reference panel released in March 2012 was undertaken using MACH^61^ and minimac^62^ or IMPUTE2 (ref. 63) with pre-imputation filters and parameters as shown in Supplementary Table 1b. Specific software guidelines were used to impute the non-pseudoautosomal part of the X chromosome. The pseudoautosomal part of the X chromosome was not included in these analyses. Variants were excluded if the imputation information, assessed using r2.hat (MACH and minimac) or .info (IMPUTE2), was <0.3.

### Data transformation and association testing in stage 1

Linear regression of age, age^2^, sex, height and principal components for population structure was undertaken on FEV_1_, FEV_1_/FVC and FVC separately for ever smokers and never smokers. The residuals were transformed to ranks and then transformed to normally distributed *z*-scores. These transformed residuals were then used as the phenotype for association testing under an additive genetic model, separately for ever smokers and never smokers. For X chromosome analyses, residuals for males and females were analysed separately and dosages for males were coded 0 for 0 copies of the coded allele and 2 for 1 copy of the coded allele. The software used was specified in Supplementary Table 1b. Studies with related individuals analysed ever smokers and never smokers together adjusting the regression for ever-smoking status and used appropriate tests for association in related individuals, as described in the Supplementary Note.

### Meta-analysis of stage-1 data

Quality-control checks on the stage-1 data were undertaken using GWAtoolbox^64^ and R version 3.0.2 (see URLs). All meta-analysis steps were undertaken using inverse variance-weighted fixed-effects meta-analysis. Effect estimates were flipped across studies so that the coded allele was the reference allele in the 1000 Genomes Project^18^ reference panel. For each study with unrelated individuals, autosomal chromosomes results were meta-analysed between ever smokers and never smokers. After that, all study-specific standard errors were corrected using genomic control^19^. Study-specific genomic inflation factor estimates are shown in Supplementary Table 1a. Finally, effect-size estimates and s.e. were combined across studies, and genomic control^19^ was applied again at the meta-analysis level. For the X chromosome, studies of unrelated individuals meta-analysed smoking strata estimates within sex strata and then meta-analysed pooled sex strata estimates. After that, genomic control^19^ was applied to each study and results were meta-analysed across studies. Genomic control^19^ was applied again after the meta-analysis. To describe the effect of imperfect imputation on power, for each variant we report the effective sample size (*N* effective), which is the sum of the study-specific products of the sample size and the imputation quality metric. Meta-analysis statistics and figures were produced using R version 3.0.2 (see URLs).

### Selection of variants for stage 2

Variants with *N* effective <70% were filtered out before selecting variants for follow-up (8,916,621 variants remained after filtering). Independent regions (±500 kb from the sentinel variant) were selected for FEV_1_, FEV_1_/FVC and FVC if the sentinel SNP or indel had *P*<5 × 10^−6^. If the same variant was selected for different traits, it was followed up for all the traits. If two different variants were selected for different traits within the same region, or if any of the regions selected had already been identified in previous GWAS^10^,^11^,^12^,^13^,^14^ but the sentinel variant was different from that previously reported, conditional analyses were undertaken to assess whether the signals within the same regions were distinct. If previously reported sentinel SNPs for a region were strongly correlated (*r*^2^>0.9), we only conditioned on the SNP that had shown the strongest association. If two variants were selected for different traits within the same new region, both variants were taken forward if their *P*-value conditioning on the other variant was <5 × 10^−6^; if not, only the variant with the most significant *P* value was taken forward. Variants within known regions were only taken forward if their *P* value conditioned on the previously reported variant was <5 × 10^−6^. Conditional analyses were undertaken using GCTA^65^, and B58C data were used to estimate LD. In total, 56 variants (49 SNPs and seven indels) were taken forward for follow-up, two of which were distinct signals within previously reported regions^10^,^12^,^13^. These variants are listed in Supplementary Table 2. Previously reported signals^10^,^11^,^12^,^13^,^14^ were not followed up.

### Stage-2 samples

The 48 SNPs and seven indels on autosomal chromosomes were followed up in up to 54,550 individuals from four studies with *in silico* data: ECRHS, PIVUS, TwinsUK and UK BiLEVE (see Supplementary Table 1a for the definitions of all abbreviations). All participants provided written informed consent and studies were approved by local Research Ethics Committees and/or Institutional Review Boards. One SNP in the chromosome X was followed up in 52,359 individuals from PIVUS, TwinsUK and UK BiLEVE. Measurements of spirometry for each study are described in the Supplementary Note.

### Meta-analysis of stage-2 data

All stage-2 studies undertook linear regression of age, age^2^, sex, height, ever-smoking status and principal components for population structure, if available, on FEV_1_, FEV_1_/FVC and FVC, then the residuals were transformed to ranks and to normally distributed Z-scores. These transformed residuals were then used as the phenotype for association testing under an additive genetic model. For the X chromosome analyses, allele dosages for hemizygous males were coded as 2. Effect sizes were flipped to be consistent with the stage-1 estimates, using the reference allele in 1000 Genomes Project^18^ as the coded allele. Genomic control^19^ was applied for studies that undertook the analysis genome-wide. Effect estimates and s.e. were combined across the stage-2 studies using an inverse variance-weighted meta-analysis.

### Combination of stage 1 and 2 and multiple testing correction

A meta-analysis of stage-1 and stage-2 results was undertaken using inverse variance-weighted meta-analysis. We take into account the multiple tests undertaken by describing an association as genome-wide significant if it has *P*<5 × 10^−8^. In addition, we assessed whether any of the findings achieved independent replication in stage 2 using a threshold corrected for the number of variants followed up (0.05/56=8.93 × 10^−4^).

### Functional characterization of novel loci

A series of analyses were undertaken to provide insights into the expression of genes within the 16 loci (defined as ±1 Mb either side of the sentinel variant) represented here. Blood^21^ and lung tissue^20^ eQTL analyses were undertaken for variants in these loci that were in LD (*r*^2^>0.3) with the sentinel variant in the region. We assessed whether variants within these loci that were strongly correlated with the sentinel variants (*r*^2^>0.8) were in DNase hypersensitivity sites as defined by ENCODE^22^ for cells potentially relevant to lung function. We also carried out conditional analyses, using GCTA^65^, of sentinel variants conditioning on functional variants within the loci to assess whether the association signals were explained by functional variants (*P* value of the sentinel variant conditioned on the functional variant, conditional *P*, >0.01). Functional variants were defined using SIFT^66^, PolyPhen-2 (ref. 67), CADD^68^ and GWAVA^69^ databases. Additional analyses were undertaken for a subset of priority genes within the 16 loci (description of the selection is given in the Supplementary Methods). These included RNA-seq analyses to confirm messenger RNA expression in a lung-relevant cell (bronchial epithelium) and detect novel splice isoforms; assessment of differential expression across pseudoglandular and canalicular stages of human fetal lung development using gestational age as a continuous variable in linear regression^25^, and assessment of differences in expression levels in bronchial brushings between COPD cases and smoking controls^70^. Details for all these analyses are provided in the Supplementary Methods.

### Associations with other traits

The association of the 16 sentinel variants with the following traits was assessed: lung function in children undertaking the same analysis as for adults in the ALSPAC data set^42^; gene by smoking interaction by undertaking a Z-test comparing the effect of a given variant in ever smokers and in never smokers using stage-1 results; smoking behaviour by undertaking a logistic regression analysis with heavy- versus never-smoking status as an outcome in the UK BiLEVE data set. In addition, the GWAS catalog^43^ was queried for variants in 2-Mb regions centred on the sentinel variant for the 16 loci. Variants that were genome-wide significant (*P*<5 × 10^−8^) in the GWAS catalog^43^ and were in LD (*r*^2^>0.3) with the sentinel variants, or were in genes that contained at least one variant in LD (*r*^2^>0.3) with the sentinel variants were selected.

### Pathway analyses

Stage-1 GWAS results were tested for enrichment of known biological pathways using MAGENTA v2 (ref. 52). Six databases of biological pathways, including Ingenuity Pathway (June 2008, number of pathways *n*=81), KEGG (2010, *n*=186), PANTHER Molecular Function (January 2010, *n*=216), PANTHER Biological Processes (January 2010, *n*=217), PANTHER Pathways (January 2010, *n*=94) and Gene Ontology (April 2010, *n*=1778), were tested. An FDR threshold of 5% was used and significance thresholds were Bonferroni corrected for each database. Genes within 500 kb either side from the sentinel variants were flagged in the analysis. Sensitivity analyses were run after removing genes in the human leukocyte antigen region on chromosome 6. More details on the method are provided in the Supplementary Methods.

### Additional analyses

Heterogeneity tests were undertaken for the 16 sentinel variants in stage 1. We undertook stepwise conditional analyses as performed by GCTA^65^ in each locus to identify additional signals. Full methods and results are described in the Supplementary Notes.

## Additional information

**How to cite this article:** Artigas, M. S. *et al*. Sixteen new lung function signals identified through 1000 Genomes Project reference panel imputation. *Nat. Commun.* 6:8658 doi: 10.1038/ncomms9658 (2015).

## Supplementary Material

Supplementary Information Supplementary Figures 1-5, Supplementary Tables 1-9, Supplementary Notes 1-4, Supplementary Methods and Supplementary References

## Figures and Tables

**Table 1:**
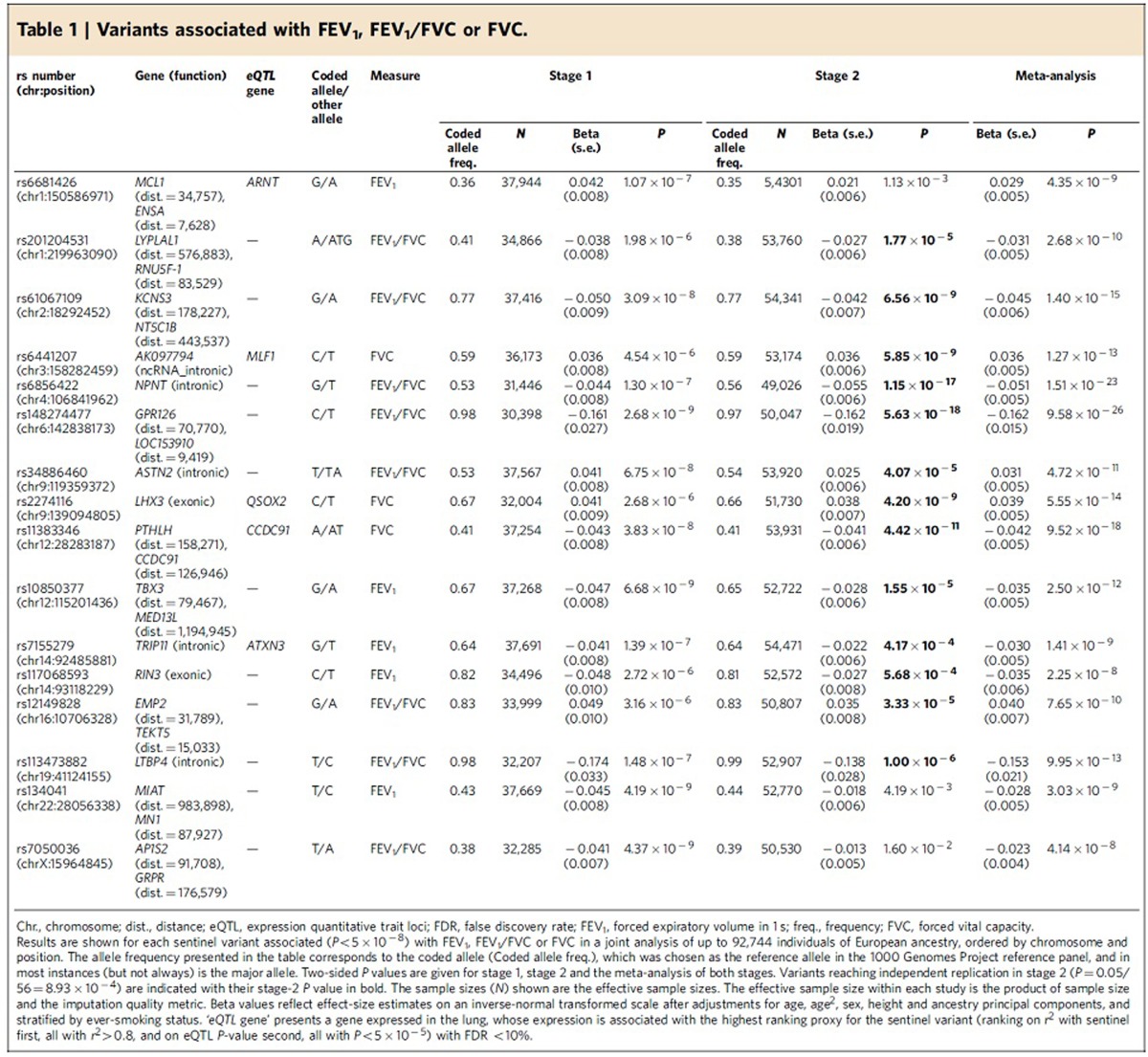
Variants associated with FEV_1_, FEV_1_/FVC or FVC.

## References

[b1] Hole D. J. . Impaired lung function and mortality risk in men and women: findings from the Renfrew and Paisley prospective population study. BMJ 313, 711–715 discussion 715–716 (1996). 8819439 10.1136/bmj.313.7059.711 PMC2352103

[b2] Young R. P., Hopkins R. & Eaton T. E. Forced expiratory volume in one second: not just a lung function test but a marker of premature death from all causes. Eur. Respir. J. 30, 616–622 (2007). 17906084 10.1183/09031936.00021707

[b3] Lopez A. D. . Chronic obstructive pulmonary disease: current burden and future projections. Eur. Respir. J. 27, 397–412 (2006). 16452599 10.1183/09031936.06.00025805

[b4] Lozano R. . Global and regional mortality from 235 causes of death for 20 age groups in 1990 and 2010: a systematic analysis for the Global Burden of Disease Study 2010. Lancet 380, 2095–2128 (2012). 23245604 10.1016/S0140-6736(12)61728-0 PMC10790329

[b5] Zappala C. J. . Marginal decline in forced vital capacity is associated with a poor outcome in idiopathic pulmonary fibrosis. Eur. Respir. J. 35, 830–836 (2010). 19840957 10.1183/09031936.00155108

[b6] Abbey D. E. . Long-term particulate and other air pollutants and lung function in nonsmokers. Am. J. Respir. Crit. Care Med. 158, 289–298 (1998). 9655742 10.1164/ajrccm.158.1.9710101

[b7] Global Initiative for Chronic Obstructive Lung Disease. Global Strategy for the Diagnosis Management and Prevention of COPD. http://www.goldcopd.org/ (2014).

[b8] Wilk J. B. . Evidence for major genes influencing pulmonary function in the NHLBI family heart study. Genet. Epidemiol. 19, 81–94 (2000). 10861898 10.1002/1098-2272(200007)19:1<81::AID-GEPI6>3.0.CO;2-8

[b9] Palmer L. J. . Familial aggregation and heritability of adult lung function: results from the Busselton Health Study. Eur. Respir. J. 17, 696–702 (2001). 11401066 10.1183/09031936.01.17406960

[b10] Hancock D. B. . Meta-analyses of genome-wide association studies identify multiple loci associated with pulmonary function. Nat. Genet. 42, 45–52 (2010). 20010835 10.1038/ng.500 PMC2832852

[b11] Loth D. W. . Genome-wide association analysis identifies six new loci associated with forced vital capacity. Nat. Genet. 46, 669–677 (2014). 24929828 10.1038/ng.3011 PMC4140093

[b12] Repapi E. . Genome-wide association study identifies five loci associated with lung function. Nat. Genet. 42, 36–44 (2010). 20010834 10.1038/ng.501 PMC2862965

[b13] Soler Artigas M. . Genome-wide association and large-scale follow up identifies 16 new loci influencing lung function. Nat. Genet. 43, 1082–1090 (2011). 21946350 10.1038/ng.941 PMC3267376

[b14] Wilk J. B. . A genome-wide association study of pulmonary function measures in the Framingham Heart Study. PLoS Genet. 5, e1000429 (2009). 19300500 10.1371/journal.pgen.1000429 PMC2652834

[b15] Maher B. Personal genomes: the case of the missing heritability. Nature 456, 18–21 (2008). 18987709 10.1038/456018a

[b16] Manolio T. A. . Finding the missing heritability of complex diseases. Nature 461, 747–753 (2009). 19812666 10.1038/nature08494 PMC2831613

[b17] Gibson G. Rare and common variants: twenty arguments. Nat. Rev. Genet. 13, 135–145 (2011). 22251874 10.1038/nrg3118 PMC4408201

[b18] 1000 Genomes Project Consortium. An integrated map of genetic variation from 1,092 human genomes. Nature 491, 56–65 (2012). 23128226 10.1038/nature11632 PMC3498066

[b19] Devlin B. & Roeder K. Genomic control for association studies. Biometrics 55, 997–1004 (1999). 11315092 10.1111/j.0006-341x.1999.00997.x

[b20] Lamontagne M. . Refining susceptibility loci of chronic obstructive pulmonary disease with lung eqtls. PLoS ONE 8, e70220 (2013). 23936167 10.1371/journal.pone.0070220 PMC3728203

[b21] Westra H. J. . Systematic identification of trans eQTLs as putative drivers of known disease associations. Nat. Genet. 45, 1238–1243 (2013). 24013639 10.1038/ng.2756 PMC3991562

[b22] Rosenbloom K. R. . ENCODE data in the UCSC Genome Browser: year 5 update. Nucleic Acids Res. 41, D56–D63 (2013). 23193274 10.1093/nar/gks1172 PMC3531152

[b23] Uhlen M. . Towards a knowledge-based Human Protein Atlas. Nat. Biotechnol. 28, 1248–1250 (2010). 21139605 10.1038/nbt1210-1248

[b24] GTEx Consortium. The Genotype-Tissue Expression (GTEx) project. Nat. Genet. 45, 580–585 (2013). 23715323 10.1038/ng.2653 PMC4010069

[b25] Melen E. . Expression analysis of asthma candidate genes during human and murine lung development. Respir. Res. 12, 86 (2011). 21699702 10.1186/1465-9921-12-86 PMC3141421

[b26] Waller-Evans H. . The orphan adhesion-GPCR GPR126 is required for embryonic development in the mouse. PLoS ONE 5, e14047 (2010). 21124978 10.1371/journal.pone.0014047 PMC2987804

[b27] Moriguchi T. . DREG, a developmentally regulated G protein-coupled receptor containing two conserved proteolytic cleavage sites. Genes Cells 9, 549–560 (2004). 15189448 10.1111/j.1356-9597.2004.00743.x

[b28] Paavola K. J., Sidik H., Zuchero J. B., Eckart M. & Talbot W. S. Type IV collagen is an activating ligand for the adhesion G protein-coupled receptor GPR126. Sci. Signal. 7, ra76 (2014). 25118328 10.1126/scisignal.2005347 PMC4159047

[b29] Obeidat M. e . GSTCD and INTS12 regulation and expression in the human lung. PLoS ONE 8, e74630 (2013). 24058608 10.1371/journal.pone.0074630 PMC3776747

[b30] Dabovic B. . Function of latent TGFbeta binding protein 4 and fibulin 5 in elastogenesis and lung development. J. Cell. Physiol. 230, 226–236 (2015). 24962333 10.1002/jcp.24704 PMC4436707

[b31] Sterner-Kock A. . Disruption of the gene encoding the latent transforming growth factor-beta binding protein 4 (LTBP-4) causes abnormal lung development, cardiomyopathy, and colorectal cancer. Genes Dev. 16, 2264–2273 (2002). 12208849 10.1101/gad.229102 PMC186672

[b32] Cho M. H. . A genome-wide association study of COPD identifies a susceptibility locus on chromosome 19q13. Hum. Mol. Genet. 21, 947–957 (2012). 22080838 10.1093/hmg/ddr524 PMC3298111

[b33] Thorgeirsson T. E. . Sequence variants at CHRNB3-CHRNA6 and CYP2A6 affect smoking behavior. Nat. Genet. 42, 448–453 (2010). 20418888 10.1038/ng.573 PMC3080600

[b34] Li J. . The anti-proliferative function of the TGF-beta1 signalling pathway involves the repression of the oncogenic TBX2 by its homologue TBX3. J. Biol. Chem. 289, 35633–35643 (2014). 25371204 10.1074/jbc.M114.596411 PMC4271245

[b35] Follit J. A. . The Golgin GMAP210/TRIP11 anchors IFT20 to the Golgi complex. PLoS Genet. 4, e1000315 (2008). 19112494 10.1371/journal.pgen.1000315 PMC2602600

[b36] Kawaguchi Y. . CAG expansions in a novel gene for Machado-Joseph disease at chromosome 14q32.1. Nat. Genet. 8, 221–228 (1994). 7874163 10.1038/ng1194-221

[b37] Brandsma C. A. . A large lung gene expression study identifying fibulin-5 as a novel player in tissue repair in COPD. Thorax 70, 21–32 (2015). 24990664 10.1136/thoraxjnl-2014-205091

[b38] Cho M. H. . Risk loci for chronic obstructive pulmonary disease: a genome-wide association study and meta-analysis. Lancet. Respir. Med. 2, 214–225 (2014). 24621683 10.1016/S2213-2600(14)70002-5 PMC4176924

[b39] Groenman F., Rutter M., Caniggia I., Tibboel D. & Post M. Hypoxia-inducible factors in the first trimester human lung. J. Histochem. Cytochem. 55, 355–363 (2007). 17189520 10.1369/jhc.6A7129.2006

[b40] Ovrevik J. . AhR and Arnt differentially regulate NF-kappaB signaling and chemokine responses in human bronchial epithelial cells. Cell Commun. Signal. 12, 48 (2014). 25201625 10.1186/s12964-014-0048-8 PMC4222560

[b41] Petrache I. . Ceramide synthases expression and role of ceramide synthase-2 in the lung: insight from human lung cells and mouse models. PLoS ONE 8, e62968 (2013). 23690971 10.1371/journal.pone.0062968 PMC3653891

[b42] Boyd A. . Cohort Profile: the ‘children of the 90s'—the index offspring of the Avon Longitudinal Study of Parents and Children. Int. J. Epidemiol. 42, 111–127 (2013). 22507743 10.1093/ije/dys064 PMC3600618

[b43] Welter D. . The NHGRI GWAS Catalog, a curated resource of SNP-trait associations. Nucleic Acids Research 42, D1001–D1006 (2014). 24316577 10.1093/nar/gkt1229 PMC3965119

[b44] Lango Allen H. . Hundreds of variants clustered in genomic loci and biological pathways affect human height. Nature 467, 832–838 (2010). 20881960 10.1038/nature09410 PMC2955183

[b45] Lettre G. . Identification of ten loci associated with height highlights new biological pathways in human growth. Nat. Genet. 40, 584–591 (2008). 18391950 10.1038/ng.125 PMC2687076

[b46] Berndt S. I. . Genome-wide meta-analysis identifies 11 new loci for anthropometric traits and provides insights into genetic architecture. Nat. Genet. 45, 501–512 (2013). 23563607 10.1038/ng.2606 PMC3973018

[b47] Albagha O. M. . Genome-wide association identifies three new susceptibility loci for Paget's disease of bone. Nat. Genet. 43, 685–689 (2011). 21623375 10.1038/ng.845

[b48] Kemp J. P. . Phenotypic dissection of bone mineral density reveals skeletal site specificity and facilitates the identification of novel loci in the genetic regulation of bone mass attainment. PLoS Genet. 10, e1004423 (2014). 24945404 10.1371/journal.pgen.1004423 PMC4063697

[b49] Pei Y. F. . Meta-analysis of genome-wide association data identifies novel susceptibility loci for obesity. Hum. Mol. Genet. 23, 820–830 (2014). 24064335 10.1093/hmg/ddt464 PMC3888264

[b50] Macgregor S. . Genome-wide association study identifies a new melanoma susceptibility locus at 1q21.3. Nat. Genet. 43, 1114–1118 (2011). 21983785 10.1038/ng.958 PMC3227560

[b51] Porcu E. . A meta-analysis of thyroid-related traits reveals novel loci and gender-specific differences in the regulation of thyroid function. PLoS Genet. 9, e1003266 (2013). 23408906 10.1371/journal.pgen.1003266 PMC3567175

[b52] Segrè A. V. . Common inherited variation in mitochondrial genes is not enriched for associations with type 2 diabetes or related glycemic traits. PLoS Genet. 6, e1001058 (2010). 20714348 10.1371/journal.pgen.1001058 PMC2920848

[b53] Wood A. R. . Defining the role of common variation in the genomic and biological architecture of adult human height. Nat. Genet. 46, 1173–1186 (2014). 25282103 10.1038/ng.3097 PMC4250049

[b54] Tada H. . Multiple associated variants increase the heritability explained for plasma lipids and coronary artery disease. circulation. Cardiovasc. Genet. 7, 583–587 (2014). 10.1161/CIRCGENETICS.113.000420 PMC4341828 25170055

[b55] Wain L. V. . Novel insights into the genetics of smoking behaviour, lung function, and chronic obstructive pulmonary disease (UK BiLEVE): a genetic association study in UK Biobank. Lancet Respir. Med. doi: 10.1016/S2213-2600(15)00283-0 (2015). 10.1016/S2213-2600(15)00283-0 PMC4593935 26423011

[b56] Soler Artigas M. . Effect of five genetic variants associated with lung function on the risk of chronic obstructive lung disease, and their joint effects on lung function. Am. J. Respir. Crit. Care Med. 184, 786–795 (2011). 21965014 10.1164/rccm.201102-0192OC PMC3398416

[b57] Castaldi P. J. . The association of genome-wide significant spirometric loci with chronic obstructive pulmonary disease susceptibility. Am. J. Respir. Cell Mol. Biol. 45, 1147–1153 (2011). 21659657 10.1165/rcmb.2011-0055OC PMC3262664

[b58] Pillai S. G. . A genome-wide association study in chronic obstructive pulmonary disease (COPD): identification of two major susceptibility loci. PLoS Genet. 5, e1000421 (2009). 19300482 10.1371/journal.pgen.1000421 PMC2650282

[b59] Cho M. H. . Variants in FAM13A are associated with chronic obstructive pulmonary disease. Nat. Genet. 42, 200–202 (2010). 20173748 10.1038/ng.535 PMC2828499

[b60] Wilk J. B. . Genome-wide association studies identify CHRNA5/3 and HTR4 in the development of airflow obstruction. Am. J. Respir. Crit. Care Med. 186, 622–632 (2012). 22837378 10.1164/rccm.201202-0366OC PMC3480517

[b61] Li Y. & Abecasis G. R. Mach 1.0: rapid haplotype construction and missing genotype inference. Am. J. Hum. Genet. S79, 2290 (2006).

[b62] Howie B., Fuchsberger C., Stephens M., Marchini J. & Abecasis G. R. Fast and accurate genotype imputation in genome-wide association studies through pre-phasing. Nat. Genet. 44, 955–959 (2012). 22820512 10.1038/ng.2354 PMC3696580

[b63] Howie B. N., Donnelly P. & Marchini J. A flexible and accurate genotype imputation method for the next generation of genome-wide association studies. PLoS Genet. 5, e1000529 (2009). 19543373 10.1371/journal.pgen.1000529 PMC2689936

[b64] Fuchsberger C., Taliun D., Pramstaller P. P., Pattaro C. & consortium C. K GWAtoolbox: an R package for fast quality control and handling of genome-wide association studies meta-analysis data. Bioinformatics 28, 444–445 (2012). 22155946 10.1093/bioinformatics/btr679

[b65] Yang J. . Conditional and joint multiple-SNP analysis of GWAS summary statistics identifies additional variants influencing complex traits. Nat. Genet. 44, S361–S363 (2012). 10.1038/ng.2213 PMC3593158 22426310

[b66] Kumar P., Henikoff S. & Ng P. C. Predicting the effects of coding non-synonymous variants on protein function using the SIFT algorithm. Nat. Protoc. 4, 1073–1081 (2009). 19561590 10.1038/nprot.2009.86

[b67] Adzhubei I. A. . A method and server for predicting damaging missense mutations. Nat. Methods 7, 248–249 (2010). 20354512 10.1038/nmeth0410-248 PMC2855889

[b68] Kircher M. . A general framework for estimating the relative pathogenicity of human genetic variants. Nat. Genet. 46, 310–315 (2014). 24487276 10.1038/ng.2892 PMC3992975

[b69] Ritchie G. R., Dunham I., Zeggini E. & Flicek P. Functional annotation of noncoding sequence variants. Nat. Methods 11, 294–296 (2014). 24487584 10.1038/nmeth.2832 PMC5015703

[b70] Steiling K. . A dynamic bronchial airway gene expression signature of chronic obstructive pulmonary disease and lung function impairment. Am. . Respir. Crit. Care Med. 187, 933–942 (2013). 10.1164/rccm.201208-1449OC PMC3707363 23471465

