# Supplementary material for: Sixteen new lung function signals identified through 1000 Genomes Project reference panel imputation
Source: Nat Commun. 2015 Dec 4;6:8658. doi: 10.1038/ncomms9658 (PMC4686825; doi:10.1038/ncomms9658)
Supplement: Supplementary Information — Supplementary Figures 1-5, Supplementary Tables 1-9, Supplementary Notes 1-4, Supplementary Methods and Supplementary References [file ncomms9658-s1.pdf]

<u>Supplementary Figures:</u>

Supplementary Figure 1 Study design for X chromosome analyses

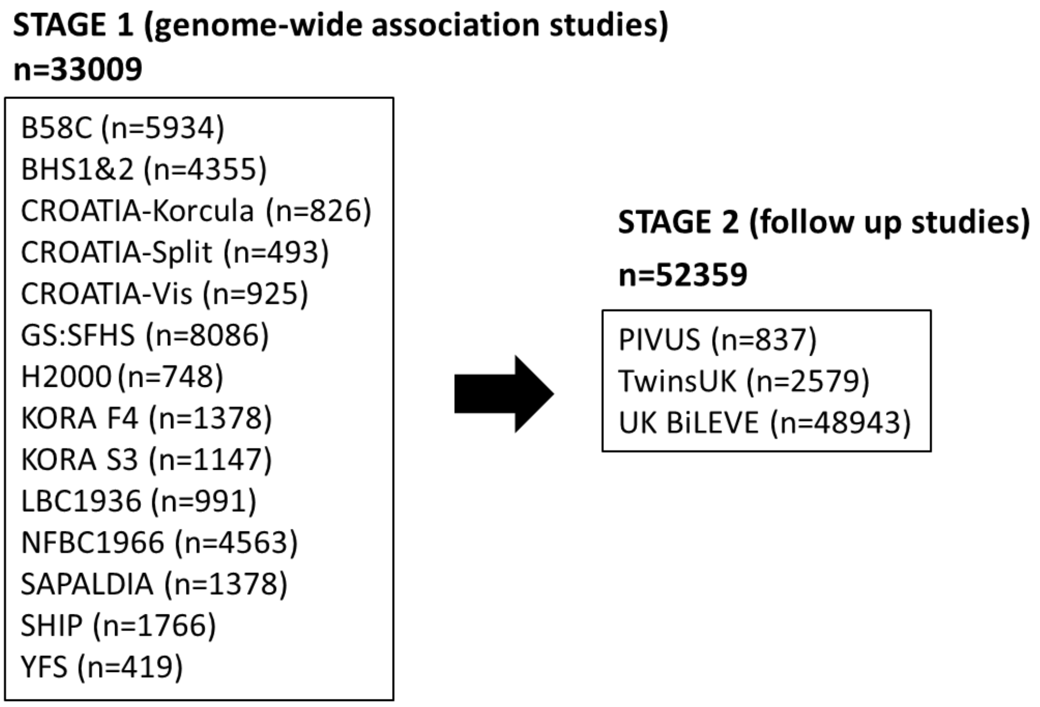

The discovery stage (stage 1) included 14 studies and 33,009 individuals. One variant was followed up in stage 2 in 3 studies and 52,359 individuals.

Supplementary Figure 2 Association test statistics for FEV_1, FEV_1/FVC and FVC

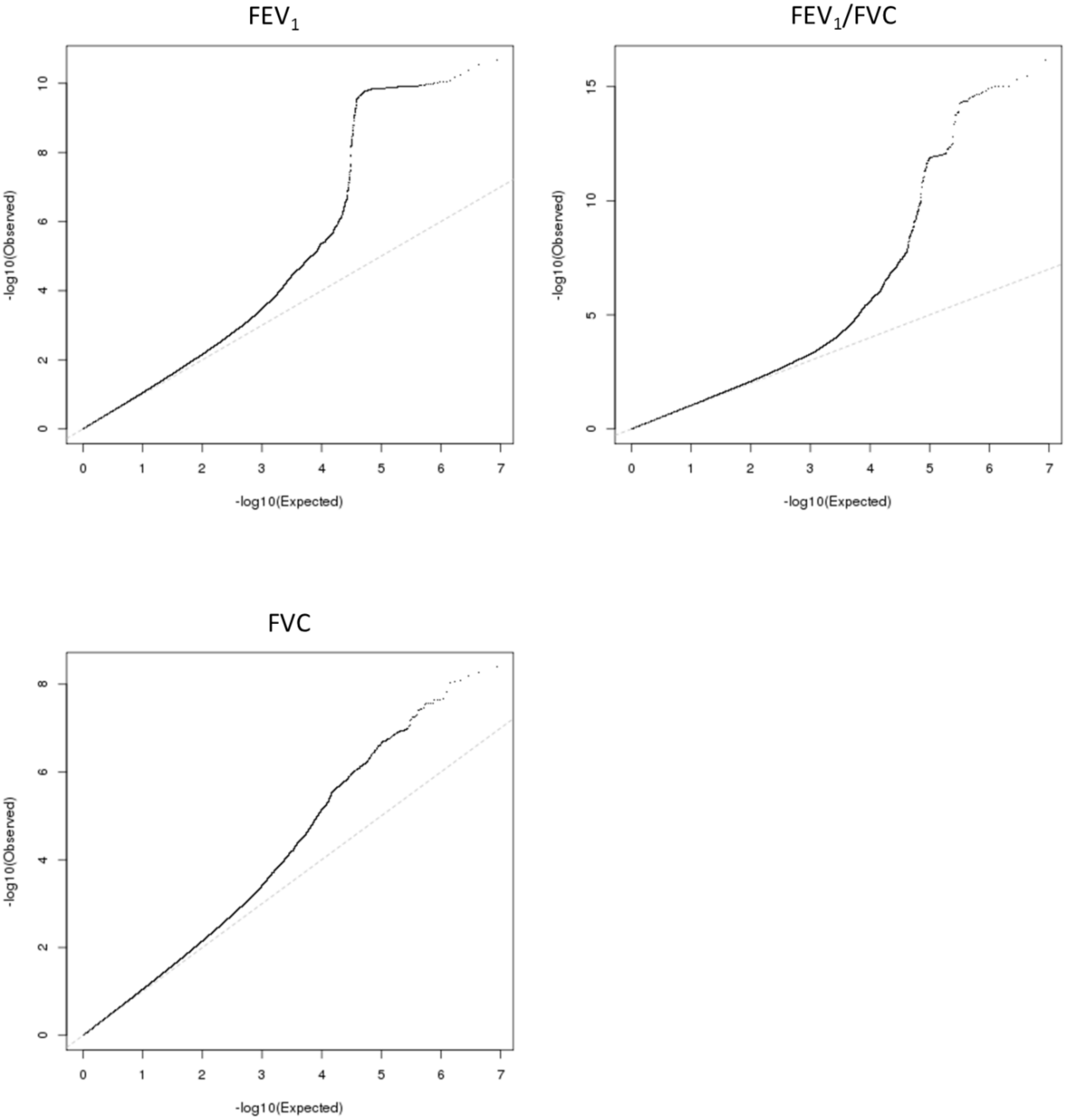

a) Quantile-quantile plots. QQplots show $-\log_{10}(P)$ of observed genome-wide association results against expected results in case of no association for autosomal chromosomes for FEV_1, FEV_1/FVC and FVC. Only variants with effective sample size (product of sample size and imputation quality summed up across studies) ≥70% are included.

FEV$_1$

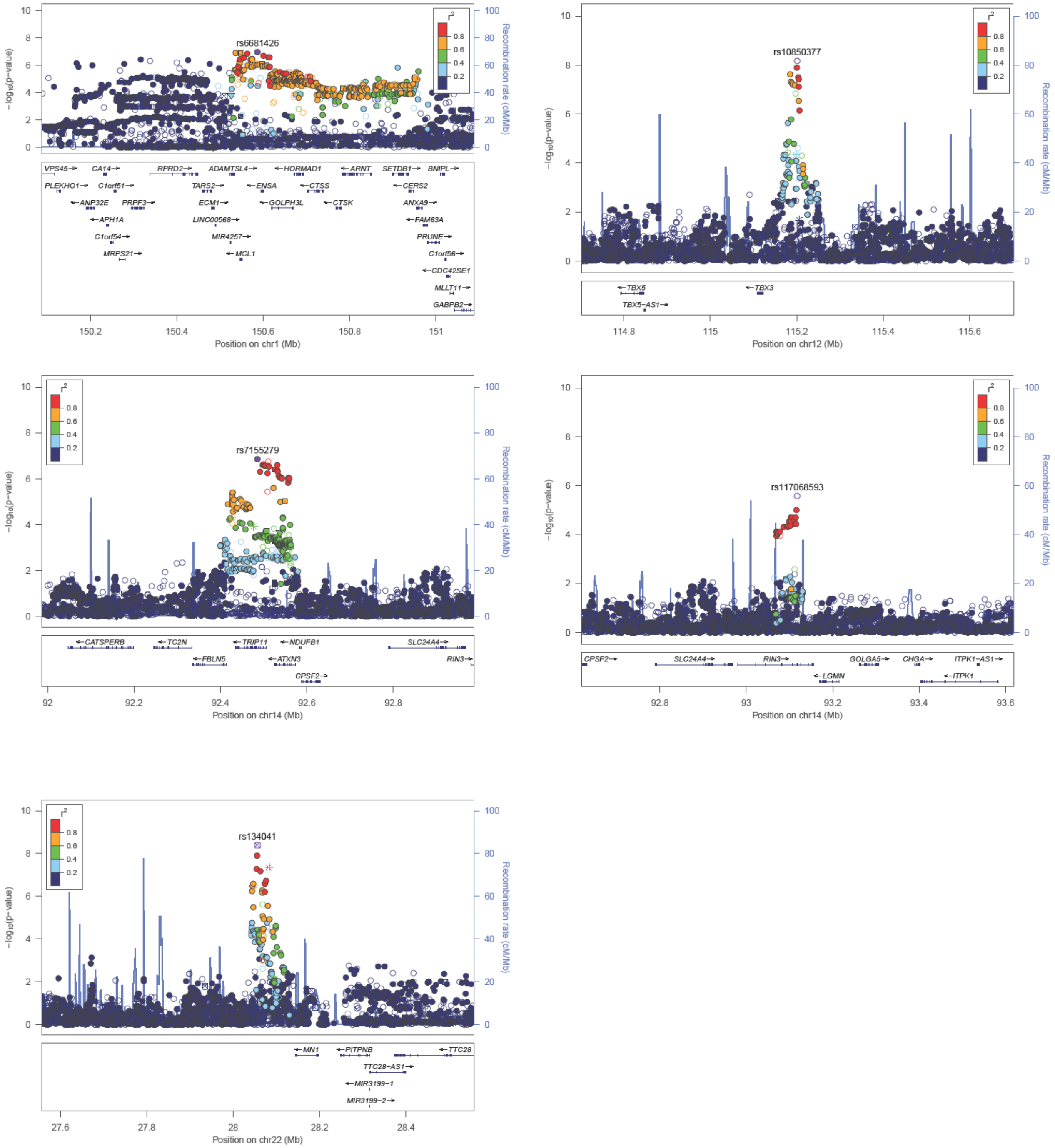
rs6681426
Position on chr1 (Mb)
VPS45 CA14 RPRD2 ADAMTSL4 HORMAD1 ARNT SETDB1 BNIPL
PLEKHO1 C1orf51 TARS2 ENSA CTSS CERS2
ANP32E PRPF3 ECM1 GOLPH3L CTSK ANXA9
APH1A LINC00568 FAM63A
C1orf54 MIR4257 PRUNE
MRPS21 MCL1 C1orf56
CDC42SE1
MLLT11
GABPB2
rs10850377
Position on chr12 (Mb)
TBX5 TBX3
TBX5−AS1
rs7155279
Position on chr14 (Mb)
CATSPERB TC2N TRIP11 NDUFB1 SLC24A4
FBLN5 ATXN3 RIN3
CPSF2
rs117068593
Position on chr14 (Mb)
CPSF2 SLC24A4 RIN3 GOLGA5 CHGA ITPK1−AS1
LGMN ITPK1
rs134041
Position on chr22 (Mb)
MN1 PITPNB TTC28
TTC28−AS1
MIR3199−1
MIR3199−2
−log10(p−value)
Recombination rate (cM/Mb)
r2

FEV_1/FVC

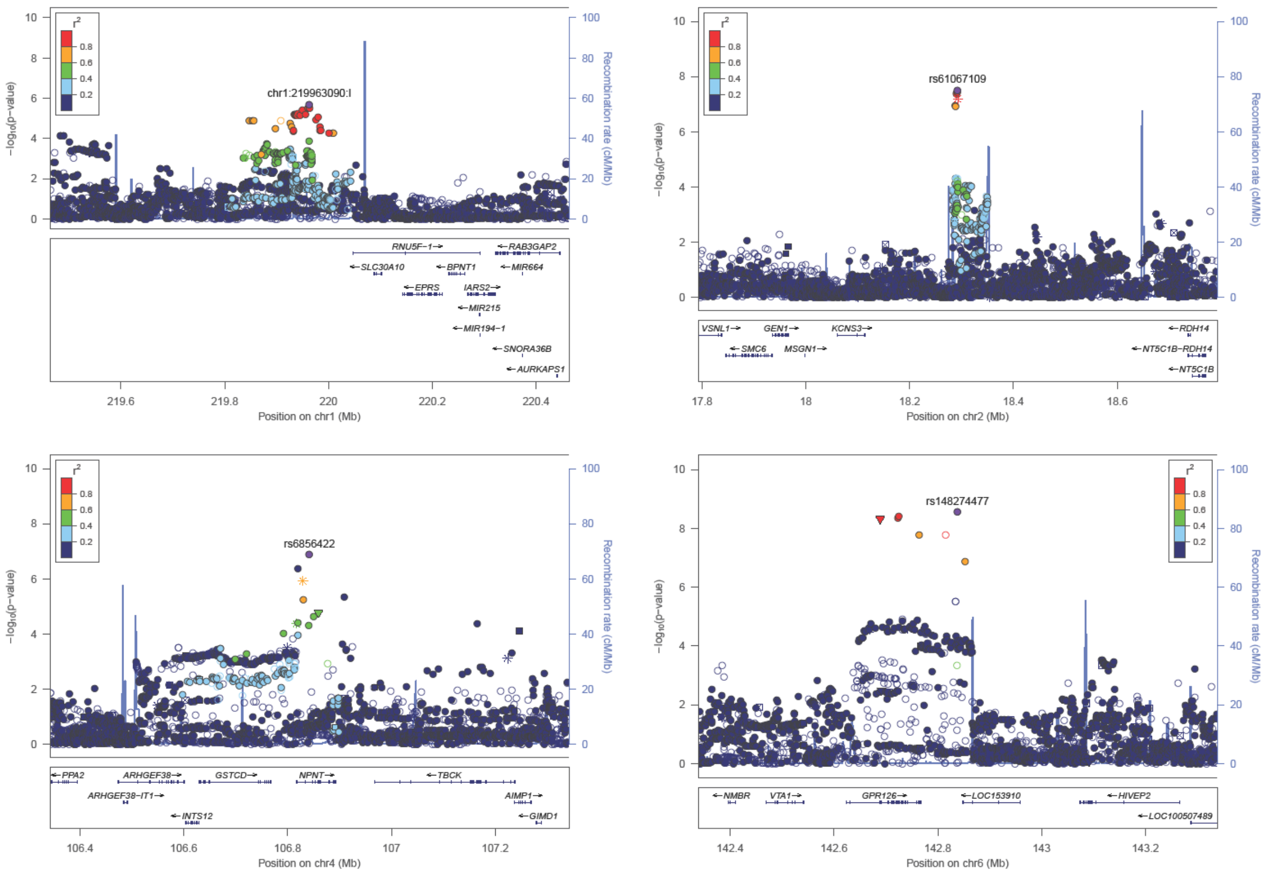
chr1:219963090:I
rs61067109
rs6856422
rs148274477
r2
−log10(p−value)
Recombination rate (cM/Mb)
Position on chr1 (Mb)
Position on chr2 (Mb)
Position on chr4 (Mb)
Position on chr6 (Mb)
RNU5F−1
RAB3GAP2
SLC30A10
BPNT1
MIR664
EPRS
IARS2
MIR215
MIR194−1
SNORA36B
AURKAPS1
VSNL1
GEN1
KCNS3
RDH14
SMC6
MSGN1
NT5C1B−RDH14
NT5C1B
PPA2
ARHGEF38
GSTCD
NPNT
TBCK
ARHGEF38−IT1
AIMP1
INTS12
GIMD1
NMBR
VTA1
GPR126
LOC153910
HIVEP2
LOC100507489

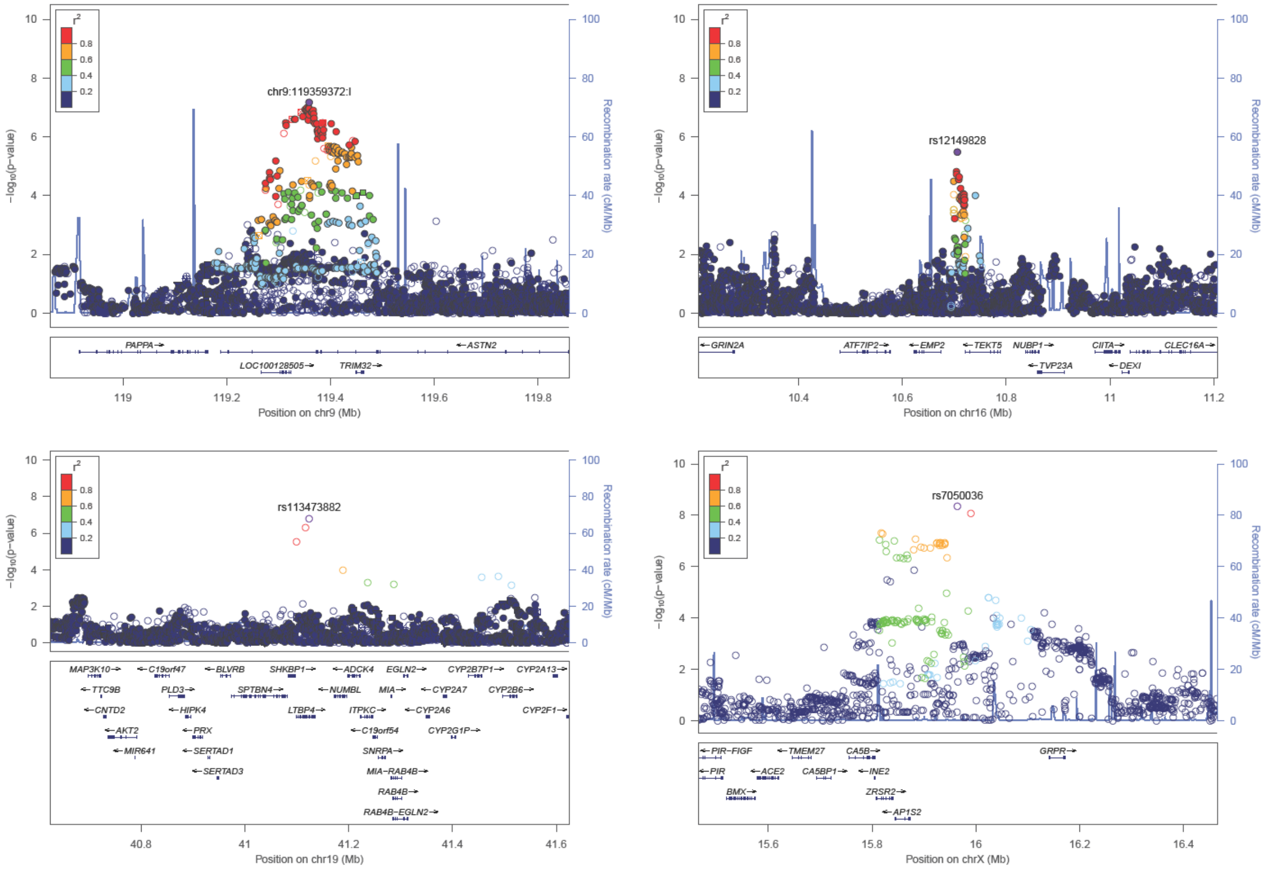
chr9:119359372:I
rs12149828
rs113473882
rs7050036
r2
0.8
0.6
0.4
0.2
−log10(p−value)
Recombination rate (cM/Mb)
Position on chr9 (Mb)
Position on chr16 (Mb)
Position on chr19 (Mb)
Position on chrX (Mb)
PAPPA
ASTN2
LOC100128505
TRIM32
GRIN2A
ATF7IP2
EMP2
TEKT5
NUBP1
CIITA
CLEC16A
TVP23A
DEXI
MAP3K10
C19orf47
BLVRB
SHKBP1
ADCK4
EGLN2
CYP2B7P1
CYP2A13
TTC9B
PLD3
SPTBN4
NUMBL
MIA
CYP2A7
CYP2B6
CNTD2
HIPK4
LTBP4
ITPKC
CYP2A6
CYP2F1
AKT2
PRX
C19orf54
CYP2G1P
MIR641
SERTAD1
SNRPA
SERTAD3
MIA−RAB4B
RAB4B
RAB4B−EGLN2
PIR−FIGF
TMEM27
CA5B
GRPR
PIR
ACE2
CA5BP1
INE2
BMX
ZRSR2
AP1S2

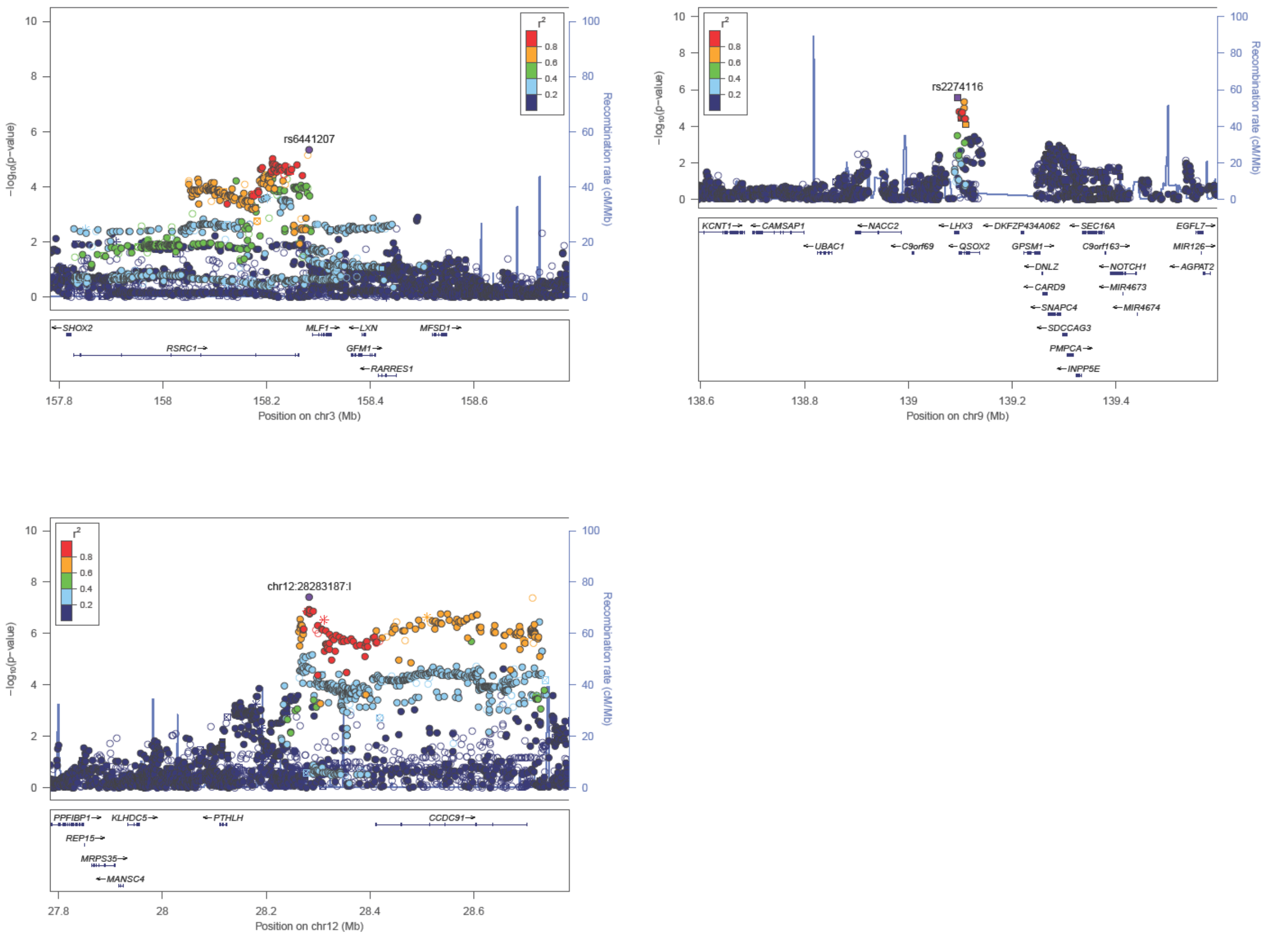

b) Regional association plots for 16 new lung function signals. –log10(P) in stage 1 meta-analysis for the trait with strongest association are plotted against chromosomal position (NCBI 37) for 1Mb regions. Sentinel variants in each plot and represented in purple. Other variants are coloured according to their correlation with the sentinel variant (see legend in each plot). Annotation for each variant is presented as default in locuszoom (http://csg.sph.umich.edu/locuszoom/): framestop and splice, triangle; non synonymous, inverted triangle; synonymous and UTR, square; TFBScons , star; MCS44 Placental, square with diagonal lines and None-of-the-above, filled circle.

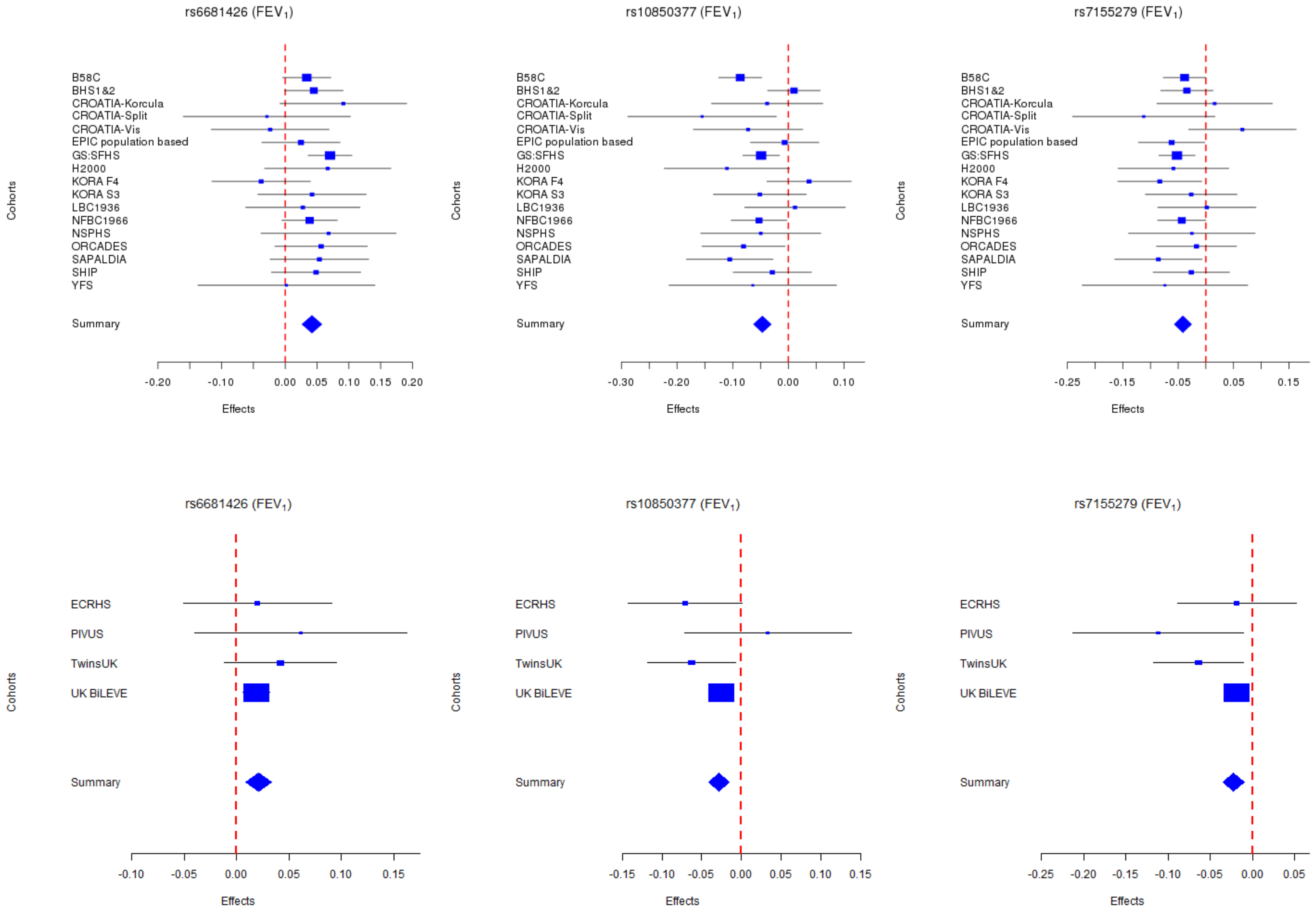
rs6681426 (FEV$_1$)
rs10850377 (FEV$_1$)
rs7155279 (FEV$_1$)
B58C
BHS1&2
CROATIA-Korcula
CROATIA-Split
CROATIA-Vis
EPIC population based
GS:SFHS
H2000
KORA F4
KORA S3
LBC1936
NFBC1966
NSPHS
ORCADES
SAPALDIA
SHIP
YFS
Summary
Cohorts
Effects
-0.20 -0.10 0.00 0.05 0.10 0.15 0.20
-0.30 -0.20 -0.10 0.00 0.10
-0.25 -0.15 -0.05 0.05 0.15
rs6681426 (FEV$_1$)
rs10850377 (FEV$_1$)
rs7155279 (FEV$_1$)
ECRHS
PIVUS
TwinsUK
UK BiLEVE
Summary
Cohorts
Effects
-0.10 -0.05 0.00 0.05 0.10 0.15
-0.15 -0.10 -0.05 0.00 0.05 0.10 0.15
-0.25 -0.20 -0.15 -0.10 -0.05 0.00 0.05

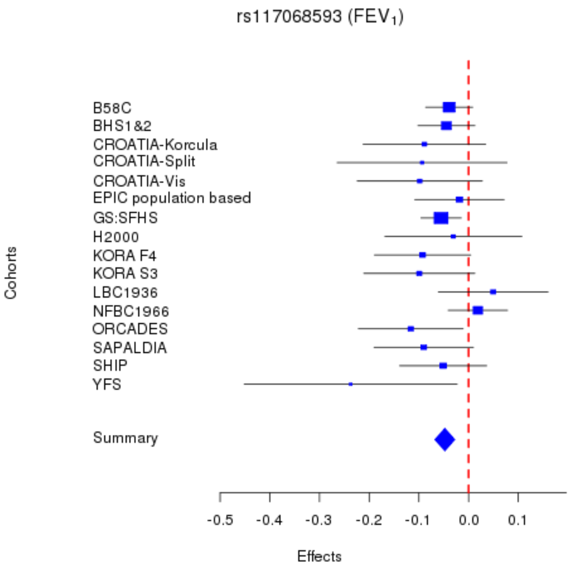
rs117068593 (FEV1)
B58C
BHS1&2
CROATIA-Korcula
CROATIA-Split
CROATIA-Vis
EPIC population based
GS:SFHS
H2000
KORA F4
KORA S3
LBC1936
NFBC1966
ORCADES
SAPALDIA
SHIP
YFS
Summary
Cohorts
-0.5 -0.4 -0.3 -0.2 -0.1 0.0 0.1
Effects

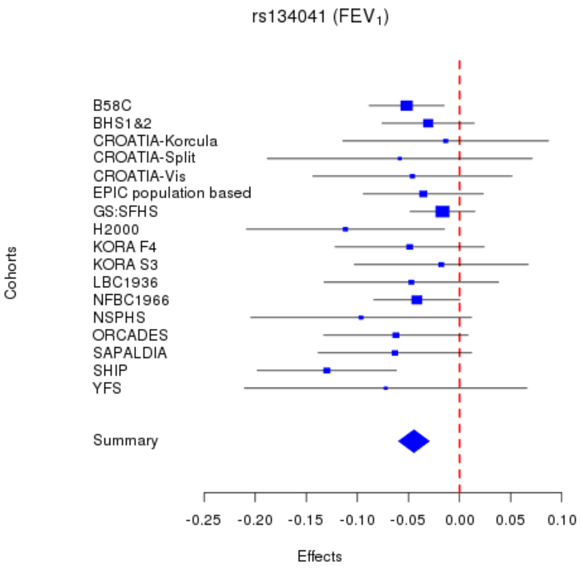
rs134041 (FEV1)
B58C
BHS1&2
CROATIA-Korcula
CROATIA-Split
CROATIA-Vis
EPIC population based
GS:SFHS
H2000
KORA F4
KORA S3
LBC1936
NFBC1966
NSPHS
ORCADES
SAPALDIA
SHIP
YFS
Summary
Cohorts
-0.25 -0.20 -0.15 -0.10 -0.05 0.00 0.05 0.10
Effects

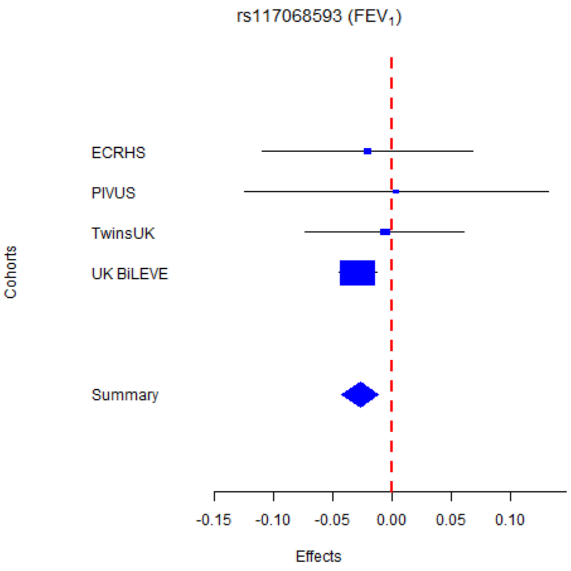
rs117068593 (FEV1)
ECRHS
PIVUS
TwinsUK
UK BiLEVE
Summary
Cohorts
-0.15 -0.10 -0.05 0.00 0.05 0.10
Effects

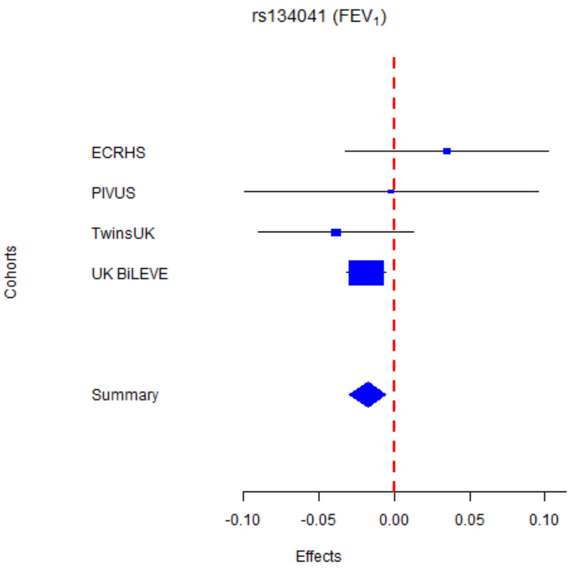
rs134041 (FEV1)
ECRHS
PIVUS
TwinsUK
UK BiLEVE
Summary
Cohorts
-0.10 -0.05 0.00 0.05 0.10
Effects

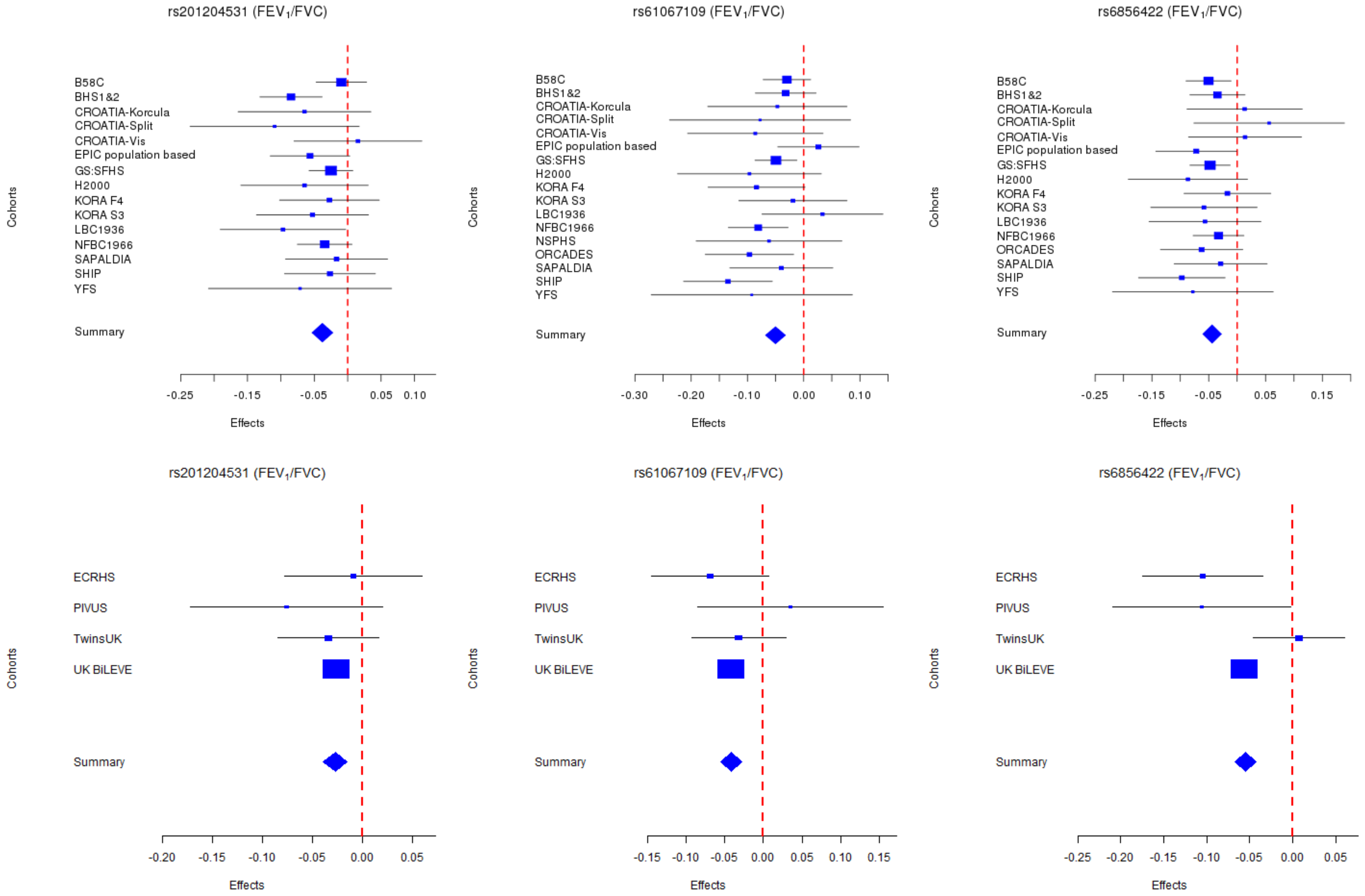
rs201204531 (FEV1/FVC)
rs61067109 (FEV1/FVC)
rs6856422 (FEV1/FVC)
Cohorts
B58C
BHS1&2
CROATIA-Korcula
CROATIA-Split
CROATIA-Vis
EPIC population based
GS:SFHS
H2000
KORA F4
KORA S3
LBC1936
NFBC1966
NSPHS
ORCADES
SAPALDIA
SHIP
YFS
Summary
Effects
-0.30 -0.25 -0.20 -0.15 -0.10 -0.05 0.00 0.05 0.10 0.15
ECRHS
PIVUS
TwinsUK
UK BiLEVE

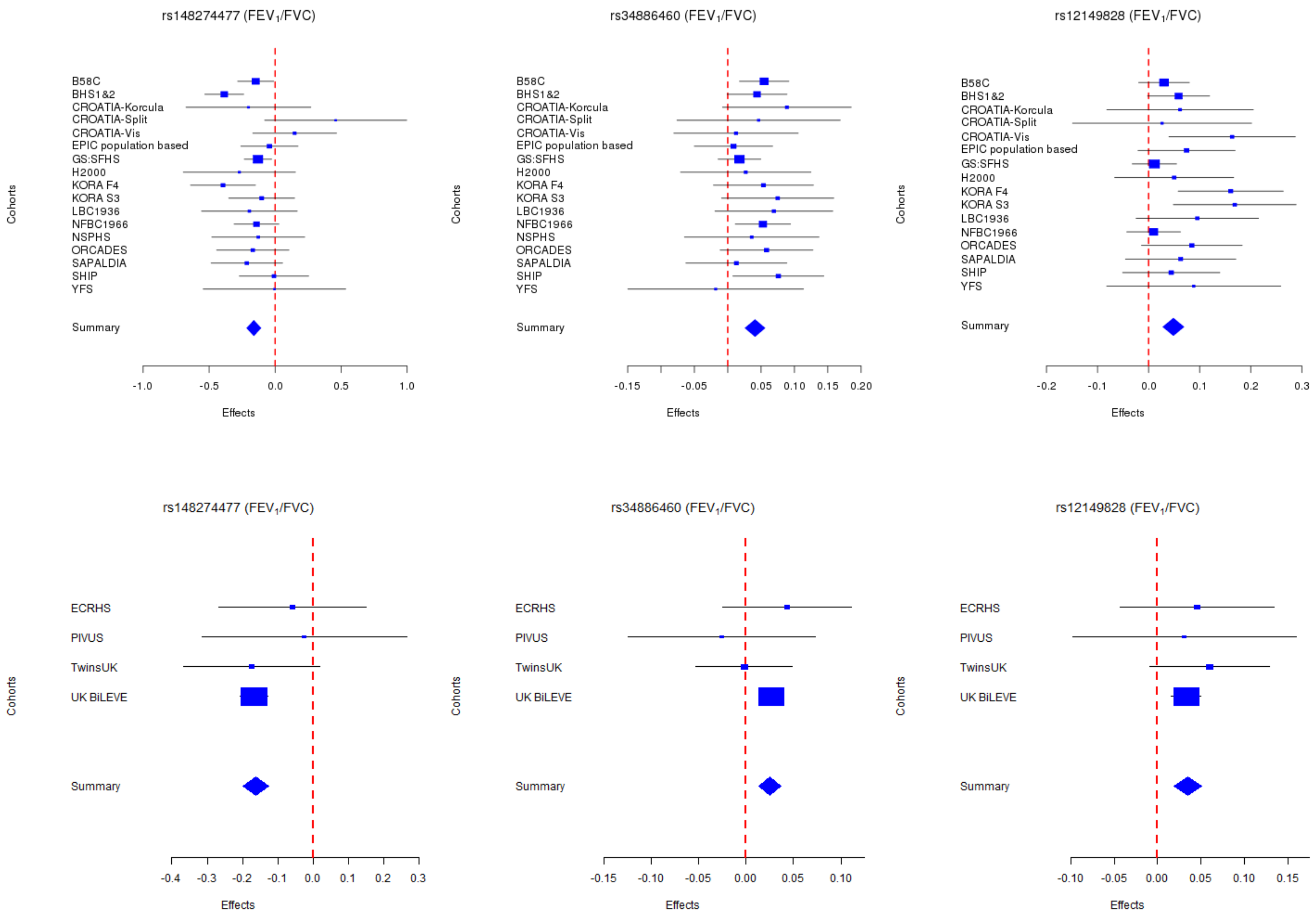
rs148274477 (FEV1/FVC)
rs34886460 (FEV1/FVC)
rs12149828 (FEV1/FVC)
B58C
BHS1&2
CROATIA-Korcula
CROATIA-Split
CROATIA-Vis
EPIC population based
GS:SFHS
H2000
KORA F4
KORA S3
LBC1936
NFBC1966
NSPHS
ORCADES
SAPALDIA
SHIP
YFS
Summary
Cohorts
Effects
-1.0 -0.5 0.0 0.5 1.0
-0.15 -0.05 0.05 0.10 0.15 0.20
-0.2 -0.1 0.0 0.1 0.2 0.3
rs148274477 (FEV1/FVC)
rs34886460 (FEV1/FVC)
rs12149828 (FEV1/FVC)
ECRHS
PIVUS
TwinsUK
UK BiLEVE
Summary
Cohorts
Effects
-0.4 -0.3 -0.2 -0.1 0.0 0.1 0.2 0.3
-0.15 -0.10 -0.05 0.00 0.05 0.10
-0.10 -0.05 0.00 0.05 0.10 0.15

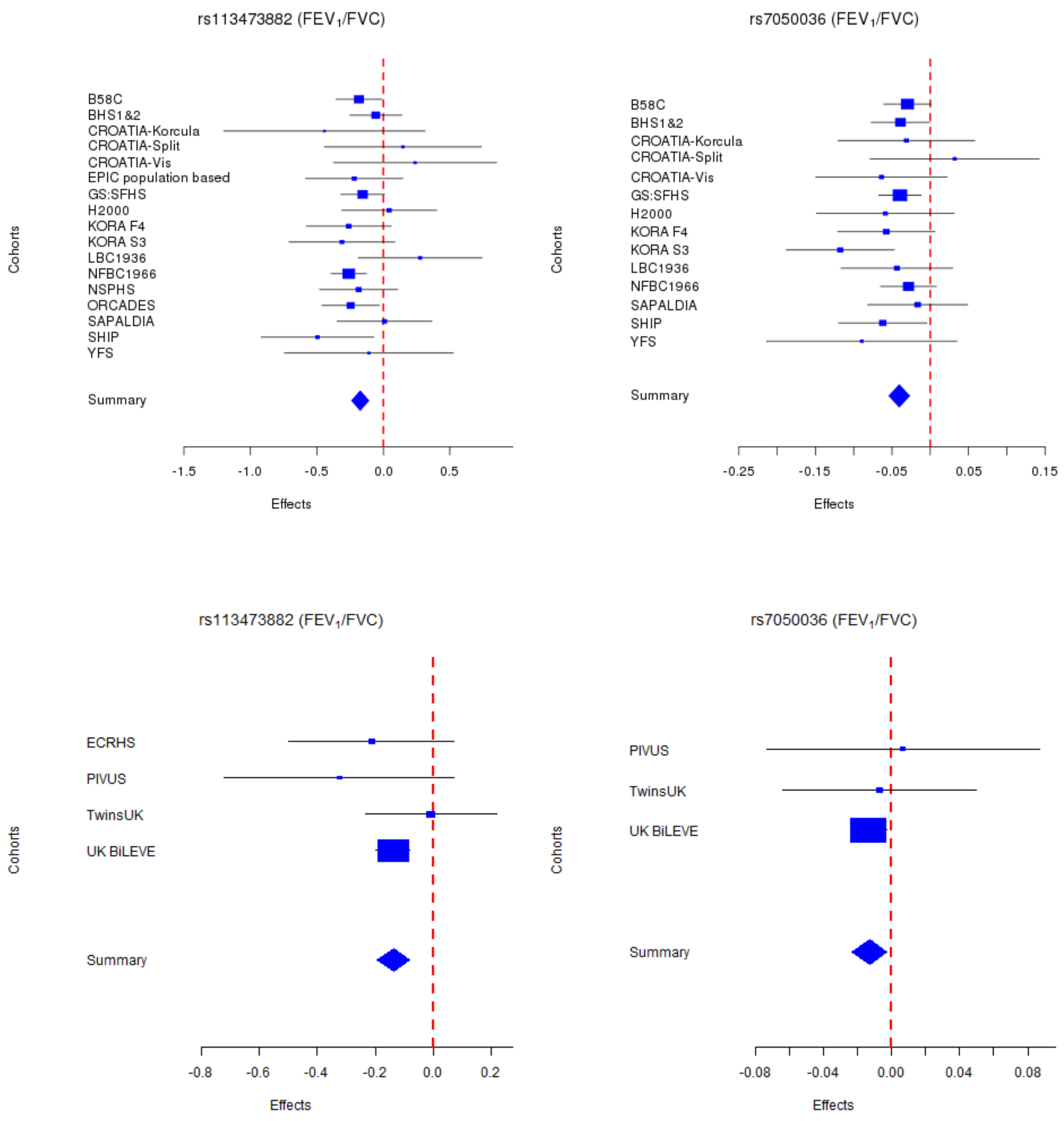

rs113473882 (FEV$_1$/FVC)
B58C
BHS1&2
CROATIA-Korcula
CROATIA-Split
CROATIA-Vis
EPIC population based
GS:SFHS
H2000
KORA F4
KORA S3
LBC1936
NFBC1966
NSPHS
ORCADES
SAPALDIA
SHIP
YFS
Summary
Cohorts
-1.5 -1.0 -0.5 0.0 0.5
Effects
rs7050036 (FEV$_1$/FVC)
B58C
BHS1&2
CROATIA-Korcula
CROATIA-Split
CROATIA-Vis
GS:SFHS
H2000
KORA F4
KORA S3
LBC1936
NFBC1966
SAPALDIA
SHIP
YFS
Summary
Cohorts
-0.25 -0.15 -0.05 0.05 0.15
Effects
rs113473882 (FEV$_1$/FVC)
ECRHS
PIVUS
TwinsUK
UK BiLEVE
Summary
Cohorts
-0.8 -0.6 -0.4 -0.2 0.0 0.2
Effects
rs7050036 (FEV$_1$/FVC)
PIVUS
TwinsUK
UK BiLEVE
Summary
Cohorts
-0.08 -0.04 0.00 0.04 0.08
Effects

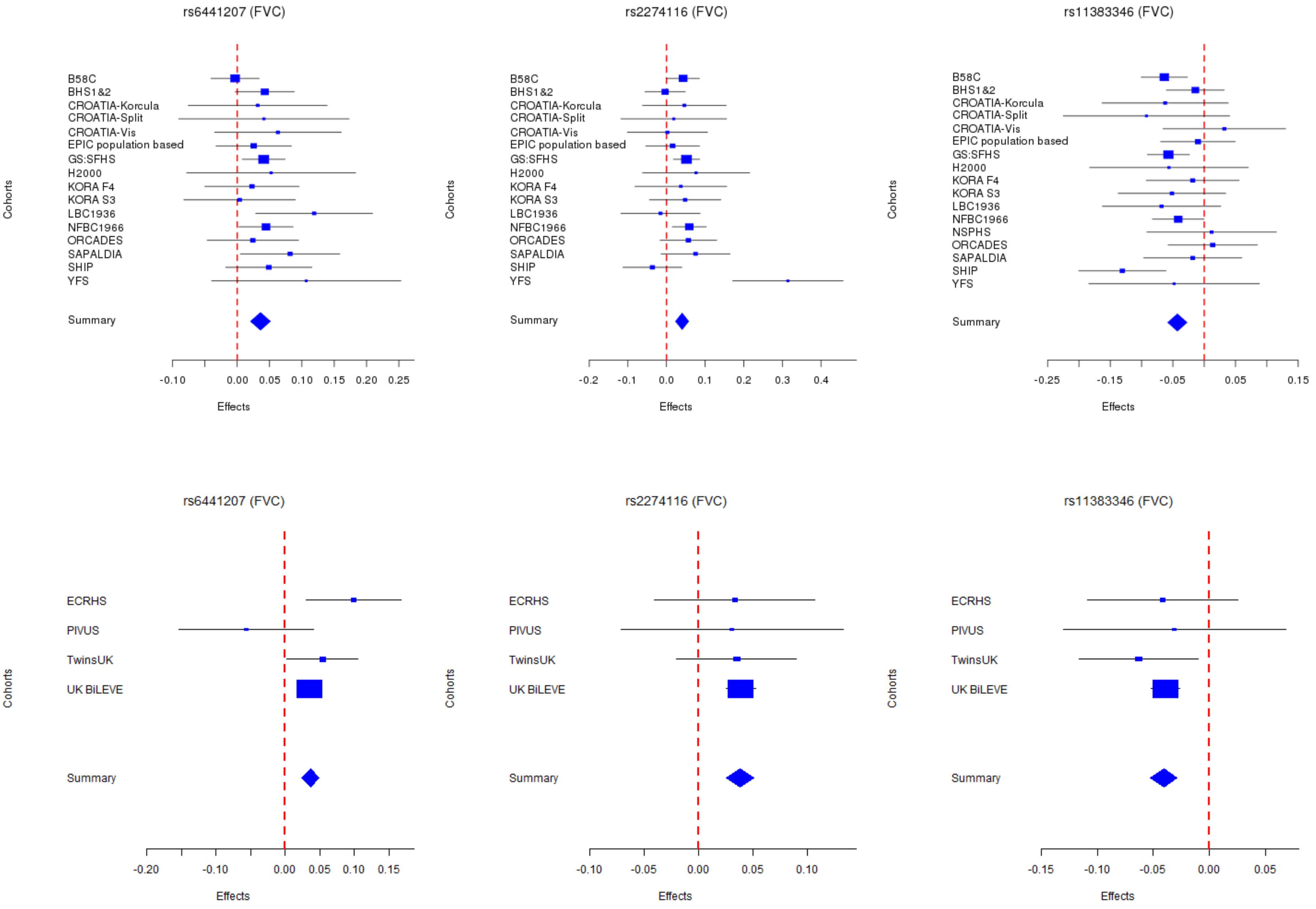
rs6441207 (FVC)
rs2274116 (FVC)
rs11383346 (FVC)
B58C
BHS1&2
CROATIA-Korcula
CROATIA-Split
CROATIA-Vis
EPIC population based
GS:SFHS
H2000
KORA F4
KORA S3
LBC1936
NFBC1966
NSPHS
ORCADES
SAPALDIA
SHIP
YFS
Summary
Cohorts
Effects
-0.10 0.00 0.05 0.10 0.15 0.20 0.25
-0.2 -0.1 0.0 0.1 0.2 0.3 0.4
-0.25 -0.15 -0.05 0.05 0.15
rs6441207 (FVC)
rs2274116 (FVC)
rs11383346 (FVC)
ECRHS
PIVUS
TwinsUK
UK BiLEVE
Summary
Cohorts
Effects
-0.20 -0.10 0.00 0.05 0.10 0.15
-0.10 -0.05 0.00 0.05 0.10
-0.15 -0.10 -0.05 0.00 0.05

c) Forest plots for the 16 loci associated with lung function for stage 1 and stage 2 separately. Each of the SNPs included in the figure showed genome-wide significant association ($P<5\times10^{-8}$) with either FEV_{1}, FEV_{1}/FVC or FVC after meta-analysing stages 1 and 2. The contributing effect (transformed beta) from each study is shown by a square, with confidence intervals indicated by horizontal lines. The contributing weight of each study to the meta-analysis is indicated by the size of the square. The combined meta-analysis estimate is shown at the bottom of each graph.

Supplementary Figure 3 Transcriptomic profiling of candidate lung function genes in primary human bronchial epithelial cells.

A

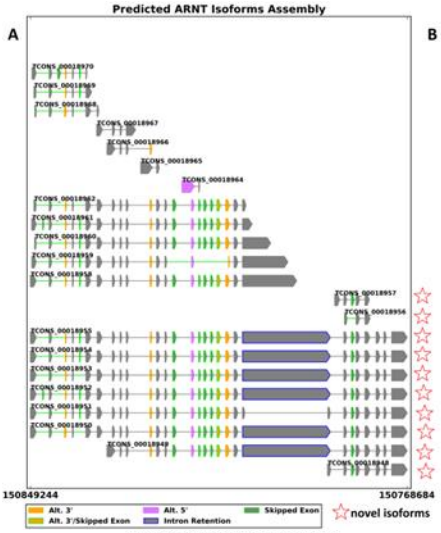

B

Transcript ID	Percent of total pool
TCONS_00018970	Less than 1%
TCONS_00018969	Between 20 and 30%
TCONS_00018968	Less than 1%
TCONS_00018967	Less than 1%
TCONS_00018966	Less than 1%
TCONS_00018965	Less than 1%
TCONS_00018964	Less than 1%
TCONS_00018963	Less than 1%
TCONS_00018962	Between 20 and 30%
TCONS_00018961	Less than 1%
TCONS_00018960	Between 10 and 20%
TCONS_00018959	Between 1 and 5%
TCONS_00018958	Between 30 and 40%
TCONS_00018957	Less than 1%
TCONS_00018956	Less than 1%
TCONS_00018955	Between 1 and 5%
TCONS_00018954	Between 1 and 5%
TCONS_00018953	Less than 1%
TCONS_00018952	Less than 1%
TCONS_00018951	Less than 1%
TCONS_00018950	Less than 1%
TCONS_00018949	Less than 1%
TCONS_00018948	Between 1 and 5%

A

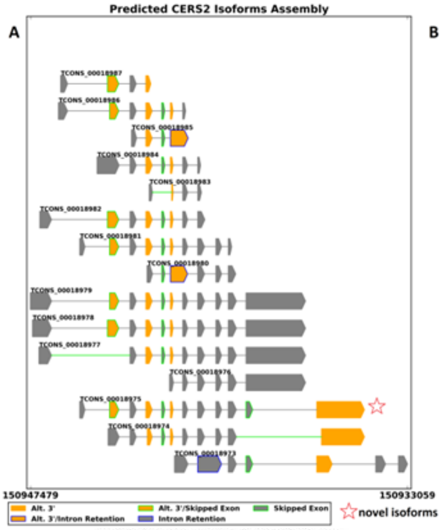

B

Transcript ID	Percent of total pool
TCONS_00018987	Less than 1%
TCONS_00018986	Less than 1%
TCONS_00018985	Less than 1%
TCONS_00018984	Less than 1%
TCONS_00018983	Between 5 and 10%
TCONS_00018982	Between 1 and 5%
TCONS_00018981	Between 40 and 50%
TCONS_00018980	Less than 1%
TCONS_00018979	Between 10 and 20%
TCONS_00018978	Less than 1%
TCONS_00018977	Less than 1%
TCONS_00018976	Between 20 and 30%
TCONS_00018975	Less than 1%
TCONS_00018974	Less than 1%
TCONS_00018973	Less than 1%

A

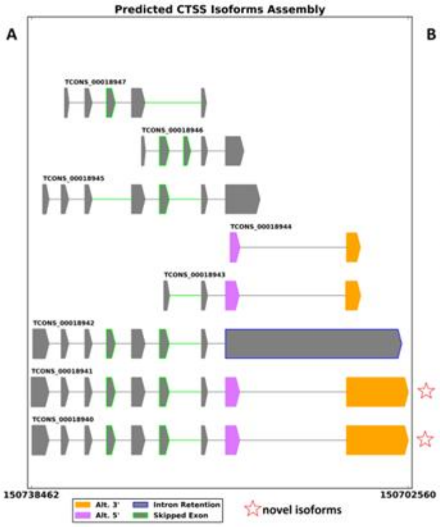

B

Transcript ID	Percent of total pool
TCONS_00018947	Between 40 and 50%
TCONS_00018946	Less than 1%
TCONS_00018945	Between 30 and 40%
TCONS_00018944	Less than 1%
TCONS_00018943	Less than 1%
TCONS_00018942	Between 10 and 20%
TCONS_00018941	Between 1 and 5%
TCONS_00018940	Less than 1%

A

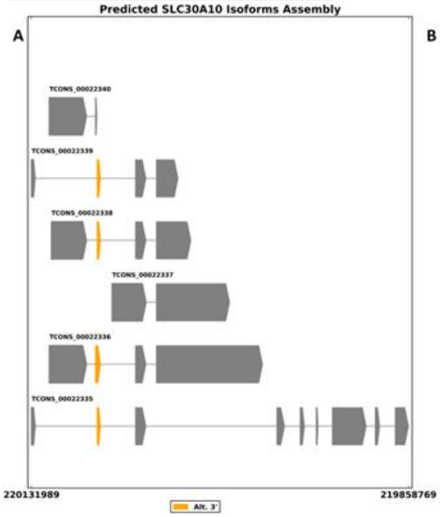

B

Transcript ID	Percent of total pool
TCONS_00022340	Less than 1%
TCONS_00022339	Less than 1%
TCONS_00022338	Less than 1%
TCONS_00022337	Less than 1%
TCONS_00022336	Less than 1%
TCONS_00022335	Less than 1%

A

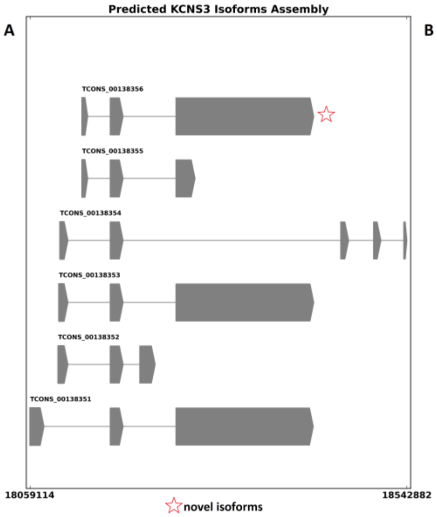

B

Transcript ID	Percent of total pool
TCONS_00138356	Less than 1%
TCONS_00138355	Less than 1%
TCONS_00138354	Less than 1%
TCONS_00138353	More than 90%
TCONS_00138352	Less than 1%
TCONS_00138351	Less than 1%

A

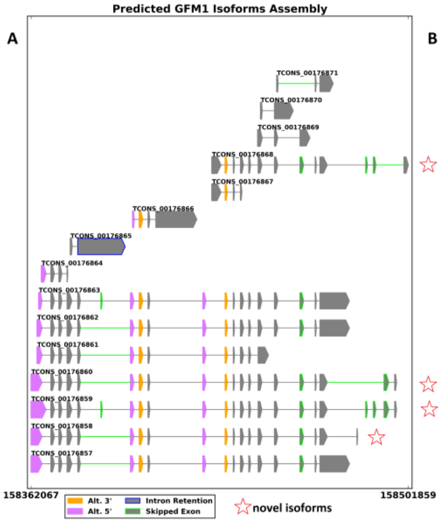

B

Transcript ID	Percent of total pool
TCONS_00176871	Between 1 and 5%
TCONS_00176870	Less than 1%
TCONS_00176869	Less than 1%
TCONS_00176868	Less than 1%
TCONS_00176867	Less than 1%
TCONS_00176866	Less than 1%
TCONS_00176865	Less than 1%
TCONS_00176864	Less than 1%
TCONS_00176863	Between 5 and 10%
TCONS_00176862	Less than 1%
TCONS_00176861	Less than 1%
TCONS_00176860	Less than 1%
TCONS_00176859	Less than 1%
TCONS_00176858	Between 20 and 30%
TCONS_00176857	Between 60 and 70%

A

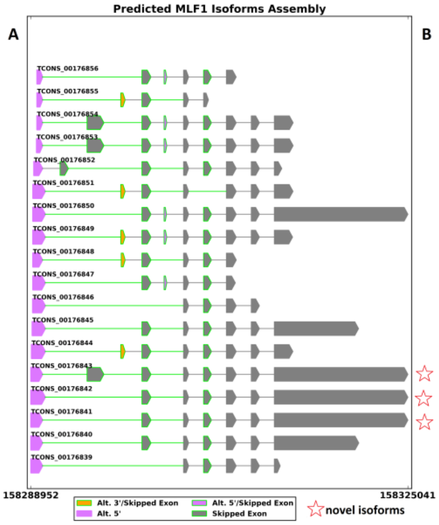

B

Transcript ID	Percent of total pool
TCONS_00176856	Less than 1%
TCONS_00176855	Between 1 and 5%
TCONS_00176854	Less than 1%
TCONS_00176853	Less than 1%
TCONS_00176852	Less than 1%
TCONS_00176851	Less than 1%
TCONS_00176850	Less than 1%
TCONS_00176849	Less than 1%
TCONS_00176848	Between 1 and 5%
TCONS_00176847	Between 10 and 20%
TCONS_00176846	Between 1 and 5%
TCONS_00176845	Between 5 and 10%
TCONS_00176844	Between 30 and 40%
TCONS_00176843	Less than 1%
TCONS_00176842	Less than 1%
TCONS_00176841	Less than 1%
TCONS_00176840	Between 30 and 40%
TCONS_00176839	Between 5 and 10%

A

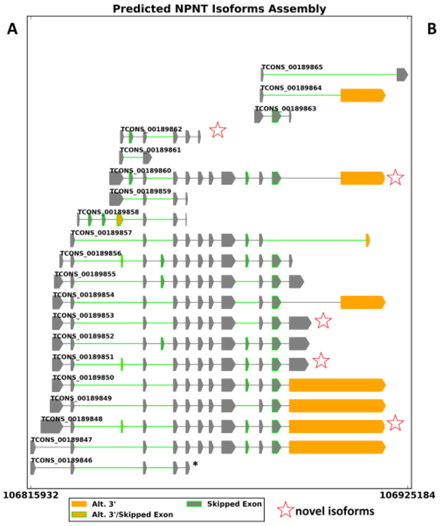

B

Transcript ID	Percent of total pool
TCONS_00189865	Less than 1%
TCONS_00189864	Less than 1%
TCONS_00189863	Less than 1%
TCONS_00189862	Less than 1%
TCONS_00189861	Less than 1%
TCONS_00189860	Less than 1%
TCONS_00189859	Less than 1%
TCONS_00189858	Between 1 and 5%
TCONS_00189857	Less than 1%
TCONS_00189856	Less than 1%
TCONS_00189855	Less than 1%
TCONS_00189854	Less than 1%
TCONS_00189853	Less than 1%
TCONS_00189852	Less than 1%
TCONS_00189851	Between 1 and 5%
TCONS_00189850	Between 60 and 70%
TCONS_00189849	Less than 1%
TCONS_00189848	Between 5 and 10%
TCONS_00189847	Between 20 and 30%
TCONS_00189846	Less than 1%

A

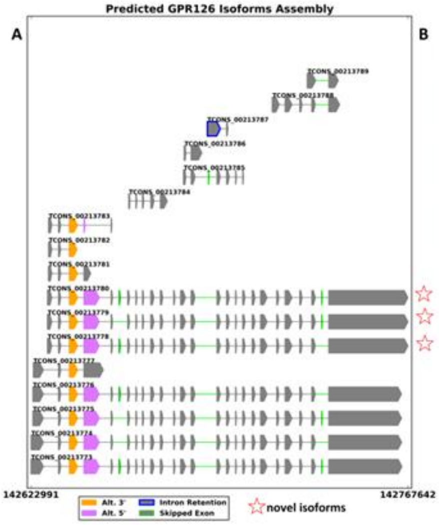

B

Transcript ID	Percent of total pool
TCONS_00213789	Less than 1%
TCONS_00213788	Less than 1%
TCONS_00213787	Less than 1%
TCONS_00213786	Less than 1%
TCONS_00213785	Less than 1%
TCONS_00213784	Less than 1%
TCONS_00213783	Between 1 and 5%
TCONS_00213782	Between 1 and 5%
TCONS_00213781	Between 1 and 5%
TCONS_00213780	Between 5 and 10%
TCONS_00213779	Less than 1%
TCONS_00213778	Less than 1%
TCONS_00213777	Less than 1%
TCONS_00213776	Between 10 and 20%
TCONS_00213775	Between 10 and 20%
TCONS_00213774	Less than 1%
TCONS_00213773	Between 60 and 70%

A

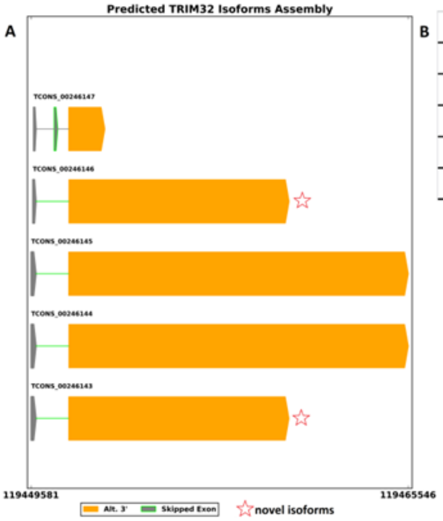

B

Transcript ID	Percent of total pool
TCONS_00246147	Between 1 and 5%
TCONS_00246146	Between 50 and 60%
TCONS_00246145	Between 10 and 20%
TCONS_00246144	Between 5 and 10%
TCONS_00246143	Between 20 and 30%

A

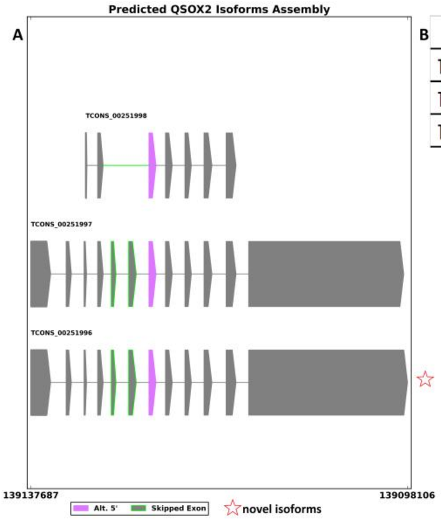

B

Transcript ID	Percent of total pool
TCONS_00251998	Between 1 and 5%
TCONS_00251997	More than 90%
TCONS_00251996	Less than 1%

A

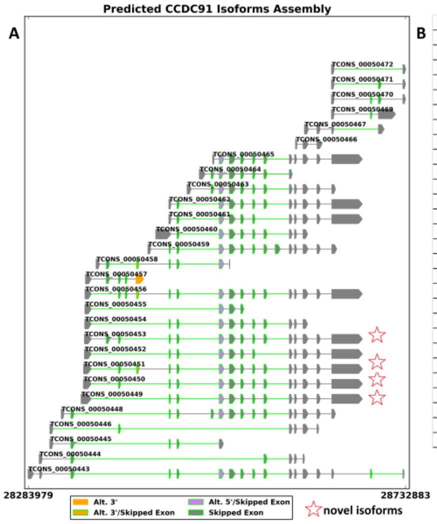

B

Transcript ID	Percent of total pool
TCONS_00050472	Less than 1%
TCONS_00050471	Between 1 and 5%
TCONS_00050470	Between 1 and 5%
TCONS_00050469	Less than 1%
TCONS_00050467	Between 1 and 5%
TCONS_00050466	Less than 1%
TCONS_00050465	Less than 1%
TCONS_00050464	Between 1 and 5%
TCONS_00050463	Less than 1%
TCONS_00050462	Between 30 and 40%
TCONS_00050461	Less than 1%
TCONS_00050460	Less than 1%
TCONS_00050459	Between 1 and 5%
TCONS_00050458	Less than 1%
TCONS_00050457	Less than 1%
TCONS_00050456	Between 5 and 10%
TCONS_00050455	Between 5 and 10%
TCONS_00050454	Between 10 and 20%
TCONS_00050453	Between 1 and 5%
TCONS_00050452	Between 1 and 5%
TCONS_00050451	Between 5 and 10%
TCONS_00050450	Between 5 and 10%
TCONS_00050449	Between 1 and 5%
TCONS_00050448	Between 1 and 5%
TCONS_00050446	Less than 1%
TCONS_00050445	Less than 1%
TCONS_00050444	Less than 1%
TCONS_00050443	Between 1 and 5%

A

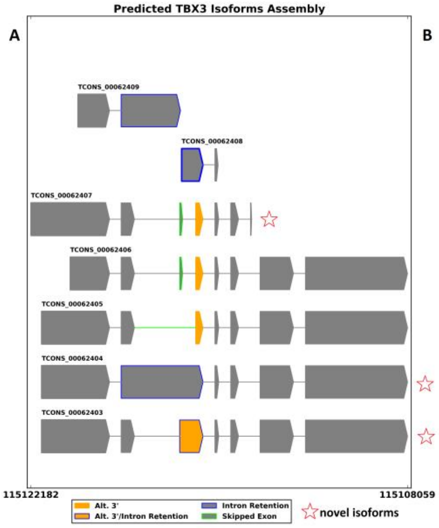

B

Transcript ID	Percent of total pool
TCONS_00062403	Less than 1%
TCONS_00062404	Between 1 and 5%
TCONS_00062405	Between 40 and 50%
TCONS_00062406	Between 30 and 40%
TCONS_00062407	Between 10 and 20%
TCONS_00062408	Between 1 and 5%
TCONS_00062409	Between 5 and 10%

A

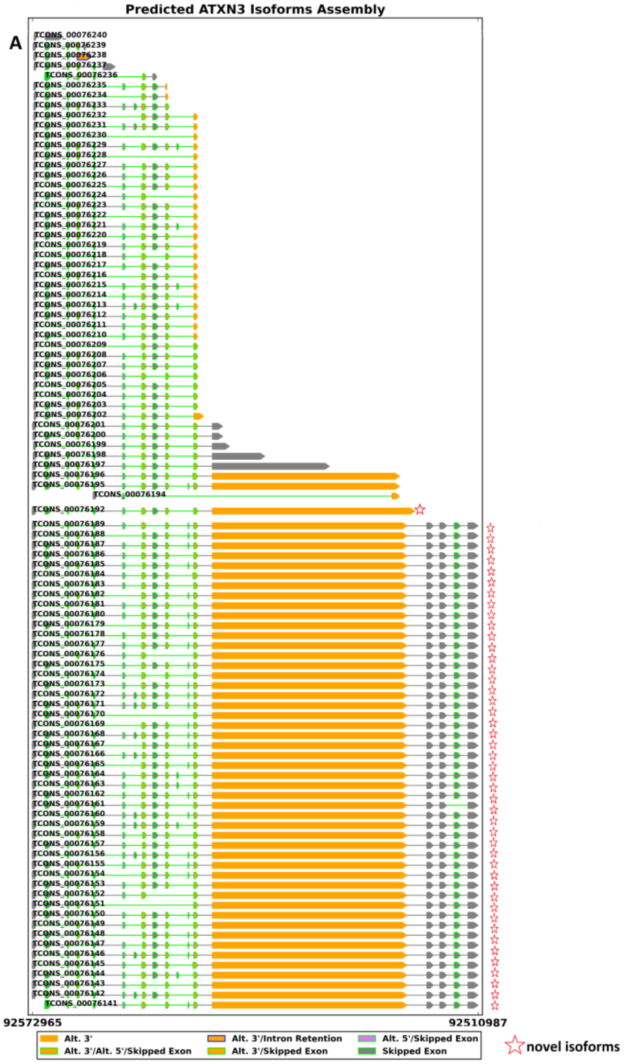

B

Transcript ID	Percent of total pool
TCONS_00076240	Less than 1%
TCONS_00076239	Between 5 and 10%
TCONS_00076238	Between 1 and 5%
TCONS_00076237	Between 1 and 5%
TCONS_00076236	Less than 1%
TCONS_00076235	Less than 1%
TCONS_00076234	Less than 1%
TCONS_00076233	Less than 1%
TCONS_00076232	Less than 1%
TCONS_00076231	Less than 1%
TCONS_00076230	Less than 1%
TCONS_00076229	Less than 1%
TCONS_00076228	Less than 1%
TCONS_00076227	Less than 1%
TCONS_00076226	Less than 1%
TCONS_00076225	Between 1 and 5%
TCONS_00076224	Less than 1%
TCONS_00076223	Less than 1%
TCONS_00076222	Less than 1%
TCONS_00076221	Between 5 and 10%
TCONS_00076220	Less than 1%
TCONS_00076219	Less than 1%
TCONS_00076218	Less than 1%
TCONS_00076217	Less than 1%
TCONS_00076216	Less than 1%
TCONS_00076215	Less than 1%
TCONS_00076214	Between 1 and 5%
TCONS_00076213	Less than 1%
TCONS_00076212	Between 10 and 20%
TCONS_00076211	Less than 1%
TCONS_00076210	Less than 1%
TCONS_00076209	Less than 1%
TCONS_00076208	Less than 1%
TCONS_00076207	Less than 1%
TCONS_00076206	Less than 1%
TCONS_00076205	Between 1 and 5%
TCONS_00076204	Less than 1%
TCONS_00076203	Between 1 and 5%
TCONS_00076202	Between 1 and 5%
TCONS_00076201	Between 1 and 5%
TCONS_00076200	Less than 1%
TCONS_00076199	Between 5 and 10%
TCONS_00076198	Between 10 and 20%
TCONS_00076197	Between 10 and 20%
TCONS_00076196	Between 1 and 5%
TCONS_00076195	Less than 1%
TCONS_00076194	Less than 1%
TCONS_00076192	Less than 1%
TCONS_00076189	Less than 1%
TCONS_00076188	Less than 1%
TCONS_00076187	Less than 1%
TCONS_00076186	Less than 1%
TCONS_00076185	Less than 1%
TCONS_00076184	Less than 1%
TCONS_00076183	Less than 1%
TCONS_00076182	Less than 1%
TCONS_00076181	Less than 1%
TCONS_00076180	Less than 1%
TCONS_00076179	Less than 1%
TCONS_00076178	Less than 1%
TCONS_00076177	Less than 1%
TCONS_00076176	Less than 1%
TCONS_00076175	Less than 1%
TCONS_00076174	Less than 1%
TCONS_00076173	Less than 1%
TCONS_00076172	Less than 1%
TCONS_00076171	Less than 1%
TCONS_00076170	Less than 1%
TCONS_00076169	Less than 1%
TCONS_00076168	Less than 1%
TCONS_00076167	Less than 1%
TCONS_00076166	Less than 1%
TCONS_00076165	Less than 1%
TCONS_00076164	Less than 1%
TCONS_00076163	Less than 1%
TCONS_00076162	Less than 1%
TCONS_00076161	Between 1 and 5%
TCONS_00076160	Less than 1%
TCONS_00076159	Less than 1%
TCONS_00076158	Less than 1%
TCONS_00076157	Less than 1%
TCONS_00076156	Less than 1%
TCONS_00076155	Less than 1%
TCONS_00076154	Less than 1%
TCONS_00076153	Between 1 and 5%
TCONS_00076152	Less than 1%
TCONS_00076151	Less than 1%
TCONS_00076150	Less than 1%
TCONS_00076149	Less than 1%
TCONS_00076148	Less than 1%
TCONS_00076147	Less than 1%
TCONS_00076146	Less than 1%
TCONS_00076145	Less than 1%
TCONS_00076144	Less than 1%
TCONS_00076143	Less than 1%
TCONS_00076142	Less than 1%
TCONS_00076141	Less than 1%

Predicted TRIP11 Isoforms Assembly

A

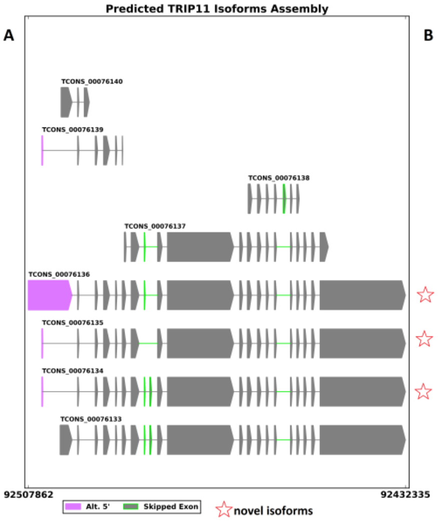

B

Transcript ID	Percent of total pool
TCONS_00076140	Between 20 and 30%
TCONS_00076139	Between 20 and 30%
TCONS_00076138	Less than 1%
TCONS_00076137	Between 50 and 60%
TCONS_00076136	Less than 1%
TCONS_00076135	Less than 1%
TCONS_00076134	Less than 1%
TCONS_00076133	Between 20 and 30%

Predicted RIN3 Isoforms Assembly

A

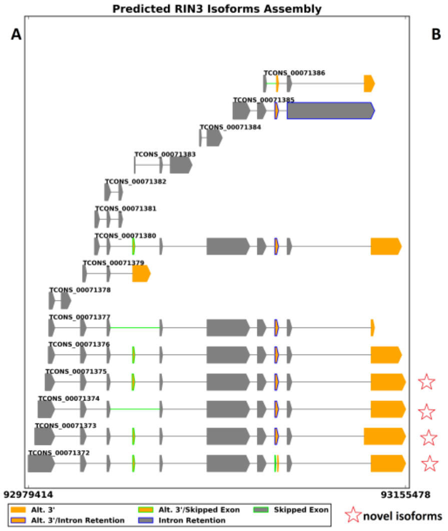

B

Transcript ID	Percent of total pool
TCONS_00071386	Between 1 and 5%
TCONS_00071385	Between 1 and 5%
TCONS_00071384	Less than 1%
TCONS_00071383	Between 1 and 5%
TCONS_00071382	Between 1 and 5%
TCONS_00071381	Less than 1%
TCONS_00071380	Less than 1%
TCONS_00071379	Less than 1%
TCONS_00071378	Between 1 and 5%
TCONS_00071377	Between 5 and 10%
TCONS_00071376	Between 60 and 70%
TCONS_00071375	Less than 1%
TCONS_00071374	Between 5 and 10%
TCONS_00071373	Between 1 and 5%
TCONS_00071372	Less than 1%

Predicted EMP2 Isoforms Assembly

A

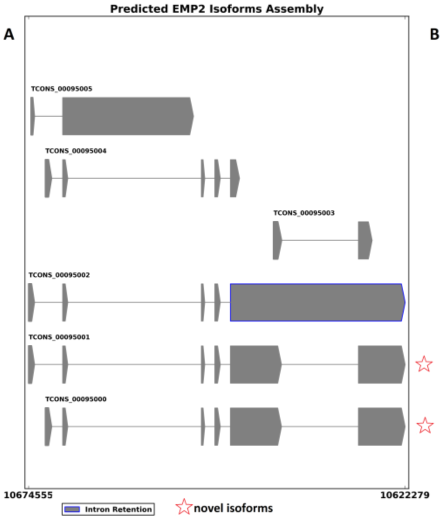

B

Transcript ID	Percent of total pool
TCONS_00095005	Less than 1%
TCONS_00095004	Between 20 and 30%
TCONS_00095003	Less than 1%
TCONS_00095002	Between 70 and 80%
TCONS_00095001	Less than 1%
TCONS_00095000	Less than 1%

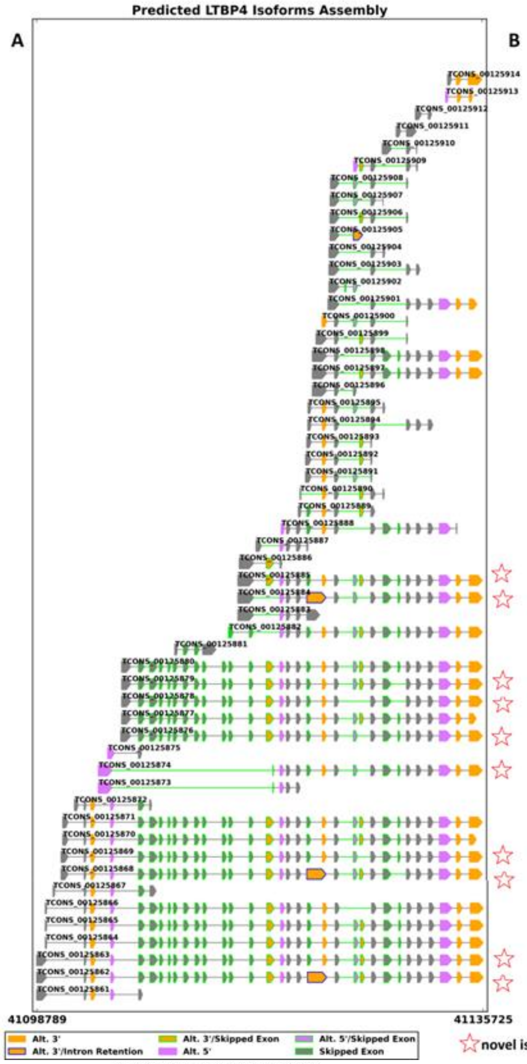

B

Transcript ID	Percent of total pool
TCONS_00125914	Less than 1%
TCONS_00125913	Less than 1%
TCONS_00125912	Between 1 and 5%
TCONS_00125911	Between 1 and 5%
TCONS_00125910	Between 1 and 5%
TCONS_00125909	Between 1 and 5%
TCONS_00125908	Between 5 and 10%
TCONS_00125907	Less than 1%
TCONS_00125906	Between 1 and 5%
TCONS_00125905	Between 1 and 5%
TCONS_00125904	Between 1 and 5%
TCONS_00125903	Less than 1%
TCONS_00125902	Less than 1%
TCONS_00125901	Between 10 and 20%
TCONS_00125900	Less than 1%
TCONS_00125899	Less than 1%
TCONS_00125898	Between 1 and 5%
TCONS_00125897	Between 5 and 10%
TCONS_00125896	Less than 1%
TCONS_00125895	Less than 1%
TCONS_00125894	Less than 1%
TCONS_00125893	Less than 1%
TCONS_00125892	Between 1 and 5%
TCONS_00125891	Less than 1%
TCONS_00125890	Less than 1%
TCONS_00125889	Less than 1%
TCONS_00125888	Between 5 and 10%
TCONS_00125887	Less than 1%
TCONS_00125886	Between 1 and 5%
TCONS_00125885	Between 20 and 30%
TCONS_00125884	Between 1 and 5%
TCONS_00125883	Less than 1%
TCONS_00125882	Less than 1%
TCONS_00125881	Between 1 and 5%
TCONS_00125880	Less than 1%
TCONS_00125879	Between 1 and 5%
TCONS_00125878	Less than 1%
TCONS_00125877	Between 5 and 10%
TCONS_00125876	Less than 1%
TCONS_00125875	Less than 1%
TCONS_00125874	Between 1 and 5%
TCONS_00125873	Less than 1%
TCONS_00125872	Less than 1%
TCONS_00125871	Less than 1%
TCONS_00125870	Less than 1%
TCONS_00125869	Less than 1%
TCONS_00125868	Between 1 and 5%
TCONS_00125867	Less than 1%
TCONS_00125866	Less than 1%
TCONS_00125865	Less than 1%
TCONS_00125864	Less than 1%
TCONS_00125863	Less than 1%
TCONS_00125862	Less than 1%
TCONS_00125861	Less than 1%

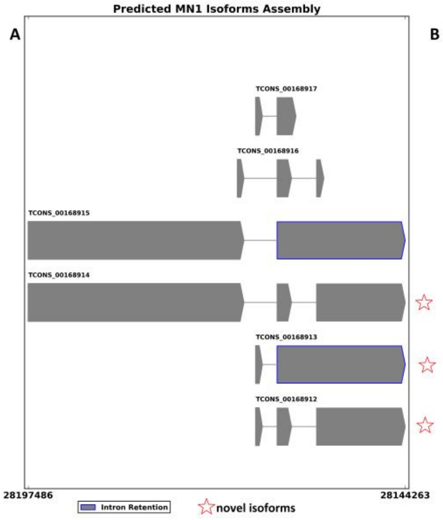

B

Transcript ID	Percent of total pool
TCONS_00168917	Less than 1%
TCONS_00168916	Less than 1%
TCONS_00168915	Between 40 and 50%
TCONS_00168914	Less than 1%
TCONS_00168913	Between 50 and 60%
TCONS_00168912	Less than 1%

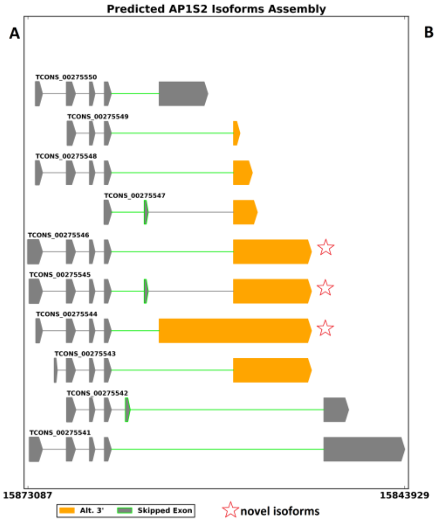

B

Transcript ID	Percent of total pool
TCONS_00275550	Between 1 and 5%
TCONS_00275549	Between 20 and 30%
TCONS_00275548	Less than 1%
TCONS_00275547	Between 1 and 5%
TCONS_00275546	Less than 1%
TCONS_00275545	Between 10 and 20%
TCONS_00275544	Between 5 and 10%
TCONS_00275543	Between 10 and 20%
TCONS_00275542	Less than 1%
TCONS_00275541	Between 30 and 40%

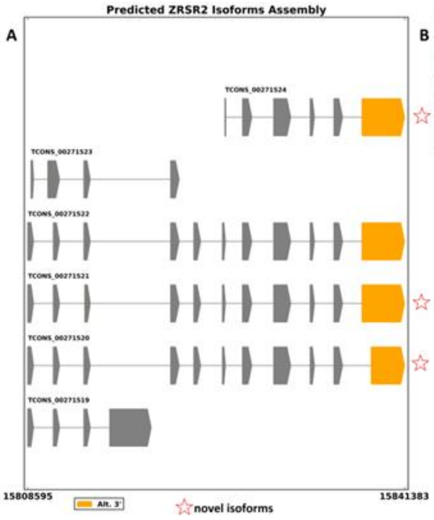

B

Transcript ID	Percent of total pool
TCONS_00271524	Less than 1%
TCONS_00271523	Less than 1%
TCONS_00271522	Between 80 and 90%
TCONS_00271521	Between 10 and 20%
TCONS_00271520	Between 1 and 5%
TCONS_00271519	Between 1 and 5%

Figures show novel and previously described (Ensembl) mRNA isoforms and their percent abundance. A: Individual predicted gene's isoforms with indicated splice variation identified using RNA-seq reference annotation based transcript assembly. Different splice events are described in the box beneath the main graph and novel transcripts are indicated by a star. X-axis contains two outmost genomic coordinates. B: Percent abundance of individual transcripts in primary human bronchial epithelial cells (passage 3) grown under basal conditions.

Supplementary Figure 4 Comparison of results before and after excluding individuals with asthma

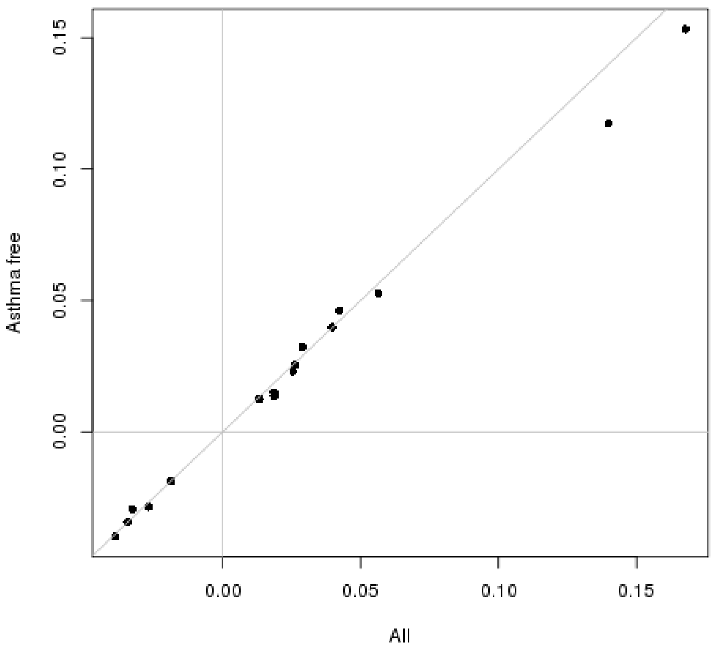

Association results are presented for the 16 novel sentinel variants with the trait that showed the strongest association, for all individuals (“All”, N=48,943) and after removing individuals with asthma (“Asthma free”, N=41,455) using UK BiLEVE data.

Supplementary Figure 5 Comparison of results obtained with 1000 Genomes variants and with HapMap for autosomal signals

a) Region plots for the new signals using HapMap and 1000 Genomes variants

i. rs6681426

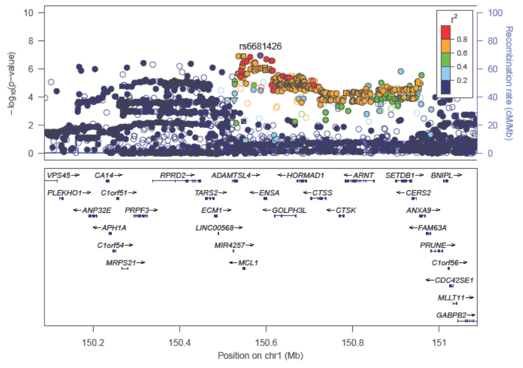

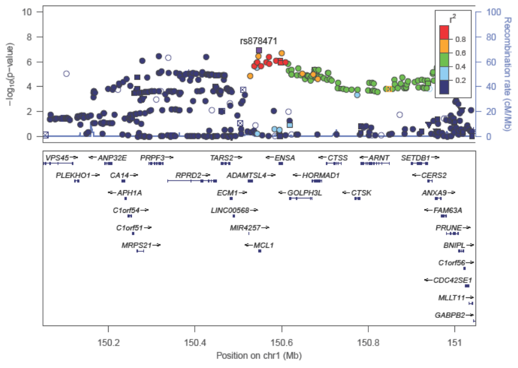

ii. rs201204531 (chr1:219963090:I)

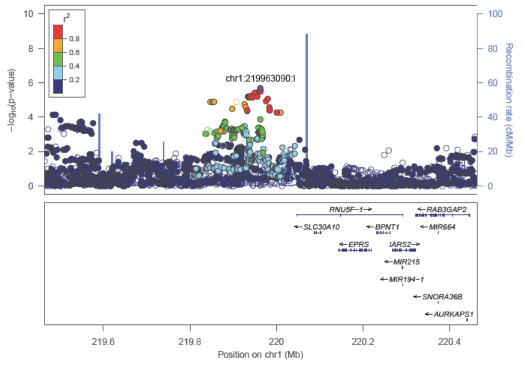

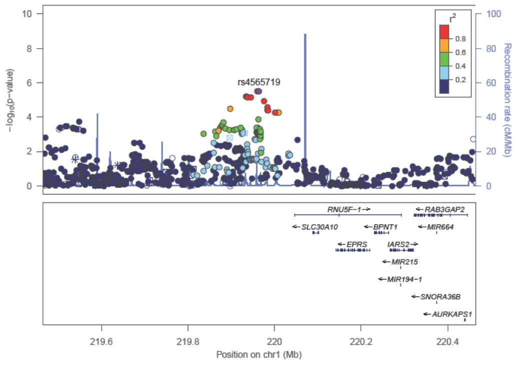

iii. rs61067109

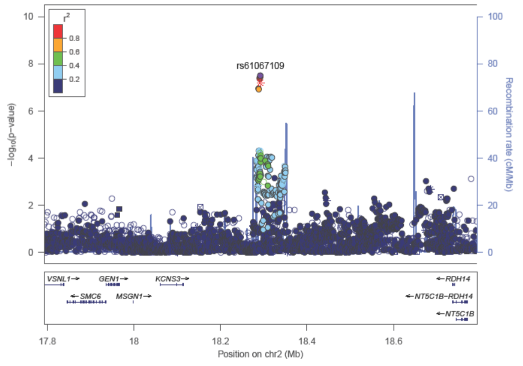

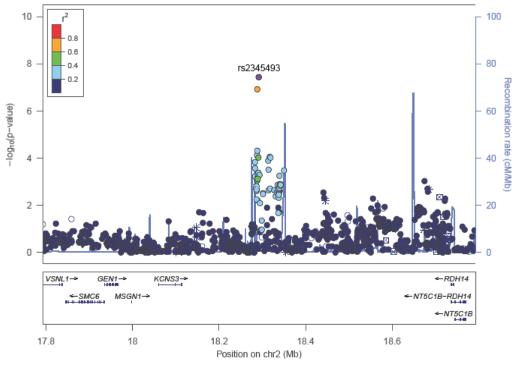

iv. rs6441207

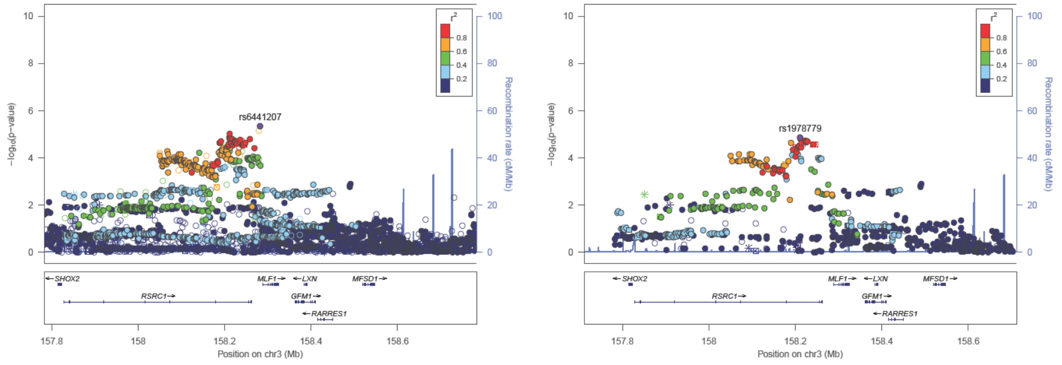

v. rs6856422

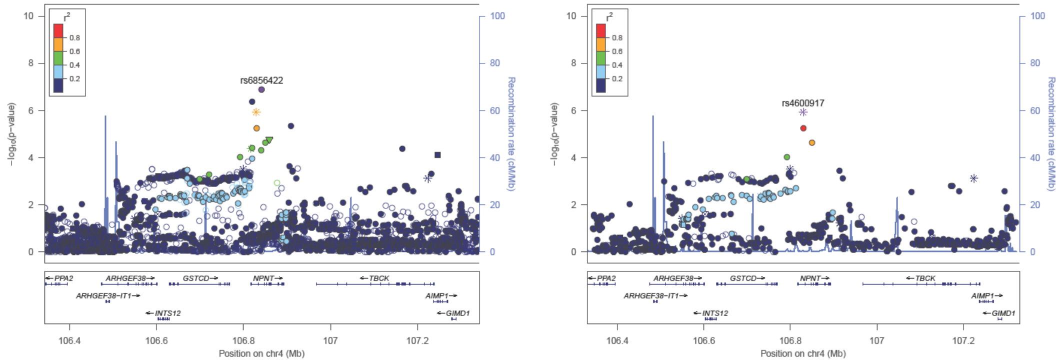

vi. rs148274477

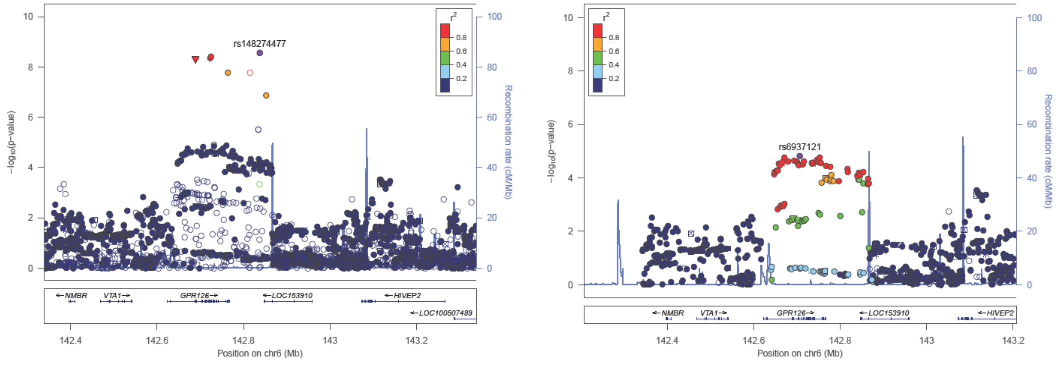

vii. rs34886460 (chr9:119359372:I)

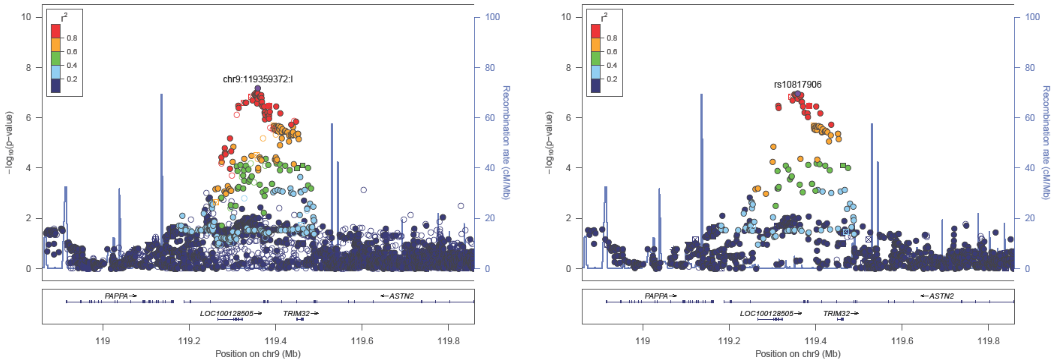

viii. rs2274116

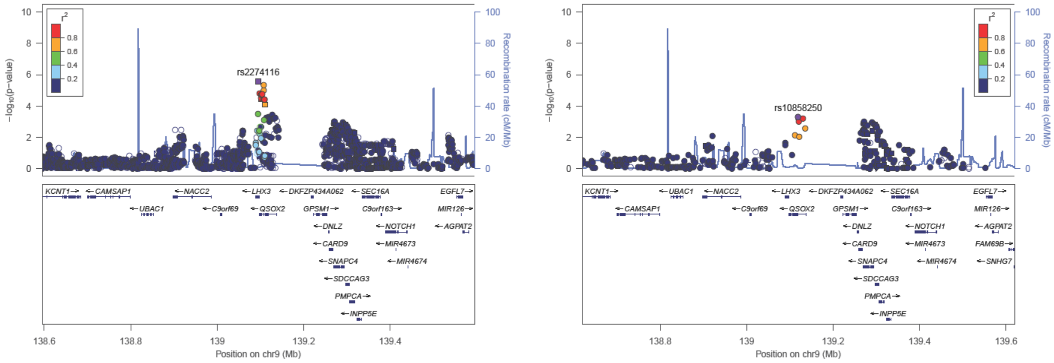

ix. rs11383346 (chr12:28283187:I)

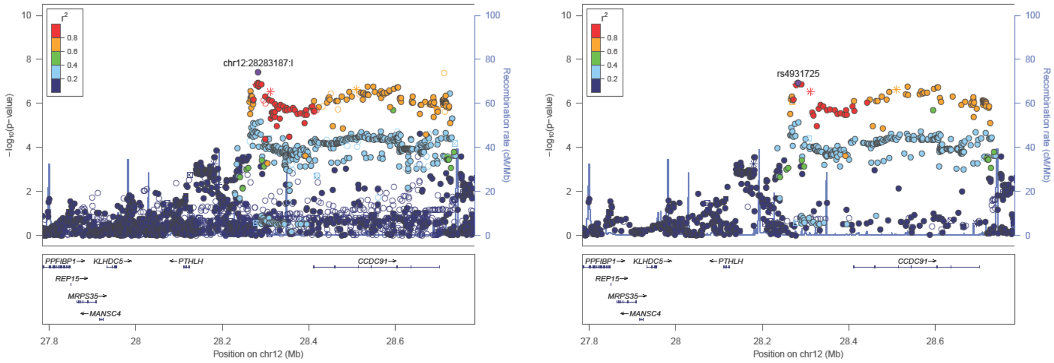

x. rs10850377

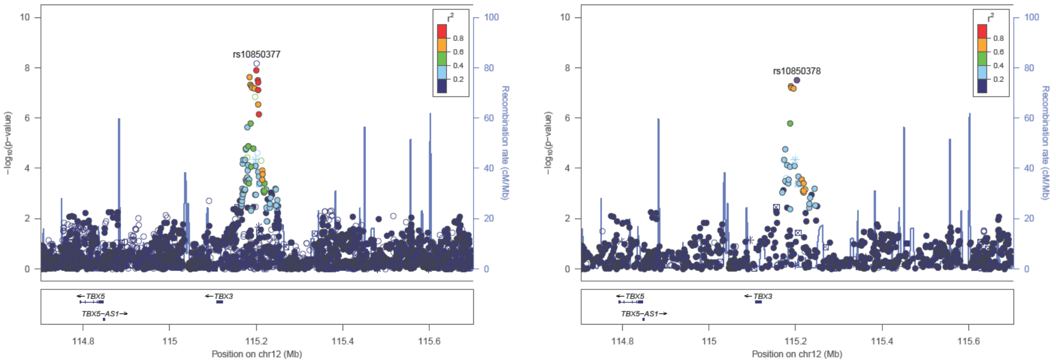

xi. rs7155279

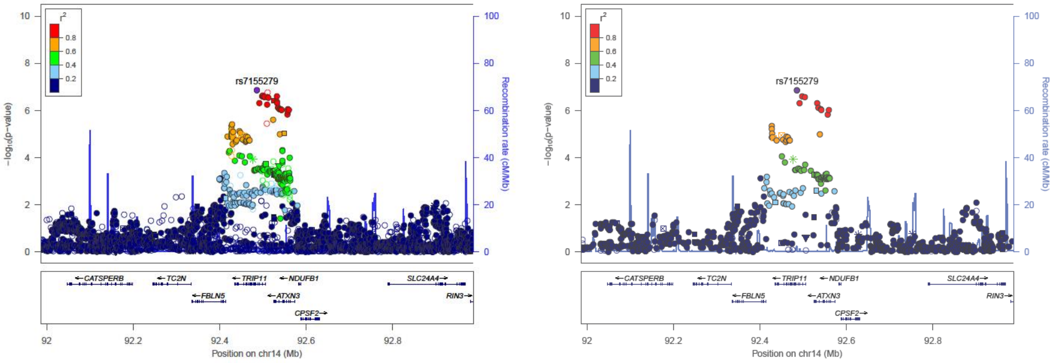

xii. rs117068593

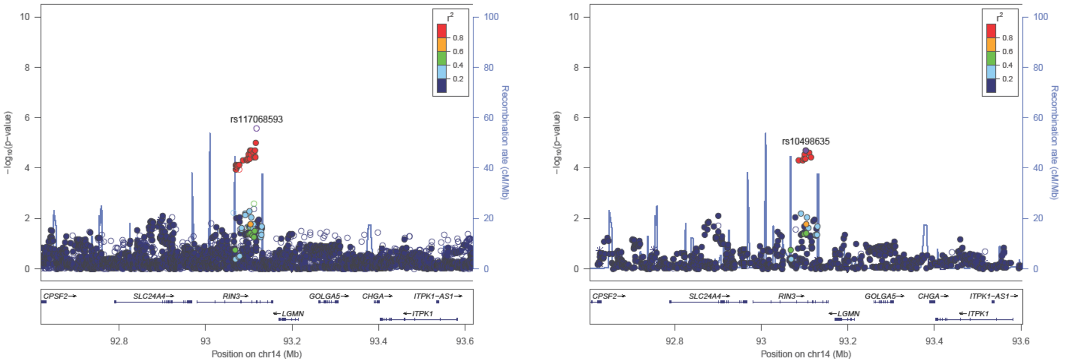

xiii. rs12149828

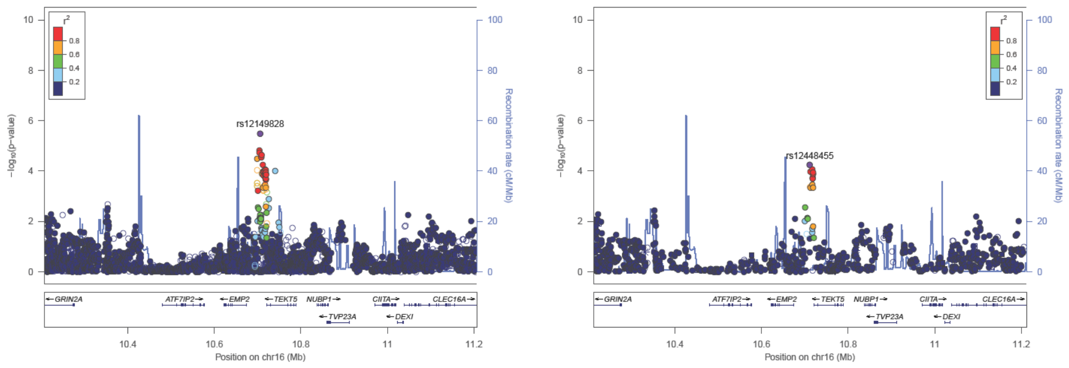

xiv. rs113473882

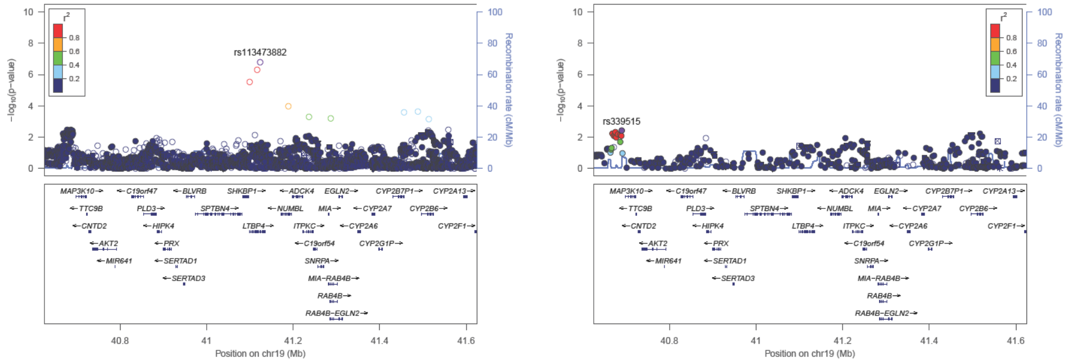

xv. rs134041

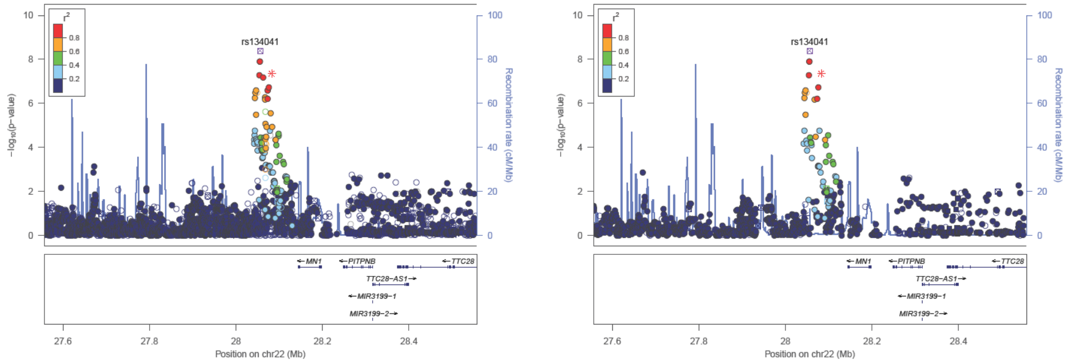

Region plots are presented with the 1000 Genomes results including all 1000 Genomes variants with *N* effective ≥70% (plot on the left) and including only HapMap variants with *N* effective ≥70% extracted from the previous HapMap meta-analysis [5] (plot on the right) for all the new signals.

b) Scatter plot of minor allele frequencies for HapMap sentinel variants vs. 1000 Genomes sentinel variants

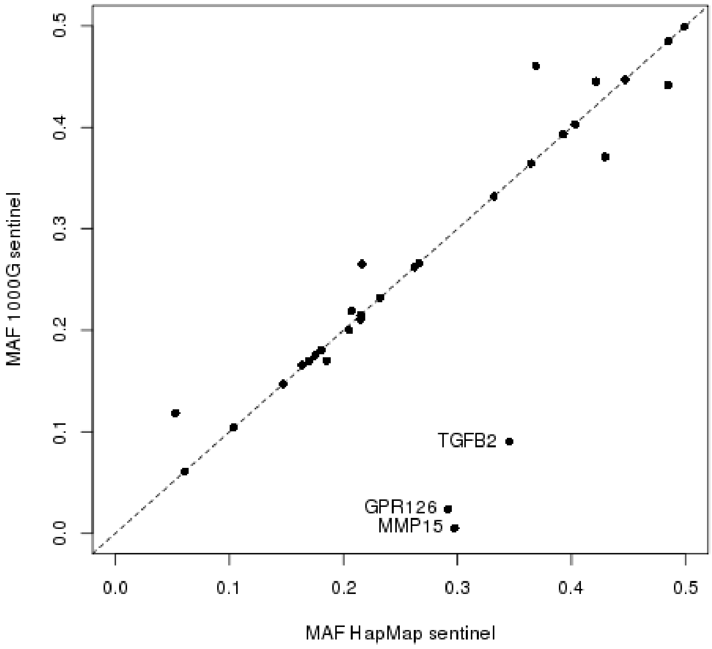

Shown are MAF for sentinel variants defined using 1000 Genomes stage 1 results vs. MAF for sentinel variants defined using 1000 Genomes stage 1 results restricted to HapMap variants only, in previously reported regions[1-5], defined as the previously reported sentinel variant[3,4] +/- 500kb either side (*MFAP2, TGFB2, EFEMP1, TNS1, HDAC4, RARB, MECOM, FAM13A, GSTCD, HHIP, SPATA9, HTR4, ADAM19, BMP6, ZKSCAN3, NCR3, AGER, ARMC2, GPR126, PTCH1, CDC123, C10orf11, HSD17B12, PRDM11, LRP1, CCDC38, THSD4, MMP15, CFDP1, WWOX, KCNJ2, KCNE2*).

Supplementary Tables:

Supplementary Table 1 Sample population characteristics and genotyping platform details for each study in stage1 and stage2

a) Sample population characteristics for each study

Characteristics are shown for studies analysed in stage 1 (GWAS meta-analysis) and stage 2 (follow-up). Stage 1 studies: B58C (B58C-T1DGC, British 1958 Birth Cohort–Type 1 Diabetes Genetics Consortium and B58C-WTCCC, British 1958 Birth Cohort–Wellcome Trust Case Control Consortium); BHS1&2, Busselton Health Study 1 and 2; the CROATIA- Korcula study; the CROATIA-Split study; the CROATIA-Vis study; EPIC population based, European Prospective Investigation into Cancer and Nutrition Cohort; GS:SFHS, Generation Scotland: Scottish Family Health Study; H2000, Finnish Health 2000 survey; KORA F4, Cooperative Health Research in the Region of Augsburg; KORA S3, Cooperative Health Research in the Region of Augsburg; LBC1936, Lothian Birth Cohort 1936; NFBC1966, Northern Finland Birth Cohort of 1966; NSPHS, Northern Sweden Population Health Study; ORCADES, Orkney Complex Disease Study; SHIP, Study of Health in Pomerania; YFS, the Young Finish Study. Stage 2 studies: ECRHS, the European Community Respiratory Health Survey; PIVUS, Prospective Investigation of the Vasculature in Uppsala Seniors; the TwinsUK study; UK BiLEVE, UK Biobank Lung Exome Variant Evaluation study.

Study name	N Total	N male	N female	Age range (y) at lung function measurement	Mean age, y (s.d.)	Mean height, cm (s.d.)	Mean FEV_1, L (s.d.)	Mean FVC, L (s.d.)	Mean FEV_1/FVC (s.d.)	N never smokers	N ever smokers	Lambda FEV_1	Lambda FVC	Lambda factor FEV_1/FVC
**Stage 1**														
B58C	5934	2955	2979	44-45	45.12 (0.38)	169.43 (9.29)	3.30 (0.76)	4.19 (0.98)	0.79 (0.08)	1709	4225	1.022	1.011	1.024
BHS1&2	4355	1922	2433	17-97	51.21 (17.00)	168.00 (9.39)	3.01 (0.96)	3.88 (1.16)	0.77 (0.07)	2301	2054	1.015	1.017	1.015
CROATIA-Korcula	826	302	524	18-90	55.63 (13.50)	168.10 (9.20)	2.72 (0.83)	3.29 (0.96)	0.83 (0.1)	403	423	1.003	1.018	1.017
CROATIA-Split	493	210	283	18-85	49.08 (14.63)	172.60 (9.49)	3.19 (0.91)	3.80 (1.06)	0.84 (0.08)	239	254	1.003	1.011	0.997
CROATIA-Vis	925	390	535	18-88	55.90 (15.51)	167.80 (9.88)	3.42 (1.21)	4.41 (1.42)	0.77 (0.09)	388	537	1.002	0.982	0.969
EPIC population based	2339	1102	1237	39-77	59.20 (9.00)	167.10 (8.80)	2.50 (0.72)	3.04 (0.90)	0.83 (0.11)	1062	1277	1.005	1.008	1.014
GS:SFHS	8093	3385	4708	18-98	51.60 (13.34)	167.90 (9.55)	2.78 (0.87)	3.91 (1.01)	0.71 (0.01)	4319	3774	1.003	1.014	1.013
H2000	821	394	427	30-75	50.47 (10.91)	169.10 (9.14)	3.29 (0.9)	4.16 (1.07)	0.79 (0.07)	249	572	0.999	1.002	1
KORA F4	1474	717	757	41-84	55.08 (9.90)	169.15 (9.42)	3.23 (0.85)	4.19 (1.05)	0.77 (0.07)	556	918	0.998	1.003	1.008
KORA S3	1147	551	596	28-89	50.82 (15.23)	169.22 (9.32)	3.34 (0.90)	4.10 (1.06)	0.81 (0.08)	520	627	1.009	1.012	1.018
LBC1936	991	501	490	68-71	69.55 (0.84)	166.44 (8.93)	2.38 (0.67)	3.04 (0.87)	0.79 (0.10)	437	554	1.011	1.05	1.002
NFBC1966	4563	2180	2383	31-31	31.00 (0)	171.00 (9.54)	3.93 (0.80)	4.71 (0.99)	0.84 (0.07)	1648	2915	1.005	0.99	0.996
NSPHS	871	400	471	14-91	49.20 (20.00)	164.00 (10.10)	2.92 (0.90)	3.53 (1.06)	0.83 (0.09)	750	121	0.996	1.011	0.977
ORCADES	1802	719	1083	16-91	54.00 (15.00)	166.00 (9.20)	2.89 (0.83)	3.61 (0.99)	0.79 (0.08)	1022	780	1.016	1.013	1.009
SAPALDIA	1378	665	713	18-61	41.30 (11.20)	169.47 (9.12)	3.53 (0.86)	4.50 (1.04)	0.78 (0.08)	631	747	0.995	1.001	1.012
SHIP	1768	863	905	25-85	52.43 (13.58)	169.66 (9.12)	3.28 (0.89)	3.87 (1.03)	0.85 (0.06)	770	998	1.019	1.007	1.005
YFS	419	198	221	30-47	38.88 (5.07)	172.25 (8.90)	3.73 (0.75)	4.68 (0.99)	0.8 (0.06)	233	186	1.012	1.02	0.997
Total stage 1 sample size	38199									17237	20962			

Study name	N Total	N male	N female	Age range (y) at lung function measurement	Mean age, y (s.d.)	Mean height, cm (s.d.)	Mean FEV_1, L (s.d.)	Mean FVC, L (s.d.)	Mean FEV_1/FVC (s.d.)	N never smokers	N ever smokers	Lambda FEV_1	Lambda FVC	Lambda factor FEV_1/FVC
**Stage 2**														
ECRHS	1747	823	924	20-48	33.97 (7.16)	170.32 (9.47)	3.73 (0.82)	4.54 (1.03)	0.82 (0.07)	763	984	NA	NA	NA
PIVUS	837	406	431	70-72	70.20 (0.17)	168.93 (9.21)	2.44 (0.68)	3.21 (0.87)	0.76 (0.10)	409	428	NA	NA	NA
TwinsUK	3023	444	2579	19-82	53.08 (13.15)	161.68 (6.21)	2.63 (0.61)	3.28 (0.65)	0.80 (0.08)	1865	1158	NA	NA	NA
UK BiLEVE	48943	24489	24454	40-70	56.93 (7.89)	168.7 (9.13)	2.65 (0.87)	3.59 (1.05)	0.73 (0.08)	24483	24460	NA	NA	NA
Total stage 2 sample size	54550									27520	27030			

b) Study, genotyping, imputation and genotype-phenotype data

Imp'n, imputation.

Study name	GWAS platform	Calling algorithm	Individual call rate filter (applied before imp'n)	SNP call rate filter (applied before imp'n)	SNP HWE *P* filter (applied before imp'n)	SNP MAF filter (applied before imp'n)	Other filters	No of SNPs after filtering (before imp'n)	Imputation software and version	NCBI; 1000 Genomes version for imp'n	Genotype-phenotype association software
**Stage 1**											
B58C	Illumina 550k/610k	GenCall	None	>=95%	≥0.0001 (tested on females only for chromosome X)	≥1%	Consistent allele frequencies across data deposits (*P*≥0.0001 for pairwise comparisons) and for chrX SNPs, consistent allele frequencies between males and females (*P*≥0.0001).	500,521 (including 11,696 chrX)	MACH 1.0.18 & Minimac 2012-11-16	Phase 1 March 2012	probABEL 0.1-9e
BHS1&2	Illumina 610-Quad (N=1,168) & Illumina 660W-Quad (N=3,428)	BeadStudio (N=1,168) & GenomeStudio (N=3,428)	0.95	0.95	1.00E-06	0.01	Individuals were removed if they had sex inconsistencies, had heterozygosity > 5 s.d. from the mean, were PCA outliers, were 1 individual from a pair of duplicates or had IBD inconsistencies.	521,307	Minimac and MACH1 v1.0.18	b37; Phase 1 March 2012	ProbABEL
CROATIA-Korcula	Illumina HumanHap370CNV duo chip	BeadStudio	97%	98%	1.00E-06	0.01		316,879	SHAPEIT2, IMPUTE2	b37; ALL (Phase 1 integrated release v3, April 2012)	ProbABEL
CROATIA-Split	Illumina HumanHap370CNV quad chip	BeadStudio	97%	98%	1.00E-06	0.01		321,727	SHAPEIT2, IMPUTE2	b37; ALL (Phase 1 integrated release v3, April 2012)	ProbABEL

Study name	GWAS platform	Calling algorithm	Individual call rate filter (applied before imp'n)	SNP call rate filter (applied before imp'n)	SNP HWE *P* filter (applied before imp'n)	SNP MAF filter (applied before imp'n)	Other filters	No of SNPs after filtering (before imp'n)	Imputation software and version	NCBI; 1000 Genomes version for imp'n	Genotype-phenotype association software
CROATIA-Vis	Illumina Infinium HumanHap300 BeadChip	BeadStudio	97%	98%	1.00E-06	0.01		273,671	SHAPEIT2, IMPUTE2	b37; ALL (Phase 1 integrated release v3, April 2012)	ProbABEL
EPIC population based	Affymetrix 500K	BRLMM	94%	90%	1.00E-06	0.01	Heterozygosity, ethnic outlier	392,573	IMPUTE 2.2.2	Mar-12	SNPTEST 2.4.0
GS:SFHS	Illumina OmniExpress+Exome	GenomeStudio	97% (Omni); 99% (Exome)	98%	1.00E-06	0.01 (Omni); 0.0001 (Exome)		690,759	SHAPEIT2, IMPUTE2	b37; ALL (Phase 1 integrated release v3, April 2012)	ProbABEL
H2000	Illumina HumanHap 610K	Illuminus	0.95	0.95 (0.99 for SNPs with MAF < 0.05)	1.00E-06	0.01		553,722	IMPUTE version 2.2.2	1,000 Genomes haplotypes -- Phase I integrated variant set release (v3) in NCBI build 37 (hg19) coordinates	SNPTest
KORA F4	Affymetrix Axiom	Affymetrix Software	0.97	0.98	5x10-6	0.01	-missmatch of phenotypic and genetic gender<br>- 5s.d. from mean heterozygosity rate<br>- check for European ancestry<br>- check for population outlier	523,260 (chr 1-26)<br>508,532 (chr 1-22)<br>14,096(chrX-nonPAR)<br>444(chrX-PAR1)<br>58(chrX-PAR2)	SHAPEIT v2, IMPUTE v2.3.0	1000g phase1 all (ALL_1000G_phase1integrated_v3_impute_mac1)	SNPTEST v2.4.1

Study name	GWAS platform	Calling algorithm	Individual call rate filter (applied before imp'n)	SNP call rate filter (applied before imp'n)	SNP HWE *P* filter (applied before imp'n)	SNP MAF filter (applied before imp'n)	Other filters	No of SNPs after filtering (before imp'n)	Imputation software and version	NCBI; 1000 Genomes version for imp'n	Genotype-phenotype association software
KORA S3	Illumina Omni 2.5/ Illumina Omni Express	Genome Studio	0.97	0.98	5x10-6	0.01	person wise: - missmatch of phenotypic and genetic gender<br>- 5s.d. from mean heterozygosity rate<br>- check for European ancestry<br>- check for population outlier<br>SNP wise: only SNPs that were genotyped with good quality on both chips	600641 (chr 1-26)<br>588307 (chr 1-22)<br>14625 (chrX-nonPAR)	SHAPEIT v2, IMPUTE v2.3.0	1000g phase1 all (ALL_1000G_phase1integrated_v3_impute_mac1)	SNPTEST v2.4.1
LBC1936	Illumina 610-Quadv1	GenomeStudio	0.95	0.98	≥0.001	0.01		549,692	minimac 2012-11-16	version 3, cosmopolitan	mach2qtl
NFBC1966	Illumina HumanCNV-370DUO Analysis BeadChip	Beadstudio	95%	95 % (99 % if MAF < 5 %)	1.00E-04	0.01	Duplicates concordance < 99%; IBS pairwise sharing>0.20; withdrew consent; gender mismatch; heterozygosity outliers>0.29; MDS outliers.	309,948	Impute version 2.3.0	1000G March 2012 pphase1 integrated v3	SNPTEST
NSPHS	Illumina Infinum HapMap 300 v2 & Illumina Human OmniExpress	BeadStudio (Infinum) & GenoneStudio (OmniExpress)	0.9	0.95	3.2E-08 (Infinum) & 1.4E-08 (OmniExpress)	0.01	FDR level of heterozygosity 0.01	306,086 (Infinum) & 631503 (OmniExpress)	Impute2 (v 2.2.2)	hg19	ProbABEL

Study name	GWAS platform	Calling algorithm	Individual call rate filter (applied before imp'n)	SNP call rate filter (applied before imp'n)	SNP HWE *P* filter (applied before imp'n)	SNP MAF filter (applied before imp'n)	Other filters	No of SNPs after filtering (before imp'n)	Imputation software and version	NCBI; 1000 Genomes version for imp'n	Genotype-phenotype association software
ORCADES	Illumina Hap300, Illumina Omni1 & Illumina OmniX	Beadstudio using Hap300v2 cluster file (Hap300) & Genome Studio, using Illumina cluster files (Omni1 & OmniX)	98%	97%	1.00E-06	1% (Hap300) & monomorphic (Omni & OmniX)	Subject Heterozygosity FDR<1%	287,208 (Hap300), 843723 (Omni) & 654651 (OmniX)	shapeit.v2.r644.+impute_v2.2.2_x86_64_static/impute2	1000G Phase I Integrated Release Version 3 Haplotypes (2010-11 data freeze, 2012-03-14 haplotypes).	probABEL v. 0.4.3
SAPALDIA	Illumina 610k quad	Gencall	95%	95%	1.00E-06	0.01	none	545,131	Mach 1.0.16.a, minimac-omp RELEASE STAMP 2012-05-29 (autosomes) & MiniMac RELEASE STAMP 2012-11-16 (chr X)	build37	probABEL
SHIP	Affymetrix SNP 6.0	Birdseed2	>92%	>80%	>0.0001	none	duplicate SNP positions, sex-mismatch or duplicate individuals (by IBD)	905,910	IMPUTE v2.2.2	b37, 1000Gv3 (Mar 2012)	QUICKTEST v0.95
YFS	Illumina 670k custom	Illuminus	0.95	0.95	1.00E-06	0.01	heterozygosity, relatedness	546,674	SHAPEIT v1 and IMPUTE v2.2.2	Phase 1, release v3, March 2012 haplotypes	SNPTEST v.2.4.1

Study name	GWAS platform	Calling algorithm	Individual call rate filter (applied before imp'n)	SNP call rate filter (applied before imp'n)	SNP HWE *P* filter (applied before imp'n)	SNP MAF filter (applied before imp'n)	Other filters	No of SNPs after filtering (before imp'n)	Imputation software and version	NCBI; 1000 Genomes version for imp'n	Genotype-phenotype association software
**Stage 2**											
ECRHS	Human 610 Quad Chip (ILLUMINA)	BeadStudio	exclusion of males with high X heterozygosity	95%	0.0001	1%	sex discrepancies, IBS analysis for relatedness, ancestry analysis using PCA	567,512	IMPUTE2	version 3	SNPTEST v2
PIVUS	Illumina OmniExpress and Metabocgip	Bead Studio	≥0.95	≥0.95 (≥0.99 for MAF<0.05)	10-6	≥0.01		738,606	SHAPEIT and IMPUTE2	Mar-12	SNPTEST
TwinsUK	Illumina 300K Duo for and HumanHap610-Quad		Illumina GenCall	if MAF>5% then 0.95, else 0.99	E-06	1%		unavailable	Impute 2	Mar-12	Gemma
UK BiLEVE	Affymetrix Axiom UK BiLEVE array	Axiom® GT1 algorithm (Affymetrix Power Tools v1.15.1)	95%	None	1.00E-06	1%	Het. outliers, PCA outliers, sex mismatch, relateds (IBD)	unavailable (regional imputation undertaken)	SHAPEIT and IMPUTE2	Mar-12	SNPTEST

Supplementary Table 2 Follow-up results

a) Association results for 56 variants followed up in stage 2

Results for stage 1, stage 2 and the meta-analysis of both are presented for the 56 variants (55 from autosomal chromosomes and 1 from chromosome X) followed up in stage 2. P<$5x10^{-8}$ after meta-analysing stage 1 and stage 2 are highlighted in bold. rs189059189 and rs117068593 appear twice on the table because they were selected for follow-up for two different traits. Chr., chromosome; dist., distance.

rs number (chr: position)	Gene (function)	Measure	Coded allele/ Non coded allele	Stage 1			Stage 2			Stage 1 + stage 2		
				Beta	SE	P	Beta	SE	P	Beta	SE	P
rs10798992 (chr1:34268324)	*CSMD2* (intronic)	FEV_1/FVC	C/A	0.041	0.008	1.25E-07	0.002	0.006	7.14E-01	0.017	0.005	3.02E-04
rs4658231 (chr1:92041502)	*CDC7* (dist=50181),*TGFBR3* (dist=104398)	FVC	A/G	0.051	0.009	5.33E-09	0.011	0.007	1.20E-01	0.027	0.006	8.36E-07
rs2764553 (chr1:111732303)	*DENND2D* (intronic)	FEV_1/FVC	C/A	0.044	0.010	3.73E-06	0.020	0.008	7.27E-03	0.030	0.006	6.37E-07
rs4926386 (chr1:149886135)	*SV2A* (intronic)	FEV_1	C/A	-0.062	0.012	1.99E-07	-0.030	0.010	3.58E-03	-0.044	0.008	**2.09E-08**
rs6681426 (chr1:150586971)	*MCL1* (dist=34757), *ENSA* (dist=7628)	FEV_1	G/A	0.042	0.008	1.07E-07	0.021	0.006	1.13E-03	0.029	0.005	**4.35E-09**
rs201204531 (chr1:219963090)	*LYPLAL1* (dist=576883), *RNU5F-1* (dist=83529)	FEV_1/FVC	A/ATG	-0.038	0.008	1.98E-06	-0.027	0.006	1.77E-05	-0.031	0.005	**2.68E-10**
rs61067109 (chr2:18292452)	*KCNS3* (dist=178227), *NT5C1B-RDH14* (dist=443537)	FEV_1/FVC	G/A	-0.050	0.009	3.09E-08	-0.042	0.007	6.56E-09	-0.045	0.006	**1.40E-15**
rs1080871 (chr2:46854708)	*CRIPT* (UTR3)	FEV_1/FVC	A/G	0.035	0.008	4.79E-06	0.007	0.006	2.38E-01	0.018	0.005	1.56E-04
rs13386197 (chr2:47798588)	*KCNK12* (dist=1118), *MSH6* (dist=211633)	FVC	C/G	0.096	0.020	1.65E-06	0.011	0.014	4.44E-01	0.039	0.011	7.48E-04
rs185216854 (chr2:119211347)	*INSIG2* (dist=343750), *LOC101927709* (dist=146012)	FEV_1/FVC	G/T	0.180	0.037	1.31E-06	-0.029	0.030	3.38E-01	0.055	0.024	1.99E-02
rs74445319 (chr2:181515383)	*CWC22* (dist=643603), *SCHLAP1* (dist=41448)	FVC	C/T	0.102	0.022	4.68E-06	0.021	0.014	1.49E-01	0.044	0.012	2.23E-04
rs11432697 (chr3:89210365)	*EPHA3* (intronic)	FVC	T/TA	0.042	0.009	4.05E-06	0.018	0.007	1.75E-02	0.027	0.006	1.97E-06
rs10935473 (chr3:98416900)	*CPOX* (dist=104445), *ST3GAL6-AS1* (dist=16277)	FVC	G/T	-0.038	0.008	4.87E-06	-0.007	0.006	2.76E-01	-0.018	0.005	3.37E-04
rs7356112 (chr3:143970905)	*C3orf58* (dist=259695), *PLOD2* (dist=1816323)	FVC	C/T	0.104	0.021	6.05E-07	0.011	0.015	4.83E-01	0.044	0.012	4.24E-04
rs6441207 (chr3:158282459)	*RP11-538P18.2* (ncRNA_intronic)	FVC	C/T	0.036	0.008	4.54E-06	0.036	0.006	5.85E-09	0.036	0.005	**1.27E-13**
rs73223499 (chr4:14300501)	*LINC01085* (dist=158825), *CPEB2-AS1* (dist=611084)	FEV_1/FVC	C/T	-0.080	0.017	3.99E-06	-0.037	0.015	1.42E-02	-0.055	0.011	1.15E-06
rs35288667 (chr4:16544514)	*LDB2* (intronic)	FVC	A/G	0.049	0.010	1.55E-06	-0.005	0.008	5.01E-01	0.015	0.006	1.81E-02
rs6856422 (chr4:106841962)	*NPNT* (intronic)	FEV_1/FVC	G/T	-0.044	0.008	1.30E-07	-0.055	0.006	1.15E-17	-0.051	0.005	**1.51E-23**
rs11269742 (chr4:177758035)	*VEGFC* (dist=44136), *NEIL3* (dist=472956)	FVC	T/TGGGAAAAAAATAC	0.043	0.009	9.91E-07	0.000	0.007	9.78E-01	0.017	0.005	2.19E-03
rs112226333 (chr5:31525207)	*DROSHA* (intronic)	FEV_1	G/T	0.051	0.010	6.12E-07	0.016	0.008	4.42E-02	0.029	0.006	3.32E-06
rs11960365 (chr5:79650724)	*CRSP8P* (dist=2939), *ZFYVE16* (dist=53108)	FVC	G/T	0.050	0.010	2.15E-06	-0.008	0.008	3.30E-01	0.014	0.006	3.25E-02
rs149178928 (chr5:106109235)	*RAB9BP1* (dist=1673436), *LOC102467213* (dist=41663)	FEV_1	G/A	0.132	0.027	1.14E-06	0.018	0.021	3.82E-01	0.060	0.016	2.57E-04
rs114372586 (chr6:32886487)	*LOC100294145* (dist=14952), *HLA-DMB* (dist=15919)	FVC	T/C	0.081	0.017	2.83E-06	0.019	0.012	1.20E-01	0.040	0.010	7.30E-05

rs number (chr: position)	Gene (function)	Measure	Coded allele/ Non coded allele	Stage 1			Stage 2			Stage 1 + stage 2		
				Beta	SE	P	Beta	SE	P	Beta	SE	P
rs79787160 (chr6:72212515)	*LINC00472* (dist=82067), *RIMS1* (dist=383891)	FEV_1/FVC	A/G	0.241	0.050	1.09E-06	0.020	0.038	5.93E-01	0.103	0.030	6.71E-04
rs9352253 (chr6:76687328)	*IMPG1* (intronic)	FEV_1	A/C	0.041	0.009	1.51E-06	-0.012	0.007	7.25E-02	0.008	0.005	1.25E-01
rs148274477 (chr6:142838173)	GPR126(dist=70770),*RP11-440G9.1* (dist=9419)	FEV_1/FVC	C/T	-0.161	0.027	2.68E-09	-0.162	0.019	5.63E-18	-0.162	0.015	**9.58E-26**
rs6570551 (chr6:143556810)	*AIG1* (intronic)	FEV_1/FVC	A/G	-0.042	0.008	7.67E-08	-0.001	0.006	9.11E-01	-0.017	0.005	4.62E-04
rs4728570 (chr7:84728349)	*SEMA3D* (intronic)	FEV_1	G/A	-0.037	0.008	3.06E-06	-0.006	0.006	3.45E-01	-0.018	0.005	2.80E-04
rs141779646 (chr7:90805723)	*CDK14* (intronic)	FVC	G/C	0.087	0.019	3.80E-06	-0.016	0.016	3.23E-01	0.028	0.012	2.40E-02
rs112225874 (chr8:69228553)	*LOC286189* (ncRNA_intronic)	FVC	C/T	0.114	0.025	3.84E-06	0.032	0.024	1.72E-01	0.071	0.017	2.82E-05
rs76163270 (chr9:24678364)	*IZUMO3* (dist=132690), *TUSC1* (dist=998023)	FEV_1/FVC	A/C	-0.079	0.017	4.65E-06	-0.018	0.014	1.80E-01	-0.042	0.011	9.64E-05
rs34886460 (chr9:119359372)	*ASTN2* (intronic)	FEV_1/FVC	T/TA	0.041	0.008	6.75E-08	0.025	0.006	4.07E-05	0.031	0.005	**4.72E-11**
rs2274116 (chr9:139094805)	*LHX3* (exonic)	FVC	C/T	0.041	0.009	2.68E-06	0.038	0.007	4.20E-09	0.039	0.005	**5.55E-14**
rs10735064 (chr12:888848)	*WNK1* (intronic)	FVC	G/A	0.042	0.009	3.49E-06	0.014	0.007	5.41E-02	0.025	0.006	1.03E-05
rs12314259 (chr12:12272466)	*LRP6* (UTR3)	FVC	C/G	0.048	0.010	4.67E-06	-0.002	0.009	8.29E-01	0.018	0.007	6.46E-03
rs11383346 (chr12:28283187)	*PTHLH* (dist=158271), *CCDC91* (dist=126946)	FVC	A/AT	-0.043	0.008	3.83E-08	-0.041	0.006	4.42E-11	-0.042	0.005	**9.52E-18**
rs10850377 (chr12:115201436)	*TBX3* (dist=79467), *MED13L* (dist=1194945)	FEV_1	G/A	-0.047	0.008	6.68E-09	-0.028	0.006	1.55E-05	-0.035	0.005	**2.50E-12**
rs6563127 (chr13:80216147)	*NDFIP2* (dist=85935), *LINC01080* (dist=369065)	FVC	A/G	-0.059	0.013	2.91E-06	-0.011	0.011	3.38E-01	-0.031	0.008	1.46E-04
rs189059189 (chr14:34231304)	*NPAS3* (intronic)	FEV_1	C/T	-0.355	0.067	1.11E-07	-0.035	0.051	4.99E-01	-0.154	0.041	1.61E-04
rs189059189 (chr14:34231304)	*NPAS3* (intronic)	FVC	C/T	-0.318	0.067	2.19E-06	-0.041	0.051	4.23E-01	-0.143	0.041	4.46E-04
rs61980882 (chr14:75021464)	*LTBP2* (intronic)	FVC	C/T	-0.039	0.008	3.72E-07	-0.007	0.006	2.22E-01	-0.020	0.005	3.65E-05
rs7155279 (chr14:92485881)	*TRIP11* (intronic)	FEV_1	G/T	-0.041	0.008	1.39E-07	-0.022	0.006	4.17E-04	-0.030	0.005	**1.41E-09**
rs117068593 (chr14:93118229)	*RIN3* (exonic)	FEV_1	C/T	-0.048	0.010	2.72E-06	-0.027	0.008	5.68E-04	-0.035	0.006	**2.25E-08**
rs117068593 (chr14:93118229)	*RIN3* (exonic)	FVC	C/T	-0.054	0.010	1.74E-07	-0.022	0.008	5.83E-03	-0.033	0.006	8.10E-08
rs4906032 (chr14:101518910)	*MIR381HG*, *MIR487A* (downstream)	FVC	A/G	0.037	0.008	4.78E-06	0.004	0.007	5.75E-01	0.018	0.005	7.31E-04
rs8027068 (chr15:100339709)	*DNM1P46* (ncRNA_intronic)	FEV_1/FVC	G/A	0.043	0.009	3.79E-06	-0.005	0.007	5.05E-01	0.012	0.006	2.97E-02
rs113733630 (chr16:4343067)	*TFAP4* (dist=20066), *GLIS2* (dist=39158)	FEV_1/FVC	A/AGTTT	0.042	0.009	3.78E-06	0.015	0.007	4.03E-02	0.025	0.006	6.92E-06
rs12149828 (chr16:10706328)	*EMP2* (dist=31789), *TEKT5* (dist=15033)	FEV_1/FVC	G/A	0.049	0.010	3.16E-06	0.035	0.008	3.33E-05	0.040	0.007	**7.65E-10**
rs12929007 (chr16:30929696)	*FBXL19-AS1* (downstream)	FEV_1	C/G	-0.049	0.010	6.21E-07	0.001	0.007	8.47E-01	-0.016	0.006	5.95E-03
rs7187961 (chr16:53826034)	*FTO* (intronic)	FVC	T/C	0.048	0.010	3.43E-06	-0.008	0.007	2.80E-01	0.011	0.006	6.76E-02
rs16953104 (chr16:54165373)	*FTO* (dist=16994), *IRX3* (dist=151839)	FEV_1	C/T	0.276	0.057	1.09E-06	-0.007	0.064	9.10E-01	0.152	0.042	3.35E-04

rs number (chr: position)	Gene (function)	Measure	Coded allele/ Non coded allele	Stage 1			Stage 2			Stage 1 + stage 2		
				Beta	SE	P	Beta	SE	P	Beta	SE	P
rs4792721 (chr17:16006827)	*NCOR1* (intronic)	FEV_1	A/G	0.038	0.008	1.67E-06	0.017	0.006	8.06E-03	0.025	0.005	4.42E-07
rs58270905 (chr18:42844617)	*SLC14A2* (intronic)	FEV_1/FVC	A/G	0.252	0.055	4.51E-06	0.013	0.049	7.92E-01	0.118	0.036	1.20E-03
rs34000248 (chr18:67464012)	*DOK6* (intronic)	FEV_1	ACT/A	-0.070	0.014	5.75E-07	0.012	0.012	3.18E-01	-0.023	0.009	1.11E-02
rs113473882 (chr19:41124155)	*LTBP4* (intronic)	FEV_1/FVC	T/C	-0.174	0.033	1.48E-07	-0.138	0.028	1.00E-06	-0.153	0.021	**9.95E-13**
rs134041 (chr22:28056338)	*MIAT* (dist=983898), *MN1* (dist=87927)	FEV_1	T/C	-0.045	0.008	4.19E-09	-0.018	0.006	4.19E-03	-0.028	0.005	**3.03E-09**
rs79017381 (chr22:31496200)	*SMTN* (intronic)	FEV_1/FVC	C/T	0.120	0.026	4.41E-06	0.026	0.019	1.71E-01	0.059	0.015	1.36E-04
rs7050036 (chrX:15964845)	*AP1S2* (dist=91708), *GRPR* (dist=176579)	FEV_1/FVC	T/A	-0.041	0.007	4.37E-09	-0.013	0.005	1.60E-02	-0.023	0.004	4.14E-08

b) Association results for FEV_1, FEV_1/FVC and FVC for the 16 sentinel variants

Results for FEV_1, FEV_1/FVC and FVC in stage 1, stage 2 and the meta-analysis of both are presented for the 16 sentinel variants. $P<5x10^{-8}$ after meta-analysing stage 1 and stage 2 are highlighted in bold. Chr., chromosome; dist., distance.

rs number (chr: position)	Gene (function)	Measure	Coded allele/ Non coded allele	Stage 1			Stage 2			Stage 1 + stage 2		
				Beta	SE	P	Beta	SE	P	Beta	SE	P
rs6681426 (chr1:150586971)	*MCL1*(dist=34757), *ENSA*(dist=7628)	FEV_1	G/A	0.042	0.008	1.07E-07	0.021	0.006	1.13E-03	0.029	0.005	**4.35E-09**
		FEV_1/FVC	G/A	0.014	0.008	7.47E-02	-0.003	0.006	6.17E-01	0.004	0.005	4.65E-01
		FVC	G/A	0.036	0.008	4.74E-06	0.024	0.006	1.42E-04	0.029	0.005	**5.56E-09**
rs201204531 (chr1:219963090)	*LYPLAL1*(dist=576883), *RNU5F-1* (dist=83529)	FEV_1	A/ATG	-0.007	0.008	3.75E-01	-0.017	0.006	7.49E-03	-0.013	0.005	8.09E-03
		FEV_1/FVC	A/ATG	-0.038	0.008	1.98E-06	-0.027	0.006	1.77E-05	-0.031	0.005	**2.68E-10**
		FVC	A/ATG	0.011	0.008	1.53E-01	-0.008	0.006	2.18E-01	0.000	0.005	9.31E-01
rs61067109 (chr2:18292452)	*KCNS3*(dist=178227), *NT5C1B-RDH14* (dist=443537)	FEV_1	G/A	-0.039	0.009	1.83E-05	-0.036	0.007	3.93E-07	-0.037	0.006	**3.21E-11**
		FEV_1/FVC	G/A	-0.050	0.009	3.09E-08	-0.042	0.007	6.56E-09	-0.045	0.006	**1.40E-15**
		FVC	G/A	-0.009	0.009	3.05E-01	-0.026	0.007	3.07E-04	-0.020	0.006	5.22E-04
rs6441207 (chr3:158282459)	*AK097794* (ncRNA_intronic)	FEV_1	C/T	0.029	0.008	2.07E-04	0.033	0.006	1.34E-07	0.031	0.005	**1.23E-10**
		FEV_1/FVC	C/T	-0.003	0.008	6.56E-01	0.001	0.006	8.13E-01	0.000	0.005	9.25E-01
		FVC	C/T	0.036	0.008	4.54E-06	0.036	0.006	5.85E-09	0.036	0.005	**1.27E-13**
rs6856422 (chr4:106841962)	*NPNT* (intronic)	FEV_1	G/T	-0.045	0.008	6.22E-08	-0.037	0.006	1.26E-08	-0.040	0.005	**5.60E-15**
		FEV_1/FVC	G/T	-0.044	0.008	1.30E-07	-0.055	0.006	1.15E-17	-0.051	0.005	**1.51E-23**
		FVC	G/T	-0.020	0.008	1.54E-02	-0.019	0.006	3.97E-03	-0.019	0.005	1.70E-04
rs148274477 (chr6:142838173)	*GPR126*(dist=70770), *LOC153910*(dist=9419)	FEV_1	C/T	0.010	0.027	7.07E-01	-0.057	0.019	2.42E-03	-0.035	0.015	2.26E-02
		FEV_1/FVC	C/T	-0.161	0.027	2.68E-09	-0.162	0.019	5.63E-18	-0.162	0.015	**9.58E-26**
		FVC	C/T	0.114	0.027	3.07E-05	0.017	0.019	3.56E-01	0.048	0.015	1.77E-03
rs34886460 (chr9:119359372)	*ASTN2* (intronic)	FEV_1	T/TA	0.023	0.008	2.85E-03	0.015	0.006	1.13E-02	0.018	0.005	1.21E-04
		FEV_1/FVC	T/TA	0.041	0.008	6.75E-08	0.025	0.006	4.07E-05	0.031	0.005	**4.72E-11**
		FVC	T/TA	0.000	0.008	9.72E-01	0.007	0.006	2.33E-01	0.004	0.005	3.62E-01
rs2274116 (chr9:139094805)	*LHX3* (exonic)	FEV_1	C/T	0.029	0.009	6.27E-04	0.029	0.007	1.09E-05	0.029	0.005	**2.53E-08**
		FEV_1/FVC	C/T	-0.013	0.009	1.29E-01	0.001	0.007	9.00E-01	-0.004	0.005	4.13E-01
		FVC	C/T	0.041	0.009	2.68E-06	0.038	0.007	4.20E-09	0.039	0.005	**5.55E-14**
rs11383346 (chr12:28283187)	*PTHLH*(dist=158271), *CCDC91*(dist=126946)	FEV_1	A/AT	-0.039	0.008	3.41E-07	-0.034	0.006	5.37E-08	-0.036	0.005	**1.07E-13**
		FEV_1/FVC	A/AT	-0.002	0.008	8.38E-01	-0.004	0.006	5.31E-01	-0.003	0.005	5.37E-01
		FVC	A/AT	-0.043	0.008	3.83E-08	-0.041	0.006	4.42E-11	-0.042	0.005	**9.52E-18**
rs10850377 (chr12:115201436)	*TBX3*(dist=79467), *MED13L*(dist=1194945)	FEV_1	G/A	-0.047	0.008	6.68E-09	-0.028	0.006	1.55E-05	-0.035	0.005	**2.50E-12**
		FEV_1/FVC	G/A	-0.020	0.008	1.10E-02	-0.010	0.006	1.38E-01	-0.014	0.005	6.00E-03
		FVC	G/A	-0.033	0.008	3.60E-05	-0.027	0.006	2.67E-05	-0.030	0.005	**4.59E-09**

rs number (chr: position)	Gene (function)	Measure	Coded allele/ Non coded allele	Stage 1			Stage 2			Stage 1 + stage 2		
				Beta	SE	P	Beta	SE	P	Beta	SE	P
rs7155279 (chr14:92485881)	*TRIP11* (intronic)	FEV_1	G/T	-0.041	0.008	1.39E-07	-0.022	0.006	4.17E-04	-0.030	0.005	**1.41E-09**
		FEV_1/FVC	G/T	-0.023	0.008	3.49E-03	-0.015	0.006	2.10E-02	-0.018	0.005	2.83E-04
		FVC	G/T	-0.031	0.008	8.62E-05	-0.020	0.006	1.50E-03	-0.024	0.005	8.06E-07
rs117068593 (chr14:93118229)	*RIN3* (exonic)	FEV_1	C/T	-0.048	0.010	2.72E-06	-0.027	0.008	5.68E-04	-0.035	0.006	**2.25E-08**
		FEV_1/FVC	C/T	0.008	0.010	4.23E-01	-0.018	0.008	2.10E-02	-0.008	0.006	1.79E-01
		FVC	C/T	-0.054	0.010	1.74E-07	-0.022	0.008	5.83E-03	-0.033	0.006	8.10E-08
rs12149828 (chr16:10706328)	*EMP2*(dist=31789), *TEKT5*(dist=15033)	FEV_1	G/A	0.037	0.010	3.38E-04	0.022	0.008	7.31E-03	0.028	0.007	1.45E-05
		FEV_1/FVC	G/A	0.049	0.010	3.16E-06	0.035	0.008	3.33E-05	0.040	0.007	**7.65E-10**
		FVC	G/A	0.005	0.010	6.12E-01	0.007	0.008	3.96E-01	0.006	0.007	3.27E-01
rs113473882 (chr19:41124155)	*LTBP4* (intronic)	FEV_1	T/C	-0.029	0.033	3.78E-01	-0.019	0.028	5.00E-01	-0.023	0.021	2.78E-01
		FEV_1/FVC	T/C	-0.174	0.033	1.48E-07	-0.138	0.028	1.00E-06	-0.153	0.021	**9.95E-13**
		FVC	T/C	0.073	0.033	2.88E-02	0.046	0.028	1.05E-01	0.057	0.022	8.02E-03
rs134041 (chr22:28056338)	*MIAT*(dist=983898), *MN1*(dist=87927)	FEV_1	T/C	-0.045	0.008	4.19E-09	-0.018	0.006	4.19E-03	-0.028	0.005	**3.03E-09**
		FEV_1/FVC	T/C	-0.025	0.008	1.06E-03	-0.016	0.006	1.11E-02	-0.019	0.005	5.45E-05
		FVC	T/C	-0.031	0.008	5.44E-05	-0.017	0.006	7.20E-03	-0.022	0.005	3.69E-06
rs7050036 (chrX:15964845)	*AP1S2*(dist=91708), *GRPR*(dist=176579)	FEV_1	T/A	-0.014	0.007	4.41E-02	-0.003	0.005	6.03E-01	-0.007	0.004	1.06E-01
		FEV_1/FVC	T/A	-0.041	0.007	4.37E-09	-0.013	0.005	1.60E-02	-0.023	0.004	**4.14E-08**
		FVC	T/A	0.010	0.007	1.47E-01	0.005	0.005	3.36E-01	0.007	0.004	1.01E-01

Supplementary Table 3 Functional characterization of novel lung function loci

a) eQTL results

Results from eQTL analyses in blood[6] and lung[7-9] (details in **Supplementary Methods**) are presented here for variants in 2Mb regions (sentinel +/- 1Mb) in LD ($r^2 > 0.3$) with the sentinel variant for all the 16 new sentinel variants, which (i) met an FDR threshold of 10%, and (ii) had eQTL $P < 5 \times 10^{-5}$. For each probeset we ranked variants according to their correlation with the sentinel SNP (as measured by r^2) first and to their eQTL *P* value second, and we only present results for the top ranked variant for each probeset. Datasets were queried both for *cis* and *trans* eQTLs, but no *trans* results were significant, therefore the table only presents *cis* results. “Effect on expression” indicates whether the “Effect allele” was associated with increased gene expression (“+”) or with decreased gene expression (“-“). In the lung eQTL analyses for the autosomal chromosomes results from three different studies were meta-analysed, as indicated by “Dataset” on the table, however for X chromosome analyses were undertaken separately for males and females in each study (details are given in the “Dataset” column). No blood eQTL results were available for the X chromosome. This table presents results for an additional SNP (rs4926386) next to the *ENSA* region. Chr., chromosome.

Sentinel rs number (chr: position)	Proxy rs number (chr: position)	R^2 with sentinel	eQTL *P*	Probeset id	Gene Symbol	Effect allele	Effect on expression	Tissue	Dataset
rs4926386 (chr1:149886135)	rs4926386 (chr1:149886135)	1.00	7.72E-06	100138305_TGI_at	*ARNT*	C	-	lung	meta-analysed
	rs3754047 (chr1:150039207)	0.60	1.51E-13	3890500	*MRPS21*	A	-	blood	-
	rs3738320 (chr1:149982626)	0.38	7.57E-06	100309612_TGI_at	*SV2A*	A	+	lung	meta-analysed
	rs4926399 (chr1:149924299)	0.35	1.82E-05	100123438_TGI_at	*MTMR11*	C	-	lung	meta-analysed
rs6681426 (chr1:150586971)	rs6681426 (chr1:150586971)	1.00	2.10E-68	100138305_TGI_at	*ARNT*	A	-	lung	meta-analysed
	rs6681426 (chr1:150586971)	1.00	5.21E-15	100302368_TGI_at	*LASS2*	A	-	lung	meta-analysed
	rs6681426 (chr1:150586971)	1.00	1.44E-13	100135055_TGI_at	*HORMAD1*	A	-	lung	meta-analysed
	rs6681426 (chr1:150586971)	1.00	1.66E-12	100151271_TGI_at	*LASS2*	A	-	lung	meta-analysed
	rs6681426 (chr1:150586971)	1.00	1.85E-12	100309723_TGI_at	*ARNT*	A	+	lung	meta-analysed
	rs6681426 (chr1:150586971)	1.00	8.55E-11	100308717_TGI_at	*GOLPH3L*	A	-	lung	meta-analysed
	rs6681426 (chr1:150586971)	1.00	6.09E-10	100144443_TGI_at	cDNA clone that overlaps *ADAMTSL4*	A	+	lung	meta-analysed
	rs6681426 (chr1:150586971)	1.00	1.09E-07	100306322_TGI_at	*ARNT*	A	+	lung	meta-analysed
	rs6681426 (chr1:150586971)	1.00	8.75E-06	100307575_TGI_at	*MRPS21*	A	+	lung	meta-analysed
	rs6681426 (chr1:150586971)	1.00	1.49E-05	100313509_TGI_at	*FAM63A*	A	+	lung	meta-analysed
	rs2280078 (chr1:150600181)	0.99	1.08E-120	6330484	*CTSS*	C	+	blood	-
	rs2280078 (chr1:150600181)	0.99	1.13E-20	1430278	*CTSK*	C	+	blood	-
	rs2280078 (chr1:150600181)	0.99	1.94E-05	2190040	*ARNT*	C	-	blood	-
	rs4970969 (chr1:150612361)	0.91	1.58E-05	100310890_TGI_at	*ARNT*	T	-	lung	meta-analysed
	rs11204718 (chr1:150709785)	0.84	4.12E-08	100159164_TGI_at	*GOLPH3L*	C	-	lung	meta-analysed
	rs11204718 (chr1:150709785)	0.84	3.00E-07	100143466_TGI_at	*GOLPH3L*	C	-	lung	meta-analysed
	rs7521898 (chr1:150707596)	0.80	2.22E-19	3370121	*LASS2*	A	+	blood	-
	rs7521898 (chr1:150707596)	0.80	2.52E-17	6380154	*LASS2*	A	+	blood	-

Sentinel rs number (chr: position)	Proxy rs number (chr: position)	R^2 with sentinel	eQTL *P*	Probeset id	Gene Symbol	Effect allele	Effect on expression	Tissue	Dataset
	rs11204691 (chr1:150644339)	0.76	4.27E-05	2640338	*ARNT*	T	+	blood	-
	rs878471 (chr1:150547747)	0.73	4.55E-08	100144721_TGI_at	*RPRD2*	G	-	lung	meta-analysed
	rs878471 (chr1:150547747)	0.73	6.87E-06	100161206_TGI_at	*ENSA*	G	-	lung	meta-analysed
	rs897815 (chr1:150879101)	0.64	4.89E-11	4480192	*CDC42SE1, C1orf56*	G	-	blood	-
	rs897815 (chr1:150879101)	0.64	3.99E-07	4390372	*BNIPL*	G	-	blood	-
	rs897815 (chr1:150879101)	0.64	5.60E-07	4540288	*CDC42SE1, C1orf56*	G	-	blood	-
	rs3768013 (chr1:150815411)	0.63	3.14E-05	6450722	*SETDB1*	A	-	blood	-
	rs11204693 (chr1:150647987)	0.55	1.20E-05	7380100	*MCL1*	T	-	blood	-
	rs11204664 (chr1:150531380)	0.48	2.47E-08	100156777_TGI_at	*MRPS21*	C	+	lung	meta-analysed
	rs11204664 (chr1:150531380)	0.48	1.50E-06	100130530_TGI_at	*ECM1*	C	+	lung	meta-analysed
	rs2305814 (chr1:150958977)	0.45	2.99E-05	4280162	*SCNM1*	C	-	blood	-
	rs486836 (chr1:150875967)	0.40	1.78E-05	100125530_TGI_at	*CTSS*	A	+	lung	meta-analysed
rs201204531 (chr1:219963090)	rs1338230 (chr1:219963090)	0.31	1.27E-06	100159938_TGI_at	*SLC30A10*	G	+	lung	meta-analysed
rs6441207 (chr3:158282459)	rs6441207 (chr3:158282459)	1.00	2.30E-08	100301782_TGI_at	*MLF1*	C	-	lung	meta-analysed
	rs6441207 (chr3:158282459)	1.00	3.21E-06	100304858_TGI_at	*MLF1*	C	-	lung	meta-analysed
	rs112353818 (chr3:158239479)	0.82	2.63E-09	100150755_TGI_at	*GFM1*	G	+	lung	meta-analysed
	rs112353818 (chr3:158239479)	0.82	2.90E-06	100123606_TGI_at	*MLF1*	G	-	lung	meta-analysed
	rs7620927 (chr3:158245883)	0.81	1.22E-05	3440195	*GFM1*	G	+	blood	-
	rs12492884 (chr3:158252566)	0.53	2.62E-06	60670	*GFM1,LXN*	G	-	blood	-
	rs1656377 (chr3:158285280)	0.50	2.32E-06	100312821_TGI_at	*MLF1*	C	+	lung	meta-analysed
	rs2731122 (chr3:158295280)	0.37	2.62E-06	100141104_TGI_at	*GFM1*	G	-	lung	meta-analysed
	rs63740961 (chr3:158295171)	0.31	8.55E-06	100301664_TGI_at	*GFM1*	C	-	lung	meta-analysed

Sentinel rs number (chr: position)	Proxy rs number (chr: position)	R^2 with sentinel	eQTL *P*	Probeset id	Gene Symbol	Effect allele	Effect on expression	Tissue	Dataset
rs6856422 (chr4:106841962)	rs4600917 (chr4:106829645)	0.77	2.89E-06	4860553	*INTS12*	C	-	blood	-
	rs2553449 (chr4:106698892)	0.42	3.17E-05	5360487	*GSTCD*	T	+	blood	-
rs34886460 (chr9:119359372)	rs1372335 (chr9:119403743)	0.62	1.10E-06	5670088	*TRIM32*	T	+	blood	-
rs2274116 (chr9:139094805)	rs2274116 (chr9:139094805)	1.00	1.81E-06	100124395_TGI_at	*QSOX2*	C	+	lung	meta-analysed
	rs10858246 (chr9:139102831)	0.88	4.78E-08	3830605	*C9orf151*	C	+	blood	-
	rs3780190 (chr9:139099073)	0.43	1.32E-05	3060494	*CARD9*	G	-	blood	-
rs11383346 (chr12:28283187)	rs11383346 (chr12:28283187)	1.00	1.73E-13	100137632_TGI_at	*cDNA clone that maps to CCDC91*	NA	-	lung	meta-analysed
	rs11049391 (chr12:28322273)	0.90	1.55E-05	100124630_TGI_at	*CCDC91*	G	+	lung	meta-analysed
	rs10843114 (chr12:28303296)	0.35	6.29E-06	100133373_TGI_at	*CCDC91*	C	-	lung	meta-analysed
rs7155279 (chr14:92485881)	rs7155279 (chr14:92485881)	1.00	1.01E-10	100132576_TGI_at	*ATXN3*	G	-	lung	meta-analysed
	rs7155279 (chr14:92485881)	1.00	3.38E-06	100160661_TGI_at	*TRIP11*	G	-	lung	meta-analysed
	rs76497846 (chr14:92531888)	0.99	1.80E-05	100127017_TGI_at	*CPSF2*	G	-	lung	meta-analysed
	rs7157056 (chr14:92498033)	0.94	3.00E-34	4670441	*FBLN5*	G	+	blood	-
	rs7157056 (chr14:92498033)	0.94	1.23E-29	5690639	*FBLN5*	G	+	blood	-
	rs7157056 (chr14:92498033)	0.94	1.12E-19	2760136	*TRIP11*	G	+	blood	-
	rs10151561 (chr14:92430184)	0.75	2.05E-06	100300526_TGI_at	*ATXN3*	A	-	lung	meta-analysed
	rs10129429 (chr14:92430831)	0.75	1.72E-05	100143127_TGI_at	*NDUFB1*	G	+	lung	meta-analysed
	rs7145052 (chr14:92461192)	0.69	3.35E-05	5090037	*NDUFB1*	T	-	blood	-
	rs111269625 (chr14:92514370)	0.56	2.50E-13	100148617_TGI_at	*ATXN3*	G	+	lung	meta-analysed
	rs111269625 (chr14:92514370)	0.56	1.13E-08	100306579_TGI_at	*ATXN3*	G	+	lung	meta-analysed
	rs111269625 (chr14:92514370)	0.56	1.60E-05	100137755_TGI_at	*ATXN3*	G	-	lung	meta-analysed

**Sentinel rs number (chr: position)**	**Proxy rs number (chr: position)**	**R^2 with sentinel**	**eQTL *P***	**Probeset id**	**Gene Symbol**	**Effect allele**	**Effect on expression**	**Tissue**	**Dataset**
rs12149828 (chr16:10706328)	rs8053116 (chr16:10719601)	0.55	5.51E-06	100123005_TGI_at	*EMP2*	A	+	lung	meta-analysed
rs113473882 (chr19:41124155)	rs113434779 (chr19:41236459)	0.49	1.15E-05	100302701_TGI_at	*NUMBL*	C	-	lung	meta-analysed
rs7050036 (chrX:15964845)	rs798183 (chrX:15840426)	0.62	1.96E-08	100302651_TGI_at	*ZRSR2*	T	+	lung	UBC_male
	rs798183 (chrX:15840426)	0.62	3.18E-07	100302651_TGI_at	*ZRSR2*	T	+	lung	Laval_male
	rs798183 (chrX:15840426)	0.62	1.34E-06	100302651_TGI_at	*ZRSR2*	T	+	lung	Groningen_male
	rs798183 (chrX:15840426)	0.62	7.79E-06	100302651_TGI_at	*ZRSR2*	T	+	lung	UBC_female
	rs798157 (chrX:15810140)	0.53	2.99E-09	100311509_TGI_at	*AP1S2*	T	+	lung	Laval_male
	rs798157 (chrX:15810140)	0.53	5.49E-08	100122762_TGI_at	*AP1S2*	T	+	lung	Laval_male

b) DNase hypersensitivity sites

DNase hypersensitivity sites as defined by ENCODE[10], which contain at least one variant strongly correlated ($r^2 > 0.8$) with any of the new 16 sentinel variants are presented here. The “Signal Value” is a measurement of average enrichment in the region and the “Score value” is a normalized value that ranges from 0 to 1,000 based on the signal value[10,11]. Only DNase hypersensitivity sites with “Score value” > 100 are presented. Cell type abbreviations are: A549, epithelial cell line derived from a lung carcinoma tissue; AG04450, fetal lung fibroblast; HMVEC-LBl, blood microvascular endothelial cells, lung-derived; HMVEC-LLy, lymphatic microvascular endothelial cells, lung-derived; HPF, pulmonary fibroblasts isolated from lung tissue; HPAEC, pulmonary artery endothelial cells; HPAF, pulmonary artery fibroblasts; NHLF, lung fibroblasts; WI-38, embryonic lung fibroblast cells, hTERT immortalized, includes Raf1 construct. Chr., chromosome.

Sentinel rs number (chr: position)	Proxy rs number (chr: position)	R^2 with sentinel	Cell type	Chr.	Start	End	Score value	Signal value
rs6681426 (chr1:150586971)	rs6655975 (chr1:150542128)	0.97	HPAEC	1	150542080	150542230	102	15
rs6681426 (chr1:150586971)	rs6655975 (chr1:150542128)	0.97	HPAEC	1	150542080	150542230	102	15
rs201204531 (chr1:219963090)	rs10863500 (chr1:219961690)	1.00	AG04450	1	219961560	219961710	101	24
rs2274116 (chr9:119359372)	rs4836757 (chr9:119326613)	0.98	A549	9	119326565	119326715	100	21
rs11383346 (chr12:28283187)	rs2881788 (chr12:28272574)	0.82	WI-38	12	28272520	28272670	100	10
rs11383346 (chr12:28283187)	rs7310453 (chr12:28283780)	0.99	AG04450	12	28283700	28283850	106	173
rs11383346 (chr12:28283187)	rs7310453 (chr12:28283780)	0.99	HPAF	12	28283700	28283850	129	1340
rs11383346 (chr12:28283187)	rs7310453 (chr12:28283780)	0.99	HPAEC	12	28283720	28283870	137	272
rs11383346 (chr12:28283187)	rs7310453 (chr12:28283780)	0.99	HMVEC-LLy	12	28283740	28283890	110	739
rs11383346 (chr12:28283187)	rs7310453 (chr12:28283780)	0.99	HPF	12	28283740	28283890	115	253
rs11383346 (chr12:28283187)	rs7310453 (chr12:28283780)	0.99	WI-38	12	28283740	28283890	116	297
rs11383346 (chr12:28283187)	rs7310453 (chr12:28283780)	0.99	NHLF	12	28283740	28283890	121	948
rs11383346 (chr12:28283187)	rs7310453 (chr12:28283780)	0.99	A549	12	28283760	28283910	100	21
rs11383346 (chr12:28283187)	rs7310453 (chr12:28283780)	0.99	HMVEC-LBl	12	28283760	28283910	182	1082
rs11383346 (chr12:28283187)	rs7310453 (chr12:28285003)	0.99	HMVEC-LBl	12	28285000	28285150	101	16
rs11383346 (chr12:28283187)	rs7310453 (chr12:28406157)	0.82	HPAF	12	28406080	28406230	100	19
rs11383346 (chr12:28283187)	rs7310453 (chr12:28406157)	0.82	NHLF	12	28406080	28406230	102	84
rs10850377 (chr12:115201436)	rs78649052 (chr12:115200597)	0.97	HPAF	12	115200500	115200650	102	77
rs10850377 (chr12:115201436)	rs78649052 (chr12:115200597)	0.97	AG04450	12	115200500	115200650	103	81
rs10850377 (chr12:115201436)	rs78649052 (chr12:115200597)	0.97	HPF	12	115200500	115200650	109	148
rs10850377 (chr12:115201436)	rs78649052 (chr12:115200597)	0.97	WI-38	12	115200500	115200650	109	172
rs117068593 (chr14:93118229)	rs78440108 (chr14:93070286)	0.90	AG04450	14	93070260	93070410	103	74
rs117068593 (chr14:93118229)	rs78440108 (chr14:93070286)	0.90	AG04450	14	93070260	93070410	103	74
rs117068593 (chr14:93118229)	rs78440108 (chr14:93070286)	0.90	WI-38	14	93070260	93070410	103	57
rs117068593 (chr14:93118229)	rs78440108 (chr14:93070286)	0.90	WI-38	14	93070260	93070410	103	57
rs117068593 (chr14:93118229)	rs78440108 (chr14:93070286)	0.90	AG04450	14	93070260	93070410	103	74
rs117068593 (chr14:93118229)	rs78440108 (chr14:93070286)	0.90	AG04450	14	93070260	93070410	103	74
rs117068593 (chr14:93118229)	rs78440108 (chr14:93070286)	0.90	WI-38	14	93070260	93070410	103	57
rs117068593 (chr14:93118229)	rs78440108 (chr14:93070286)	0.90	WI-38	14	93070260	93070410	103	57
rs117068593 (chr14:93118229)	rs78440108 (chr14:93070286)	0.90	HPAF	14	93070260	93070410	104	164
rs117068593 (chr14:93118229)	rs78440108 (chr14:93070286)	0.90	HPAF	14	93070260	93070410	104	164
rs117068593 (chr14:93118229)	rs78440108 (chr14:93070286)	0.90	HPF	14	93070260	93070410	104	66
rs117068593 (chr14:93118229)	rs78440108 (chr14:93070286)	0.90	HPF	14	93070260	93070410	104	66
rs117068593 (chr14:93118229)	rs78440108 (chr14:93070286)	0.90	NHLF	14	93070260	93070410	104	191

Sentinel rs number (chr: position)	Proxy rs number (chr: position)	R^2 with sentinel	Cell type	Chr.	Start	End	Score value	Signal value
rs117068593 (chr14:93118229)	rs78440108 (chr14:93070286)	0.90	NHLF	14	93070260	93070410	104	191
rs117068593 (chr14:93118229)	rs78440108 (chr14:93070286)	0.90	HPAF	14	93070260	93070410	104	164
rs117068593 (chr14:93118229)	rs78440108 (chr14:93070286)	0.90	HPAF	14	93070260	93070410	104	164
rs117068593 (chr14:93118229)	rs78440108 (chr14:93070286)	0.90	HPF	14	93070260	93070410	104	66
rs117068593 (chr14:93118229)	rs78440108 (chr14:93070286)	0.90	HPF	14	93070260	93070410	104	66
rs117068593 (chr14:93118229)	rs78440108 (chr14:93070286)	0.90	NHLF	14	93070260	93070410	104	191
rs117068593 (chr14:93118229)	rs78440108 (chr14:93070286)	0.90	NHLF	14	93070260	93070410	104	191
rs117068593 (chr14:93118229)	rs72697299 (chr14:93070367)	0.90	AG04450	14	93070260	93070410	103	74
rs117068593 (chr14:93118229)	rs72697299 (chr14:93070367)	0.90	AG04450	14	93070260	93070410	103	74
rs117068593 (chr14:93118229)	rs72697299 (chr14:93070367)	0.90	WI-38	14	93070260	93070410	103	57
rs117068593 (chr14:93118229)	rs72697299 (chr14:93070367)	0.90	WI-38	14	93070260	93070410	103	57
rs117068593 (chr14:93118229)	rs72697299 (chr14:93070367)	0.90	AG04450	14	93070260	93070410	103	74
rs117068593 (chr14:93118229)	rs72697299 (chr14:93070367)	0.90	AG04450	14	93070260	93070410	103	74
rs117068593 (chr14:93118229)	rs72697299 (chr14:93070367)	0.90	WI-38	14	93070260	93070410	103	57
rs117068593 (chr14:93118229)	rs72697299 (chr14:93070367)	0.90	WI-38	14	93070260	93070410	103	57
rs117068593 (chr14:93118229)	rs72697299 (chr14:93070367)	0.90	HPAF	14	93070260	93070410	104	164
rs117068593 (chr14:93118229)	rs72697299 (chr14:93070367)	0.90	HPAF	14	93070260	93070410	104	164
rs117068593 (chr14:93118229)	rs72697299 (chr14:93070367)	0.90	HPF	14	93070260	93070410	104	66
rs117068593 (chr14:93118229)	rs72697299 (chr14:93070367)	0.90	HPF	14	93070260	93070410	104	66
rs117068593 (chr14:93118229)	rs72697299 (chr14:93070367)	0.90	NHLF	14	93070260	93070410	104	191
rs117068593 (chr14:93118229)	rs72697299 (chr14:93070367)	0.90	NHLF	14	93070260	93070410	104	191
rs117068593 (chr14:93118229)	rs72697299 (chr14:93070367)	0.90	HPAF	14	93070260	93070410	104	164
rs117068593 (chr14:93118229)	rs72697299 (chr14:93070367)	0.90	HPAF	14	93070260	93070410	104	164
rs117068593 (chr14:93118229)	rs72697299 (chr14:93070367)	0.90	HPF	14	93070260	93070410	104	66
rs117068593 (chr14:93118229)	rs72697299 (chr14:93070367)	0.90	HPF	14	93070260	93070410	104	66
rs117068593 (chr14:93118229)	rs72697299 (chr14:93070367)	0.90	NHLF	14	93070260	93070410	104	191
rs117068593 (chr14:93118229)	rs72697299 (chr14:93070367)	0.90	NHLF	14	93070260	93070410	104	191
rs117068593 (chr14:93118229)	rs78440108 (chr14:93070286)	0.90	HPAEC	14	93070280	93070430	103	21
rs117068593 (chr14:93118229)	rs78440108 (chr14:93070286)	0.90	HPAEC	14	93070280	93070430	103	21
rs117068593 (chr14:93118229)	rs78440108 (chr14:93070286)	0.90	HPAEC	14	93070280	93070430	103	21
rs117068593 (chr14:93118229)	rs78440108 (chr14:93070286)	0.90	HPAEC	14	93070280	93070430	103	21
rs117068593 (chr14:93118229)	rs72697299 (chr14:93070367)	0.90	HPAEC	14	93070280	93070430	103	21
rs117068593 (chr14:93118229)	rs72697299 (chr14:93070367)	0.90	HPAEC	14	93070280	93070430	103	21
rs117068593 (chr14:93118229)	rs72697299 (chr14:93070367)	0.90	HPAEC	14	93070280	93070430	103	21

Sentinel rs number (chr: position)	Proxy rs number (chr: position)	R^2 with sentinel	Cell type	Chr.	Start	End	Score value	Signal value
rs117068593 (chr14:93118229)	rs72697299 (chr14:93070367)	0.90	HPAEC	14	93070280	93070430	103	21
rs117068593 (chr14:93118229)	rs72697299 (chr14:93070367)	0.90	HMVEC-LLy	14	93070300	93070450	100	19
rs117068593 (chr14:93118229)	rs72697299 (chr14:93070367)	0.90	HMVEC-LLy	14	93070300	93070450	100	19
rs117068593 (chr14:93118229)	rs72697299 (chr14:93070367)	0.90	HMVEC-LLy	14	93070300	93070450	100	19
rs117068593 (chr14:93118229)	rs72697299 (chr14:93070367)	0.90	HMVEC-LLy	14	93070300	93070450	100	19
rs117068593 (chr14:93118229)	rs7143806 (chr14:93103729)	0.94	WI-38	14	93103660	93103810	102	44
rs117068593 (chr14:93118229)	rs7143806 (chr14:93103729)	0.94	WI-38	14	93103660	93103810	102	44
rs117068593 (chr14:93118229)	rs7143806 (chr14:93103729)	0.94	WI-38	14	93103660	93103810	102	44
rs117068593 (chr14:93118229)	rs7143806 (chr14:93103729)	0.94	WI-38	14	93103660	93103810	102	44
rs117068593 (chr14:93118229)	rs7143806 (chr14:93103729)	0.94	HPF	14	93103660	93103810	103	48
rs117068593 (chr14:93118229)	rs7143806 (chr14:93103729)	0.94	HPF	14	93103660	93103810	103	48
rs117068593 (chr14:93118229)	rs7143806 (chr14:93103729)	0.94	HPF	14	93103660	93103810	103	48
rs117068593 (chr14:93118229)	rs7143806 (chr14:93103729)	0.94	HPF	14	93103660	93103810	103	48
rs117068593 (chr14:93118229)	rs7143806 (chr14:93103729)	0.94	AG04450	14	93103680	93103830	101	29
rs117068593 (chr14:93118229)	rs7143806 (chr14:93103729)	0.94	AG04450	14	93103680	93103830	101	29
rs117068593 (chr14:93118229)	rs7143806 (chr14:93103729)	0.94	AG04450	14	93103680	93103830	101	29
rs117068593 (chr14:93118229)	rs7143806 (chr14:93103729)	0.94	AG04450	14	93103680	93103830	101	29
rs117068593 (chr14:93118229)	rs7143806 (chr14:93103729)	0.94	HPAF	14	93103720	93103870	100	23
rs117068593 (chr14:93118229)	rs7143806 (chr14:93103729)	0.94	HPAF	14	93103720	93103870	100	23
rs117068593 (chr14:93118229)	rs7143806 (chr14:93103729)	0.94	HPAF	14	93103720	93103870	100	23
rs117068593 (chr14:93118229)	rs7143806 (chr14:93103729)	0.94	HPAF	14	93103720	93103870	100	23
rs117068593 (chr14:93118229)	rs201493181 (chr14:93112309)	0.99	HPAF	14	93112300	93112450	102	85
rs117068593 (chr14:93118229)	rs201493181 (chr14:93112309)	0.99	HPAF	14	93112300	93112450	102	85
rs117068593 (chr14:93118229)	rs201493181 (chr14:93112309)	0.99	HPAF	14	93112300	93112450	102	85
rs117068593 (chr14:93118229)	rs201493181 (chr14:93112309)	0.99	HPAF	14	93112300	93112450	102	85
rs12149828 (chr16:10706328)	rs12596425 (chr16:10713425)	0.95	NHLF	16	10713420	10713570	100	12
rs134041 (chr22:28056338)	rs134077 (chr22:28063227)	0.97	HPAF	22	28063120	28063270	101	64
rs134041 (chr22:28056338)	rs134117 (chr22:28082715)	0.86	HPAEC	22	28082620	28082770	106	47
rs134041 (chr22:28056338)	rs134117 (chr22:28082715)	0.86	HMVEC-LLy	22	28082640	28082790	100	21
rs134041 (chr22:28056338)	rs134117 (chr22:28082715)	0.86	AG04450	22	28082640	28082790	101	28
rs134041 (chr22:28056338)	rs134117 (chr22:28082715)	0.86	HPF	22	28082640	28082790	103	59
rs134041 (chr22:28056338)	rs134117 (chr22:28082715)	0.86	WI-38	22	28082640	28082790	103	57
rs134041 (chr22:28056338)	rs134117 (chr22:28082715)	0.86	NHLF	22	28082640	28082790	104	190
rs134041 (chr22:28056338)	rs134117 (chr22:28082715)	0.86	HPAF	22	28082640	28082790	109	411

Sentinel rs number (chr: position)	Proxy rs number (chr: position)	R^2 with sentinel	Cell type	Chr.	Start	End	Score value	Signal value
rs134041 (chr22:28056338)	rs134117 (chr22:28082715)	0.86	HMVEC-LBI	22	28082640	28082790	117	228
rs134041 (chr22:28056338)	rs134117 (chr22:28082715)	0.86	A549	22	28082680	28082830	100	45

c) Human Protein Atlas results

Protein expression results in the respiratory system for epithelial cells, pneumocytes and macrophages for a subset of genes for each of the regions that contain the 16 sentinel variants (see gene selection criteria in **Supplementary Methods**) were assessed in the Human Protein Atlas[12] and are presented here. If no results were available in the Human Protein Atlas a hyphen (“-“) is presented on the table. Chr., chromosome; H, high; M, medium; L, low; ND not detected.

rs number (chr: position)	Measure	Gene Symbol	Respiratory epithelial cells, Nasopharynx (antibody1 staining, antibody2 staining, annotated protein expression)	Respiratory epithelial cells, Bronchus(antibody1 staining, antibody2 staining, annotated protein expression)	Pneumocytes, lung (antibody1 staining, antibody2 staining, annotated protein expression)	Macrophages, lung(antibody1 staining, antibody2 staining, annotated protein expression)
rs6681426 (chr1:150586971)	FEV_1	*ARNT*	M,M,M	M,L,L	M,M,M	M,M,M
rs6681426 (chr1:150586971)	FEV_1	*CTSS*	M,L,M	M,M,M	ND,ND,ND	H,H,H
rs6681426 (chr1:150586971)	FEV_1	*LASS2*	L,-,-	M,-,-	H,-,-	ND,-,-
rs201204531 (chr1:219963090)	FF	*SLC30A10*	ND,-,-	ND,-,-	L,-,-	M,-,-
rs61067109 (chr2:18292452)	FF	*KCNS3*	M,-,-	-,-,-	L,-,-	M,-,-
rs6441207 (chr3:158282459)	FVC	*GFM1*	M,-,-	H,-,-	ND,-,-	H,-,-
rs6441207 (chr3:158282459)	FVC	*MLF1*	H,-,-	M,-,-	ND,-,-	H,-,-
rs6856422 (chr4:106841962)	FF	*NPNT*	ND,-,-	ND,-,-	H,-,-	ND,-,-
rs6856422 (chr4:106841962)	FF	*INTS12*	H,-,-	M,-,-	M,-,-	M,-,-
rs6856422 (chr4:106841962)	FF	*GSTCD*	M,-,-	M,-,-	ND,-,-	M,-,-
rs148274477 (chr6:142838173)	FF	*GPR126*	ND,-,-	ND,-,-	ND,-,-	ND,-,-
rs34886460 (chr9:119359372)	FF	*TRIM32*	M,-,-	M,-,-	ND,-,-	ND,-,-
rs2274116 (chr9:139094805)	FVC	*QSOX2*	M,-,-	H,-,-	ND,-,-	L,-,-
rs11383346 (chr12:28283187)	FVC	*CCDC91*	H,-,-	H,-,-	M,-,-	M,-,-
rs10850377 (chr12:115201436)	FEV_1	*TBX3*	L,-,-	L,-,-	ND,-,-	H,-,-
rs7155279 (chr14:92485881)	FEV_1	*TRIP11*	H,-,-	H,-,-	H,-,-	H,-,-
rs7155279 (chr14:92485881)	FEV_1	*ATXN3*	M,H,H	ND,H,H	ND,H,H	ND,H,H
rs117068593 (chr14:93118229)	FEV_1	*RIN3*	-,-,-	ND,-,-	ND,-,-	M,-,-
rs12149828 (chr16:10706328)	FF	*EMP2*	ND,-,-	M,-,-	M,-,-	M,-,-
rs113473882 (chr19:41124155)	FF	*LTBP4*	M,-,-	-,-,-	M,-,-	M,-,-
rs134041 (chr22:28056338)	FEV_1	*MN1*	H,-,-	H,-,-	H,-,-	ND,-,-
rs7050036 (chrX:15964845)	FF	*ZRSR2*	L,-,-	M,-,-	M,-,-	M,-,-
rs7050036 (chrX:15964845)	FF	*AP1S2*	ND,-,-	L,-,-	ND,-,-	ND,-,-

d) Differential gene expression in fetal lung

Publicly available gene expression data from fetal lung samples spanning pseudoglandular and canalicular stages of lung development[13] were queried for a subset of genes for each of the regions that contain the 16 sentinel variants (see criteria in the **Supplementary Methods**). Adj. P < 0.05 are highlighted in bold, and genes with all the probes showing an effect on the same direction and with at least 33% of the probes with adjusted $P < 0.05$ are also highlighted in bold. Chr., chromosome, Probe id, Affymetrix probe id; Average expression, average expression for probe during the entire time period (genes with Average expression values > 8 can be considered highly expressed); Adj. P, adjusted *P* (Benjamini & Hochberg method[14]) for differential expression over time; Beta, mean change in gene expression per day during the studied period (7-22 weeks of gestational age); chr., chromosome.

rs number (chr: position)	Probe id	Gene Symbol	Average expression	Adj. P	Beta
rs6681426 (chr1:150586971)	233724_at	***ARNT***	4.55	**4.58E-03**	0.009
rs6681426 (chr1:150586971)	230619_at	***ARNT***	7.55	**6.32E-03**	0.012
rs6681426 (chr1:150586971)	210828_s_at	***ARNT***	5.03	3.13E-01	0.003
rs6681426 (chr1:150586971)	218222_x_at	***ARNT***	6.82	3.52E-01	0.002
rs6681426 (chr1:150586971)	218221_at	***ARNT***	8.11	5.17E-01	0.002
rs6681426 (chr1:150586971)	231016_s_at	***ARNT***	7.36	9.51E-01	0.000
rs6681426 (chr1:150586971)	232617_at	***CTSS***	6.17	**3.02E-04**	0.015
rs6681426 (chr1:150586971)	202901_x_at	***CTSS***	3.59	**5.90E-03**	0.010
rs6681426 (chr1:150586971)	202902_s_at	***CTSS***	6.06	1.20E-01	0.007
rs6681426 (chr1:150586971)	222212_s_at	*LASS2*	9.61	1.97E-01	0.003
rs201204531 (chr1:219963090)	220435_at	*SLC30A10*	3.42	2.56E-01	0.002
rs61067109 (chr2:18292452)	205968_at	*KCNS3*	7.98	9.79E-01	0.000
rs6441207 (chr3:158282459)	232296_s_at	*GFM1*	7.02	**1.10E-02**	-0.008
rs6441207 (chr3:158282459)	225158_at	*GFM1*	7.73	**2.39E-02**	-0.008
rs6441207 (chr3:158282459)	225161_at	*GFM1*	7.76	**4.34E-02**	-0.006
rs6441207 (chr3:158282459)	232295_at	*GFM1*	3.57	1.94E-01	-0.002
rs6441207 (chr3:158282459)	225153_at	*GFM1*	9.50	6.64E-01	0.001
rs6441207 (chr3:158282459)	220903_at	*GFM1*	3.81	8.44E-01	0.000
rs6441207 (chr3:158282459)	204783_at	***MLF1***	7.23	**1.42E-02**	0.010
rs6441207 (chr3:158282459)	204784_s_at	***MLF1***	8.41	9.07E-01	0.001
rs6856422 (chr4:106841962)	235387_at	*GSTCD*	6.32	**8.55E-04**	-0.012
rs6856422 (chr4:106841962)	1554518_at	*GSTCD*	5.80	**1.50E-02**	-0.005
rs6856422 (chr4:106841962)	220063_at	*GSTCD*	5.61	8.62E-02	-0.004
rs6856422 (chr4:106841962)	241126_at	*GSTCD*	3.46	4.88E-01	0.001
rs6856422 (chr4:106841962)	218616_at	*INTS12*	8.22	2.10E-01	-0.004
rs6856422 (chr4:106841962)	225911_at	*NPNT*	9.01	**6.65E-06**	0.011
rs6856422 (chr4:106841962)	244747_at	*NPNT*	4.36	9.75E-01	0.000
rs148274477 (chr6:142838173)	1553025_at	*GPR126*	3.77	**1.69E-02**	0.004
rs148274477 (chr6:142838173)	213094_at	*GPR126*	9.73	1.98E-01	-0.003
rs148274477 (chr6:142838173)	233887_at	*GPR126*	7.66	9.87E-01	0.000
rs34886460 (chr9:119359372)	236233_at	*TRIM32*	4.75	4.48E-01	0.002
rs34886460 (chr9:119359372)	203846_at	*TRIM32*	8.21	5.55E-01	0.001
rs2274116 (chr9:139094805)	235239_at	*QSOX2*	5.35	6.01E-02	-0.003
rs2274116 (chr9:139094805)	227146_at	*QSOX2*	7.05	1.34E-01	-0.003
rs11383346 (chr12:28283187)	218545_at	*CCDC91*	7.08	6.76E-01	0.002
rs11383346 (chr12:28283187)	1570571_at	*CCDC91*	4.55	8.94E-01	0.001
rs10850377 (chr12:115201436)	219682_s_at	*TBX3*	8.67	**9.91E-03**	0.006
rs10850377 (chr12:115201436)	225544_at	*TBX3*	9.56	2.70E-01	0.002
rs10850377 (chr12:115201436)	222917_s_at	*TBX3*	5.83	4.56E-01	0.002
rs10850377 (chr12:115201436)	229576_s_at	*TBX3*	7.36	5.21E-01	0.002
rs10850377 (chr12:115201436)	229565_x_at	*TBX3*	4.41	6.14E-01	0.001
rs10850377 (chr12:115201436)	228344_s_at	*TBX3*	4.41	7.96E-01	-0.001
rs10850377 (chr12:115201436)	243234_at	*TBX3*	3.63	9.83E-01	0.000
rs7155279 (chr14:92485881)	216657_at	*ATXN3*	3.89	**9.97E-04**	0.006
rs7155279 (chr14:92485881)	217321_x_at	*ATXN3*	3.38	1.00E-01	0.002
rs7155279 (chr14:92485881)	238723_at	*ATXN3*	6.46	1.89E-01	0.007

rs number (chr: position)	Probe id	Gene Symbol	Average expression	Adj. P	Beta
rs7155279 (chr14:92485881)	205416_s_at	*ATXN3*	5.48	4.77E-01	-0.002
rs7155279 (chr14:92485881)	235240_at	*ATXN3*	6.87	6.96E-01	-0.001
rs7155279 (chr14:92485881)	205415_s_at	*ATXN3*	6.50	9.51E-01	0.000
rs7155279 (chr14:92485881)	233182_x_at	*ATXN3*	5.27	9.59E-01	0.000
rs7155279 (chr14:92485881)	230211_at	*TRIP11*	4.74	3.53E-01	0.003
rs7155279 (chr14:92485881)	210760_x_at	*TRIP11*	4.62	6.02E-01	0.001
rs7155279 (chr14:92485881)	236160_at	*TRIP11*	6.77	6.46E-01	-0.001
rs7155279 (chr14:92485881)	209778_at	*TRIP11*	5.61	6.75E-01	0.002
rs117068593 (chr14:93118229)	1562005_at	*RIN3*	4.13	5.05E-02	0.004
rs117068593 (chr14:93118229)	60471_at	*RIN3*	7.46	2.57E-01	0.002
rs117068593 (chr14:93118229)	220439_at	*RIN3*	4.30	4.91E-01	-0.001
rs117068593 (chr14:93118229)	219457_s_at	*RIN3*	5.94	7.43E-01	-0.001
rs117068593 (chr14:93118229)	219456_s_at	*RIN3*	5.00	7.92E-01	0.001
rs12149828 (chr16:10706328)	204975_at	***EMP2***	8.29	**3.68E-06**	0.014
rs12149828 (chr16:10706328)	225078_at	***EMP2***	9.08	**1.42E-05**	0.011
rs12149828 (chr16:10706328)	225079_at	***EMP2***	8.71	**8.62E-05**	0.010
rs12149828 (chr16:10706328)	238500_at	***EMP2***	4.73	9.78E-02	0.004
rs113473882 (chr19:41124155)	227989_at	*LTBP4*	4.68	1.30E-01	-0.003
rs113473882 (chr19:41124155)	204442_x_at	*LTBP4*	8.44	3.89E-01	0.004
rs113473882 (chr19:41124155)	213176_s_at	*LTBP4*	8.03	9.31E-01	0.001
rs113473882 (chr19:41124155)	210628_x_at	*LTBP4*	6.83	9.96E-01	0.000
rs134041 (chr22:28056338)	205330_at	*MN1*	5.44	1.60E-01	-0.005
rs7050036 (chrX:15964845)	228415_at	***AP1S2***	7.82	**9.12E-05**	-0.009
rs7050036 (chrX:15964845)	230264_s_at	***AP1S2***	9.67	**3.97E-02**	-0.004
rs7050036 (chrX:15964845)	203300_x_at	***AP1S2***	9.07	1.52E-01	-0.004
rs7050036 (chrX:15964845)	203299_s_at	***AP1S2***	7.55	6.26E-01	-0.001
rs7050036 (chrX:15964845)	208174_x_at	*ZRSR2*	6.99	4.29E-01	0.002
rs7050036 (chrX:15964845)	213876_x_at	*ZRSR2*	7.18	5.82E-01	0.001

e) **Differential gene expression in COPD vs. non COPD ever smoker samples**

Publicly available gene expression data from bronchial brushings of individuals with COPD and of ever smoking individuals without COPD[15] were queried for a subset of genes for each of the regions that contain the 16 sentinel variants (see criteria in **Supplementary Methods**) are presented. There was no probe available for *ZRSR2* in this analysis. Adj. P < 0.05 are highlighted in bold. “Probe id”, affymetrix probe id; Adj.P, *P* value after adjustment for multiple testing using the Benjamini & Hochberg method[14]; FC, fold change between cases and controls (>1 up regulated in cases); chr., chromosome.

rs number (chr: position)	Probe id	Gene Symbol	Adj. P	FC
rs6681426 (chr1:150586971)	405_at	*ARNT*	8.53E-02	1.04
rs6681426 (chr1:150586971)	1520_at	*CTSS*	1.04E-01	1.06
rs6681426 (chr1:150586971)	29956_at	*LASS2*	5.78E-01	1.02
rs201204531 (chr1:219963090)	55532_at	*SLC30A10*	9.35E-01	1.00
rs61067109 (chr2:18292452)	3790_at	*KCNS3*	9.23E-01	0.99
rs6441207 (chr3:158282459)	85476_at	*GFM1*	7.28E-01	0.99
rs6441207 (chr3:158282459)	4291_at	*MLF1*	**1.74E-03**	1.12
rs6856422 (chr4:106841962)	79807_at	*GSTCD*	3.84E-01	1.03
rs6856422 (chr4:106841962)	57117_at	*INTS12*	2.57E-01	1.04
rs6856422 (chr4:106841962)	255743_at	*NPNT*	7.90E-02	1.10
rs148274477 (chr6:142838173)	57211_at	*GPR126*	2.86E-01	1.07
rs34886460 (chr9:119359372)	22954_at	*TRIM32*	8.29E-01	1.01
rs2274116 (chr9:139094805)	169714_at	*QSOX2*	3.22E-01	0.97
rs11383346 (chr12:28283187)	55297_at	*CCDC91*	1.26E-01	1.05
rs10850377 (chr12:115201436)	6926_at	*TBX3*	5.43E-01	0.97
rs7155279 (chr14:92485881)	4287_at	*ATXN3*	**2.07E-02**	1.07
rs7155279 (chr14:92485881)	9321_at	*TRIP11*	5.72E-01	1.03
rs117068593 (chr14:93118229)	79890_at	*RIN3*	1.65E-01	0.96
rs12149828 (chr16:10706328)	2013_at	*EMP2*	9.84E-01	1.00
rs113473882 (chr19:41124155)	8425_at	*LTBP4*	1.18E-01	0.95
rs134041 (chr22:28056338)	4330_at	*MN1*	8.36E-01	0.99
rs7050036 (chrX:15964845)	8905_at	*AP1S2*	6.61E-01	1.02
rs7050036 (chrX:15964845)	NA	*ZRSR2*	NA	NA

f) Sentinel and functional variants annotation and joint analysis results

Annotations were provided using ENSEMBL's Variant Effect Predictor[16]. Functional variants were defined as variants within 1 Mb of the sentinel variant, which were annotated as 'deleterious' by SIFT[17], 'probably damaging' or 'potentially damaging' by PolyPhen-2[18], had a CADD[19] scaled score ≥20, or had a GWAVA score >0.5, and were in linkage disequilibrium (LD) with the sentinel variant (r^2>0.3) and/or had nominal evidence of association ($P<5\times10^{-4}$) (see **Supplementary Methods**). Only functional variants, which explained the association of the sentinel variant (sentinel variant joint P>0.01), are listed. *RIN3* locus, where the sentinel variant is a missense variant, is also presented. Stage 1 unconditional analysis results and joint analysis results are shown in alternating white and grey colours. Conditional analyses were undertaken using GCTA[20] and LD information was estimated using UK BiLEVE data (details in the **Supplementary Methods**). GCTA did not produce joint results for rs34093919 and rs113473882, since they are almost in perfect LD. Chr., chromosome; freq., frequency.

rs number (chr: position)	Variant type	Function	Gene Symbol	SIFT	PolyPhen	GWAVA score	CADD scaled score	Measure	R^2 with sentinel	MAF	Unconditional results			Joint analysis		
											Beta	SE	P	Beta	SE	P
rs6681426 (chr1:150586971)	sentinel	Intron variant	*ENSA*	-	-	0.21	0.93	FEV_1	1.00	0.36	0.042	0.008	1.07E-07	0.034	0.014	1.23E-02
rs9803935 (chr1:150552622)	functional	Upstream gene variant	*MCL1*	-	-	0.51	7.67	FEV_1	0.68	0.48	0.036	0.008	2.14E-06	0.009	0.013	4.83E-01
rs6681426 (chr1:150586971)	sentinel	Intron variant	*ENSA*	-	-	0.21	0.93	FEV_1	1.00	0.36	0.042	0.008	1.07E-07	0.032	0.014	2.02E-02
rs1053732 (chr1:150597890)	functional	Intron variant	*ENSA*	-	-	0.55	3.88	FEV_1	0.68	0.48	0.037	0.008	1.20E-06	0.011	0.013	3.93E-01
rs6441207 (chr3:158282459)	sentinel	Intron variant, nc transcript variant	*AK097794*	-	-	0.1	0.68	FVC	1.00	0.41	0.036	0.008	4.54E-06	0.030	0.014	3.76E-02
rs939117 (chr3:158200869)	functional	Intron variant	*RSRC1*	-	-	0.6	16.60	FVC	0.73	0.47	0.031	0.008	3.89E-05	0.007	0.014	6.29E-01
rs148274477 (chr6:142838173)	sentinel	Intergenic variant	-	-	-	0.04	12.33	FEV_1/ FVC	1.00	0.02	-0.161	0.027	2.68E-09	-0.095	0.056	8.98E-02
rs17280293 (chr6:142688969)	functional	Missense variant	*GPR126*	Deleterious (0)	Possibly damaging (0.602)	-	21.90	FEV_1/ FVC	0.85	0.02	-0.154	0.026	4.72E-09	-0.073	0.055	1.82E-01
rs12149828 (chr16:10706328)	sentinel	Intergenic variant	-	-	-	0.21	1.06	FEV_1/ FVC	1.00	0.17	0.049	0.010	3.16E-06	0.048	0.021	1.88E-02
rs12448455 (chr16:10711678)	functional	Intergenic variant	-	-	-	0.6	3.84	FEV_1/ FVC	0.80	0.19	0.039	0.010	5.74E-05	0.000	0.019	9.86E-01
rs113473882 (chr19:41124155)	sentinel	Intron variant	*LTBP4*	-	-	0.11	2.46	FEV_1/ FVC	1.00	0.02	-0.174	0.033	1.48E-07	NA	NA	NA
rs34093919 (chr19:41117300)	functional	Missense variant, splice region variant	*LTBP4*	Deleterious (0.05)	Possibly damaging (0.841)	-	18.35	FEV_1/ FVC	0.99	0.02	-0.163	0.032	4.82E-07	NA	NA	NA
rs134041 (chr22:28056338)	sentinel	Intergenic variant	-	-	-	0.46	19.28	FEV_1	1.00	0.43	-0.045	0.008	4.19E-09	-0.036	0.016	2.63E-02
rs134117 (chr22:28082715)	functional	Intron variant, nc transcript variant	*RP1-213J1P_B .1*	-	-	0.6	12.58	FEV_1	0.78	0.41	-0.042	0.008	4.43E-08	-0.010	0.016	5.51E-01
rs117068593 (chr4:93118229)	sentinel	Missense variant	*RIN3*	Tolerated (0.07)	Unknown (0)	-	14.37	FEV_1	1	0.18	-0.048	0.010	2.72E-06	NA	NA	NA

Supplementary Table 4 Literature search results for genes overlapping or proximal to novel lung function sentinel SNP and genes which contain a SNP with r^2>0.3 with lead SNP

A systematic literature search was performed for each gene. Three main websites were used. Firstly, each 'gene name' was searched for in Gene Cards (www.genecards.org), as well as the International Mouse Phenotyping Consortium database (www.mousephenotype.org). Secondly, each 'gene name', 'gene name' and 'lung', 'gene name' and 'lung disease', and 'gene name' and 'development' were searched for in Pubmed (www.pubmed.com). Information regarding gene function, association to human disease and animal model phenotyping was identified. Genes included in the gene column include genes containing SNP with r^2>0.3 with sentinel SNP or genes selected as priority genes for each region (see criteria in **Supplementary Methods**). Chr., chromosome..

rs number (chr: postion:variant type, function)	Genes containing SNPs with r^2>0.3 with sentinel variant or implicated through eQTL analyses	Description of genes in region and biological plausibility	Animal model with lung function or pulmonary phenotype
rs6681426 (1:150586971:SNP, intergenic)	*ADAMTSL4;ADAMTSL4-AS1;ANXA9;ARNT;CERS2 (LASS2);CTSK;CTSS; CYCSP51;ENSA;GOLPH3L;HORMAD1;LINC00568;MCL1;RN7SL473P;RP11-235D19.2;RP11-363I22.3;SETDB1;SNORA40*	***ADAMTSL4:*** Member of the ADAMTS (a disintegrin and metalloproteinase with thrombospondin motifs)-like gene family. Associated diseases include eye disorders such as ectopia lentis[21].<br>***ADAMTSL4-AS1:*** ADAMTSL4 antisense RNA 1.<br>***ANXA9:*** Annexins are calcium-dependent phospholipid-binding proteins. Has calcium-binding sites which are required for annexins to aggregate and cooperatively bind anionic phospholipids and extracellular matrix proteins.<br>***ARNT:*** Binds to ligand-bound aryl hydrocarbon receptor. Aids the movement of a complex to the nucleus, where it promotes the expression of genes involved in xenobiotic metabolism. Also a co-factor for transcriptional regulation by hypoxia-inducible factor 1 which is important in lung development[22]. Chromosomal translocation ARNT with ETV6 gene on chromosome 12 has been described in leukaemia[23].<br>***CERS2* (*LASS2*):** Also known as LASS2. Involved in sphingolipid synthesis[24]. Associated with Breast Cancer[25].<br>***CTSK:*** Lysosomal cysteine proteinase involved in bone remodeling and resorption[26]. Mutations in this gene are the cause of pycnodysostosis[27].<br>***CTSS:*** Lysosomal cysteine proteinase which is involved in the degradation of antigenic proteins to peptides for presentation on MHC class II molecules[28]. Associated diseases include Athersclerosis, Lung Fibrosis and defects in the immune system[29].<br>***CYCSP51:*** Cytochrome C, Somatic Pseudogene 51.<br>***ENSA:*** Inhibits sulfonylurea binding to beta-cell membranes, reduces cloned K(ATP) channel currents, and stimulates insulin secretion from beta-cells, therefore ia a candidate gene involved in Diabetes[30].<br>***GOLPH3L:*** Potential regulatory role in Golgi trafficking[31]. Associated with Rhabdomyosarcoma[32].<br>***HORMAD1:*** May play a role in the progression of meiosis and is a potential marker for cancer [33].<br>***LINC00568:*** Long Intergenic Non-Protein Coding RNA 568.<br>***MCL1:*** Regulates apoptosis[34]. Mutations found in Leukemia [35].<br>***RN7SL473P:*** RNA, 7SL, Cytoplasmic 473, Pseudogene.<br>***SETDB1:*** Histone methyltransferase which regulates histone methylation, gene silencing, transcriptional repression and is associated with Huntington's disease[36].<br>***SNORA40:*** Small nucleolar RNA40.	***CERS2* (*LASS2*):** null mice show lung inflammation and airway obstruction[37].<br>***CTSK:*** null mice develop more lung fibrosis than wild type mice[38].
rs201204531 (1:219963090:INDEL, intergenic)	*AC096643.1;RNA5SP76;SLC30A10*	***RNA5SP76:*** RNA, 5S Ribosomal Pseudogene 76, no data available.<br>***SLC30A10:*** Protein critical in maintaining manganese levels with loss of function mutations resulting in dystonia, adult-onset parkinsonism and chronic liver disease[39].	
rs61067109 (2:18292452:SNP, intergenic)	*KCNS3*	***KCNS3:*** Regualtes the resting membrane potential and SNP's in this gene are associated with airway hyper-responsiveness[40]. Associated with migraine[41].	

rs number (chr: postion:variant type, function)	Genes containing SNPs with r^2>0.3 with sentinel variant or implicated through eQTL analyses	Description of genes in region and biological plausibility	Animal model with lung function or pulmonary phenotype
rs6441207 (3:158282459:SNP, ncRNA_intronic)	*GFM1; MLF1;MTAPP1;RARRES1;AK097794(RP11-538P18.2);RP11-79M21.3;RSRC1*	***GFM1***: Mitochondrial GTPase that catalyzes the GTP-dependent ribosomal translocation step during translation elongation[42]. Diseases associated with GFM1 include combined oxidative phosphorylation deficiency 1[43].<br>***MLF1:*** Involved in lineage commitment of primary haemopoietic progenitors by restricting erythroid formation and enhancing myeloid formation [44]. Mutations involved in myelodysplastic syndromes and myeloid leukemia [45]. Binds DNA and affects the expression of a number of genes so may function as a transcription factor in the nucleus [46].<br>***MTAPP1:*** methylthioadenosine phosphorylase pseudogene 1.<br>***RARRES1:*** Retinoid acid (RA) receptor-responsive gene which inhibits the cytoplasmic carboxypeptidase AGBL2 and may regulate the alpha-tubulin tyrosination cycle[47]. Associated with Ovarian Endometriosis and downregulated in prostate cancer[48].<br>***RSRC1:*** Encodes a serine and arginine rich-related protein involved in mRNA splicing. Associated with height [49] and Schizophrenia[50].	
rs6856422 (4:106841962:SNP, intronic)	*GSTCD;INTS12;NPNT*	***GSTCD:*** Has homology with the glutathione S-transferase (GST) super family of enzymes[51]. May be involved in the detoxification of products of oxidative stress and synthesis of steroid hormones[52]. However, GSTCD lacks key functional domains important for GST activity. *GSTCD* expression in human lung tissue is ubiquitous[9].<br>***INTS12:*** Subunit of the Integrator complex, which associates with the C-terminal domain of RNA polymerase II large subunit and mediates 3` end processing of small nuclear RNAs[53]. *INTS12* expression in human lung is found predominantly in epithelial cells and pneumocytes[9]<br>***NPNT:*** Potentially involved in the development of many tissues[54]. Associated with include acute and chronic hepatitis[55].	
rs148274477 (6:142838173:SNP, intergenic)	*GPR126;LOC153910(RP11-440G9.1)*	***GPR126:*** Encodes a G protein-coupled receptor, with variations affecting stature[49,56-58], birth length[59], and adolescent idiopathic scoliosis[60]. May be required for normal differentiation of promyelinating Schwann cells and for normal myelination of axons[61]. It is expressed in adult mice lung[62]. It is required for mice embryonic viability and cardiovascular development[63]	
rs34886460 (9:119359372:INDEL, intronic)	*TRIM32*	***ASTN2:*** Expressed in the brain and may function in neuronal migration. Associated with migraine[64].<br>***TRIM32:*** Protein interacts with the activation domain of the HIV-1 Tat protein. Associated with Bardet-Biedl syndrome (a ciliopathic disorder)[65].	
rs2274116 (9:139094805:SNP, exonic)	*LHX3;QSOX2*	***LHX3:*** Carries the LIM domain, a unique cysteine-rich zinc-binding domain. Transcription factor required for pituitary development and motor neuron specification. Mutations cause combined pituitary hormone deficiency 3[66].<br>***QSOX2:*** May contribute to disulfide bond formation in a variety of secreted proteins. Regulates the sensitization of neuroblastoma cells for IFN-gamma-induced cell death[67].	
rs11383346 (12:28283187:INDEL, intergenic)	*CCDC91;RP11-874G11.1;RP11-967K21.1;RP11-967K21.2*	***CCDC91:*** Associated with brain white matter structure and development[68].	
rs10850377(12:115201436:SNP, intergenic)	*RP4-601P9.1;RP4-601P9.2; TBX3*	***TBX3:*** Encodes a transcription factor involved in the regulation of developmental processes in limbs and genitals[69]. Mutations cause ulnar-mammary and Holt-Oram syndromes[70]. It is involved in the TGFβ1 signaling pathway[71,72].	
rs7155279 (14:92485881:SNP, intronic)	*ATXN3;FBLN5;RP11-529H20.6;TRIP11*	***ATXN3:*** Deubiquitinating enzyme involved in protein homeostasis maintenance, transcription, cytoskeleton regulation, myogenesis and degradation of misfolded chaperone substrates. Associated with Machado-Joseph disease (spinocerebellar ataxia-3)[73].<br>***FBLN5:*** Encodes a secreted, extracellular matrix protein. Promotes adhesion of endothelial cells through interaction of integrins and the RGD motif[74]. Defects in gene cause Cutis Laxa, autosomal recessive, type ia, and Hereditary Sensorimotor Neuropathy with Hyperelastic Skin[75]. Involved in tissue repair in COPD[76] and elastogenesis and lung	***FBLN5:*** Homozygous mutant mice have expanded lungs containing dilated alveoli (most severe in peripheral regions)[80].

rs number (chr: postion:variant type, function)	Genes containing SNPs with r^2>0.3 with sentinel variant or implicated through eQTL analyses	Description of genes in region and biological plausibility	Animal model with lung function or pulmonary phenotype
		development[77].<br>***TRIP11:*** Protein is associated with the Golgi apparatus[78]. Mutations in this gene cause achondrogenesis type IA (most severe form of dwarfism bone/cartilage malformations)[79].	Adult *fibulin-5 -/-* mice have disruptions in the alveolar structure and disruption to distal airways was found in P3 neonates[81].
rs117068593 (14:93118229:SNP, exonic)	*RIN3*	***RIN3:*** Member of the RIN family of Ras interaction-interference protein[82]. Associated with Paget's disease[83], COPD[84] and Alzheimer's disease[85].	
rs12149828 (16:10706328:SNP, intergenic)	*EMP2;TEKT5*	***EMP2:*** Involved in molecular interactions during implantation[86]. Diseases associated include charcot-marie-tooth disease type 1[87].<br>***TEKT5:*** Tektins are structural components of ciliary and flagellar microtubules. TEKT5 is implicated in sperm motility in mice[88].	
rs113473882 (19:41124155:SNP, intronic)	*CTC-490E21.12;CYP2B6;CYP2B7P;ITPKC;LTBP4;NUMBL;RAB4B*	***CYP2B6 and CYP2B7P:*** Cytochrome P450 proteins are monooxygenases which are involved in drug metabolism and synthesis of cholesterol, steroids and lipids. Enzymes are involved in an NADPH-dependent electron transport pathway**.**<br>***ITPKC:*** Encodes inositol 1,4,5-trisphosphate (Ins(1,4,5)P(3)) 3-kinase enzymes. Has nuclear import and export activity. Associated with Kawasaki disease[89].<br>***LTBP4:*** Binds transforming growth factor beta (TGFB) as it is secreted and targeted to the extracellular matrix. COPD risk associated gene with deregulated expression in the basal cells of smokers[90].<br>***NUMBL:*** Has a role in neurogenesis[91].<br>***RAB4B:*** RAB proteins are members of the RAS superfamily of small GTPases that are involved in vesicular trafficking[92].	***LTBP4:*** Mice deficient in Latent TGFβ Binding Protein 4 (Ltbp4) display a defect in lung septation and elastogenesis[77]. Mice with *LTBP4* disruption show a pulmonary emphysema-like condition at birth which increases in severity with age. The lungs of *LTBP4*-disrupted mice had less alveolar spaces and those present were enlarged and inflated. Mice also developed cardiomyopathy and colorectal cancers[93].
rs134041(22:28056338:SNP, intergenic)	*MN1; RP1-213J1P__B.1;RP11-375H17.1*	***MN1:*** Translocation of MN1 is associated with Leukemia[94].	
rs7050036(X:15964845:SNP, intergenic)	*AP1S2;GRPR;RNU5F-7P;SETP15;ZRSR2*	***AP1S2:*** Adaptor protein complex 1 subunit 2 found at the cytoplasmic face of coated vesicles in the Golgi complex. Mediates the recruitment of clathrin and the recognition of sorting signals within transmembrane receptors. Associated diseases include dandy-walker malformation with mental retardation[95,96], and basal ganglia disease [97].<br>***GRPR:*** Receptor for Gastrin-releasing peptide which regulates gastrointestinal and central nervous system functions including smooth muscle cell contraction and epithelial cell proliferation.Expressed by pulmonary neuroendocrine cells and known to stimulate growth and maturation of lung development [98,99].<br>***RNU5F-7P:*** RNA, U5F Small Nuclear 7, Pseudogene.<br>***SETP15:*** SET Pseudogene 15.<br>***ZRSR2:*** Zinc Finger with RNA-Binding Motif which encodes an essential splicing factor which associates with U2 snRNA. Linked to myelodysplasia[100].	

Supplementary Table 5 Associations of top signals with other traits

Associations with lung function in children (a)), separately in ever and never smokers (b)), with smoking behaviour (c)) and with other traits as in the GWAS catalog[101] (d)).

a) Effect of top hits on children

Stage 2 results, as well as results in 5,0625062 children (8-9 years of age) in ALSPAC[102] are presented for the new 16 sentinel variants. *P* values that meet a Bonferroni correction for 16 tests ($P < 3.13 \times 10^{-3}$) in ALSPAC are highlighted in bold. Chr., chromosome; dist., distance; Freq., frequency; N., effective sample size; Imp., imputation.

rs number (chr:position)	Gene Symbol (function)	Measure	Coded allele	Other allele	**Stage 2**				**ALSPAC**				
					**Coded allele freq.**	**Beta**	**SE**	**P**	**Coded allele freq.**	**Imp quality**	**Beta**	**SE**	**P**
rs6681426 (chr1:150586971)	*MCL1* (dist=34757), *ENSA* (dist=7628)	FEV_1	G	A	0.35	0.021	0.006	1.11E-03	0.35	0.99	0.046	0.030	1.25E-01
rs201204531 (chr1:219963090)	*LYPLAL1* (dist=576883), *RNU5F-1* (dist=83529)	FEV_1/FVC	A	ATG	0.38	-0.027	0.006	1.76E-05	0.38	0.97	-0.048	0.029	9.79E-02
rs61067109 (chr2:18292452)	*KCNS3* (dist=178227), *NT5C1B-RDH14* (dist=443537)	FEV_1/FVC	G	A	0.77	-0.042	0.007	6.61E-09	0.76	1.00	-0.043	0.033	1.93E-01
rs6441207 (chr3:158282459)	*RP11-538P18.2* (ncRNA_intronic)	FVC	C	T	0.59	0.036	0.006	5.70E-09	0.59	0.98	0.118	0.029	**4.72E-05**
rs6856422 (chr4:106841962)	*NPNT* (intronic)	FEV_1/FVC	G	T	0.56	-0.055	0.006	1.13E-17	0.55	0.86	-0.074	0.030	1.36E-02
rs148274477 (chr6:142838173)	*GPR126* (dist=70770), *RP11-440G9.1* (dist=9419)	FEV_1/FVC	C	T	0.97	-0.162	0.019	5.66E-18	0.97	0.76	-0.165	0.094	7.92E-02
rs34886460 (chr9:119359372)	*ASTN2* (intronic)	FEV_1/FVC	T	TA	0.54	0.025	0.006	4.09E-05	0.54	0.98	-0.019	0.028	4.97E-01
rs2274116 (chr9:139094805)	*LHX3* (exonic)	FVC	C	T	0.66	0.038	0.007	4.16E-09	0.68	0.84	0.060	0.033	6.90E-02
rs11383346 (chr12:28283187)	*PTHLH* (dist=158271), *CCDC91* (dist=126946)	FVC	A	AT	0.41	-0.041	0.006	4.29E-11	0.42	0.97	-0.014	0.029	6.29E-01
rs10850377 (chr12:115201436)	*TBX3* (dist=79467), *MED13L* (dist=1194945)	FEV_1	G	A	0.65	-0.028	0.006	1.50E-05	0.65	0.96	-0.031	0.031	3.17E-01
rs7155279 (chr14:92485881)	*TRIP11* (intronic)	FEV_1	G	T	0.64	-0.022	0.006	4.02E-04	0.64	0.98	-0.013	0.031	6.75E-01
rs117068593 (chr14:93118229)	*RIN3* (exonic)	FEV_1	C	T	0.81	-0.027	0.008	5.68E-04	0.82	0.94	-0.021	0.039	5.90E-01
rs12149828 (chr16:10706328)	*EMP2* (dist=31789), *TEKT5* (dist=15033)	FEV_1/FVC	G	A	0.83	0.035	0.008	3.33E-05	0.83	0.91	-0.007	0.039	8.58E-01
rs113473882 (chr19:41124155)	*LTBP4* (intronic)	FEV_1/FVC	T	C	0.99	-0.138	0.028	9.94E-07	0.99	0.76	0.177	0.147	2.29E-01
rs134041 (chr22:28056338)	*MIAT*(dist=983898), *MN1* (dist=87927)	FEV_1	T	C	0.44	-0.018	0.006	4.12E-03	0.44	0.99	0.003	0.029	9.18E-01
rs7050036 (chrX:15964845)	*AP1S2* (dist=91708), *GRPR* (dist=176579)	FEV_1/FVC	T	A	0.39	-0.013	0.005	1.60E-02	0.40	0.98	0.017	0.023	4.70E-01

b) **Effect of novel signals on ever smokers and never smokers in stage 1**

Stage 1 results for genome-wide significant associations ($P<5x10^{-8}$) with either FEV_1, FEV_1/FVC or FVC separately in ever smokers and never smokers, and the interaction *P* value are shown. Chr., chromosome.

rs number (chr:position)	Coded allele	Measure	Ever smokers			Never smokers			Interaction
			Beta	SE	P	Beta	SE	P	P
rs6681426 (chr1:150586971)	G	FEV_1	0.038	0.010	2.54E-04	0.049	0.012	2.07E-05	4.82E-01
rs201204531 (chr1:219963090)	A	FEV_1/FVC	-0.039	0.010	1.94E-04	-0.036	0.012	2.63E-03	8.32E-01
rs61067109 (chr2:18292452)	G	FEV_1/FVC	-0.043	0.012	3.61E-04	-0.054	0.013	4.04E-05	5.51E-01
rs6441207 (chr3:158282459)	C	FVC	0.029	0.010	5.38E-03	0.042	0.012	3.02E-04	4.09E-01
rs6856422 (chr4:106841962)	G	FEV_1/FVC	-0.043	0.011	1.02E-04	-0.044	0.012	4.27E-04	9.69E-01
rs148274477 (chr6:142838173)	C	FEV_1/FVC	-0.195	0.037	1.01E-07	-0.118	0.039	2.51E-03	1.51E-01
rs34886460 (chr9:119359372)	T	FEV_1/FVC	0.027	0.010	6.96E-03	0.055	0.011	7.06E-07	6.27E-02
rs2274116 (chr9:139094805)	C	FVC	0.038	0.011	8.04E-04	0.041	0.013	1.19E-03	8.75E-01
rs11383346 (chr12:28283187)	A	FVC	-0.036	0.010	5.67E-04	-0.049	0.011	1.54E-05	3.91E-01
rs10850377 (chr12:115201436)	G	FEV_1	-0.052	0.011	1.66E-06	-0.042	0.012	3.20E-04	5.35E-01
rs7155279 (chr14:92485881)	G	FEV_1	-0.049	0.010	3.11E-06	-0.035	0.011	2.47E-03	3.66E-01
rs117068593 (chr14:93118229)	C	FEV_1	-0.053	0.014	7.86E-05	-0.038	0.015	1.09E-02	4.45E-01
rs12149828 (chr16:10706328)	G	FEV_1/FVC	0.057	0.014	3.87E-05	0.038	0.015	1.41E-02	3.45E-01
rs113473882 (chr19:41124155)	T	FEV_1/FVC	-0.206	0.044	3.32E-06	-0.135	0.048	5.42E-03	2.78E-01
rs134041 (chr22:28056338)	T	FEV_1	-0.043	0.010	2.25E-05	-0.045	0.011	4.93E-05	8.88E-01
rs7050036 (chrX:15964845)	T	FEV_1/FVC	-0.048	0.011	1.72E-05	-0.033	0.015	3.16E-02	4.46E-01

c) Associations with smoking behaviour in UK BiLEVE for novel signals

Association of the 16 sentinel variants with heavy vs. never smoking status in UK BiLEVE (N heavy=24,457, N never=24,474). *P* that met a Bonferroni corrected threshold for 16 tests ($P<3.13\times10^{-3}$) are highlighted in bold. Chr., chromosome.

rs number (chr: position)	Coded allele	Other allele	MAF	Imputation quality	Beta	SE	P
rs6681426 (chr1:150586971)	A	G	0.35	1.00	-0.003	0.013	8.07E-01
rs201204531 (chr1:219963090)	ATG	A	0.38	0.99	0.042	0.013	**1.54E-03**
rs61067109 (chr2:18292452)	A	G	0.23	1.00	-0.007	0.015	6.33E-01
rs6441207 (chr3:158282459)	T	C	0.40	1.00	-0.008	0.013	5.30E-01
rs6856422 (chr4:106841962)	T	G	0.45	0.95	-0.010	0.013	4.50E-01
rs148274477 (chr6:142838173)	T	C	0.03	0.98	-0.067	0.040	9.45E-02
rs10512249 (chr9:98256309)	A	G	0.10	0.99	0.034	0.022	1.16E-01
rs34886460 (chr9:119359372)	TA	T	0.47	1.00	0.020	0.013	1.23E-01
rs2274116 (chr9:139094805)	T	C	0.34	0.98	-0.018	0.014	1.75E-01
rs11383346 (chr12:28283187)	AT	A	0.41	1.00	-0.029	0.013	2.47E-02
rs10850377 (chr12:115201436)	A	G	0.34	0.98	-0.019	0.014	1.52E-01
rs7155279 (chr14:92485881)	T	G	0.36	1.00	0.015	0.013	2.63E-01
rs117068593 (chr14:93118229)	T	C	0.19	0.98	-0.003	0.016	8.50E-01
rs12149828 (chr16:10706328)	A	G	0.16	0.94	-0.017	0.018	3.53E-01
rs113473882 (chr19:41124155)	C	T	0.01	0.95	-0.150	0.067	2.55E-02
rs134041 (chr22:28056338)	C	T	0.44	0.98	-0.007	0.013	5.80E-01
rs7050036 (chrX:15964845)	A	T	0.39	0.98	0.000	0.011	9.69E-01

d) Associations of top hits with other traits

The GWAS catalog[101] was searched for variants in 2Mb regions centred on the sentinel variant for the 16 sentinel variants. Variants that were genome-wide significant ($P<5x10^{-8}$) in the GWAS catalog[101] and were in LD (r^2 >0.3) with the sentinel variants, or were in genes that contained at least one variant in LD (r^2>0.3) with the sentinel variants were selected. Chr., chromosome.

Sentinel rs number (chr:position)	rs number (chr:position)	R^2 with sentinel	PubMed id	Trait	Gene Symbol	P
rs6681426 (chr1:150586971)	rs2230061 (chr1:150727539)	6.44E-01	24064335	Fat body mass	*CTSS*	4.00E-08
rs6681426 (chr1:150586971)	rs7412746 (chr1:150860471)	4.09E-01	21983785	Melanoma	*ARNT, SETDB1, LASS2, ANXA9, MCL1, CTSK*	9.00E-11
rs6681426 (chr1:150586971)	rs267734 (chr1:150951477)	1.30E-01	20383146	Chronic kidney disease	*ANXA9,FAM63A,PRUNE,BNIPL,LAS S2,SETDB1*	1.00E-12
rs6681426 (chr1:150586971)	rs267733 (chr1:150958836)	8.34E-02	24097068	LDL cholesterol	*ANXA9, CERS2*	5.00E-09
rs201204531 (chr1:219963090)	rs2820443 (chr1:219753509)	2.93E-02	23754948	Sexual dimorphism in anthropometric traits	*LYPLAL1, SLC30A10, ZC3H11B*	7.00E-16
rs6441207 (chr3:158282459)	rs2362965 (chr3:158109379)	6.33E-01	23563607	Height	*RSRC1,SHOX2*	2.00E-09
rs6856422 (chr4:106841962)	rs11727189 (chr4:106619140)	7.48E-02	20010835	Pulmonary function	*INTS12, NPNT, FLJ20184, GSTCD*	5.00E-17
rs6856422 (chr4:106841962)	rs10516526 (chr4:106688904)	7.50E-02	20010834	Pulmonary function	*GSTCD*	2.00E-23
rs6856422 (chr4:106841962)	rs17331332 (chr4:106808107)	7.89E-02	23284291	Pulmonary function (interaction)	*INTS12, GSTCD, NPNT*	1.00E-16
rs148274477 (chr6:142838173)	rs7741741 (chr6:142655801)	6.51E-02	23563607	Height	*GPR126*	1.00E-20
rs148274477 (chr6:142838173)	rs6570507 (chr6:142679572)	5.94E-02	23666238	Scoliosis	*GPR126*	4.00E-14
rs148274477 (chr6:142838173)	rs6570507 (chr6:142679572)	5.94E-02	19343178	Height	*GPR126*	4.00E-11
rs148274477 (chr6:142838173)	rs4896582 (chr6:142703877)	5.52E-02	18391950	Height	*GPR126*	2.00E-18
rs148274477 (chr6:142838173)	rs3817928 (chr6:142750516)	1.02E-01	23284291	Pulmonary function (interaction)	*GPR126*	3.00E-12
rs148274477 (chr6:142838173)	rs3817928 (chr6:142750516)	1.02E-01	20010835	Pulmonary function	*GPR126*	1.00E-09
rs148274477 (chr6:142838173)	rs3748069 (chr6:142767633)	6.58E-02	18391951	Height	*GPR126*	5.00E-14
rs148274477 (chr6:142838173)	rs7763064 (chr6:142797289)	6.47E-02	20881960	Height	*GPR126*	1.00E-33
rs34886460 (chr9:119359372)	rs6478241 (chr9:119252629)	5.27E-02	23793025	Migraine - clinic-based	*ASTN2*	1.00E-09
rs34886460 (chr9:119359372)	rs6478241 (chr9:119252629)	5.27E-02	22683712	Migraine	*ASTN2*	4.00E-08
rs2274116 (chr9:139094805)	rs7860634 (chr9:139089679)	3.74E-01	23408906	Thyroid hormone levels	*LHX3*	2.00E-14
rs2274116 (chr9:139094805)	rs7849585 (chr9:139111870)	1.51E-01	20881960	Height	*QSOX2*	5.00E-14
rs2274116 (chr9:139094805)	rs12338076 (chr9:139121740)	1.55E-01	20189936	Height	*LHX3, QSOX2*	2.00E-08
rs11383346 (chr12:28283187)	rs2638953 (chr12:28534415)	3.00E-01	20881960	Height	*CCDC91*	7.00E-17

Sentinel rs number (chr:position)	rs number (chr:position)	R^2 with sentinel	PubMed id	Trait	Gene Symbol	P
rs11383346 (chr12:28283187)	rs10843164 (chr12:28569714)	2.89E-01	23563607	Height	*CCDC91*	6.00E-12
rs7155279 (chr14:92485881)	rs7153027 (chr14:92427222)	7.41E-01	18391951	Height	*TRIP11, FBLN5, ATXN3, CPSF2*	1.00E-10
rs7155279 (chr14:92485881)	rs8007661 (chr14:92459958)	6.45E-01	18391950	Height	*TRIP11,ATXN3*	6.00E-10
rs7155279 (chr14:92485881)	rs7155279 (chr14:92485881)	1.00E+00	20881960	Height	*TRIP11*	1.00E-10
rs117068593 (chr14:93118229)	rs10498633 (chr14:92926952)	2.75E-05	24162737	Alzheimer's disease (late onset)	*SLC24A4, RIN3*	6.00E-09
rs117068593 (chr14:93118229)	rs10498635 (chr14:93103309)	9.39E-01	21623375	Paget's disease	*RIN3*	3.00E-11
rs117068593 (chr14:93118229)	rs754388 (chr14:93115410)	9.94E-01	24621683	Chronic obstructive pulmonary disease (moderate to severe)	*RIN3*	5.00E-09
rs117068593 (chr14:93118229)	rs754388 (chr14:93115410)	9.94E-01	24945404	Bone mineral density (paediatric, total body less head)	*RIN3*	3.00E-09
rs117068593 (chr14:93118229)	rs754388 (chr14:93115410)	9.94E-01	24945404	Bone mineral density (paediatric, lower limb)	*RIN3*	1.00E-10
rs113473882 (chr19:41124155)	rs28493229 (chr19:41224204)	6.63E-04	22081228	Kawasaki disease	*ITPKC*	2.00E-12
rs113473882 (chr19:41124155)	rs2233152 (chr19:41281016)	5.98E-04	22446962	Kawasaki disease	*NUMBL,MIA*	4.00E-10
rs113473882 (chr19:41124155)	rs7937 (chr19:41302706)	5.19E-03	22080838	Chronic obstructive pulmonary disease	*RAB4B, EGLN2, CYP2A6*	3.00E-09

Supplementary Table 6 List of 49 lung function signals detected to date

Variants associated with FEV_1, FEV_1/FVC or FVC in lung function GWAS to date are presented. The study in which each variant was firstly identified is indicated ("Identified in"), and the results presented ("Results from") correspond to the study with largest sample size available for the previously discovered variants and to the current study for the novel variants. Effect sizes correspond to inverse normally transformed lung function traits in all instances except for those FVC results extracted from Loth 2014[4], which correspond to ml. Previous studies referred to in the table are: Wilk_2009[1], Repapi_2010[5], Hancock_2010[2], Soler_Artigas_2011[3] and Loth_2014[4].

rs number (chr: position)	Gene (closest)	Measure	Identified in	Results from	Alleles	Coded allele	Coded allele freq	Beta	SE	Pval
rs2284746 (chr1:17306675)	*MFAP2*	FEV1/FVC	Soler_Artigas_2011	Soler_Artigas_2011	C/G	G	0.58	-0.040	0.005	7.50E-16
rs6681426 (chr1:150586971)	*ENSA*	FEV1	current	current	A/G	G	0.36	0.029	0.005	4.35E-09
rs993925 (chr1:218860068)	*TGFB2*	FEV1/FVC	Soler_Artigas_2011	Soler_Artigas_2011	C/T	T	0.42	0.034	0.006	1.16E-08
rs201204531 (chr1:219963090)	*RNU5F-1*	FEV1/FVC	current	current	ATG/A	A	0.41	-0.031	0.005	2.68E-10
rs61067109 (chr2:18292452)	*KCNS3*	FEV1/FVC	current	current	A/G	G	0.77	-0.045	0.006	1.40E-15
rs1430193 (chr2:56120853)	*EFEMP1*	FVC	Loth_2014	Loth_2014	A/T	T	0.37	-21.125	2.999	1.86E-12
rs2571445 (chr2:218683154)	*TNS1*	FEV1	Repapi_2010	Soler_Artigas_2011	A/G	G	0.62	0.047	0.007	9.83E-11
rs12477314 (chr2:239877148)	*HDAC4*	FEV1/FVC	Soler_Artigas_2011	Soler_Artigas_2011	C/T	T	0.13	0.041	0.006	1.68E-12
rs1529672 (chr3:25520582)	*RARB*	FEV1/FVC	Soler_Artigas_2011	Soler_Artigas_2011	A/C	C	0.86	-0.048	0.006	3.97E-14
rs6441207 (chr3:158282459)	*RP11-538P18.2*	FVC	current	current	T/C	C	0.59	0.036	0.005	1.27E-13
rs1344555 (chr3:169300219)	*MECOM*	FEV1	Soler_Artigas_2011	Soler_Artigas_2011	C/T	T	0.17	-0.034	0.006	2.65E-08
rs2045517 (chr4:89870964)	*FAM13A*	FEV1/FVC	Hancock_2010	Soler_Artigas_2011	C/T	T	0.36	-0.047	0.007	2.00E-11
rs10516526 (chr4:106688904)	*GSTCD*	FEV1	Repapi_2010 & Hancock_2010	Soler_Artigas_2011	A/G	G	0.08	0.108	0.014	4.75E-14
rs6856422 (chr4:106841962)	*NPNT*	FEV1/FVC	current	current	T/G	G	0.53	-0.051	0.005	1.51E-23
rs11100860 (chr4:145479139)	*HHIP*	FEV1/FVC	Wilk_2009	Soler_Artigas_2011	A/G	G	0.43	0.064	0.007	6.81E-20
rs153916 (chr5:95036700)	*SPATA9*	FEV1/FVC	Soler_Artigas_2011	Soler_Artigas_2011	C/T	T	0.52	-0.031	0.005	2.12E-08
rs1985524 (chr5:147847788)	*HTR4*	FEV1	Repapi_2010	Soler_Artigas_2011	C/G	G	0.61	-0.048	0.007	3.06E-11
rs11134779 (chr5:156936766)	*ADAM19*	FEV1/FVC	Hancock_2010	Soler_Artigas_2011	A/G	G	0.31	-0.042	0.007	6.01E-09

rs number (chr: position)	Gene (closest)	Measure	Identified in	Results from	Alleles	Coded allele	Coded allele freq	Beta	SE	Pval
rs6923462 (chr6:7801112)	*BMP6*	FVC	Loth_2014	Loth_2014	C/T	T	0.84	30.883	4.288	5.89E-13
rs6903823 (chr6:28322296)	*ZKSCAN3*	FEV1	Soler_Artigas_2011	Soler_Artigas_2011	A/G	G	0.19	-0.037	0.006	2.18E-10
rs2857595 (chr6:31568469)	*NCR3*	FEV1/FVC	Soler_Artigas_2011	Soler_Artigas_2011	A/G	G	0.79	0.037	0.006	2.28E-10
rs2070600 (chr6:32151443)	*AGER*	FEV1/FVC	Repapi_2010 & Hancock_2010	Soler_Artigas_2011	C/T	T	0.05	0.126	0.016	9.07E-15
rs2798641 (chr6:109268050)	*ARMC2*	FEV1/FVC	Soler_Artigas_2011	Soler_Artigas_2011	C/T	T	0.13	-0.041	0.007	8.35E-09
rs148274477 (chr6:142838173)	*GPR126*	FEV1/FVC	current	current	T/C	C	0.98	-0.162	0.015	9.58E-26
rs262129 (chr6:142853144)	*LOC153910*	FEV1/FVC	Hancock_2010	Soler_Artigas_2011	A/G	G	0.26	0.056	0.008	2.91E-13
rs16909859 (chr9:98204792)	*PTCH1*	FEV1/FVC	Soler_Artigas_2011	Soler_Artigas_2011	A/G	G	0.89	0.080	0.013	7.45E-10
rs16909898 (chr9:98231008)	*PTCH1*	FEV1/FVC	Hancock_2010	Soler_Artigas_2011	A/G	G	0.85	-0.072	0.012	3.94E-09
rs34886460 (chr9:119359372)	*ASTN2*	FEV1/FVC	current	current	TA/T	T	0.53	0.031	0.005	4.72E-11
rs2274116 (chr9:139094805)	*LHX3*	FVC	current	current	T/C	C	0.67	0.039	0.005	5.55E-14
rs7068966 (chr10:12277992)	*CDC123*	FEV1/FVC	Soler_Artigas_2011	Soler_Artigas_2011	C/T	T	0.43	0.033	0.005	6.13E-13
rs11001819 (chr10:78315224)	*C10orf11*	FEV1	Soler_Artigas_2011	Soler_Artigas_2011	A/G	G	0.50	-0.029	0.004	2.98E-12
rs4237643 (chr11:43648368)	*HSD17B12*	FVC	Loth_2014	Loth_2014	T/G	T	0.31	-16.666	3.023	3.53E-08
rs2863171 (chr11:45250732)	*PRDM11*	FVC	Loth_2014	Loth_2014	A/C	C	0.16	23.924	3.906	8.97E-10
rs11383346 (chr12:28283187)	*CCDC91*	FVC	current	current	AT/A	A	0.41	-0.042	0.005	9.52E-18
rs11172113 (chr12:57527283)	*LRP1*	FEV1/FVC	Soler_Artigas_2011	Soler_Artigas_2011	C/T	T	0.66	-0.032	0.006	1.24E-08
rs1036429 (chr12:96271428)	*CCDC38*	FEV1/FVC	Soler_Artigas_2011	Soler_Artigas_2011	C/T	T	0.25	0.038	0.006	2.30E-11
rs10850377 (chr12:115201436)	*TBX3*	FEV1	current	current	A/G	G	0.67	-0.035	0.005	2.50E-12
rs7155279 (chr14:92485881)	*TRIP11*	FEV1	current	current	T/G	G	0.64	-0.030	0.005	1.41E-09
rs117068593 (chr14:93118229)	*RIN3*	FEV1	current	current	T/C	C	0.82	-0.035	0.006	2.25E-08
rs8033889 (chr15:71680080)	*THSD4*	FEV1/FVC	Repapi_2010 & Hancock_2010	Soler_Artigas_2011	G/T	T	0.14	-0.072	0.008	2.03E-17
rs12149828 (chr16:10706328)	*EMP2*	FEV1/FVC	current	current	A/G	G	0.83	0.040	0.007	7.65E-10
rs12447804 (chr16:58075282)	*MMP15*	FEV1/FVC	Soler_Artigas_2011	Soler_Artigas_2011	C/T	T	0.24	-0.038	0.007	3.59E-08
rs2865531 (chr16:75390316)	*CFDP1*	FEV1/FVC	Soler_Artigas_2011	Soler_Artigas_2011	A/T	T	0.47	0.031	0.005	1.77E-11
rs1079572 (chr16:78187138)	*WWOX*	FVC	Loth_2014	Loth_2014	G/A	G	0.42	16.258	2.837	9.95E-09
rs6501431 (chr17:68976415)	*KCNJ2*	FVC	Loth_2014	Loth_2014	C/T	T	0.80	23.053	3.884	2.94E-09
rs113473882 (chr19:41124155)	*LTBP4*	FEV1/FVC	current	current	C/T	T	0.98	-0.153	0.021	9.95E-13
rs9978142 (chr21:35652239)	*KCNE2*	FEV1/FVC	Soler_Artigas_2011	Soler_Artigas_2011	A/T	T	0.22	-0.043	0.008	2.65E-08

rs number (chr: position)	Gene (closest)	Measure	Identified in	Results from	Alleles	Coded allele	Coded allele freq	Beta	SE	Pval
rs134041 (chr22:28056338)	*MN1*	FEV1	current	current	C/T	T	0.43	-0.028	0.005	3.03E-09
rs7050036 (chrX:15964845)	*AP1S2*	FEV1/FVC	current	current	A/T	T	0.38	-0.023	0.004	4.14E-08

Supplementary Table 7 Proportion of variance explained

Stage 1 results for the 33 previously discovered signals[1-5] and the 16 novel signals are shown with the proportion of the variance of the lung function traits that they explain (r^2), separately for FEV_1, FEV_1/FVC and FVC. Additive polygenic variance is estimated assuming a heritability for lung function of 40%[103-105] . Chr., chromosome; freq., frequency.

a. FEV_1

rs number (chr: position)	Closest gene	Coded allele	Non coded allele	Coded allele freq.	Beta (SE)	P	N	r^2 (%)
**Known**								
rs2284746 (chr1:17306675)	*MFAP2*	C	G	0.50	-0.011 (0.008)	1.40E-01	36224	0.006
rs993925 (chr1:218860068)	*TGFB2*	C	T	0.66	-0.016 (0.008)	5.32E-02	35599	0.010
rs1430193 (chr2:56120853)	*EFEMP1*	A	T	0.61	0.02 (0.008)	1.42E-02	35824	0.017
rs2571445 (chr2:218683154)	*TNS1*	A	G	0.40	-0.034 (0.008)	8.56E-06	37553	0.053
rs12477314 (chr2:239877148)	*HDAC4*	C	T	0.80	-0.032 (0.009)	5.28E-04	37932	0.032
rs1529672 (chr3:25520582)	*RARB*	C	A	0.83	-0.025 (0.01)	1.17E-02	36309	0.018
rs1344555 (chr3:169300219)	*MECOM*	C	T	0.79	0.036 (0.01)	1.62E-04	35867	0.040
rs2045517 (chr4:89870964)	*FAM13A*	C	T	0.59	0 (0.008)	9.52E-01	38095	0.000
rs10516526 (chr4:106688904)	*GSTCD*	A	G	0.94	-0.100 (0.016)	1.52E-10	37284	0.110
rs11100860 (chr4:145479139)	*HHIP*	A	G	0.58	-0.028 (0.008)	1.96E-04	38063	0.036
rs153916 (chr5:95036700)	*SPATA9*	C	T	0.44	-0.004 (0.008)	5.90E-01	37448	0.001
rs1985524 (chr5:147847788)	*HTR4*	G	C	0.54	-0.045 (0.008)	3.11E-09	37265	0.094
rs11134779 (chr5:156936766)	*ADAM19*	A	G	0.66	0.029 (0.008)	1.85E-04	38090	0.037
rs6923462 (chr6:7801112)	*BMP6*	T	C	0.84	0.037 (0.01)	3.00E-04	37005	0.035
rs6903823 (chr6:28322296)	*ZKSCAN3*	A	G	0.78	0.035 (0.01)	3.71E-04	37973	0.033
rs114327456 (chr6:31568469)	*NCR3*	G	A	0.78	0.036 (0.01)	3.22E-04	34997	0.037
rs114177847 (chr6:32151443)	*AGER*	C	T	0.95	-0.03 (0.021)	1.63E-01	25796	0.008
rs2798641 (chr6:109268050)	*ARMC2*	C	T	0.82	0.026 (0.01)	8.57E-03	37706	0.018
rs262129 (chr6:142853144)	*LOC153910*	A	G	0.70	-0.01 (0.008)	2.24E-01	37585	0.004
rs16909859 (chr9:98204792)	*PTCH1*	G	A	0.92	-0.007 (0.014)	6.06E-01	33748	0.001
rs16909898 (chr9:98231008)	*PTCH1*	A	G	0.90	-0.031 (0.013)	1.53E-02	36168	0.016
rs7068966 (chr10:12277992)	*CDC123*	C	T	0.48	-0.037 (0.008)	9.53E-07	37832	0.063
rs11001819 (chr10:78315224)	*C10orf11*	G	A	0.52	-0.027 (0.008)	3.44E-04	38018	0.034
rs4237643 (chr11:43648368)	*HSD17B12*	T	G	0.30	-0.026 (0.008)	1.27E-03	38018	0.027
rs2863171 (chr11:45250732)	*PRDM11*	A	C	0.84	-0.032 (0.01)	2.11E-03	38138	0.025
rs11172113 (chr12:57527283)	*LRP1*	T	C	0.60	-0.011 (0.008)	1.46E-01	37724	0.006
rs1036429 (chr12:96271428)	*CCDC38*	T	C	0.19	0.011 (0.01)	2.29E-01	38123	0.004
rs8033889 (chr15:71680080)	*THSD4*	G	T	0.78	0.032 (0.009)	3.29E-04	38080	0.034
rs12447804 (chr16:58075282)	*MMP15*	C	T	0.77	0.008 (0.009)	3.89E-01	37782	0.002
rs2865531 (chr16:75390316)	*CFDP1*	T	A	0.41	0.025 (0.008)	1.24E-03	37942	0.027
rs1079572 (chr16:78187138)	*WWOX*	G	A	0.43	0.016 (0.008)	3.41E-02	37809	0.012
rs6501431 (chr17:68976415)	*KCNJ2*	C	T	0.21	-0.021 (0.009)	2.35E-02	37869	0.014
rs9978142 (chr21:35652239)	*KCNE2*	A	T	0.85	0.010 (0.011)	3.46E-01	37633	0.002
**Total r^2 for known variants**								**0.855**
**Total additive polygenic variance for known variants**								**2.138**
**New**								
rs6681426 (chr1:150586971)	*ENSA*	G	A	0.36	0.042 (0.008)	1.07E-07	37944	0.074
rs201204531 (chr1:219963090)	*RNU5F-1*	A	ATG	0.41	-0.007 (0.008)	3.75E-01	34887	0.002

rs number (chr: position)	Closest gene	Coded allele	Non coded allele	Coded allele freq.	Beta (SE)	P	N	r^2 (%)
rs61067109 (chr2:18292452)	*KCNS3*	G	A	0.77	-0.039 (0.009)	1.83E-05	37433	0.049
rs6441207 (chr3:158282459)	*AK097794*	C	T	0.59	0.029 (0.008)	2.07E-04	36426	0.038
rs6856422 (chr4:106841962)	*NPNT*	G	T	0.53	-0.045 (0.008)	6.22E-08	31460	0.093
rs148274477 (chr6:142838173)	*LOC153910*	C	T	0.98	0.010 (0.027)	7.07E-01	30318	0.000
rs34886460 (chr9:119359372)	*ASTN2*	T	TA	0.53	0.023 (0.008)	2.85E-03	37583	0.024
rs2274116 (chr9:139094805)	*LHX3*	C	T	0.67	0.029 (0.009)	6.27E-04	32226	0.036
rs11383346 (chr12:28283187)	*CCDC91*	A	AT	0.41	-0.039 (0.008)	3.41E-07	37510	0.069
rs10850377 (chr12:115201436)	*TBX3*	G	A	0.67	-0.047 (0.008)	6.68E-09	37268	0.090
rs7155279 (chr14:92485881)	*TRIP11*	G	T	0.64	-0.041 (0.008)	1.39E-07	37691	0.074
rs117068593 (chr14:93118229)	*RIN3*	C	T	0.82	-0.048 (0.01)	2.72E-06	34496	0.064
rs12149828 (chr16:10706328)	*TEKT5*	G	A	0.83	0.037 (0.01)	3.38E-04	34022	0.038
rs113473882 (chr19:41124155)	*LTBP4*	T	C	0.98	-0.029 (0.033)	3.78E-01	32220	0.002
rs134041 (chr22:28056338)	*MN1*	T	C	0.43	-0.045 (0.008)	4.19E-09	37669	0.092
rs7050036 (chrX:15964845)	*AP1S2*	T	A	0.38	-0.014 (0.007)	4.41E-02	32292	0.013
**Total r2 for new variants**								0.758
**Total additive polygenic variance for new variants**								1.896
**Total r^2 for known and new variants**								1.614
**Total additive polygenic variance for known and new variants**								4.034

b. FEV_1/FVC

rs number (chr: position)	Closest gene	Coded allele	Non coded allele	Coded allele freq.	Beta (SE)	P	N	r^2 (%)
**Known**								
rs2284746 (chr1:17306675)	*MFAP2*	C	G	0.50	0.043 (0.008)	2.19E-08	36208	0.086
rs993925 (chr1:218860068)	*TGFB2*	C	T	0.66	-0.021 (0.008)	1.04E-02	35582	0.018
rs1430193 (chr2:56120853)	*EFEMP1*	A	T	0.61	-0.012 (0.008)	1.25E-01	35808	0.007
rs2571445 (chr2:218683154)	*TNS1*	A	G	0.40	-0.012 (0.008)	1.11E-01	37537	0.007
rs12477314 (chr2:239877148)	*HDAC4*	C	T	0.80	-0.052 (0.009)	3.59E-08	37915	0.080
rs1529672 (chr3:25520582)	*RARB*	C	A	0.83	-0.046 (0.01)	6.43E-06	36292	0.056
rs1344555 (chr3:169300219)	*MECOM*	C	T	0.79	0.021 (0.01)	2.81E-02	35856	0.013
rs2045517 (chr4:89870964)	*FAM13A*	C	T	0.59	0.035 (0.008)	4.78E-06	38078	0.055
rs10516526 (chr4:106688904)	*GSTCD*	A	G	0.94	-0.051 (0.016)	1.08E-03	37267	0.029
rs11100860 (chr4:145479139)	*HHIP*	A	G	0.58	-0.054 (0.008)	8.60E-13	38046	0.134
rs153916 (chr5:95036700)	*SPATA9*	C	T	0.44	0.032 (0.008)	3.55E-05	37429	0.046
rs1985524 (chr5:147847788)	*HTR4*	G	C	0.54	-0.039 (0.008)	2.50E-07	37246	0.071
rs11134779 (chr5:156936766)	*ADAM19*	A	G	0.66	0.036 (0.008)	4.06E-06	38073	0.056
rs6923462 (chr6:7801112)	*BMP6*	T	C	0.84	0 (0.01)	9.92E-01	36987	0.000
rs6903823 (chr6:28322296)	*ZKSCAN3*	A	G	0.78	0.013 (0.01)	1.78E-01	37958	0.005
rs114327456 (chr6:31568469)	*NCR3*	G	A	0.78	0.023 (0.01)	2.08E-02	34980	0.015
rs114177847 (chr6:32151443)	*AGER*	C	T	0.95	-0.118 (0.021)	2.08E-08	25785	0.122
rs2798641 (chr6:109268050)	*ARMC2*	C	T	0.82	0.033 (0.01)	7.86E-04	37689	0.030
rs262129 (chr6:142853144)	*LOC153910*	A	G	0.70	-0.033 (0.008)	5.92E-05	37568	0.043
rs16909859 (chr9:98204792)	*PTCH1*	G	A	0.92	0.055 (0.014)	1.43E-04	33738	0.043
rs16909898 (chr9:98231008)	*PTCH1*	A	G	0.90	0.052 (0.013)	5.48E-05	36153	0.045
rs7068966 (chr10:12277992)	*CDC123*	C	T	0.48	-0.034 (0.008)	6.00E-06	37816	0.054
rs11001819 (chr10:78315224)	*C10orf11*	G	A	0.52	-0.018 (0.008)	2.00E-02	38002	0.014
rs4237643 (chr11:43648368)	*HSD17B12*	T	G	0.30	-0.004 (0.008)	6.64E-01	38001	0.000
rs2863171 (chr11:45250732)	*PRDM11*	A	C	0.84	-0.009 (0.01)	3.77E-01	38121	0.002
rs11172113 (chr12:57527283)	*LRP1*	T	C	0.60	-0.032 (0.008)	3.41E-05	37707	0.046
rs1036429 (chr12:96271428)	*CCDC38*	T	C	0.19	0.03 (0.01)	1.84E-03	38106	0.025
rs8033889 (chr15:71680080)	*THSD4*	G	T	0.78	0.071 (0.009)	4.31E-15	38063	0.162
rs12447804 (chr16:58075282)	*MMP15*	C	T	0.77	0.024 (0.009)	6.36E-03	37766	0.020
rs2865531 (chr16:75390316)	*CFDP1*	T	A	0.41	0.037 (0.008)	1.29E-06	37925	0.062
rs1079572 (chr16:78187138)	*WWOX*	G	A	0.43	-0.002 (0.008)	7.84E-01	37792	0.000
rs6501431 (chr17:68976415)	*KCNJ2*	C	T	0.21	0.014 (0.009)	1.24E-01	37852	0.006
rs9978142 (chr21:35652239)	*KCNE2*	A	T	0.85	0.028 (0.011)	7.58E-03	37623	0.019
**Total r^2 for known variants**								1.372
**Total additive polygenic variance for known variants**								3.430
**New**								
rs6681426 (chr1:150586971)	*ENSA*	G	A	0.36	0.014 (0.008)	7.47E-02	37927	0.008
rs201204531 (chr1:219963090)	*RNU5F-1*	A	ATG	0.41	-0.038 (0.008)	1.98E-06	34866	0.065

rs number (chr: position)	Closest gene	Coded allele	Non coded allele	Coded allele freq.	Beta (SE)	P	N	r^2 (%)
rs61067109 (chr2:18292452)	*KCNS3*	G	A	0.77	-0.05 (0.009)	3.09E-08	37416	0.082
rs6441207 (chr3:158282459)	*AK097794*	C	T	0.59	-0.003 (0.008)	6.56E-01	36408	0.001
rs6856422 (chr4:106841962)	*NPNT*	G	T	0.53	-0.044 (0.008)	1.30E-07	31446	0.089
rs148274477 (chr6:142838173)	*LOC153910*	C	T	0.98	-0.161 (0.027)	2.68E-09	30398	0.116
rs34886460 (chr9:119359372)	*ASTN2*	T	TA	0.53	0.041 (0.008)	6.75E-08	37567	0.078
rs2274116 (chr9:139094805)	*LHX3*	C	T	0.67	-0.013 (0.009)	1.29E-01	32205	0.007
rs11383346 (chr12:28283187)	*CCDC91*	A	AT	0.41	-0.002 (0.008)	8.38E-01	37497	0.000
rs10850377 (chr12:115201436)	*TBX3*	G	A	0.67	-0.02 (0.008)	1.10E-02	37245	0.017
rs7155279 (chr14:92485881)	*TRIP11*	G	T	0.64	-0.023 (0.008)	3.49E-03	37675	0.023
rs117068593 (chr14:93118229)	*RIN3*	C	T	0.82	0.008 (0.01)	4.23E-01	34476	0.002
rs12149828 (chr16:10706328)	*TEKT5*	G	A	0.83	0.049 (0.01)	3.16E-06	33999	0.064
rs113473882 (chr19:41124155)	*LTBP4*	T	C	0.98	-0.174 (0.033)	1.48E-07	32207	0.086
rs134041 (chr22:28056338)	*MN1*	T	C	0.43	-0.025 (0.008)	1.06E-03	37653	0.028
rs7050036 (chrX:15964845)	*AP1S2*	T	A	0.38	-0.041 (0.007)	4.37E-09	32285	0.107
**Total r^2 for new variants**								0.772
**Total additive polygenic variance for new variants**								1.931
**Total r^2 for known and new variants**								2.144
**Total additive polygenic variance for known and new variants**								5.361

c. FVC

rs number (chr: position)	Closest gene	Coded allele	Non coded allele	Coded allele freq.	Beta (SE)	P	N	r^2 (%)
**Known**								
rs2284746 (chr1:17306675)	*MFAP2*	C	G	0.50	-0.039 (0.008)	3.61E-07	35967	0.072
rs993925 (chr1:218860068)	*TGFB2*	C	T	0.66	-0.006 (0.008)	4.86E-01	35348	0.001
rs1430193 (chr2:56120853)	*EFEMP1*	A	T	0.61	0.032 (0.008)	9.67E-05	35571	0.043
rs2571445 (chr2:218683154)	*TNS1*	A	G	0.40	-0.031 (0.008)	6.97E-05	37297	0.042
rs12477314 (chr2:239877148)	*HDAC4*	C	T	0.80	0.002 (0.009)	8.15E-01	37678	0.000
rs1529672 (chr3:25520582)	*RARB*	C	A	0.83	0.003 (0.01)	7.58E-01	36053	0.000
rs1344555 (chr3:169300219)	*MECOM*	C	T	0.79	0.023 (0.01)	1.46E-02	35627	0.017
rs2045517 (chr4:89870964)	*FAM13A*	C	T	0.59	-0.023 (0.008)	2.66E-03	37839	0.024
rs10516526 (chr4:106688904)	*GSTCD*	A	G	0.94	-0.076 (0.016)	1.54E-06	37028	0.062
rs11100860 (chr4:145479139)	*HHIP*	A	G	0.58	0.009 (0.008)	2.30E-01	37807	0.004
rs153916 (chr5:95036700)	*SPATA9*	C	T	0.44	-0.029 (0.008)	1.62E-04	37195	0.038
rs1985524 (chr5:147847788)	*HTR4*	G	C	0.54	-0.02 (0.008)	1.13E-02	37010	0.017
rs11134779 (chr5:156936766)	*ADAM19*	A	G	0.66	0.007 (0.008)	3.60E-01	37834	0.002
rs6923462 (chr6:7801112)	*BMP6*	T	C	0.84	0.048 (0.01)	3.52E-06	36750	0.059
rs6903823 (chr6:28322296)	*ZKSCAN3*	A	G	0.78	0.03 (0.01)	2.55E-03	37719	0.024
rs114327456 (chr6:31568469)	*NCR3*	G	A	0.78	0.025 (0.01)	1.47E-02	34741	0.017
rs114177847 (chr6:32151443)	*AGER*	C	T	0.95	0.048 (0.022)	2.79E-02	25540	0.019
rs2798641 (chr6:109268050)	*ARMC2*	C	T	0.82	0.007 (0.01)	4.81E-01	37450	0.001
rs262129 (chr6:142853144)	*LOC153910*	A	G	0.70	0.005 (0.008)	5.48E-01	37330	0.001
rs16909859 (chr9:98204792)	*PTCH1*	G	A	0.92	-0.048 (0.014)	9.58E-04	33507	0.033
rs16909898 (chr9:98231008)	*PTCH1*	A	G	0.90	-0.072 (0.013)	2.87E-08	35924	0.086
rs7068966 (chr10:12277992)	*CDC123*	C	T	0.48	-0.015 (0.008)	5.33E-02	37577	0.010
rs11001819 (chr10:78315224)	*C10orf11*	G	A	0.52	-0.016 (0.008)	3.26E-02	37763	0.012
rs4237643 (chr11:43648368)	*HSD17B12*	T	G	0.30	-0.028 (0.008)	7.14E-04	37763	0.030
rs2863171 (chr11:45250732)	*PRDM11*	A	C	0.84	-0.031 (0.01)	3.23E-03	37882	0.023
rs11172113 (chr12:57527283)	*LRP1*	T	C	0.60	0.013 (0.008)	8.95E-02	37468	0.008
rs1036429 (chr12:96271428)	*CCDC38*	T	C	0.19	-0.006 (0.01)	5.48E-01	37867	0.001
rs8033889 (chr15:71680080)	*THSD4*	G	T	0.78	-0.011 (0.009)	2.25E-01	37824	0.004
rs12447804 (chr16:58075282)	*MMP15*	C	T	0.77	-0.005 (0.009)	6.06E-01	37525	0.001
rs2865531 (chr16:75390316)	*CFDP1*	T	A	0.41	0.004 (0.008)	6.11E-01	37685	0.001
rs1079572 (chr16:78187138)	*WWOX*	G	A	0.43	0.017 (0.008)	2.21E-02	37553	0.014
rs6501431 (chr17:68976415)	*KCNJ2*	C	T	0.21	-0.033 (0.009)	3.74E-04	37612	0.034
rs9978142 (chr21:35652239)	*KCNE2*	A	T	0.85	-0.011 (0.011)	2.81E-01	37382	0.003
**Total r^2 for known variants**								0.703
**Total additive polygenic variance for known variants**								1.757
**New**								
rs6681426 (chr1:150586971)	*ENSA*	G	A	0.36	0.036 (0.008)	4.74E-06	37688	0.056
rs201204531 (chr1:219963090)	*RNU5F-1*	A	ATG	0.41	0.011 (0.008)	1.53E-01	34632	0.006

rs number (chr: position)	Closest gene	Coded allele	Non coded allele	Coded allele freq.	Beta (SE)	P	N	r^2 (%)
rs61067109 (chr2:18292452)	*KCNS3*	G	A	0.77	-0.009 (0.009)	3.05E-01	37177	0.003
rs6441207 (chr3:158282459)	*AK097794*	C	T	0.59	0.036 (0.008)	4.54E-06	36173	0.058
rs6856422 (chr4:106841962)	*NPNT*	G	T	0.53	-0.02 (0.008)	1.54E-02	31226	0.019
rs148274477 (chr6:142838173)	*LOC153910*	C	T	0.98	0.114 (0.027)	3.07E-05	30110	0.058
rs34886460 (chr9:119359372)	*ASTN2*	T	TA	0.53	0 (0.008)	9.72E-01	37330	0.000
rs2274116 (chr9:139094805)	*LHX3*	C	T	0.67	0.041 (0.009)	2.68E-06	32004	0.069
rs11383346 (chr12:28283187)	*CCDC91*	A	AT	0.41	-0.043 (0.008)	3.83E-08	37254	0.081
rs10850377 (chr12:115201436)	*TBX3*	G	A	0.66	-0.033 (0.008)	3.60E-05	37015	0.046
rs7155279 (chr14:92485881)	*TRIP11*	G	T	0.64	-0.031 (0.008)	8.62E-05	37439	0.041
rs117068593 (chr14:93118229)	*RIN3*	C	T	0.82	-0.054 (0.01)	1.74E-07	34254	0.080
rs12149828 (chr16:10706328)	*TEKT5*	G	A	0.83	0.005 (0.01)	6.12E-01	33774	0.001
rs113473882 (chr19:41124155)	*LTBP4*	T	C	0.98	0.073 (0.033)	2.88E-02	31982	0.015
rs134041 (chr22:28056338)	*MN1*	T	C	0.43	-0.031 (0.008)	5.44E-05	37415	0.044
rs7050036 (chrX:15964845)	*AP1S2*	T	A	0.38	0.01 (0.007)	1.47E-01	32283	0.007
**Total r^2 for new variants**								0.582
**Total additive polygenic variance for new variants**								1.454
**Total r^2 for known and new variants**								1.284
**Total additive polygenic variance for known and new variants**								3.211

Supplementary Table 8 Results for 1000 Genomes sentinel variants with lower allele frequency than HapMap sentinel variants in two known loci

Shown are stage 1 and UK BiLEVE results for sentinel variants in previously reported lung function regions [2-5] which had minor allele frequency (<10%) more than two-fold lower than the HapMap sentinel variants in these regions, with the exception of a signal in *GPR126* (rs148274477) whose results are presented in **Table 1**. “GWAS sentinel” and “GWAS gene” present the sentinel SNP reported in a previous GWAS [3] and the closest gene to sentinel SNP respectively. Chr., chromosome.

**GWAS sentinel**	**GWAS gene**	**rs number (chr:position)**	**R2 with sentinel**	**Coded/ other allele**	**Stage 1**					**UK BiLEVE**				
					**MAF**	**Neff**	**Beta**	**SE**	**P**	**MAF**	**Neff**	**Beta**	**SE**	**P**
rs993925	*TGFB2*	rs147187942 (chr1:218773216)	1.73E-02	T/A	0.090	31881	-0.047	0.014	8.72E-04	0.085	46263	-0.032	0.012	5.72E-03
rs12447804	*MMP15*	rs150232756 (chr16:57691218)	9.78E-06	C/T	0.005	28940	0.203	0.057	3.65E-04	0.005	42760	-0.057	0.051	2.67E-01

Supplementary Table 9 MAGENTA results

MAGENTA results both for the main analysis ("main") and the sensitivity analysis after removing the HLA region ("sensitivity (no HLA)") for pathways that met a Bonferroni threshold corrected for the number of pathways tested per database and had an FDR<5% in the main analysis are presented.

**Measure**	**Analysis**	**Database**	**Bonferroni corrected threhold**	**Pathway**	**P**	**FDR**
FVC	main	Ingenuity	6.17E-04	PDGF.Signaling	2.00E-04	0.0028
FVC	main	PANTHER BIOLOGICAL PROCESS	2.30E-04	Chromatin packaging and remodeling	1.82E-04	0.0357
FVC	sensitivity (no HLA)	Ingenuity	6.17E-04	PDGF Signaling	6.60E-05	0.0023
FVC	sensitivity (no HLA)	PANTHER BIOLOGICAL PROCESS	2.30E-04	Chromatin packaging and remodeling	4.00E-04	0.0781

Supplementary Notes:

Supplementary Note 1: Heterogeneity test

A Chi-square test for heterogeneity was undertaken and I^2 calculated for the stage 1 results across studies for the 16 novel sentinel variants. Results are presented below; none of the results meets a Bonferroni correted threshold for 16 tests (P<3.13x10^{-3}).

rs number (chr:position)	Gene (function)	Measure	X^2	P	Degrees of freedom	I^2
rs6681426 (chr1:150586971)	*MCL1* (dist=34757), *ENSA* (dist=7628)	FEV_1	12.37	7.18E-01	16	0.00
rs201204531 (chr1:219963090)	*LYPLAL1* (dist=576883), *RNU5F-1* (dist=83529)	FEV_1/FVC	12.51	5.65E-01	14	0.00
rs61067109 (chr2:18292452)	*KCNS3* (dist=178227), *NT5C1B* (dist=443537)	FEV_1/FVC	17.30	3.66E-01	16	7.54
rs6441207 (chr3:158282459)	*AK097794* (ncRNA_intronic)	FVC	11.69	7.02E-01	15	0.00
rs6856422 (chr4:106841962)	*NPNT* (intronic)	FEV_1/FVC	9.62	8.43E-01	15	0.00
rs148274477 (chr6:142838173)	*GPR126* (dist=70770), *LOC153910* (dist=9419)	FEV_1/FVC	25.12	6.77E-02	16	36.31
rs34886460 (chr9:119359372)	*ASTN2* (intronic)	FEV_1/FVC	9.20	9.05E-01	16	0.00
rs2274116 (chr9:139094805)	*LHX3* (exonic)	FVC	25.52	4.34E-02	15	41.22
rs11383346 (chr12:28283187)	*PTHLH* (dist=158271), *CCDC91* (dist=126946)	FVC	18.35	3.04E-01	16	12.82
rs10850377 (chr12:115201436)	*TBX3* (dist=79467), *MED13L* (dist=1194945)	FEV_1	25.10	6.81E-02	16	36.26
rs7155279 (chr14:92485881)	*TRIP11* (intronic)	FEV_1	12.43	7.14E-01	16	0.00
rs117068593 (chr14:93118229)	*RIN3* (exonic)	FEV_1	16.86	3.27E-01	15	11.02
rs12149828 (chr16:10706328)	*EMP2* (dist=31789), *TEKT5*(dist=15033)	FEV_1/FVC	19.17	2.06E-01	15	21.75
rs113473882 (chr19:41124155)	*LTBP4* (intronic)	FEV_1/FVC	15.91	4.59E-01	16	0.00
rs134041 (chr22:28056338)	*MIAT* (dist=983898), *MN1* (dist=87927)	FEV_1	13.89	6.07E-01	16	0.00
rs7050036 (chrX:15964845)	*AP1S2* (dist=91708), *GRPR* (dist=176579)	FF	9.49	7.35E-01	13	0.00

Supplementary Note 2: Stepwise conditional analysis to identify multiple signals within each region

In order to identify independent signals within regions already associated with lung function, we undertook a stepwise conditional analysis using GCTA[20] for each of the previously discovered[1-5] and new regions. This method uses a stepwise procedure to select a subset of independent variants that when fitted together in the model each has a joint *P* value below a pre specified threshold. In order to be comprehensive we defined the threshold for inclusion in the model as joint *P* value < 10^{-3} and we defined regions as 1Mb either side of the sentinel variant; when two regions overlapped we merged them into one larger region.

We identified one additional independent (r^2 < 0.02 with sentinel variant) variant with joint *P* value < 10^{-5} in two regions, near the previously discovered signals in *ZKSCAN3* and *CCDC38* (see bold *P*-values in the table below). Models including the variants selected by the stepwise conditional analysis in these two regions were fitted using UK BiLEVE data and none of these two variants was significant.

Known sentinel rs number (chr:position)	Known GWAS gene symbol	rs number (chr:position)	Coded allele / other allele	R^2 with sentinel	Dataset	Unconditional results				Joint analysis			Correlation (r) with the next variant
						Coded allele frequency	Beta	SE	P	Beta	SE	P	
rs6903823 (chr6:28322296)	*ZKSCAN3*	rs73395396 (chr6:27479793)	C/T	0.02	stage 1	0.99	-0.046	0.045	3.13E-01	0.389	0.107	2.76E-04	0.92
					UK BiLEVE	0.99	0.055	0.032	8.78E-02	0.090	0.089	3.10E-01	0.93
		rs200955 (chr6:27839312)	T/C	0.02	stage 1	0.99	-0.123	0.042	3.68E-03	-0.477	0.100	**2.00E-06**	0.14
					UK BiLEVE	0.99	-0.044	0.032	1.75E-01	-0.065	0.090	4.67E-01	0.14
		rs1778483 (chr6:28240991)	T/G	0.66	stage 1	0.73	0.034	0.009	2.25E-04	0.036	0.009	8.64E-05	
					UK BiLEVE	0.68	0.037	0.007	6.71E-08	0.036	0.007	1.84E-07	
rs1036429 (chr12:96271428)	*CCDC38*	rs200233711 (chr12:95306163)	T/TG	0.00	stage 1	0.60	0.033	0.009	1.23E-04	0.038	0.009	**9.20E-06**	-0.05
					UK BiLEVE	0.59	0.013	0.007	5.37E-02	0.013	0.007	5.63E-02	-0.04
		rs66498225 (chr12:95330172)	C/T	0.00	stage 1	0.94	-0.052	0.016	9.66E-04	-0.056	0.016	4.11E-04	-0.06
					UK BiLEVE	0.93	-0.036	0.013	4.54E-03	-0.036	0.013	5.20E-03	-0.07
		rs2060304 (chr12:95486073)	A/G	0.00	stage 1	0.94	-0.049	0.016	1.58E-03	-0.065	0.016	4.12E-05	0.01
					UK BiLEVE	0.93	0.000	0.013	9.69E-01	-0.007	0.013	6.08E-01	0.00
		rs150198783 (chr12:96141778)	G/A	0.00	stage 1	0.99	0.154	0.037	3.11E-05	0.155	0.037	2.57E-05	0.02
					UK BiLEVE	0.99	0.020	0.043	6.38E-01	0.015	0.043	7.19E-01	0.05
		rs7134025 (chr12:96153846)	C/T	0.25	stage 1	0.15	0.045	0.011	2.43E-05	0.046	0.011	1.38E-05	0.01
					UK BiLEVE	0.14	0.023	0.009	1.60E-02	0.023	0.009	1.47E-02	0.03
		rs7959665 (chr12:97069873)	G/A	0.00	stage 1	0.02	-0.096	0.028	6.89E-04	-0.103	0.028	2.71E-04	
					UK BiLEVE	0.02	-0.026	0.023	2.45E-01	-0.028	0.023	2.13E-01	

Supplementary Note 3: Individual study descriptions

GWAS samples and phenotype measurement

This section describes study-specific characteristics that are not presented in the tables. All participants provided written informed consent and studies were approved by local Research Ethics Committees and/or Institutional Review boards.

Details of the **British 1958 Birth Cohort** biomedical follow-up have been previously reported [117]. Spirometry at age 44–45 years was done in the standing position without nose clips, using a Vitalograph handheld spirometer as previously described [118]. In the analysis, all readings with a best-test variation greater than 10% were excluded.

The **Busselton Health Study** (BHS) is a longitudinal survey of the town of Busselton in the south-western region of Western Australia that began in 1966. In 1994/1995 a cross-sectional community follow-up study was undertaken where blood was taken for DNA extraction. A sample of 1,168 European-ancestry individuals were genotyped using the Illumina 610-Quad BeadChip (BHS1), and subsequent genotyping was carried out on an independent group of 3,428 European-ancestry individuals using Illumina 660W-Quad (BHS2). Spirometric measures of forced expired volume in one second (FEV_1) and forced vital capacity (FVC) were assessed.

The CROATIA study was initiated to investigate the use of isolated rather than urban populations for the identification of genes associated with medically-relevant quantitative traits. Three cohorts have been recruited as part of the CROATIA study: **CROATIA-Vis**[119], **CROATIA-Korcula**[120] and

CROATIA-Split[121]. CROATIA-Vis was the first to be collected when 1,008 Croatians aged 18-93 recruited from the villages of Komiza and Vis on the Dalmatian island of Vis. Recruitment occurred from 2003 to 2004 with participants donating blood for DNA extraction and biochemical measurements as well as undergoing some anthropometric measurements and physiological tests to measure traits such as height, weight and blood pressure, and finally completing several questionnaires relating to general health, medical history, diet and lifestyle. CROATIA-Korcula was recruited from 2007 to 2008 from the town of Korcula and the villages of Lumbarda, Zrnovo and Racisce on the island of Korcula, Croatia with 969 adults aged 18-98 agreeing to participate. This study followed the same recruitment procedures as CROATIA-Vis and the same samples and tests were collected with a few additions to reflect the research interests and expertise in Edinburgh. Volunteers were recruited to be part of the CROATIA-Split cohort in 2009-2010 from the Dalmatian mainland city of Split. This is the main ferry port to the islands and is the second largest city in Croatia and the largest along the Dalmatian coast. 1,012 adults aged 18-85 were recruited using the same methodology and with the same samples collected as in CROATIA-Korcula. Ethical approval was obtained from appropriate regulatory bodies in both Scotland and Croatia and participants gave informed consent prior to joining the study.

The **EPIC Norfolk** GWA cohort includes 2,566 participants randomly selected from the EPIC-Norfolk Study, a population-based cohort study of 25,663 men and women of European descent aged 39-79 years recruited in Norfolk, UK between 1993 and 1997[122]. Respiratory function was assessed by spirometry[123,124]. Forced expiratory volume in 1 second (FEV_1) and forced vital capacity (FVC) were measured twice using a portable spirometer (Micro Medical, Rochester, United Kingdom). The higher values of the two readings for FEV_1 and FVC were used for the analyses.

The **Generation Scotland: Scottish Family Health Study** is a collaboration between the Scottish Universities and the NHS, funded by the Chief Scientist Office of the Scottish Government. GS:SFHS is a family-based genetic epidemiology cohort with DNA, other biological samples (serum, urine and cryopreserved whole blood) and socio-demographic and clinical data from ~24,000 volunteers, aged 18-98 years, in ~7,000 family groups. Participants were recruited across Scotland, with some family members from further afield, from 2006-2011. Most (87%) participants were born in Scotland and 96% in the UK or Ireland. The cohort profile has been published[125]. GS:SFHS operates under appropriate ethical approvals, and all participants gave written informed consent. Generation Scotland is a collaboration between the University Medical Schools and National Health Service in Aberdeen, Dundee, Edinburgh and Glasgow (UK).

The DNA archive established from the **Health 2000** Survey Cohort was used. Details of this study population and phenotyping procedures have been previously reported[126]. Genome-wide genotyping was available for 2124 individuals selected from the Health 2000 cohort as metabolic syndrome cases and their matched controls[127]. Spirometry was done in the standing position without nose clips, using a Vitalograph 2150 spirometer. In the analysis, the maximum permissible difference between the two highest FEV_1 and FVC values was 10%.

The KORA studies (Cooperative Health Research in the Region of Augsburg) are a series of independent population based studies from the general population living in the region of Augsburg, Southern Germany[128,129]. **KORA F4** including 3,080 individuals was conducted from 2006-2008 as a follow-up study to KORA S4 (1999-2001). Lung function tests were performed in a random subsample of subjects born between 1946 and 1965 (age range 41–63 years). Spirometry was performed in line with the ATS/ERS recommendations[130] using a pneumotachograph-type spirometer

(Masterscreen PC, CardinalHealth, Würzburg, Germany) before and after inhalation of 200µg salbutamol. The present study is based on maximum values of FEV_1 and FVC measured before bronchodilation. The spirometer was calibrated daily using a calibration pump (CardinalHealth, Würzburg, Germany), and additionally, an internal control was used to ensure constant instrumental conditions. For KORA F4 participants without spirometry measurements in 2006-2008, we used measurements from the KORA-Age time point conducted in 2008/09. KORA Age contains subjects from all KORA studies born until 1943 (aged 65-90 years)[131]. Spirometry was measured in 935 randomly selected participants. Conditions including the examiner were the same as in 2008/09 except that inhalation of salbutamol was not performed due to the high number of contraindications anticipated in this aged population.

The KORA studies (Cooperative Health Research in the Region of Augsburg) are a series of independent population based studies from the general population living in the region of Augsburg, Southern Germany[128,129]. The **KORA S3** study including 4,856 individuals was conducted in 1994/95. Spirometry was measured during a follow up in 1997/98 for all participants younger than 60 years who did not smoke or use inhalers one hour before the test. All spirometric tests were performed strictly adhering to the ECRHS protocol[132,133] using Biomedin Spirometers (Biomedin srl, Padova, Italy). Tests were accounted valid if at least two technically satisfactory manoeuvres could be obtained throughout a maximum of nine trials. FEV_1 and FVC were defined as the maximum value within all valid manoeuvers. For KORA S3 participants without spirometry measurements in 1997/98 we used measurements from the KORA-Age time point conducted in 2008/09. KORA Age contains subjects from all KORA studies born until 1943 (aged 65–90 years) [131]. Spirometry was measured in 935 randomly selected participants. Conditions including the examiner were the same as in KORA F4

(see below) except that inhalation of salbutamol was not performed due to the high number of contraindications anticipated in this aged population.

The **Lothian Birth Cohort 1936** consists of 1,091 relatively healthy individuals assessed on cognitive and medical traits at about 70 years of age. They were all born in 1936 and most took part in the Scottish Mental Survey of 1947. At baseline the sample of 548 men and 543 women had a mean age 69.6 years (s.d. = 0.8). They were all Caucasian, community-dwelling, and almost all lived in the Lothian region (Edinburgh city and surrounding area) of Scotland. A full description of participant recruitment and testing can be found elsewhere[134]. Genotyping was performed at the Wellcome Trust Clinical Research Facility, Edinburgh. Quality control measures were applied and 1,005 participants remained. Lung function assessing peak expiratory flow rate, forced expiratory volume in 1 second, and forced vital capacity (each the best of three), using a Micro Medical Spirometer was assessed, sitting down without nose clips, at age 70 years. The accuracy of the spirometer is ±3% (to ATS recommendations Standardisation of Spirometry 1994 update for flows and volumes).

The **Northern Finland Birth Cohort** (NFBC) study programme was initiated in the 1960s. The cohort of women and their offspring was established in the provinces of Oulu and Lapland and had an expected date of birth in 1966 comprising 12,231 children (NFBC1966, 15). The NFBC1966 had spirometry and other measurements done at the age of 31 years. In NFBC1966, we used a Vitalograph P-model spirometer (Vitalograph Ltd., Buckingham, UK), with a volumetric accuracy of ±2% or ±50 mL whichever was greater. The spirometer was calibrated regularly using a 1-Litre precision syringe. The spirometric manoeuvre was performed three times but was repeated if the coefficient of variation between two maximal readings was >4%.

The **Northern Sweden Population Health Study** (NSPHS) represents a cross-sectional study conducted in the communities of Karesuando (samples gathered in 2006) and Soppero (2009) in the subarctic region of the County of Norrbotten, Sweden. Spirometry was performed in sitting position without noseclips using a MicroMedicalSpida 5 spirometer (http://www. medisave.co.uk). Three consecutive 28 lung function measurements per participant were done and the maximum value per measured lung function parameter was used for further analysis. Relatedness was taken into account by applying the "polygenic" linear mixed effects model. Genome-wide association analysis was performed using a score test, a family-based association test[135] which uses the residuals and the variance-covariance matrix from the polygenic model and the SNP fixed effect coded under an additive model.

The **Orkney Complex Disease Study** (ORCADES) is an ongoing family-based, cross-sectional study in the isolated Scottish archipelago of Orkney. Spirometry was performed in the sitting position without nose clips, using a Spida handheld spirometer. Measurements were repeated once and the better reading was used for analysis.

The **SAPALDIA** cohort is a population-based multi-center study in eight geographic areas representing the range of environmental, meteorological and socio-demographic conditions in Switzerland[136,137]. It was initiated in 1991 (SAPALDIA 1) with a follow-up assessment in 2002 (SAPALDIA 2) and 2010 (SAPALDIA3). This study has specifically been designed to investigate longitudinally lung function, respiratory and cardiovascular health; to study and identify the

associations of these health indicators with individual long term exposure to air pollution, other toxic inhalants, life style and molecular factors.

The **Study of Health in West Pomerania** (SHIP) is a cross-sectional, population based survey in a region in the Northeast of Germany. Study details are given elsewhere[138,139]. The examinations were conducted using a bodyplethysmograph equipped with a pneumotachograph (VIASYS Healthcare, JAEGER, Hoechberg, Germany) which meets the American Thoracic Society (ATS) criteria[140]. The volume signal of the equipment was calibrated with a 3.0 litre syringe connected to the pneumotachograph in accordance with the manufacturer´s recommendations and at least once on each day´s testing. Barometric pressure, temperature and relative humidity were registered every morning. Calibration of reference gas and volume was examined under ATS-conditions (Ambient Temperature Pressure) and the integrated volumes were BTPS (Body Temperature Pressure Saturated) corrected[140,141]. Lung function variables were measured continuously throughout the baseline breathing and the forced manoeuvres using a VIASYS HEALTHCARE system (MasterScreen Body/Diff.). Spirometry flow volume loops were conducted in accordance with ATS recommendations 40 in a sitting position and with wearing noseclips. The participants performed at least three forced expiratory lung function manoeuvres in order to obtain a minimum of two acceptable and reproducible values 41. Immediate on-screen error codes indicating the major acceptability (includingstart, duration and end of test) and reproducibility criteria supported the attempt for standardised procedures. The procedure was continuously monitored by a physician. The best results for FVC, FEV_1, peak expiratory flow (PEF) and expiratory flow at 75%, 50%, 25% of FVC (MEF 75, MEF 50, MEF 25) were taken. The ratio of FEV_1 to FVC was calculated from the largest FEV_1 and FVC.

The **YFS** is a population-based follow up-study started in 1980[142]. The main aim of the YFS is to determine the contribution made by childhood lifestyle, biological and psychological measures to the risk of cardiovascular diseases in adulthood. In 1980, over 3,500 children and adolescents all around Finland participated in the baseline study. The follow-up studies have been conducted mainly with 3-year intervals. The latest 30-year follow-up study was conducted in 2010-2011 (ages 33-49 years) with 2,063 participants. The study was approved by the local ethics committees (University Hospitals of Helsinki, Turku, Tampere, Kuopio and Oulu) and was conducted following the guidelines of the Declaration of Helsinki. All participants gave their written informed consent.

Follow-up samples and phenotype measurement

The **Avon Longitudinal Study of Parents and their Children** (ALSPAC) is a population-based birth cohort study consisting initially of over 13,000 women and their children recruited in the county of Avon, UK in the early 1990s[143]. At 8–9 years of age, children's lung function was measured by spirometry (Vitalograph 2120, Maids Moreton, UK) according to American Thoracic Society criteria. Flow-volume curves were reviewed by a respiratory physician to ensure adherence to standards. GWA data and lung function measurements were available on 5,062 of the children from this study. Ethical approval for the study was obtained from the ALSPAC Ethics and Law Committee and the Local Research Ethics Committees. Please note that the study website contains details of all the data that is available through a fully searchable data dictionary (http://www.bris.ac.uk/alspac/researchers/data-access/data-dictionary).

Details of the methods of **ECRHS I** and **ECRHS II**, a multicentre international cohort study, have been published elsewhere[132,144]. Participants within the ECRHS were eligible for inclusion in this analysis if

they were identified by random sampling of those who fulfilled the following criteria 1) lived in centres that took part in genome-wide genotyping initiative under the auspices of GABRIEL[145] AND 2) were initially selected to take part in the ECRHS clinical measurements as part of the random sample (ie not specifically selected for inclusion because of any pre-existing disease). Participants were included in this analysis if they also provided a technically satisfactory forced expiratory manoeuvre, compliant with ATS spirometry criteria, at the time of the first survey (aged 20-48). Most centers used the BIOMEDIN water-sealed spirometer for lung function measures and all centers conducted manoeuvres in the sitting position with nose clips on. Further details are available in[146].

The **Prospective Investigation of the Vasculature in Uppsala Seniors** (PIVUS)[147] is a population-based study of the cardiovascular health in the elderly. The main purpose of PIVUS was to investigate the role The Prospective Investigation of the Vasculature in Uppsala Seniors (PIVUS) of endothelial function in cardiovascular risk. Mailed invitations were sent to subjects who lived in Uppsala, Sweden, within 2 months after their 70th birthday. The subjects were randomly selected from the community register. A total of 1,016 men and women participated in the baseline investigation (participation rate, 50.1%). Spirometry was performed in 901 subjects at baseline in accordance with American Thoracic Society recommendations (α spirometer; Vitalograph Ltd; Buckingham, UK). The best value from three recordings was used. The Ethics Committee of the University of Uppsala approved the study, and the participants gave their informed consent. Genotyping of all samples was undertaken using the Illumina Omni Express and CardioMetabochip. Genotypes were called using GENCALL. A total of 738,879 SNPs passed quality control (thresholds: call rate < 0.95, and call rate < 0.99 for MAF<5%; HWE $P < 10^{-6}$). SNPs with MAF<1% were removed from the imputation scaffold. Imputation was performed using IMPUTE up to CEU haplotypes from Phase II HapMap reference panel.

For this analysis, 3,023 participants (85% female and all of Caucasian ancestry) within the **TwinsUK** adult twin registry based at St. Thomas' Hospital in London were analyzed for whom both genotype and IOP information was available. Twins largely volunteered unaware of the eye studies interests at the time of enrolment and gave fully informed consent under a protocol reviewed by the St. Thomas' Hospital Local Research Ethics Committee. Subjects were genotyped in two different batches of approximately the same size, using two genotyping platforms from Illumina: 300K Duo for and HumanHap610-Quad arrays. Whole genome imputation of the genotypes was performed using HapMap2 (www.hapmap.org) haplotypes. Stringent quality control (QC) measures were implemented, including minimum genotyping success rate (>95%), Hardy-Weinberg equilibrium ($P>10^{-6}$), minimum MAF (>1%) and imputation quality score (>0.7). Subjects of non Caucasian ancestry were excluded from the analysis. Association was calculated for all SNPs using a score test as implemented in the software GEMMA[148] (http://www.xzlab.org/software/gemma-0.94.tar.gz), accounting for possible covariates as explained in the sections explaining the general methodology.

The **UK Biobank Lung Exome Variant Evaluation** (UK BiLEVE) project was the first genetic study in UK Biobank and is described in[149]. UK Biobank (http://www.ukbiobank.ac.uk/) contains data from 502,682 individuals (94% of self-reported European ancestry) with extensive health and life-style questionnaire data, physical measures (including spirometry) and DNA. Spirometry was undertaken using a Vitalograph Pneumotrac 6800. The participant was asked to record two to three blows (lasting for at least 6 seconds) within a period of about 6 minutes. The computer compared the reproducibility of the first two blows and, if acceptable (defined as a <5% difference in forced volume vital capacity (FVC) and Forced Expiratory Volume in 1 second (FEV_1), a third blow was not required. For the UK BiLEVE project, a sampling frame of 275,939 individuals was defined as those who were of European

ancestry and had spirometry measures which met ERS/ATS guidelines[130]. A total of 50,008 samples were selected from the extremes and middle of the distributions of percent predicted FEV_1, separately in never smokers and heavy smokers (20,005 individuals with low FEV_1, 19,997 with average FEV_1 and 10,006 with high FEV_1). DNA was extracted and genotyped with the custom-designed Affymetrix Axiom UK BiLEVE array. Following thorough variant and sample QC, 48,943 unrelated individuals remained for further analysis.

Supplementary Note 4: Investigators, contributions, funding and acknowledgments per study

SpiroMeta consortium

Analysis group: Ian P Hall (co-chair), Suzanne Miller, Nick Shrine, María Soler Artigas, David P Strachan, Martin D Tobin (co-chair), Louise V Wain.

Bioinformatics and functional assessment group: Ian P Hall, Abdul K Kheirallah, Erik Melén, Suzanne Miller, Ioanna Ntalla, Ian Sayers, María Soler Artigas, Martin D Tobin, Louise V Wain.

Writing group: Ian P Hall (co-chair), Suzanne Miller, María Soler Artigas, David P Strachan, Martin D Tobin (co-chair), Louise V Wain.

Funding & Acknowledgements:

The research undertaken by MDT, MSA and LVW was part-funded funded by the National Institute for Health Research (NIHR). The views expressed are those of the author(s) and not necessarily those of the NHS, the NIHR or the Department of Health. MDT holds a Medical Research Council Senior Clinical Fellowship (G0902313). This research used the ALICE High Performance Computing Facility at the University of Leicester. The Universities of Leicester and Nottingham acknowledge receipt of a Collaborative Research and Development grant from the Healthcare and Bioscience iNet, a project funded by the East Midlands Development Agency (EMDA), part-financed by the European Regional Development Fund and delivered by Medilink East Midlands. IPH holds a Medical Research Council programme grant (G1000861).

GWAS studies

B58C

Investigators: Wendy L McArdle, David P Strachan.
Contributions: project conception, design and management: D.P.S., phenotype collection and data management: W.L.M., D.P.S., data analysis: D.P.S. **Funding & Acknowledgements**: We acknowledge use of phenotype and genotype data from the British 1958 Birth Cohort DNA collection, funded by the Medical Research Council grant G0000934 and the Wellcome Trust grant 068545/Z/02. (http://www.b58cgene.sgul.ac.uk/). Genotyping for the B58C-WTCCC subset was funded by the Wellcome Trust grant 076113/B/04/Z. The B58C-T1DGC genotyping utilized resources provided by the Type 1 Diabetes Genetics Consortium, a collaborative clinical study sponsored by the National Institute of Diabetes and Digestive and Kidney Diseases (NIDDK), National Institute of Allergy and Infectious Diseases (NIAID), National Human Genome Research Institute (NHGRI), National Institute of Child Health and Human Development (NICHD), and Juvenile Diabetes Research Foundation International (JDRF) and supported by U01 DK062418. B58C-T1DGC GWAS data were deposited by the Diabetes and Inflammation Laboratory, Cambridge Institute for Medical Research (CIMR), University of Cambridge, which is funded by Juvenile Diabetes Research Foundation International, the Wellcome Trust and the National Institute for Health Research Cambridge Biomedical Research Centre; the CIMR is in receipt of a Wellcome Trust Strategic Award (079895).

The B58C-GABRIEL genotyping was supported by a contract from the European Commission Framework Programme 6 (018996) and grants from the French Ministry of Research.

BHS1&2

Investigators: John Beilby, Jennie Hui, Alan L James, AW Musk.
Contributions: phenotype collection and data management: J.B., J. Hui, A.L.J, A.W.M. **Funding & Acknowledgements**: The Busselton Health Study (BHS) acknowledges the generous support for the 1994/5 follow-up study from Healthway, Western Australia and the numerous Busselton community volunteers who assisted with data collection and the study participants from the Shire of Busselton. The Busselton Health Study is supported by The Great Wine Estates of the Margaret River region of Western Australia. GWAS genotyping was supported by a research collaboration with Pfizer.

CROATIA-Korcula

Investigators: Caroline Hayward, Jennifer E Huffman, Pau Navarro.
Contributions: project conception, design and management: C.H., phenotype collection and data management: C.H., J.E.H., P.N., data analysis: J.E.H., P.N. **Funding & Acknowledgements**: The CROATIA study was supported through grants from the Medical Research Council UK, the Ministry of Science, Education and Sport in the Republic of Croatia (number 216-1080315-0302), Croatian Science Foundation (grant 8875) and the European Union framework program 6 EUROSPAN project (contract no. LSHG-CT-2006-018947). SNP genotyping for CROATIA-Vis was performed by the Wellcome Trust Clinical Research Facility (WTCRF) at the Western General Hospital, Edinburgh, UK. CROATIA-Korcula was genotyped by Helmholz Zentrum München, GmbH, Neuherberg, Germany and CROATIA-Split by AROS Applied Biotechnology, Aarhus, Denmark. We would like to acknowledge the invaluable contributions of the recruitment teams in Croatia (including those from the Institute of Anthropological Research in Zagreb and the Croatian Centre for Global Health at the University of Split), the administrative teams in Croatia and Edinburgh and the people of Korcula, Vis and Split.

CROATIA-Split

Investigators: Ivana Kolcic, Ozren Polasek, Veronique Vitart, Tatijana Zemunik.
Contributions: project conception, design and management: I.K., O.P., V.V., T.Z., phenotype collection and data management: I.K., O.P., V.V., T.Z., **Funding & Acknowledgements**: The CROATIA study was supported through grants from the Medical Research Council UK, the Ministry of Science, Education and Sport in the Republic of Croatia (number 216-1080315-0302), Croatian Science Foundation (grant 8875) and the European Union framework program 6 EUROSPAN project (contract no. LSHG-CT-2006-018947). SNP genotyping for CROATIA-Vis was performed by the Wellcome Trust Clinical Research Facility (WTCRF) at the Western General Hospital, Edinburgh, UK. CROATIA-Korcula was genotyped by Helmholz Zentrum München, GmbH, Neuherberg, Germany

and CROATIA-Split by AROS Applied Biotechnology, Aarhus, Denmark. We would like to acknowledge the invaluable contributions of the recruitment teams in Croatia (including those from the Institute of Anthropological Research in Zagreb and the Croatian Centre for Global Health at the University of Split), the administrative teams in Croatia and Edinburgh and the people of Korcula, Vis and Split.

CROATIA-Vis

Investigators: Jonathan Marten, Igor Rudan, Alan F Wright.
Contributions: project conception, design and management: I.R., A.F.W., phenotype collection and data management: J.M., data analysis: J.M. **Funding & Acknowledgements**: The CROATIA study was supported through grants from the Medical Research Council UK, the Ministry of Science, Education and Sport in the Republic of Croatia (number 216-1080315-0302), Croatian Science Foundation (grant 8875) and the European Union framework program 6 EUROSPAN project (contract no. LSHG-CT-2006-018947). SNP genotyping for CROATIA-Vis was performed by the Wellcome Trust Clinical Research Facility (WTCRF) at the Western General Hospital, Edinburgh, UK. CROATIA-Korcula was genotyped by Helmholz Zentrum München, GmbH, Neuherberg, Germany and CROATIA-Split by AROS Applied Biotechnology, Aarhus, Denmark. We would like to acknowledge the invaluable contributions of the recruitment teams in Croatia (including those from the Institute of Anthropological Research in Zagreb and the Croatian Centre for Global Health at the University of Split), the administrative teams in Croatia and Edinburgh and the people of Korcula, Vis and Split.

EPIC

Investigators: Jing Hua Zhao, Robert A Scott, Nicholas J Wareham.
Contributions: project conception, design and management: J.H.Z., R.A.S., N.J.W., phenotype collection and data management: J.H.Z., data analysis: J.H.Z., **Funding & Acknowledgements**: The EPIC Norfolk Study is funded by program grants from the Medical Research Council UK and Cancer Research UK, and by additional support from the European Union, Stroke Association, British Heart Foundation, Department of Health, Food Standards Agency, and the Wellcome Trust.

GS:SFHS

Investigators: Lynne Hocking, Sandosh Padmanabhan, Generation Scotland, Holly Trochet.
Contributions: project conception, design and management: L.H., S.P., G.S., phenotype collection and data management: L.H., S.P., G.S., H.T. **Funding & Acknowledgements**: GS:SFHS is funded by the Scottish Executive Health Department, Chief Scientist Office, grant number CZD/16/6. Exome array genotyping for GS:SFHS was funded by the Medical Research Council UK and performed at the Wellcome Trust Clinical Research Facility Genetics Core at Western General Hospital, Edinburgh, UK. We would like to acknowledge the invaluable contributions of the families who took part in the

Generation Scotland: Scottish Family Health Study, the general practitioners and Scottish School of Primary Care for their help in recruiting them, and the whole Generation Scotland team, which includes academic researchers, IT staff, laboratory technicians, statisticians and research managers.

H2000

Investigators: Markku Heliövaara, Mika Kähönen, Samuli Ripatti, Ida Surakka.
Contributions: project conception, design and management: M. Heliövaara, M.K., phenotype collection and data management: M. Heliövaara, M.K., S.R, I. Surakka, data analysis: M.K., S.R, I. Surakka **Funding & Acknowledgements**: This study was financially supported by the Medical Research Fund of the Tampere University Hospital. S.R. was supported by the Academy of Finland (251217 and 255847), Center of Excellence in Complex Disease Genetics, EU FP7 projects ENGAGE (201413) and BioSHaRE (261433), the Finnish Foundation for Cardiovascular Research, Biocentrum Helsinki, and the Sigrid Juselius Foundation.

KORA F4

Investigators: Eva Albrecht, Harald Grallert, Joachim Heinrich, Janina S Ried.
Contributions: project conception, design and management: J. Heinrich, phenotype collection and data management: H.G., J.S.R., data analysis: E.A., J.S.R. **Funding & Acknowledgements**: The KORA authors acknowledge all members of field staffs who were involved in the planning and conduction of the KORA Augsburg studies, as well as all KORA study participants. The KORA research platform (KORA, Cooperative Health Research in the Region of Augsburg) was initiated and financed by the Helmholtz Zentrum München - German Research Center for Environmental Health which is funded by the German Federal Ministry of Education and Research and by the State of Bavaria. The KORA-Age project was financed by the German Federal Ministry of Education and Research (BMBF FKZ 01ET0713 and 01ET1003A) as part of the 'Health in old age' program. Furthermore, KORA research was supported within the Munich Center of Health Sciences (MC Health), Ludwig-Maximilians-Universität, as part of LMUinnovativ. Further support was provided by the Competence Network ASCONET, subnetwork COSYCONET (FKZ 01GI0882).

KORA S3

Investigators: Christian Gieger, Stefan Karrasch, Rajesh Rawal, Holger Schulz.
Contributions: project conception, design and management: C.G., S.K., H.S., phenotype collection and data management: C.G., S.K., R.R., H.S., data analysis: R.R., **Funding & Acknowledgements**: The KORA authors acknowledge all members of field staffs who were involved in the planning and conduction of the KORA Augsburg studies, as well as all KORA study participants. The KORA research platform (KORA, Cooperative Health Research in the Region of Augsburg) was initiated and financed by the Helmholtz Zentrum München - German Research Center for Environmental Health which is funded by the German Federal Ministry of Education and Research and by the State of Bavaria. The KORA-Age project was financed by the German Federal Ministry of Education and

Research (BMBF FKZ 01ET0713 and 01ET1003A) as part of the ‘Health in old age’ program. Furthermore, KORA research was supported within the Munich Center of Health Sciences (MC Health), Ludwig-Maximilians-Universität, as part of LMUinnovativ. Further support was provided by the Competence Network ASCONET, subnetwork COSYCONET (FKZ 01GI0882).

LBC1936

Investigators: Ian J Deary, Sarah E Harris, Lorna M Lopez, John M Starr
Contributions: project conception, design and management: I.J.D., J.M.S., phenotype collection and data management: I.J.D., S.E.H., L.M.L., J.M.S., data analysis: L.M.L. **Funding & Acknowledgements**: We thank the cohort participants who contributed to this study. Genotyping was supported by the UK’s Biotechnology and Biological Sciences Research Council (BBSRC) (Ref. BB/F019394/1). Phenotype collection was supported by Research Into Ageing (continues as part of Age UK’s The Disconnected Mind project). The work was undertaken by The University of Edinburgh Centre for Cognitive Ageing and Cognitive Epidemiology, part of the cross council Lifelong Health and Wellbeing Initiative (MR/K026992/1). Funding from the BBSRC and Medical Research Council (MRC) is gratefully acknowledged.

NFBC1966

Investigators: Alexessander Couto Alves, Anna-Liisa Hartikainen, Momoko Horikoshi, Marjo-Riitta Jarvelin.
Contributions: project conception, design and management: M-R.J., phenotype collection and data management: A.C.A., A-L.H., M-R.J., data analysis: A.C.A., M. Horikoshi, M-R.J. **Funding & Acknowledgements**: We thank the late Professor Paula Rantakallio (launch of NFBC1966), and Ms Outi Tornwall and Ms MinttuJussila (DNA biobanking). NFBC1966 received financial support from the Academy of Finland (project grants 104781, 120315, 129269, 1114194, 24300796), University Hospital Oulu, Biocenter, University of Oulu, Finland (75617), NHLBI grant 5R01HL087679-02 through the STAMPEED program (1RL1MH083268-01), NIH/NIMH (5R01MH63706:02), EU FP7 (HEALTH-F4-2007-201413), EU FP8 (277849), Medical Research Council, UK (G0500539, G1002319, G0600705).

NSPHS

Investigators: Stefan Enroth, Ulf Gyllensten, Åsa Johansson.
Contributions: project conception, design and management: U.G., phenotype collection and data management: S.E., A.J., data analysis: S.E. **Funding & Acknowledgements**: U.G. acknowledges Swedish Medical Research Council (K2007-66X-20270-01-3, 2012-2884), Foundation for Strategic Research (SSF) and European Commission FP6 STRP (LSHG-CT-2006-01947). A.J. acknowledges Swedish Society for Medical Research (SSMF).

ORCADES

Investigators: Harry Campbell, Peter K Joshi, Sarah H Wild, James F Wilson.
Contributions: project conception, design and management: H.C., S.H.W., J.F.W., phenotype collection and data management: H.C., S.H.W., J.F.W., data analysis: P.K.J. **Funding & Acknowledgements**: The ORCADES study was funded by the Chief Scientist Office of the Scottish Government, the Royal Society and the MRC Human Genetics Unit. DNA extraction was performed at the Wellcome Trust Clinical Research Facility in Edinburgh. Genotyping was funded by the European Union Framework Programme 6 EUROSPAN project.

SAPALDIA

Investigators: Medea Imboden, Ashish Kumar, Nicole M Probst-Hensch.
Contributions: project conception, design and management: N.M.P-H., phenotype collection and data management: M.I., A.K., N.M.P-H., data analysis: M.I., A.K., N.M.P-H. **Funding & Acknowledgements**: Study directorate: NM Probst-Hensch (PI; e/g); T Rochat (p), C Schindler (s), N Künzli (e/exp), JM Gaspoz (c) Scientific team: JC Barthélémy (c), W Berger (g), R Bettschart (p), A Bircher (a), C Brombach (n), PO Bridevaux (p), L Burdet (p), Felber Dietrich D (e), M Frey (p), U Frey (pd), MW Gerbase (p), D Gold (e), E de Groot (c), W Karrer (p), F Kronenberg (g), B Martin (pa), A Mehta (e), D Miedinger (o), M Pons (p), F Roche (c), T Rothe (p), P Schmid-Grendelmeyer (a), D Stolz (p), A Schmidt-Trucksäss (pa), J Schwartz (e), A Turk (p), A von Eckardstein (cc), E Zemp Stutz (e). Scientific team at coordinating centers: M Adam (e), I Aguilera (exp), S Brunner (s), D Carballo (c), S Caviezel (pa), I Curjuric (e), A Di Pascale (s), J Dratva (e), R Ducret (s), E Dupuis Lozeron (s), M Eeftens (exp), I Eze (e), E Fischer (g), M Foraster (e), M Germond (s), L Grize (s), S Hansen (e), A Hensel (s), M Imboden (g), A Ineichen (exp), A Jeong (g), D Keidel (s), A Kumar (g), N Maire (s), A Mehta (e), R Meier (exp), E Schaffner (s), T Schikowski (e), M Tsai (exp)
(a) allergology, (c) cardiology, (cc) clinical chemistry, (e) epidemiology, (exp) exposure, (g) genetic and molecular biology, (m) meteorology, (n) nutrition, (o) occupational health, (p) pneumology, (pa) physical activity, (pd) pediatrics, (s) statistic. The study could not have been done without the help of the study participants, technical and administrative support and the medical teams and field workers at the local study sites. Local fieldworkers: Aarau: S Brun, G Giger, M Sperisen, M Stahel, Basel: C Bürli, C Dahler, N Oertli, I Harreh, F Karrer, G Novicic, N Wyttenbacher, Davos: A Saner, P Senn, R Winzeler, Geneva: F Bonfils, B Blicharz, C Landolt, J Rochat, Lugano: S Boccia, E Gehrig, MT Mandia, G Solari, B Viscardi, Montana: AP Bieri, C Darioly, M Maire, Payerne: F Ding, P Danieli A Vonnez, Wald: D Bodmer, E Hochstrasser, R Kunz, C Meier, J Rakic, U Schafroth, A Walder. Administrative staff: N Bauer Ott, C Gabriel, R Gutknecht. Funding: The Swiss National Science Foundation (grants no 33CS30-148470/1, 33CSCO-134276/1, 33CSCO-108796, 3247BO-104283, 3247BO-104288, 3247BO-104284, 3247-065896, 3100-059302, 3200-052720, 3200-042532, 4026-028099, PMPDP3_129021/1, PMPDP3_141671/1), the Federal Office for the Environment, the Federal Office of Public Health, the Federal Office of Roads and Transport, the canton's government

of Aargau, Basel-Stadt, Basel-Land, Geneva, Luzern, Ticino, Valais, and Zürich, the Swiss Lung League, the canton's Lung League of Basel Stadt/ Basel Landschaft, Geneva, Ticino, Valais, Graubünden and Zurich, Stiftung ehemals Bündner Heilstätten, SUVA, Freiwillige Akademische Gesellschaft, UBS Wealth Foundation, Talecris Biotherapeutics GmbH, Abbott Diagnostics, European Commission 018996 (GABRIEL), Wellcome Trust WT 084703MA.

SHIP

Investigators: Sven Gläser, Beate Koch, Alexander Teumer, Henry Völzke.
Contributions: project conception, design and management: S.G., B.K., H.V., phenotype collection and data management: S.G., B.K., A.T., H.V., data analysis: A.T. **Funding & Acknowledgements**: SHIP is part of the Community Medicine Research net of the University of Greifswald, Germany, which is funded by the Federal Ministry of Education and Research, the Ministry of Cultural Affairs as well as the Social Ministry of the Federal State of Mecklenburg-West Pomerania, and the network 'Greifswald Approach to Individualized Medicine (GANI_MED)' funded by the Federal Ministry of Education and Research, and the German Asthma and COPD Network (COSYCONET) (grant no.01ZZ9603, 01ZZ0103, 01ZZ0403, 03IS2061A, BMBF 01GI0883). Genome-wide data have been supported by the Federal Ministry of Education and Research and a joint grant from Siemens Healthcare, Erlangen, Germany and the Federal State of Mecklenburg- West Pomerania (grant no. 03ZIK012). The University of Greifswald is a member of the 'Center of Knowledge Interchange' program of the Siemens AG and the Caché Campus program of the InterSystems GmbH.

YFS

Investigators: Nina Hutri-Kähönen, Terho Lehtimäki, Leo-Pekka Lyytikäinen, Olli T Raitakari.
Contributions: project conception, design and management: T.L., O.T.R., phenotype collection and data management: N.H-K., T.L., O.T.R., data analysis: L-P.L. **Funding & Acknowledgements**: The authors acknowledge Academy of Finland (126925, 121584, 124282), Academy of Finland (Eye) (134309), Academy of Finland (Salve) (129378), Academy of Finland (Gendi) (117787), Academy of Finland (Skidi) (41071), Social Insurance Institution of Finland,Tampere University Hospital Medical Funds (X51001 for T.L.), Kuopio University Hospital Medical Funds, Turku University Hospital Medical Funds, Juho Vainio Foundation, Paavo Nurmi Foundation, Finnish Foundation of Cardiovascular Research (T.L.), Finnish Cultural Foundation, Tuberculosis Foundation (T.L.), Emil Aaltonen Foundation (T.L), and Yrjö Jahnsson Foundation (T.L.).

Follow-up studies

ALSPAC

Investigators: David M Evans, Raquel Granell, John Henderson, John P Kemp.

Contributions: phenotype collection and data management: R.G., J. Henderson, J.P.K., data analysis: D.M.E. **Funding & Acknowledgements**: We are extremely grateful to all the families who took part in the study, the midwives for their help in recruiting them, and the whole ALSPAC team, which includes interviewers, computer and laboratory technicians, clerical workers, research scientists, volunteers, managers, receptionists and nurses. The UK Medical Research Council and the Wellcome Trust (Grant ref: 092731) and the University of Bristol provide core support for ALSPAC. This publication is the work of the authors and D.M.E will serve as guarantor for the contents of this paper.

UK BiLEVE

Funding & Acknowledgements: This work was funded by a Medical Research Council (MRC) strategic award to MDT, IPH, DS and LVW (MC_PC_12010). This research has been conducted using the UK Biobank Resource. MDT has been supported by MRC fellowships G0501942 and G0902313. IPH is supported by an MRC programme grant (G1000861). Jonathan Marchini is funded by an ERC Consolidator Grant (617306). This article presents independent research funded partially by the National Institute for Health Research (NIHR). The views expressed are those of the author(s) and not necessarily those of the NHS, the NIHR or the Department of Health. We would like to acknowledge all members of the UK Biobank Array Design Group: Peter Donnelly (chair) (University of Oxford), Jeff Barrett (Wellcome Trust Sanger Institute), Jose Bras (University College London), Adam Butterworth (University of Cambridge), Richard Durbin (Wellcome Trust Sanger Institute), Paul Elliott (Imperial College London), Ian Hall (University of Nottingham), John Hardy (University College London), Mark McCarthy (University of Oxford), Gil McVean (University of Oxford), Tim Peakman (UK Biobank), Nazneen Rahman (The Institute of Cancer Research), Nilesh Samani (University of Leicester), Martin Tobin (University of Leicester), Hugh Watkins (University of Oxford).

ECRHS

Investigators: Claudia Flexeder, Juan R González, Joachim Heinrich, Deborah L Jarvis.
Contributions: project conception, design and management: J. Heinrich, D.L.J., phenotype collection and data management: J.R.G., J. Heinrich, D.L.J., data analysis: C.F. **Funding & Acknowledgements**: Authors acknowledge EU funding (GABRIEL GRANT Number: 018996, ECRHS II Coordination Number: QLK4-CT-1999-01237).

PIVUS

Investigators: Erik Ingelsson, Lars Lind, Anubha Mahajan, Andrew P Morris.
Contributions: project conception, design and management: L.L., phenotype collection and data management: E.I., L.L., A.M., A.P.M., data analysis: A.M., A.P.M. **Funding & Acknowledgements**: A.P.M. acknowledges the Wellcome Trust (WT098017,WT064890, WT090532). The PIVUS study acknowledges The Swedish Foundation for Strategic Research (ICA08-0047), The Swedish

Research Council (2012-1397), The Swedish Heart-Lung Foundation (20120197), The Swedish Society of Medicine and Uppsala University. The computations were performed on resources provided by SNIC through Uppsala Multidisciplinary Center for Advanced Computational Science (UPPMAX) under Project p2013056. APM is a Wellcome Trust Senior Research Fellow in Basic Biomedical Science (grant number WT098017).

TwinsUK

Investigators: Chris J Hammond, Pirro G Hysi, Tim D Spector, Ana Viñuela.
Contributions: project conception, design and management: T.D.S., phenotype collection and data management: C.J.H., P.G.H., T.D.S., A.V., data analysis: P.G.H. **Funding & Acknowledgements**: This cohort received funding from the Wellcome Trust; the European Community's Seventh Framework Programme (FP7 /2007-13); US National Institutes of Health/National Eye Institute (1RO1EY018246); NIH Center for Inherited Disease Research; the National Institute for Health Research (NIHR)- funded BioResource, Clinical Research Facility and Biomedical Research Centre based at Guy's and St Thomas' NHS Foundation Trust in partnership with King's College London.

Lung eQTL study

Investigators: Yohan Bossé, Corry-Anke Brandsma, David C Nickle, Ma'en Obeidat, Don D Sin.
Contributions: phenotype collection and data management: Y.B., C-A.B., D.C.N., D.D.S., data analysis: M.O. **Funding & Acknowledgements**: The authors would like to thank the research staff at the Respiratory Health Network Tissue Bank of the FRQS for collecting lung specimens for the lung eQTL study at Laval University. Lung tissue eQTL study was funded by Merck Research Laboratories. M.O. is a Postdoctoral Fellow of the Michael Smith Foundation for Health Research (MSFHR) and the Canadian Institute for Health Research (CIHR) Integrated and Mentored Pulmonary and Cardiovascular Training program (IMPACT). Y.B. is the recipient of a Junior 2 Research Scholar award from the Fonds de recherche Québec – Santé (FRQS).

Supplementary Methods:

Lung eQTL analyses

Variants in 2Mb regions (new sentinel variant +/- 1Mb) which were in LD ($r^2 > 0.3$) with the sentinel variant, for each of the 16 new sentinel variants, were assessed in a lung eQTL resource based on lung tissues of 1,111 individuals. Variants were selected if they (i) met an FDR threshold of 10%, and (ii) had eQTL P value < 5×10^{-5}. In addition, for each probeset we ranked variants according to their correlation with the sentinel SNP (as measured by r^2) first and to their eQTL P value second, and we only selected results for the top ranked variant for each probeset.

The descriptions of the lung eQTL dataset and subject demographics have been published previously[7-9]. Briefly, non-tumor lung tissues were collected from patients who underwent lung resection surgery at three participating sites: Laval University (Quebec City, Canada), University of Groningen (Groningen, The Netherlands), and University of British Columbia (Vancouver, Canada). Whole-genome gene expression and genotyping data were obtained from these specimens. Gene expression profiling was performed using an Affymetrix custom array (GPL10379) testing 51,627 non-control probe sets and normalized using RMA[106]. Genotyping was performed using the Illumina Human1M-Duo BeadChip array. Genotype imputation was undertaken using the 1000 Genomes Project[107] reference panel. Following standard microarray and genotyping quality controls, 1111 patients were available including 409 from Laval, 363 from Groningen, and 339 from UBC. Association testing for each variant with mRNA expression in either *cis* (within 1 Mb of transcript start site) or in *trans* (all other eQTLs) was undertaken separately in each study, then their results were meta-analysed using inverse variance weighting meta-analysis and a 10% FDR genome-wide was applied. For the X chromosome only HapMap imputed data was available and the association testing was undertaken separately for males and females in each study.

Blood eQTL analyses

Variants in 2Mb regions (new sentinel variant +/- 1Mb) which were in LD ($r^2 > 0.3$) with the sentinel variant, for each of the 16 new sentinel variants, and were in HapMap were assessed in a publicly available blood eQTL dataset[6] with results from the analysis of 5,311 individuals. Association testing was undertaken both for *cis* (distance between the SNP and the probe midpoint < 250kb) and *trans* (distance between the SNP and the probe midpoint >5Mb) eQTL signals. No X chromosome data was available. Variants were selected if they (i) met an FDR threshold of 10%, and (ii) had eQTL *P* value $< 5 \times 10^{-5}$. In addition, for each probeset we ranked variants according to their correlation with the sentinel SNP (as measured by r^2) first and to their eQTL *P* value second, and we only selected results for the top ranked variant for each probeset.

DNase hypersensitivity analysis

DNase hypersensitivity sites as defined by ENCODE[108] within 2Mb regions centred in the sentinel variant for the 16 new sentinel variants were accessed using the Uniform DNaseI HS track in the UCSC table browser[109] in the following cell types: A549, epithelial cell line derived from a lung carcinoma tissue; IMR90, fetal lung fibroblasts; AG04450, fetal lung fibroblast; BC_Lung_01-11002, lung, donor 01-11002, age 83, caucasian, DNA and RNA extract; BC_Lung_H12817N, lung, donor H12817N, age 71, caucasian, DNA and RNA extra; FibroP_AG08396, lung fibroblasts taken from individuals with Parkinson's disease; HMVEC-LBl, blood microvascular endothelial cells, lung-derived; HMVEC-LLy, lymphatic microvascular endothelial cells, lung-derived; HPF, pulmonary fibroblasts isolated from lung tissue; HPAEC, pulmonary artery endothelial cells; HPAEpiC, pulmonary alveolar epithelial cells; HPAF, pulmonary artery fibroblasts; Lung_BC, lung, parental cell type to donors 01-11002 and H12817N; Lung_OC, primary frozen lung tissue from NICHD donor IDs 1104 (Rep B1

DNase), 602 (Rep B2 DNase), 1442 (Rep B3 DNase) and CF Center donor IDs DD006I (Rep B1 FAIRE) and DD007I (Rep B2 FAIRE); NHLF, lung fibroblasts; TBEC, trachea and bronchial epithelial cells from frush lung tissue provided by National Disease Research Interchange, passage 2 primary, non-immortalized cells, donor was 21 years old; WI-38, embryonic lung fibroblast cells, hTERT immortalized, includes Raf1 construct. More details on cell type definitions can be found here http://genome.ucsc.edu/ENCODE/cellTypes.html. Sites for any cell which contained at least one variant strongly correlated ($r^2 > 0.8$) with any of the new 16 sentinel variants were selected.

Conditional analysis: functional annotation

In order to identify suggestive functional variants responsible for the new signals we observed or already known signals from previous GWAS studies[13,15-17], 2Mb windows (1Mb either side of the sentinel SNP for the novel and known signals) were searched for functional variants. Variants were annotated using ENSEMBL's Variant Effect Predictor [16] and functional effects were predicted with SIFT[17], PolyPhen-2[18], CADD[19], and GWAVA[110] databases. If a variant was annotated as 'deleterious' by SIFT, 'probably damaging' or 'potentially damaging' by PolyPhen-2, had a CADD scaled score ≥ 20 (CADD_PHRED ≥ 20), or had a GWAVA score > 0.5, it was treated as a functional variant. CADD (Combined Annotation-Dependent Depletion) is a method for integrating many diverse annotations, namely conservation metrics, functional genomic data, transcript information, and protein level scores into a single score for each coding and noncoding variant. Scaled CADD score (CADD_PHRED) ranks each variant relative to all possible substitutions of the human genome (~8.6 billion SNVs of the GRCh37/hg19 reference genome). A scaled CADD score greater or equal to 20 for a variant, indicates that the variant is within the 1% most deleterious variants in the human genome [19]. GWAVA (genome-wide annotation of variants) is a tool that combines information from a wide range of annotations to predict the functional impact of noncoding variants. We used a GWAVA score

threshold of 0.5, as proposed by authors, above which noncoding variants were considered as 'deleterious' [110]. Annotation results were filtered with VEP's --pick flag, which selects only one consequence per variant based on the canonical biotype status and length of the transcript as well as the ranking of the consequence type. For variants with multiple annotations, we selected the most deleterious annotation (i.e. if a variant was annotated as frameshift variant and intronic variant, the variant was considered to be frameshift). For each locus, functional variants that had a stage 1 *P* value < 5×10^{-4} or were in LD with the sentinel SNP ($r^2 > 0.3$) were selected. Each functional variant was then tested jointly with the sentinel variant in the region, using GCTA[20] and data from 5,000 individuals from UK BiLEVE to estimate LD, to see whether the functional variant would explain for the signal. We considered that a signal was explained by a functional variant if the joint *P* value for the sentinel variant when fitted with the functional variant was > 0.01.

Selection of priority genes

For each of the 16 loci that comprised the novel signals a small number of genes were selected as priority genes for subsequent analyses. These genes were selected according to the following criteria, based on eQTL results from blood and lung tissue for the sentinel variant in each region and for variants in LD ($r^2>0.3$) with the sentinel (**Supplementary Table 3 a**), and the results of the conditional analysis on functional variants (**Supplementary Table 3 f**). eQTL results were restricted to variants that met a 10% FDR threshold and had eQTL $P<5x10^{-5}$. For each probeset, variants were ranked first according to r^2 with the sentinel variant in the region, and second by the eQTL *P* value. Only the highest ranking variant was kept for each probeset. If the association of a sentinel variant was explained by a missense variant according to the conditional analysis, the gene where the missense variant lies was selected (this was the case for three regions: *GPR126, RIN3* and *LTBP4*). If no missense variant explained the association and no eQTL results met the FDR and *P* value

thresholds for a region, the gene closest to the sentinel variant was chosen (this was the case for three regions: *KCNS3*, *TBX3*, and *MN1*). If there was only evidence from blood eQTL up to two of the most significant genes that met the FDR and *P* value thresholds were selected (this was the case for one region: *NPNT*). In the *NPNT* region, *NPNT* was added to the list of priority genes as well as the genes implicated by the eQTL analysis, given the prior evidence of association with lung function for *NPNT* [2]. If there was evidence from lung tissue eQTL up to two of the most significant genes that met the FDR and *P* value thresholds for lung were selected (this was the case for 8 regions: *RNU5F-1*, *AK097794*, *ASTN2*, LHX3, *CCDC91*, *TRIP11*, *EMP2* and *AP1S2*), except for the *ENSA* region where an additional gene with a very significant *P* value for the blood eQTL ($P>5\times10^{-120}$) which also had a $P< 5\times10^{-5}$ for lung tissue was included.

Messenger RNA sequencing

Passage 3 normal human bronchial epithelial cells (NHBECs) (Lonza, UK), were cultured in growth factor-supplemented medium (BEGM, Lonza as described previously[111]). Cells were grown under these conditions and four different experimental conditions as part of a related RNA interference (RNAi) project each in three independent biological replicates (12 samples in total). Total RNA was extracted using established methods for RNA isolation (Sigma-Aldrich GenElute Mammalian Total RNA Miniprep Kit) and RNA quality was assessed for degradation on Agilent 2100 Bioanalyzer with all twelve samples having a RNA Integrity Number (RIN) at ~8 or above 8. The sequencing library was prepared with Illumina TruSeq RNA Sample Prep Kit v2. mRNA was poly-A selected by capturing total RNA samples with oligo-dT coated magnetic beads. The mRNA was then fragmented and randomly primed. cDNA was synthesised using random primers. Finally ready-for-sequencing library was prepared by end-repair, phosphorylation, A-tailing, adapter ligation and PCR amplification. Paired-end sequencing was performed on the Illumina HiSeq2000 platform using TruSeq v3

chemistry over 100 cycles yielding approximately 40 million reads per sample. The generated raw reads FastQ files (100 base pairs; Sanger / Illumina 1.9 encoding) were quality evaluated using FastQC. Mean quality scores across the bases for all reads in all twelve samples were above 28. Un-modified reads were used for subsequent analysis on Ubuntu 12.04 LTS operating system. Un-spliced alignments onto human genome build GRCh37 were performed for each sample individually using Bowtie2 tool utilized by TopHat v2.0.12 [112]. Reads aligning to more than 20 positions were discarded. The subset of reads that were not aligned uniquely were used by TopHat [112] to identify splice junctions. Cufflinks v2.2.1 programme [113,114] was used to assemble transcriptome for each individual sample. Transcriptomes from all the samples were merged using Cuffmerge v1.0.0 feature [113,114] in order to identify low-expression transcripts requiring deep sequencing coverage. The Cuffmerge [113,114] generated novel gene transfer format (GTF) annotation file was compared to Ensembl GTF annotation of GRCh37 genome build by using Cuffcompare v2.2.1 [113,114]. All 12 NHBEC samples were used to identify reported and novel transcripts. The three untreated basal NHBEC samples were used to determine abundance under basal culture conditions, mean FPKM expressions of individual isoforms were calculated and their percentage of total transcripts expression determined. Splicing graphs depicting novel and known splice transcripts were generated using SpliceGrapher v0.2.4 [115].

Fetal differential expression

Publically available Affymetrix U133 Plus 2 array data (Gene expression omnibus: GSE14334) of 38 fetal lung samples spanning the Pseudoglandular (7–16 weeks) and Canalicular (17–22 weeks) stages of lung development was mined as previously reported[13]. *P* values were adjusted for multiple testing using the Benjamini & Hochberg method[14]. Probes for each of a subset of genes in the 16 loci

(see criteria for selection in the “Selection of priority genes” section in the **Supplementary Methods**) were searched for within the results.

COPD differential expression

Publically available microarray data (GSE37147[15]) was mined using GEO2R on the gene expression omnibus website (http://www.ncbi.nlm.nih.gov/geo/info/geo2r.html). Two sample groups were defined. Affymetrix Human ST1.0 array expression data, which includes one probe per gene, for 87 bronchial brushings in the lungs of individuals with COPD was defined as the first group, whilst the second group had the expression profiles of 151 bronchial brushings from ever smoker individuals without COPD. Differential expression between the 2 groups was identified using the default array statistics. *P* values were adjusted for multiple testing using the Benjamini & Hochberg method [14]. Probes for each of a subset of genes in the 16 loci (see criteria for selection in the “Selection of priority genes” section in the **Supplementary Methods**) were searched for within the results.

MAGENTA

Stage 1 GWAS results were enriched for known biological pathways using MAGENTA v2[116]. MAGENTA defines a *P* value for each gene that is the lowest *P* value within 110kb upstream and 40kb downstream of the gene and is corrected for gene size, number of SNPs per gene and linkage disequilibrium within the region. For each gene set, the null hypothesis that there is a random distribution of gene association score ranks within the gene set is tested against the alternative hypothesis that there are more gene association score ranks above a given rank cut-off (75th percentile cut-off is recommended for polygenic traits) compared to random sampling of 10,000 gene sets of identical size. For each gene set, a false discovery rate (FDR) is calculated as the fraction of

all randomly sampled gene sets (10,000 x number of gene sets tested) that have more genes with *P* value below the cut off (75th percentile) than in the gene set being tested, divided by the fraction of real gene sets that have more genes with *P* value below the cut off (75th percentile) than in the gene set being tested.

Six databases of biological pathways were tested: including Ingenuity Pathway (June 2008, number of pathways n=81), KEGG (2010, n=186), PANTHER Molecular Function (January 2010, n=216), PANTHER Biological Processes (January 2010, n=217), PANTHER Pathways (January 2010, n=94) and Gene Ontology (April 2010, n=1778). Significance thresholds were Bonferroni corrected for each database.

Genes within 500kb either side from the sentinel variants were flagged in the analysis. Sensitivity analyses were run removing genes in the HLA region on chromosome 6.

Supplementary References:

1. Wilk, J.B. *et al.* A genome-wide association study of pulmonary function measures in the Framingham Heart Study. *PLoS Genet* 5, e1000429 (2009).
2. Hancock, D.B. *et al.* Meta-analyses of genome-wide association studies identify multiple loci associated with pulmonary function. *Nat Genet* 42, 45-52 (2010).
3. Soler Artigas, M. *et al.* Genome-wide association and large-scale follow up identifies 16 new loci influencing lung function. *Nature Genetics* 43, 1082-1090 (2011).
4. Loth, D.W. *et al.* Genome-wide association analysis identifies six new loci associated with forced vital capacity. *Nat Genet* 46, 669-77 (2014).
5. Repapi, E. *et al.* Genome-wide association study identifies five loci associated with lung function. *Nat Genet* 42, 36-44 (2010).
6. Westra, H.J. *et al.* Systematic identification of trans eQTLs as putative drivers of known disease associations. *Nat Genet* 45, 1238-43 (2013).
7. Hao, K. *et al.* Lung eQTLs to help reveal the molecular underpinnings of asthma. *PLoS Genet* 8, e1003029 (2012).
8. Lamontagne, M. *et al.* Refining susceptibility loci of chronic obstructive pulmonary disease with lung eqtls. *PLoS One* 8, e70220 (2013).
9. Obeidat, M.e. *et al.* GSTCD and INTS12 Regulation and Expression in the Human Lung. *Plos One* 8(2013).
10. Encode Project Consortium. An integrated encyclopedia of DNA elements in the human genome. *Nature* 489, 57-74 (2012).
11. Thurman, R.E. *et al.* The accessible chromatin landscape of the human genome. *Nature* 489, 75-82 (2012).
12. Uhlen, M. *et al.* Towards a knowledge-based Human Protein Atlas. *Nat Biotechnol* 28, 1248-50 (2010).
13. Melen, E. *et al.* Expression analysis of asthma candidate genes during human and murine lung development. *Respir Res* 12, 86 (2011).
14. Benjamini, Y., Hochberg, Y. Controlling the false discovery rate: a practical and powerful approach to multiple testing. *J R Stat Soc B* 57, 289-300 (1995).
15. Steiling, K. *et al.* A dynamic bronchial airway gene expression signature of chronic obstructive pulmonary disease and lung function impairment. *Am J Respir Crit Care Med* 187, 933-42 (2013).
16. McLaren, W. *et al.* Deriving the consequences of genomic variants with the Ensembl API and SNP Effect Predictor. *Bioinformatics* 26, 2069-70 (2010).
17. Kumar, P., Henikoff, S. & Ng, P.C. Predicting the effects of coding non-synonymous variants on protein function using the SIFT algorithm. *Nat Protoc* 4, 1073-81 (2009).
18. Adzhubei, I.A. *et al.* A method and server for predicting damaging missense mutations. *Nat Methods* 7, 248-9 (2010).
19. Kircher, M. *et al.* A general framework for estimating the relative pathogenicity of human genetic variants. *Nat Genet* 46, 310-5 (2014).
20. Yang, J. *et al.* Conditional and joint multiple-SNP analysis of GWAS summary statistics identifies additional variants influencing complex traits. *Nat Genet* 44, 369-75, S1-3 (2012).
21. Ahram, D. *et al.* A homozygous mutation in ADAMTSL4 causes autosomal-recessive isolated ectopia lentis. *Am J Hum Genet* 84, 274-8 (2009).
22. Groenman, F., Rutter, M., Caniggia, I., Tibboel, D. & Post, M. Hypoxia-inducible factors in the first trimester human lung. *J Histochem Cytochem* 55, 355-63 (2007).
23. Otsubo, K. *et al.* ETV6-ARNT fusion in a patient with childhood T lymphoblastic leukemia. *Cancer Genet Cytogenet* 202, 22-6 (2010).

24. Laviad, E.L. *et al.* Characterization of ceramide synthase 2: tissue distribution, substrate specificity, and inhibition by sphingosine 1-phosphate. *J Biol Chem* 283, 5677-84 (2008).
25. Erez-Roman, R., Pienik, R. & Futerman, A.H. Increased ceramide synthase 2 and 6 mRNA levels in breast cancer tissues and correlation with sphingosine kinase expression. *Biochem Biophys Res Commun* 391, 219-23 (2010).
26. Saftig, P. *et al.* Impaired osteoclastic bone resorption leads to osteopetrosis in cathepsin-K-deficient mice. *Proc Natl Acad Sci U S A* 95, 13453-8 (1998).
27. Gelb, B.D., Shi, G.P., Chapman, H.A. & Desnick, R.J. Pycnodysostosis, a lysosomal disease caused by cathepsin K deficiency. *Science* 273, 1236-8 (1996).
28. Costantino, C.M., Ploegh, H.L. & Hafler, D.A. Cathepsin S regulates class II MHC processing in human CD4+ HLA-DR+ T cells. *J Immunol* 183, 945-52 (2009).
29. Reiser, J., Adair, B. & Reinheckel, T. Specialized roles for cysteine cathepsins in health and disease. *J Clin Invest* 120, 3421-31 (2010).
30. Heron, L., Virsolvy, A., Apiou, F., Le Cam, A. & Bataille, D. Isolation, characterization, and chromosomal localization of the human ENSA gene that encodes alpha-endosulfine, a regulator of beta-cell K(ATP) channels. *Diabetes* 48, 1873-6 (1999).
31. Ng, M.M., Dippold, H.C., Buschman, M.D., Noakes, C.J. & Field, S.J. GOLPH3L antagonizes GOLPH3 to determine Golgi morphology. *Mol Biol Cell* 24, 796-808 (2013).
32. Kunigou, O. *et al.* Role of GOLPH3 and GOLPH3L in the proliferation of human rhabdomyosarcoma. *Oncol Rep* 26, 1337-42 (2011).
33. Chen, Y.T. *et al.* Identification of CT46/HORMAD1, an immunogenic cancer/testis antigen encoding a putative meiosis-related protein. *Cancer Immun* 5, 9 (2005).
34. Zhan, Q., Bieszczad, C.K., Bae, I., Fornace, A.J., Jr. & Craig, R.W. Induction of BCL2 family member MCL1 as an early response to DNA damage. *Oncogene* 14, 1031-9 (1997).
35. Okita, H. *et al.* Acute myeloid leukemia possessing jumping translocation is related to highly elevated levels of EAT/mcl-1, a Bcl-2 related gene with anti-apoptotic functions. *Leuk Res* 24, 73-7 (2000).
36. Ryu, H. *et al.* ESET/SETDB1 gene expression and histone H3 (K9) trimethylation in Huntington's disease. *Proc Natl Acad Sci U S A* 103, 19176-81 (2006).
37. Petrache, I. *et al.* Ceramide synthases expression and role of ceramide synthase-2 in the lung: insight from human lung cells and mouse models. *PLoS One* 8, e62968 (2013).
38. Buhling, F. *et al.* Pivotal role of cathepsin K in lung fibrosis. *Am J Pathol* 164, 2203-16 (2004).
39. Quadri, M. *et al.* Mutations in SLC30A10 cause parkinsonism and dystonia with hypermanganesemia, polycythemia, and chronic liver disease. *Am J Hum Genet* 90, 467-77 (2012).
40. Hao, K., Niu, T., Xu, X. & Fang, Z. Single-nucleotide polymorphisms of the KCNS3 gene are significantly associated with airway hyperresponsiveness. *Hum Genet* 116, 378-83 (2005).
41. Nyholt, D.R. *et al.* A high-density association screen of 155 ion transport genes for involvement with common migraine. *Hum Mol Genet* 17, 3318-31 (2008).
42. Wintermeyer, W. *et al.* Mechanisms of elongation on the ribosome: dynamics of a macromolecular machine. *Biochem Soc Trans* 32, 733-7 (2004).
43. Balasubramaniam, S. *et al.* Infantile Progressive Hepatoencephalomyopathy with Combined OXPHOS Deficiency due to Mutations in the Mitochondrial Translation Elongation Factor Gene GFM1. *JIMD Rep* 5, 113-22 (2012).
44. Winteringham, L.N., Kobelke, S., Williams, J.H., Ingley, E. & Klinken, S.P. Myeloid Leukemia Factor 1 inhibits erythropoietin-induced differentiation, cell cycle exit and p27Kip1 accumulation. *Oncogene* 23, 5105-9 (2004).
45. Yoneda-Kato, N. *et al.* The t(3;5)(q25.1;q34) of myelodysplastic syndrome and acute myeloid leukemia produces a novel fusion gene, NPM-MLF1. *Oncogene* 12, 265-75 (1996).

46. Winteringham, L.N. *et al.* Myeloid leukemia factor 1 associates with a novel heterogeneous nuclear ribonucleoprotein U-like molecule. *J Biol Chem* 281, 38791-800 (2006).
47. Sahab, Z.J. *et al.* Tumor suppressor RARRES1 interacts with cytoplasmic carboxypeptidase AGBL2 to regulate the alpha-tubulin tyrosination cycle. *Cancer Res* 71, 1219-28 (2011).
48. Ellinger, J. *et al.* Hypermethylation of cell-free serum DNA indicates worse outcome in patients with bladder cancer. *J Urol* 179, 346-52 (2008).
49. Berndt, S.I. *et al.* Genome-wide meta-analysis identifies 11 new loci for anthropometric traits and provides insights into genetic architecture. *Nat Genet* 45, 501-12 (2013).
50. Potkin, S.G. *et al.* Gene discovery through imaging genetics: identification of two novel genes associated with schizophrenia. *Mol Psychiatry* 14, 416-28 (2009).
51. Board, P.G. & Menon, D. Glutathione transferases, regulators of cellular metabolism and physiology. *Biochim Biophys Acta* 1830, 3267-88 (2013).
52. Hayes, J.D. & Strange, R.C. Glutathione S-transferase polymorphisms and their biological consequences. *Pharmacology* 61, 154-66 (2000).
53. Baillat, D. *et al.* Integrator, a multiprotein mediator of small nuclear RNA processing, associates with the C-terminal repeat of RNA polymerase II. *Cell* 123, 265-76 (2005).
54. Huang, J.T. & Lee, V. Identification and characterization of a novel human nephronectin gene in silico. *Int J Mol Med* 15, 719-24 (2005).
55. Inagaki, F.F. *et al.* Nephronectin is upregulated in acute and chronic hepatitis and aggravates liver injury by recruiting CD4 positive cells. *Biochem Biophys Res Commun* 430, 751-6 (2013).
56. Soranzo, N. *et al.* Meta-analysis of genome-wide scans for human adult stature identifies novel Loci and associations with measures of skeletal frame size. *PLoS Genet* 5, e1000445 (2009).
57. Lango Allen, H. *et al.* Hundreds of variants clustered in genomic loci and biological pathways affect human height. *Nature* 467, 832-8 (2010).
58. Lettre, G. *et al.* Identification of ten loci associated with height highlights new biological pathways in human growth. *Nat Genet* 40, 584-91 (2008).
59. van der Valk, R.J. *et al.* A novel common variant in DCST2 is associated with length in early life and height in adulthood. *Hum Mol Genet* (2014).
60. Kou, I. *et al.* Genetic variants in GPR126 are associated with adolescent idiopathic scoliosis. *Nat Genet* 45, 676-9 (2013).
61. Mogha, A. *et al.* Gpr126 functions in Schwann cells to control differentiation and myelination via G-protein activation. *J Neurosci* 33, 17976-85 (2013).
62. Moriguchi, T. *et al.* DREG, a developmentally regulated G protein-coupled receptor containing two conserved proteolytic cleavage sites. *Genes Cells* 9, 549-60 (2004).
63. Waller-Evans, H. *et al.* The orphan adhesion-GPCR GPR126 is required for embryonic development in the mouse. *PLoS One* 5, e14047 (2010).
64. Freilinger, T. *et al.* Genome-wide association analysis identifies susceptibility loci for migraine without aura. *Nat Genet* 44, 777-82 (2012).
65. Frosk, P. *et al.* Limb-girdle muscular dystrophy type 2H associated with mutation in TRIM32, a putative E3-ubiquitin-ligase gene. *Am J Hum Genet* 70, 663-72 (2002).
66. Netchine, I. *et al.* Mutations in LHX3 result in a new syndrome revealed by combined pituitary hormone deficiency. *Nat Genet* 25, 182-6 (2000).
67. Wittke, I. *et al.* Neuroblastoma-derived sulfhydryl oxidase, a new member of the sulfhydryl oxidase/Quiescin6 family, regulates sensitization to interferon gamma-induced cell death in human neuroblastoma cells. *Cancer Res* 63, 7742-52 (2003).
68. Sprooten, E. *et al.* Common genetic variants and gene expression associated with white matter microstructure in the human brain. *Neuroimage* 97, 252-61 (2014).
69. Bamshad, M. *et al.* Mutations in human TBX3 alter limb, apocrine and genital development in ulnar-mammary syndrome. *Nat Genet* 16, 311-5 (1997).

70. Bogarapu, S. *et al.* Phenotype of a patient with contiguous deletion of TBX5 and TBX3: expanding the disease spectrum. *Am J Med Genet A* 164A, 1304-9 (2014).
71. Li, J. *et al.* The Anti-proliferative Function of the TGF-beta1 Signalling Pathway Involves the Repression of the Oncogenic TBX2 by its Homologue TBX3. *J Biol Chem* (2014).
72. Li, J., Weinberg, M.S., Zerbini, L. & Prince, S. The oncogenic TBX3 is a downstream target and mediator of the TGF-beta1 signaling pathway. *Mol Biol Cell* 24, 3569-76 (2013).
73. Kawaguchi, Y. *et al.* CAG expansions in a novel gene for Machado-Joseph disease at chromosome 14q32.1. *Nat Genet* 8, 221-8 (1994).
74. Albig, A.R. & Schiemann, W.P. Fibulin-5 antagonizes vascular endothelial growth factor (VEGF) signaling and angiogenic sprouting by endothelial cells. *DNA Cell Biol* 23, 367-79 (2004).
75. Loeys, B. *et al.* Homozygosity for a missense mutation in fibulin-5 (FBLN5) results in a severe form of cutis laxa. *Hum Mol Genet* 11, 2113-8 (2002).
76. Brandsma, C.A. *et al.* A large lung gene expression study identifying fibulin-5 as a novel player in tissue repair in COPD. *Thorax* 70, 21-32 (2015).
77. Dabovic, B. *et al.* Function of Latent TGFbeta Binding Protein 4 and Fibulin 5 in Elastogenesis and Lung Development. *J Cell Physiol* 230, 226-36 (2015).
78. Follit, J.A. *et al.* The Golgin GMAP210/TRIP11 anchors IFT20 to the Golgi complex. *PLoS Genet* 4, e1000315 (2008).
79. Smits, P. *et al.* Lethal skeletal dysplasia in mice and humans lacking the golgin GMAP-210. *N Engl J Med* 362, 206-16 (2010).
80. Yanagisawa, H. *et al.* Fibulin-5 is an elastin-binding protein essential for elastic fibre development in vivo. *Nature* 415, 168-71 (2002).
81. Nakamura, T. *et al.* Fibulin-5/DANCE is essential for elastogenesis in vivo. *Nature* 415, 171-5 (2002).
82. Kajiho, H. *et al.* RIN3: a novel Rab5 GEF interacting with amphiphysin II involved in the early endocytic pathway. *J Cell Sci* 116, 4159-68 (2003).
83. Albagha, O.M. *et al.* Genome-wide association identifies three new susceptibility loci for Paget's disease of bone. *Nat Genet* 43, 685-9 (2011).
84. Cho, M.H. *et al.* Risk loci for chronic obstructive pulmonary disease: a genome-wide association study and meta-analysis. *Lancet Respir Med* 2, 214-25 (2014).
85. Lambert, J.C. *et al.* Meta-analysis of 74,046 individuals identifies 11 new susceptibility loci for Alzheimer's disease. *Nat Genet* 45, 1452-8 (2013).
86. Wadehra, M. *et al.* Expression of epithelial membrane protein-2 is associated with endometrial adenocarcinoma of unfavorable outcome. *Cancer* 107, 90-8 (2006).
87. Street, V.A. *et al.* Mapping of Charcot-Marie-Tooth disease type 1C to chromosome 16p identifies a novel locus for demyelinating neuropathies. *Am J Hum Genet* 70, 244-50 (2002).
88. Cao, W., Ijiri, T.W., Huang, A.P. & Gerton, G.L. Characterization of a novel tektin member, TEKT5, in mouse sperm. *J Androl* 32, 55-69 (2011).
89. Onouchi, Y. *et al.* ITPKC functional polymorphism associated with Kawasaki disease susceptibility and formation of coronary artery aneurysms. *Nat Genet* 40, 35-42 (2008).
90. Ryan, D.M. *et al.* Smoking dysregulates the human airway basal cell transcriptome at COPD risk locus 19q13.2. *PLoS One* 9, e88051 (2014).
91. Zhong, W., Jiang, M.M., Weinmaster, G., Jan, L.Y. & Jan, Y.N. Differential expression of mammalian Numb, Numblike and Notch1 suggests distinct roles during mouse cortical neurogenesis. *Development* 124, 1887-97 (1997).
92. He, H. *et al.* Identification and characterization of nine novel human small GTPases showing variable expressions in liver cancer tissues. *Gene Expr* 10, 231-42 (2002).

93. Sterner-Kock, A. *et al.* Disruption of the gene encoding the latent transforming growth factor-beta binding protein 4 (LTBP-4) causes abnormal lung development, cardiomyopathy, and colorectal cancer. *Genes Dev* 16, 2264-73 (2002).
94. Buijs, A. *et al.* Translocation (12;22) (p13;q11) in myeloproliferative disorders results in fusion of the ETS-like TEL gene on 12p13 to the MN1 gene on 22q11. *Oncogene* 10, 1511-9 (1995).
95. Tarpey, P.S. *et al.* Mutations in the gene encoding the Sigma 2 subunit of the adaptor protein 1 complex, AP1S2, cause X-linked mental retardation. *Am J Hum Genet* 79, 1119-24 (2006).
96. Cacciagli, P. *et al.* AP1S2 is mutated in X-linked Dandy-Walker malformation with intellectual disability, basal ganglia disease and seizures (Pettigrew syndrome). *Eur J Hum Genet* 22, 363-8 (2014).
97. Saillour, Y. *et al.* Mutations in the AP1S2 gene encoding the sigma 2 subunit of the adaptor protein 1 complex are associated with syndromic X-linked mental retardation with hydrocephalus and calcifications in basal ganglia. *J Med Genet* 44, 739-44 (2007).
98. Shan, L. *et al.* Bombesin-like peptide receptor gene expression, regulation, and function in fetal murine lung. *Am J Physiol Lung Cell Mol Physiol* 286, L165-73 (2004).
99. Sunday, M.E., Choi, N., Spindel, E.R., Chin, W.W. & Mark, E.J. Gastrin-releasing peptide gene expression in small cell and large cell undifferentiated lung carcinomas. *Hum Pathol* 22, 1030-9 (1991).
100. Yoshida, K. *et al.* Frequent pathway mutations of splicing machinery in myelodysplasia. *Nature* 478, 64-9 (2011).
101. Hindorff LA, M.J., Morales J , Junkins HA, Hall PN, Klemm AK, and Manolio TA. A. Catalog of Published Genome-Wide Association Studies. Accessed 05-01-2015.
102. Golding, J., Pembrey, M., Jones, R. & Team, A.S. ALSPAC--the Avon Longitudinal Study of Parents and Children. I. Study methodology. *Paediatr Perinat Epidemiol* 15, 74-87 (2001).
103. Wilk, J.B. *et al.* Evidence for major genes influencing pulmonary function in the NHLBI family heart study. *Genet Epidemiol* 19, 81-94 (2000).
104. Lewitter, F.I., Tager, I.B., McGue, M., Tishler, P.V. & Speizer, F.E. Genetic and environmental determinants of level of pulmonary function. *Am J Epidemiol* 120, 518-30 (1984).
105. Palmer, L.J. *et al.* Familial aggregation and heritability of adult lung function: results from the Busselton Health Study. *Eur Respir J* 17, 696-702 (2001).
106. Irizarry, R.A. *et al.* Exploration, normalization, and summaries of high density oligonucleotide array probe level data. *Biostatistics* 4, 249-64 (2003).
107. Abecasis, G.R. *et al.* An integrated map of genetic variation from 1,092 human genomes. *Nature* 491, 56-65 (2012).
108. Rosenbloom, K.R. *et al.* ENCODE data in the UCSC Genome Browser: year 5 update. *Nucleic Acids Res* 41, D56-63 (2013).
109. Karolchik, D. *et al.* The UCSC Table Browser data retrieval tool. *Nucleic Acids Res* 32, D493-6 (2004).
110. Ritchie, G.R., Dunham, I., Zeggini, E. & Flicek, P. Functional annotation of noncoding sequence variants. *Nat Methods* 11, 294-6 (2014).
111. Stewart, C.E., Nijmeh, H.S., Brightling, C.E. & Sayers, I. uPAR regulates bronchial epithelial repair in vitro and is elevated in asthmatic epithelium. *Thorax* 67, 477-87 (2012).
112. Trapnell, C., Pachter, L. & Salzberg, S.L. TopHat: discovering splice junctions with RNA-Seq. *Bioinformatics* 25, 1105-11 (2009).
113. Trapnell, C. *et al.* Transcript assembly and quantification by RNA-Seq reveals unannotated transcripts and isoform switching during cell differentiation. *Nat Biotechnol* 28, 511-5 (2010).
114. Roberts, A., Pimentel, H., Trapnell, C. & Pachter, L. Identification of novel transcripts in annotated genomes using RNA-Seq. *Bioinformatics* 27, 2325-9 (2011).

115. Rogers, M.F., Thomas, J., Reddy, A.S. & Ben-Hur, A. SpliceGrapher: detecting patterns of alternative splicing from RNA-Seq data in the context of gene models and EST data. *Genome Biol* 13, R4 (2012).
116. Segrè, A.V. *et al.* Common inherited variation in mitochondrial genes is not enriched for associations with type 2 diabetes or related glycemic traits. *PLoS Genet* 6(2010).
117. Strachan, D.P. *et al.* Lifecourse influences on health among British adults: effects of region of residence in childhood and adulthood. *Int J Epidemiol* 36, 522-31 (2007).
118. Marossy, A.E., Strachan, D.P., Rudnicka, A.R. & Anderson, H.R. Childhood chest illness and the rate of decline of adult lung function between ages 35 and 45 years. *Am J Respir Crit Care Med* 175, 355-9 (2007).
119. Vitart, V. *et al.* SLC2A9 is a newly identified urate transporter influencing serum urate concentration, urate excretion and gout. *Nat Genet* 40, 437-42 (2008).
120. Zemunik, T. *et al.* Genome-wide association study of biochemical traits in Korcula Island, Croatia. *Croat Med J* 50, 23-33 (2009).
121. Rudan, I. *et al.* "10001 Dalmatians:" Croatia launches its national biobank. *Croat Med J* 50, 4-6 (2009).
122. Day, N. *et al.* EPIC-Norfolk: study design and characteristics of the cohort. European Prospective Investigation of Cancer. *Br J Cancer* 80 Suppl 1, 95-103 (1999).
123. Canoy, D. *et al.* Abdominal obesity and respiratory function in men and women in the EPIC-Norfolk Study, United Kingdom. *Am J Epidemiol* 159, 1140-9 (2004).
124. Cox, B., Huppert, F. & Whichelow, M. *The Health and Lifestyle Survey: seven years on*, (Dartmouth Publishing Company, Aldershot, United Kingdom, 1993).
125. Smith, B.H. *et al.* Cohort Profile: Generation Scotland: Scottish Family Health Study (GS:SFHS). The study, its participants and their potential for genetic research on health and illness. *Int J Epidemiol* 42, 689-700 (2013).
126. Heistaro, S. Methodology report. Health 2000 survey. in *Publications of National Public Health Institute* (ed. Heistaro, S.) (2000).
127. Kristiansson, K. *et al.* Genome-wide screen for metabolic syndrome susceptibility Loci reveals strong lipid gene contribution but no evidence for common genetic basis for clustering of metabolic syndrome traits. *Circ Cardiovasc Genet* 5, 242-9 (2012).
128. Holle, R., Happich, M., Lowel, H., Wichmann, H.E. & Group, M.K.S. KORA--a research platform for population based health research. *Gesundheitswesen* 67 Suppl 1, S19-25 (2005).
129. Wichmann, H.E., Gieger, C., Illig, T. & Group, M.K.S. KORA-gen--resource for population genetics, controls and a broad spectrum of disease phenotypes. *Gesundheitswesen* 67 Suppl 1, S26-30 (2005).
130. Miller, M.R. *et al.* Standardisation of spirometry. *Eur Respir J* 26, 319-38 (2005).
131. Peters, A. *et al.* [Multimorbidity and successful aging: the population-based KORA-Age study]. *Z Gerontol Geriatr* 44 Suppl 2, 41-54 (2011).
132. Burney, P.G., Luczynska, C., Chinn, S. & Jarvis, D. The European Community Respiratory Health Survey. *Eur Respir J* 7, 954-60 (1994).
133. Main Protocol for The European Community Respiratory Health Survey (ECRHS) I, http://www.ecrhs.org/ECRHS%20I/Main%20protocol.pdf.
134. Deary, I.J. *et al.* The Lothian Birth Cohort 1936: a study to examine influences on cognitive ageing from age 11 to age 70 and beyond. *BMC Geriatr* 7, 28 (2007).
135. Aulchenko, Y.S., Ripke, S., Isaacs, A. & van Duijn, C.M. GenABEL: an R library for genome-wide association analysis. *Bioinformatics* 23, 1294-6 (2007).
136. Martin, B.W. *et al.* SAPALDIA: methods and participation in the cross-sectional part of the Swiss Study on Air Pollution and Lung Diseases in Adults. *Soz Praventivmed* 42, 67-84 (1997).

137. Ackermann-Liebrich, U. *et al.* Follow-up of the Swiss Cohort Study on Air Pollution and Lung Diseases in Adults (SAPALDIA 2) 1991-2003: methods and characterization of participants. *Soz Praventivmed* 50, 245-63 (2005).
138. John, U. *et al.* Study of Health In Pomerania (SHIP): a health examination survey in an east German region: objectives and design. *Soz Praventivmed* 46, 186-94 (2001).
139. Volzke, H. *et al.* Reference intervals of serum thyroid function tests in a previously iodine-deficient area. *Thyroid* 15, 279-85 (2005).
140. Nelson, S.B., Gardner, R.M., Crapo, R.O. & Jensen, R.L. Performance evaluation of contemporary spirometers. *Chest* 97, 288-97 (1990).
141. Standardization of spirometry--1987 update. Statement of the American Thoracic Society. *Am Rev Respir Dis* 136, 1285-98 (1987).
142. Raitakari, O.T. *et al.* Cohort profile: the cardiovascular risk in Young Finns Study. *Int J Epidemiol* 37, 1220-6 (2008).
143. Boyd, A. *et al.* Cohort Profile: the 'children of the 90s'--the index offspring of the Avon Longitudinal Study of Parents and Children. *Int J Epidemiol* 42, 111-27 (2013).
144. Knox, J., Jarvis, D., Walter, E.H. & Committee, E.I.S. The European Community Respiratory Health Survey II. *Eur Respir J* 21, 556; author reply 556 (2003).
145. Moffatt, M.F. *et al.* A large-scale, consortium-based genomewide association study of asthma. *N Engl J Med* 363, 1211-21 (2010).
146. Chinn, S. *et al.* Smoking cessation, lung function, and weight gain: a follow-up study. *Lancet* 365, 1629-35; discussion 1600-1 (2005).
147. Lind, L., Fors, N., Hall, J., Marttala, K. & Stenborg, A. A comparison of three different methods to evaluate endothelium-dependent vasodilation in the elderly: the Prospective Investigation of the Vasculature in Uppsala Seniors (PIVUS) study. *Arterioscler Thromb Vasc Biol* 25, 2368-75 (2005).
148. Zhou, X. & Stephens, M. Genome-wide efficient mixed-model analysis for association studies. *Nat Genet* 44, 821-4 (2012).
149. Wain, L.V., . & et al. Utilising UK Biobank to generate novel insights into the genetics of lung health and disease, and smoking behaviour. *The Lancet Respiratory Medicine* Manuscript in preparation(2015).
